# Second-Generation AURKA-Targeting PROTACs: Structural Optimization toward in Vivo Degradation in Neuroblastoma

**DOI:** 10.1021/acs.jmedchem.5c01271

**Published:** 2025-11-18

**Authors:** Simon Krols, Muhammad Rishfi, Fien Martens, Anouk Van Hauwermeiren, Ellen Sanders, Pieter-Jan De Sutter, An Vermeulen, Kaat De Wever, Sarah-Lee Bekaert, M. Emmy M. Dolman, Gabor Tax, Alvin Kamili, Jamie I. Fletcher, Lisa Depestel, Bram De Wilde, Kaat Durinck, Serge Van Calenbergh

**Affiliations:** † Laboratory of Medicinal Chemistry, Department of Pharmaceutics, Faculty of Pharmaceutical Sciences, 26656 Ghent University, Ottergemsesteenweg 460, 9000 Ghent, Belgium; ‡ Pediatric Precision Oncology Lab, Department of Biomolecular Medicine, Faculty of Medicine & Health Sciences, 26656 Ghent University, Corneel Heymanslaan 10, 9000 Ghent, Belgium; § Cancer Research Institute Ghent, Corneel Heymanslaan 10, 9000 Ghent, Belgium; ∥ Laboratory of Medical Biochemistry and Clinical Analysis, Department of Bioanalysis, 26656 Ghent University, Ottergemsesteenweg 460, 9000 Ghent, Belgium; ⊥ Children’s Cancer Institute, Lowy Cancer Research Centre, UNSW Sydney, Kensington, Sydney, NSW 2052, Australia; # School of Clinical Medicine, UNSW Medicine & Health, UNSW Sydney, Kensington, Sydney, NSW 2052, Australia; ∇ Department of Internal Medicine and Pediatrics, Faculty of Medicine & Health Sciences, 26656 Ghent University, Corneel Heymanslaan 10, 9000 Ghent, Belgium

## Abstract

Aurora kinase A (AURKA) is an established oncogenic factor and therapeutic target in neuroblastoma due to its roles in mitosis and stability of the MYCN protein. We previously identified SK2188 as a highly potent, selective, and fast-acting AURKA degrader capable of inducing MYCN destabilization. However, SK2188 showed low systemic exposure and rapid clearance in mice, necessitating further chemical optimization. Here, we describe our structure–activity optimization efforts involving linker rigidification and incorporation of alternative cereblon- and AURKA-recruiting ligands, leading to two optimized PROTACs, SK4454 and SK5527. Both compounds retained rapid and selective AURKA degradation with improved pharmacokinetic properties. Importantly, single intravenous administration of either degrader efficiently reduced AURKA levels in vivo in a neuroblastoma xenograft mouse model. Moreover, MDR1-mediated PROTAC efflux was identified as a key intrinsic mechanism limiting in vitro potency. These results establish SK4454 and SK5527 as advanced AURKA degraders with improved pharmacokinetic properties, warranting further preclinical evaluation.

## Graphical abstract

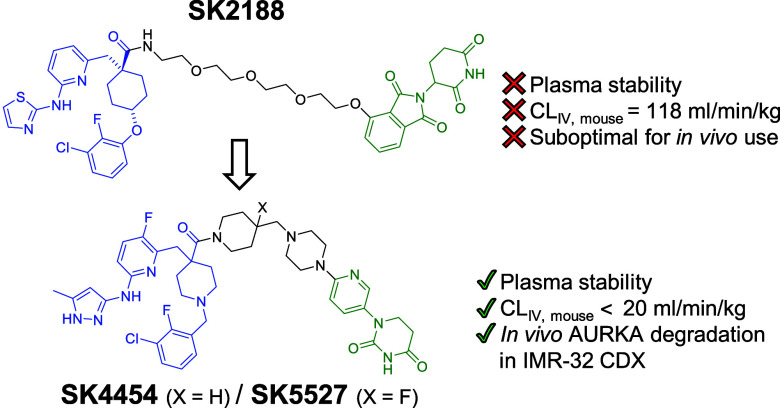

## Introduction

Neuroblastoma is the most common extracranial solid tumor in children. Despite intensive multimodal therapies, including surgery, chemotherapy, radiotherapy, immunotherapy, and stem cell transplantation, half of all high-risk patients ultimately succumb to their disease, underscoring the urgent need for novel therapeutic strategies.^1^ While the exact origin and mechanism of neuroblastoma formation remain unclear, amplification of the *MYCN* oncogene is one of the strongest markers for high-risk disease and poor overall survival.^2–4^ However, direct inhibition of MYCN remains a significant challenge due to the absence of a well-defined binding pocket.^5^

Aurora kinase A (AURKA) is a serine/threonine kinase that plays a critical role in centrosome maturation, spindle assembly, and mitotic entry through its catalytic activities. Dysregulation of AURKA has been implicated as a key oncogenic driver across multiple cancer types, making it an attractive therapeutic target for which several selective ATP-competitive inhibitors have already been developed, including MK-5108, LY3295668, and TAS-119 ^6–8^ (Figure 1A). In neuroblastoma, AURKA initially emerged as a potential drug target due to its kinase-independent role in binding and stabilizing the MYCN oncoprotein.^9^ More specifically, AURKA directly interacts with MYCN and prevents its recognition by the E3 ubiquitin ligase SCF^FBXW7^, thereby protecting MYCN from proteasomal degradation.^10^ This AURKA/MYCN complex formation was also shown to play a crucial role in mitigating transcription-replication conflicts during S-phase through phosphorylation of CDK12.^11,12^ Another study revealed additional kinase-independent functions of AURKA, regulating DNA damage repair and replication fork stability in complex with TPX2.^13^ Given these diverse functions, there is a growing interest in developing AURKA degraders, using proteolysis-targeting chimeras (PROTACs), which have the unique potential to simultaneously target both kinase-dependent and -independent activities.^14–22^

**Figure 1:**
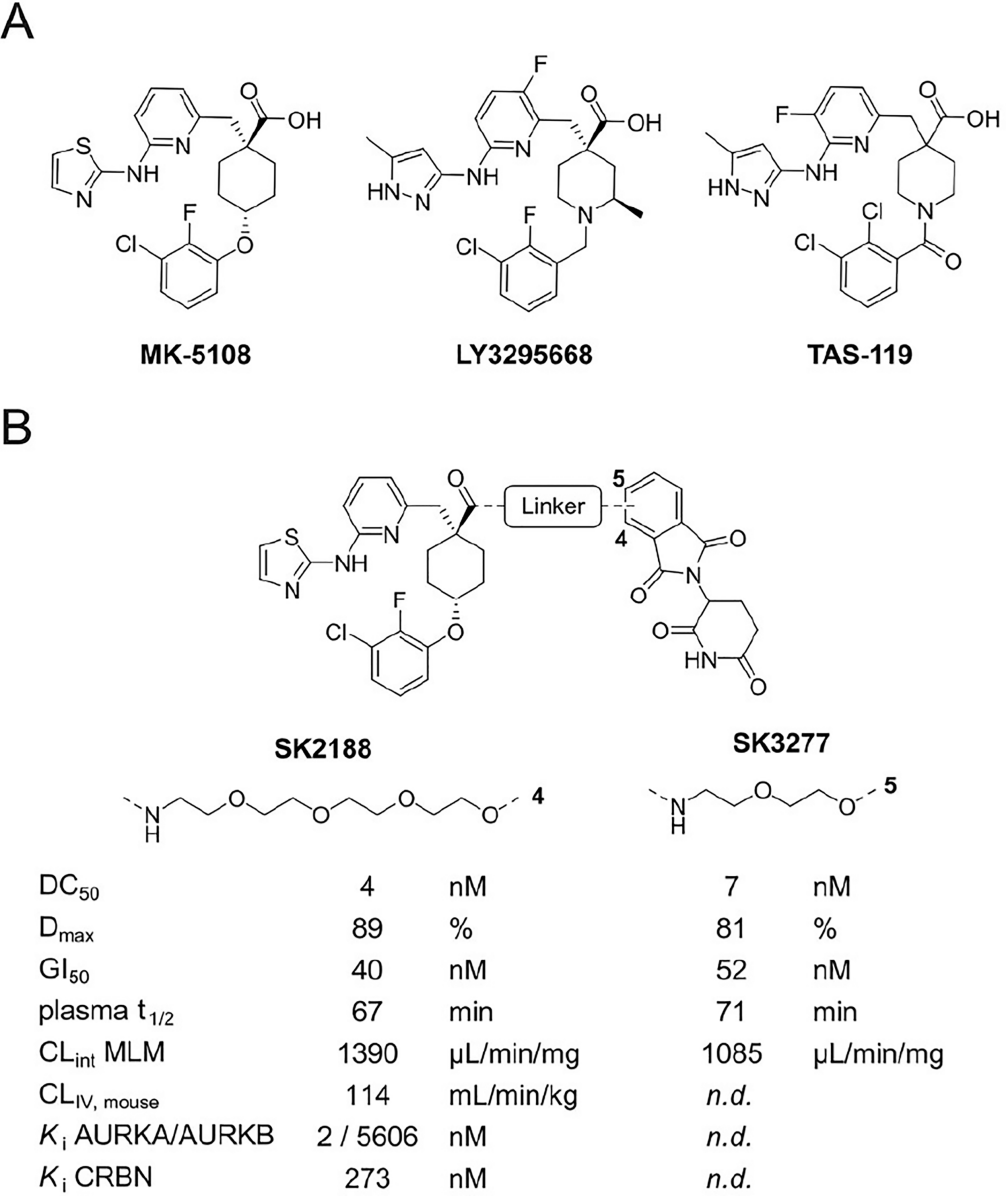
(A) Chemical structures of selective AURKA inhibitors MK-5108, LY3295668, and TAS-119. (B) Previously developed AURKA degraders SK2188 and SK3277 generated by coupling MK-5108 to thalidomide using PEG-based linkers of varying lengths.^18^ Both PROTACs exhibited potent biological activities (DC_50_, *D*_max_, GI_50_) and, in the case of SK2188, a high binding selectivity for AURKA over AURKB (*K*_i_ AURKA/AURKB) and confirmed binding with CRBN (*K*_i_ CRBN). However, both PROTACs showed low plasma stabilities (plasma *t*_1/2_), high turnover rates in mouse liver microsomes (CL_int_ MLM), and in the case of SK2188, a high intravenous clearance (CL_IV_) (n.d. = not determined).

Unlike classical inhibitors, which rely on sustained target binding to maintain efficacy, PROTACs induce short proximity interactions between an E3 ligase and a target protein, triggering ubiquitination and proteasomal degradation of the latter.^23^ We previously synthesized potent AURKA-degrading PROTACs by connecting the highly selective AURKA inhibitor MK-5108 with the cereblon (CRBN) E3-ligase-recruiting ligand thalidomide via PEG-based linkers of varying lengths (Figure 1B).^18^ The PEG4-based PROTAC **SK2188** demonstrated highly potent, rapid, and selective AURKA degradation in vitro, which was associated with reduced MYCN levels and increased replication stress, DNA damage, and apoptosis in NGP, a *MYCN* amplified neuroblastoma cell line. We also found that PROTAC **SK3277**, featuring a shorter PEG2 linker attached to the 5-position of thalidomide, was tolerated and still resulted in potent AURKA degradation. However, further analyses revealed that **SK2188** and **SK3277** were unstable in murine plasma, liver microsomes, and hepatocytes, limiting their potential for in vivo use (Figure 1B). In this study, we report the development of novel AURKA-targeting PROTACs with enhanced pharmacokinetic (PK) properties by optimizing the linker and exploring the use of alternative CRBN- and AURKA-recruiting ligands.

## Results and Discussion

### Development of Optimized AURKA PROTACs with Improved Pharmacokinetics

Our previously developed AURKA degraders **SK2188** and **SK3277** ^18^ were unsuitable for in vivo applications due to their low plasma stability, high turnover rates in mouse liver microsomes (MLM) and, in the case of **SK2188**, a high intravenous clearance (CL_IV_) (Figure 1B). To address this, we initiated a structural optimization study to generate novel AURKA degraders with improved pharmacokinetic properties. Building on our previous work, in which we identified CRBN as a suitable E3 ligase for efficient AURKA degradation,^18^ we continued to develop CRBN-recruiting PROTACs while concentrating on structural optimization of the linker, AURKA-binding moiety, and CRBN ligand. In total, we synthesized and screened 38 novel PROTACs, categorized into 4 series based on different optimization strategies: (1) linker rigidification, (2) exploration of arylaminoglutarimide- and (3) dihydrouracil-based CRBN ligands, and (4) the use of a LY3295668-like AURKA ligand. All compounds were screened in vitro using the *MYCN* amplified neuroblastoma cell line NGP. We used IncuCyte live-cell imaging to measure antiproliferative effects (GI_50_) (Supplementary Figure S1) and Simple Western to monitor reduction in AURKA protein levels following 24h treatment with 0.1 and 1 μM (Supplementary Figure S2A). For promising candidates, we more accurately assessed their degradation potency (DC_50_, *D*_max_) (Simple Western) (Supplementary Figure S2B,C) and monitored their stability in plasma (plasma *t*_1/2_), mouse liver microsomes (CL_int_ MLM), and mouse hepatocytes (CL_int_ MH) using Eurofins Discovery Services. A summary of the chemical structures and biological data for all synthesized PROTACs can be found in Supplementary Tables S1–S4, while key compounds are highlighted in Figure 2.

**Figure 2:**
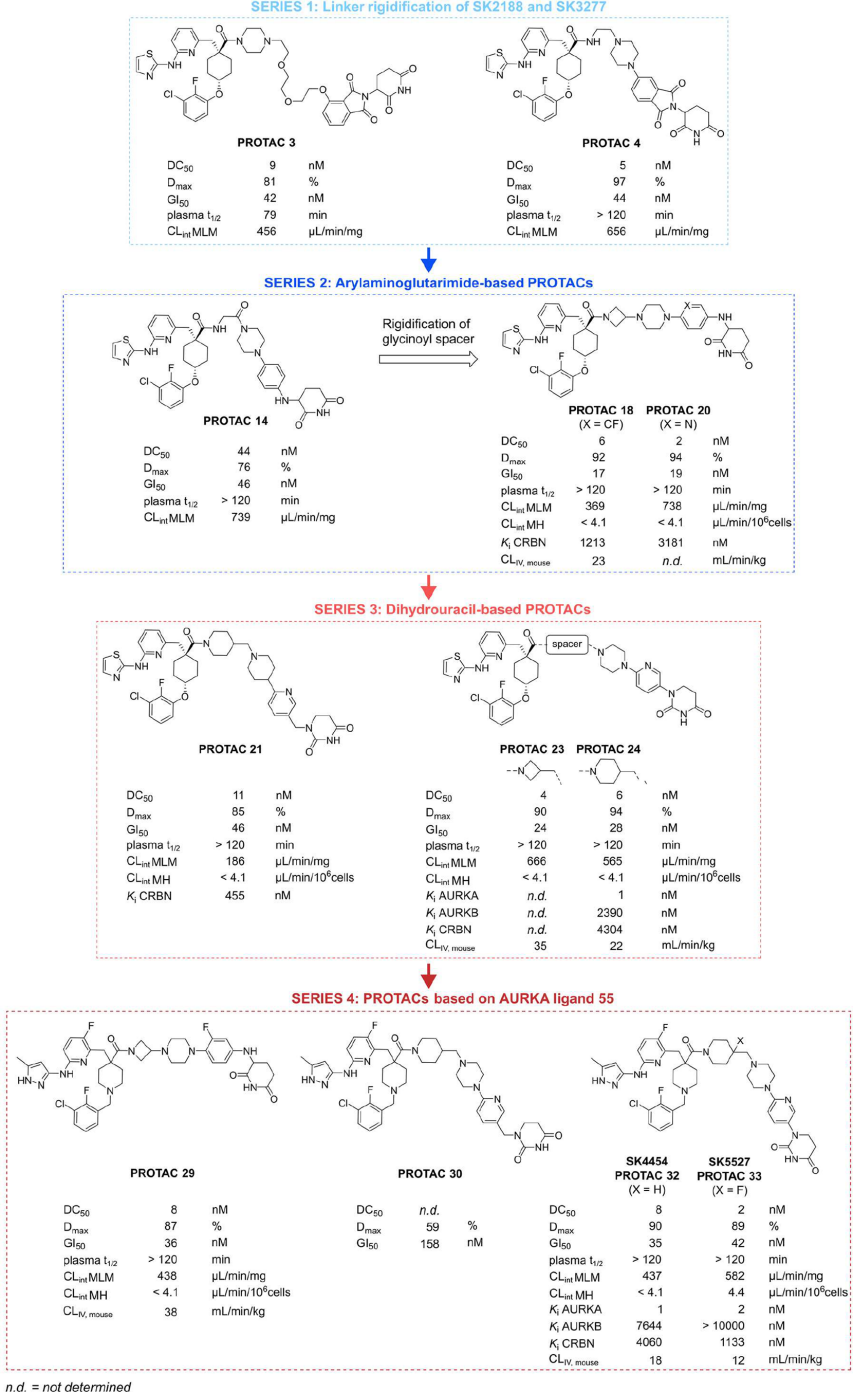
Key compounds developed during the PROTAC structural optimization study and their corresponding in vitro and in vivo parameters. Key compounds are categorized into series based on different optimization strategies: (*series 1*) linker rigidification, (*series 2*) exploration of arylaminoglutarimide- and (*series 3*) dihydrouracil-based CRBN ligands, and (*series 4*) the use of a new AURKA ligand analogous to LY3295668. Antiproliferative effects were monitored using IncuCyte live-cell imaging in NGP and expressed as GI_50_ 72h after treatment (*n* = 3) (see also Supplementary Figure S1). AURKA degradation potency was assessed using Simple Western after 24 h treatment in NGP and expressed as DC_50_ and *D*_max_ (*n* = 3) (see also Supplementary Figure S2B). Plasma half-life (*t*_1/2_) and metabolic stability (CL_int_) were measured by Eurofins Discovery services through incubation of the compounds in mouse plasma, mouse liver microsomes (MLM), or mouse hepatocytes (MH). The percentage of the compound remaining over time was measured by LC–MS analysis (*n* = 2 replicate samples). Binding to AURKA, AURKB, and CRBN was measured using the KINOMEscan or E3scan profiling services by Eurofins Discovery and expressed as *K*_i_ (*n* = 2 replicate samples) (see also Supplementary Figure S3). In vivo systemic clearance (CL_IV_) was obtained by a noncompartmental analysis following IV administration at a dose of 15 mg/kg in mice (*n* = 3 mice) (see also Supplementary Figure S4). Data for all synthesized compounds can be found in Supplementary Tables 1–4.

With the development of novel PROTACs, we aimed to enhance plasma and metabolic stability while retaining degradation potency. Given that reducing linker flexibility has been shown to improve metabolic stability,^24^ we decided to rigidify the linker in degraders **SK2188** & **SK3277** by incorporation of a piperazine ring at either end of the linker which proved to be largely tolerated (Figure 2, **series 1** and Supplementary Table S1). Among the PROTACs in series 1, piperazine-substituted thalidomide PROTAC **4** exhibited the most potent AURKA degradation, with a DC_50_ of 5 nM, along with improved plasma stability. Additionally, both piperazine-containing PROTACs **3** and **4** had lower CL_int_ MLM values compared to **SK2188**, indicating reduced metabolic turnover.

Next, we sought to overcome common challenges associated with thalidomide, including stability issues in aqueous media, racemization of the glutarimide moiety, and the unintended degradation of multiple zinc-finger-containing transcription factors.^25,26^ Therefore, we explored alternative CRBN ligands starting with several arylaminoglutarimides^27–30^ (Figure 2, **series 2** and Supplementary Table S2). A bifunctional molecule featuring only a piperazine linker between MK-5108 and the arylaminoglutarimide (PROTAC **12**) failed to reduce AURKA levels and instead led to its upregulation—an effect commonly observed with classical AURKA inhibitors.^31^ Constructs with longer linkers (PROTACs **15** and **16**) showed only weak AURKA-degrading activity. In contrast, the introduction of a glycine spacer between MK-5108 and the piperazine moiety (PROTAC **14**) resulted in significantly stronger degradation activity. Further rigidification of the glycine spacer led to azetidine PROTACs **18** and **20**, which showed DC_50_ values of 6 nM and 2 nM, respectively.

Subsequent optimization efforts focused on eliminating the stereocenter in the glutarimide moiety of PROTACs **18**/**20** by swapping the aniline nitrogen with the stereogenic carbon, resulting in dihydrouracil-based CRBN ligands (Figure 2, **series 3** and Supplementary Table S3). This modification also removes an H-bond donor, which may enhance permeability and oral absorption.^32^ The resulting 1-(pyridin-3-ylmethyl)­dihydrouracil-based PROTAC **21** exhibited slightly reduced degradation potency compared to **SK2188**. Additionally, we explored analogues in which the dihydrouracil was directly attached to the pyridyl ring by removing the methylene bridge. Applying this to PROTAC **21** restored degradation activity, as seen with PROTACs **23** and **24**.

Finally, we explored the integration of alternative AURKA ligands. We synthesized the simplified LY3295668 analogue **55** (see Chemistry section, Scheme 1) and coupled it to the previously developed arylaminoglutarimide- and dihydrouracil-based CRBN ligands using a plug-and-play strategy (Figure 2, **series 4**, and Supplementary Table S4). Among these, the 1-(pyridin-3-ylmethyl)­dihydrouracil analogue PROTAC **30** exhibited weak degradation potency. In contrast, fluorophenylaminoglutarimide-based PROTAC **29** and 1-(6-(piperazin-1-yl)­pyridin-3-yl)­dihydrouracil-based PROTACs **32** and **33** were potent AURKA degraders, with DC_50_ values in the single-digit nanomolar range. Kinase binding assays using the KINOMEscan technology^33^ demonstrated that PROTACs **32** and **33** strongly bind AURKA with similar *K*i values as the MK-5108-based PROTAC **24**, with which they share an identical/similar linker (Supplementary Figure S3A). Additionally, just like PROTAC **24** and **SK2188**, PROTACs **32** and **33** selectively bind AURKA over AURKB with more than 2000-fold selectivity, demonstrating a superior selectivity profile compared to inhibitor MK-5108. Notably, while the degradation potencies of the novel series 2, 3, and 4 degraders are comparable to **SK2188**, their affinity for CRBN is 2 to 16 times weaker (Supplementary Figure S3B). However, they exhibit improved plasma and metabolic stability, with CL_int_ values in mouse liver microsomes (MLM) ranging from 186 to 1061 μL/min/mg and most of them showing full stability in mouse hepatocytes (CL_int_ MH < 4.1 μL/min/10^6^ cells) (Figure 2 and Supplementary Tables S1–S4).

To demonstrate that our optimization efforts led to PROTACS with an improved PK profile, we compiled a structurally diverse panel of the most potent degraders from series 2, 3, and 4 and subjected them to an in vivo mouse PK study, comparing their PK-parameters to **SK2188**. PROTACs **18**, **23**, **24**, **29**, **32** and **33** were selected and administered intravenously at a dose of 15 mg/kg (Figure 2, and Supplementary Figure S4). Notably, all tested PROTACs exhibited significantly reduced intravenous (IV) clearance compared to **SK2188**, with PROTACs **32** (**SK4454**) and **33** (**SK5527**) displaying the lowest IV clearances (18.1 and 12.2 mL/min/kg, respectively). Based on their overall biological profiles, we selected these PROTACs for further characterization. A summary of their key in vitro ADME characteristics is provided in Supplementary Table S5.

### SK4454 and SK5527 are Rapid, Potent, and Selective AURKA Degraders

To confirm AURKA engagement in cells, we employed a NanoBRET (Nanoluciferace Bioluminescence Resonance Energy Transfer) assay^34^ (Supplementary Figure S5). In permeabilized cell lysates, **SK4454** and **SK5527** displaced the tracer from NanoLuc-AURKA with an IC_50_ of ∼20 nM. The inhibitors MK-5108, LY3295668 and TAS-119 are at least 10 times more potent, consistent with kinase binding data (Supplementary Figure S3A). **SK4454** and **SK5527** were also able to bind NanoLuc-AURKA in intact cells, confirming good cellular uptake and in-cell AURKA binding. Furthermore, MK-5108 and LY3295668 displayed stronger AURKA engagement than TAS-119 in intact cells, suggesting that TAS-119 has the lowest permeability among the tested AURKA inhibitors.

Next, we assessed the AURKA degradation potential of **SK4454** and **SK5527** across an extended panel of neuroblastoma cell lines using Simple Western. Both PROTACs induced comparable AURKA and concomitant MYCN degradation (Figure 3A,B and Supplementary Figure S6A), with the strongest effects seen in *MYCN* amplified IMR-32, NGP and SJNB-8, all exhibiting AURKA DC_50_ values below 25 nM. Degradation was less pronounced in *MYCN* amplified SK-N-BE(2)-C and in *MYCN* nonamplified SK-N-AS and SJNB-1, with a substantial hook effect observed in SK-N-AS (Supplementary Figure S6B). Not surprisingly, potent AURKA degradation was also observed in the benign immortalized cell lines HEK293T and RPE (Supplementary Figure S6B).

**Figure 3:**
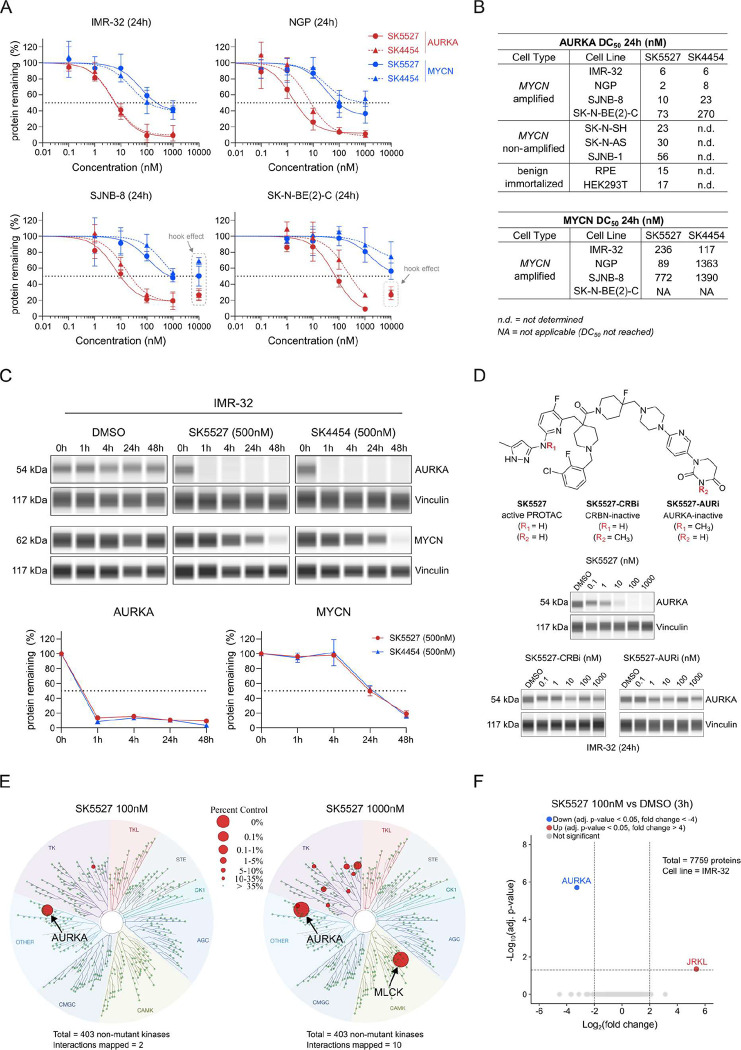
(A) Dose–response curves depicting percent change in AURKA (red) and MYCN (blue) protein levels relative to a DMSO control in MYCN amplified neuroblastoma cell lines, as measured by Simple Western after 24h treatment with SK5527 or SK4454 (mean ± SD) (*n* = 3) (see also Supplementary Figure S6A). (B) Table with AURKA and MYCN DC_50_ values across MYCN amplified and nonamplified neuroblastoma, as well as benign immortalized cell lines, as derived from their respective dose–response curves (see also Supplementary Figure S6B). (C) (Top) Representative Simple Western lane view showing changes in AURKA and MYCN protein levels over time following treatment with DMSO, SK5527 (500 nM), or SK4454 (500 nM) in IMR-32, using vinculin as the loading control. (Bottom) Linear plots showing the quantification of Simple Western data showing percent protein remaining relative to the DMSO control of their corresponding time points (mean ± SD) (*n* = 2). (D) Chemical structures of SK5527 and its inactive analogues SK5527-CRBi and SK5527-AURi, along with representative Simple Western lane views showing AURKA protein levels following a 24h treatment in IMR-32, using vinculin as the loading control (*n* = 3). (E) KINOMEscan TREEspot map of nonmutant kinases (total = 403), representing the binding-selectivity profile of SK5527 at 100 and 1000 nM. (F) LC–MS/MS proteome profiling of IMR-32 following 3h treatment with SK5527 (100 nM) (*n* = 4) (data for 24h time point included in Supplementary Figure S6F).

To further characterize degradation kinetics, we performed a time-resolved analysis (1, 4, 24, and 48 h) of AURKA degradation and MYCN destabilization.^8^ Both **SK4454** and **SK5527** induced robust AURKA depletion already after 1 h, whereas MYCN codepletion reached ∼50% only at 24h postexposure (Figure 3C). This temporal delay and reduced magnitude of MYCN depletion compared to AURKA are consistent with MYCN loss being a secondary consequence of AURKA degradation rather than a direct effect of the PROTAC. Interestingly, the recently described AURKA inhibitor TAS-119 has also been reported to destabilize MYCN,^8^ a phenomenon observed with several AURKA inhibitors (e.g., MLN-8054, Alisertib, CD532) and attributed to inhibitor-induced conformational changes that disrupt AURKA–MYCN binding.^10,35,36^ In line with this, we observed TAS-119 to induce MYCN destabilization to a similar extent as PROTACs **SK4454** and **SK5527** (Supplementary Figure S6C), although the underlying mechanisms remain to be clarified.

Next, to confirm the mechanism of action, we synthesized two methylated analogues of SK5527 designed to disrupt binding to either CRBN (**SK5527-CRBi**) or AURKA (**SK5527-AURi**). As expected, both control compounds failed to degrade AURKA (Figure 3D), consistent with their inability to bind CRBN or AURKA, as confirmed by kinase-binding assays (Supplementary Figure S3). In addition, both inactive analogues failed to destabilize MYCN, indicating that the inhibitor component of the PROTACs alone is not sufficient to elicit MYCN depletion (Supplementary Figure S6D). Furthermore, competition binding assays through cotreatment with thalidomide, MK-5108, or the proteasome inhibitor MG-132 rescued AURKA degradation, demonstrating that the observed degradation induced by **SK4454** and **SK5527** requires both AURKA and CRBN binding, as well as proteasomal activity (Supplementary Figure S6E).

We further evaluated target selectivity by performing a kinome-wide binding scan (Figure 3E). At 100 nM of **SK5527**, only AURKA and TrKA (Tropomyosin receptor kinase A) were significantly engaged. At 1000 nM, only 9 out of 403 nonmutant kinases, in addition to AURKA, were bound, indicating excellent kinome-wide selectivity (Figure 3E). Additionally, mass spectrometry-based proteomics confirmed an exceptional degradation selectivity profile, with AURKA as the only significantly downregulated protein after 3h of treatment (Figure 3F). A broader impact on differential protein expression was observed at 24 h (Supplementary Figure S6F), including the upregulation of PLK1, a known AURKA downstream target, and the downregulation of HAND2 and ESCO2, which are linked to the neuroblastoma core transcriptional regulatory circuitry and DNA damage regulation, respectively.^37–39^ Notably, reliably quantified CRBN neosubstrates, including GSPT1, CSNK1A1, ZFP91, SALL4, ZNF654, ZNF787, E4F1, and PATZ1 were not significantly downregulated (Supplementary Figure S6F).^25,26^ Overall, these findings establish **SK4454** and **SK5527** as high-quality degrader probes,^40,41^ demonstrating rapid and potent AURKA degradation, with high target selectivity confirmed for **SK5527.**

### SK4454 and SK5527 Demonstrate Strong Antiproliferative Activity in Neuroblastoma in Vitro Cell Lines

Next, we used IncuCyte live-cell imaging to assess the antiproliferative effects of PROTACs **SK4454** and **SK5527** across a panel of neuroblastoma cell lines and compared these to **SK2188** and the AURKA inhibitors TAS-119, LY3295668 and MK-5108. (Figure 4A, and Supplementary Figure S7A). At 72h postexposure with **SK4454** and **SK5527**, the strongest antiproliferative effects were seen in IMR-32, NGP and SJNB-8 with GI_50_ values below 100 nM, consistent with the observed potent AURKA degradation (Figure 4B). **SK4454** and **SK5527** clearly outperformed MK-5108 in IMR-32, NGP and SJNB-8 and showed similar potency in the other neuroblastoma cell lines tested. In contrast, compared to LY3295668 and TAS-119, **SK4454** and **SK5527** exhibited comparable antiproliferative effects in IMR-32, NGP and SJNB-8 but were less potent in SK-N-SH, SK-N-BE(2)-C and SJNB-1. Of note, among the three inhibitors, TAS-119 and LY3295668 were the most potent with comparable GI_50_ values while MK-5108 was the least effective at inhibiting neuroblastoma proliferation despite having the strongest AURKA binding affinity, as previously demonstrated by the NanoBRET assay (Supplementary Figure S5). Furthermore, both PROTACs were generally more effective than **SK2188** at inhibiting neuroblastoma cell growth (Figure 4B).

**Figure 4:**
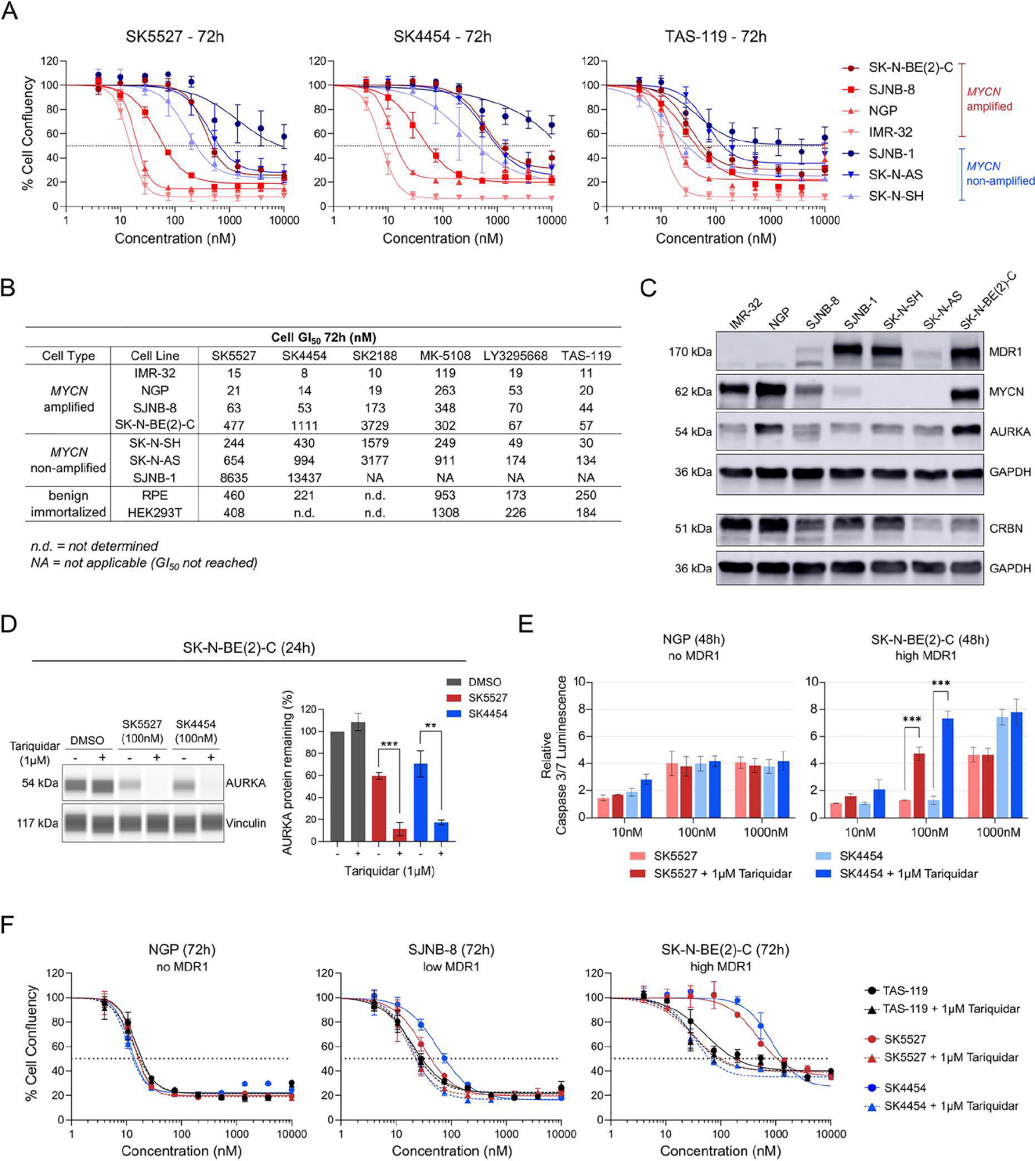
(A) Dose–response curves representing percent cell confluency relative to a DMSO control as measured by IncuCyte live-cell imaging at 72h after treatment with SK5527, SK4454, and TAS-119 (mean ± SD) (*n* = 3). (B) Table with cell GI_50_ values across cell lines as derived from their respective dose–response curves (see also Supplementary Figure S7A,B). (C) Representative Western blot showing levels of endogenous proteins MDR1, MYCN, AURKA, and CRBN in untreated cell lines, using GAPDH as the loading control (*n* = 3) (quantified data included in Supplementary Figure S7D). (D) Representative Simple Western lane view and quantification of SK-N-BE(2)-C treated with SK5527 or SK4454 (100 nM) for 24h with and without addition of tariquidar (1 μM), using vinculin as the loading control. Bars represent percent AURKA levels relative to a DMSO control (mean ± SD) (*n* = 3). Statistical significance was calculated using unpaired *t* tests (**p ≤ 0.01, ***p ≤ 0.001). (E) Caspase-Glo 3/7 assay showing induction of apoptosis in NGP and SK-N-BE(2)-C following 48 h treatment with SK5527 or SK4454, either alone or combined with tariquidar (1 μM). Bars represent caspase 3/7 luminescence relative to a DMSO control (mean ± SD) (*n* = 3). Statistical significance was calculated using multiple unpaired *t* tests with correction for multiple testing using the Bonferroni–Dunn method (blank = nonsignificant, ***p ≤ 0.001). (F) Dose–response curves representing percent cell confluency relative to a DMSO control in NGP, SJNB-8, and SK-N-BE(2)-C as measured by IncuCyte live-cell imaging at 72 h, following treatment with TAS-119, SK5527, or SK4454, with or without the addition of tariquidar (1 μM) (mean ± SD) (*n* = 3).

Notably, PROTAC **SK5527** exhibited only modest inhibitory effects in the benign immortalized cell lines HEK293T and RPE, despite potent AURKA degradation, with GI_50_ values of approximately 0.5 μM (Figure 4B, and Supplementary Figure S7B). Among the inactive control compounds, **SK5527-CRBi**, which retains AURKA binding but cannot recruit CRBN, displayed modest antiproliferative activity in IMR-32 (GI_50_ ≈ 0.5 μM), likely reflecting residual AURKA inhibition. In contrast, **SK5527-AURi**, which can bind CRBN but not AURKA, showed nearly complete inactivity in IMR-32 (GI_50_ ≈ 9 μM). Together, these results demonstrate that the superior phenotypic effects of PROTACs **SK4454** and **SK5527** are due to the protein degradation of AURKA (Supplementary Figure S7C).

### MDR1-Mediated Drug Efflux is a Key Limiting Factor for SK4454 and SK5527 Sensitivity in Vitro

Drug efflux pumps have previously been identified as key factors limiting PROTAC sensitivity in cancer cells.^42,43^ We therefore hypothesized that the reduced activity of **SK4454** and **SK5527** in SK-N-BE(2)-C, SK-N-SH, and SJNB-1 (Figures 3A and 4A) was due to drug efflux, as these cell lines exhibit high expression of multidrug resistance protein 1 (MDR1), encoded by the *ABCB1* gene (Figure 4C, and Supplementary Figure S7D). In an initial experiment, we observed stronger AURKA degradation in SH-SY5Y cells with a stable shRNA-mediated *ABCB1* knockdown compared to wild-type cells following treatment with 1 μM **SK2188**, which was accompanied by an almost 10x fold decrease in GI_50_ (Supplementary Figure S7E,F). This finding prompted us to evaluate the AURKA degradation potential of our novel PROTACs in the presence of the MDR1 inhibitor tariquidar. In SK-N-BE(2)-C, which exhibits the highest MDR1 expression in our panel, cotreatment with 1 μM tariquidar significantly enhanced the AURKA degradation potency of both **SK4454** and **SK5527** (Figure 4D). This effect was accompanied by a significant increased induction of apoptosis in SK-N-BE(2)-C, while no difference was observed in NGP, which lack MDR1 expression (Figure 4E). In contrast, none of the AURKA inhibitors showed increased apoptotic activity upon cotreatment with tariquidar in the high MDR1-expressing cell line SK-N-BE(2)-C, suggesting that they are not affected by the MDR1 drug efflux pump (Supplementary Figure S7G). In addition, overall, we see a clear dose-dependent increase of caspase 3/7 activity with PROTAC **SK4454** and **SK5527**, suggesting that the previously observed antiproliferative effects are associated with increased apoptotic activity (Figure 4E).

Finally, in a head-to-head comparison, when drug efflux was eliminated by cotreatment with tariquidar, **SK4454** and **SK5527** exhibited growth-inhibitory activity comparable to AURKA inhibitor TAS-119, with nearly identical GI_50_ values across cell lines (Figure 4F). The only exception was SK-N-AS, where TAS-119 still outperformed the PROTACs despite the low MDR1 expression in this cell line (Supplementary Figure S7H). We hypothesize that this may be due to the low CRBN expression in SK-N-AS (Figure 4C and Supplementary Figure S7D), a previously described major bottleneck in the activity of CRBN-based PROTACs.^44^

### SK4454 and SK5527 Can Induce AURKA Protein Depletion in Vivo

As previously shown, both **SK4454** and **SK5527** showed reduced clearance in mice after IV administration compared to our previously reported PROTAC SK2188. Additionally, we conducted mouse PK studies following IP (intraperitoneal) and PO (per oral) administration for both degraders. The IP profiles of both PROTACs were highly similar to their IV profiles (Figure 5A). The oral bioavailability was relatively low, at 7% for **SK4454** and 11% for **SK5527** (Supplementary Figure S8A,B), which may be partly explained by their susceptibility to MDR1-mediated efflux, given that high transporter expression in the intestinal epithelium has been shown to limit compound absorption following oral administration.^45^ Overall, both **SK4454** and **SK5527** exhibited a suitable PK profile in mice, with free drug levels remaining above the DC_50_ value for approximately 6–8 h at a dose of 15 mg/kg following IV and IP administration. Given that AURKA degradation with these PROTACs already occurs at 1 h after treatment in vitro, we hypothesized that this short exposure could be sufficient to induce AURKA degradation in vivo.

**Figure 5:**
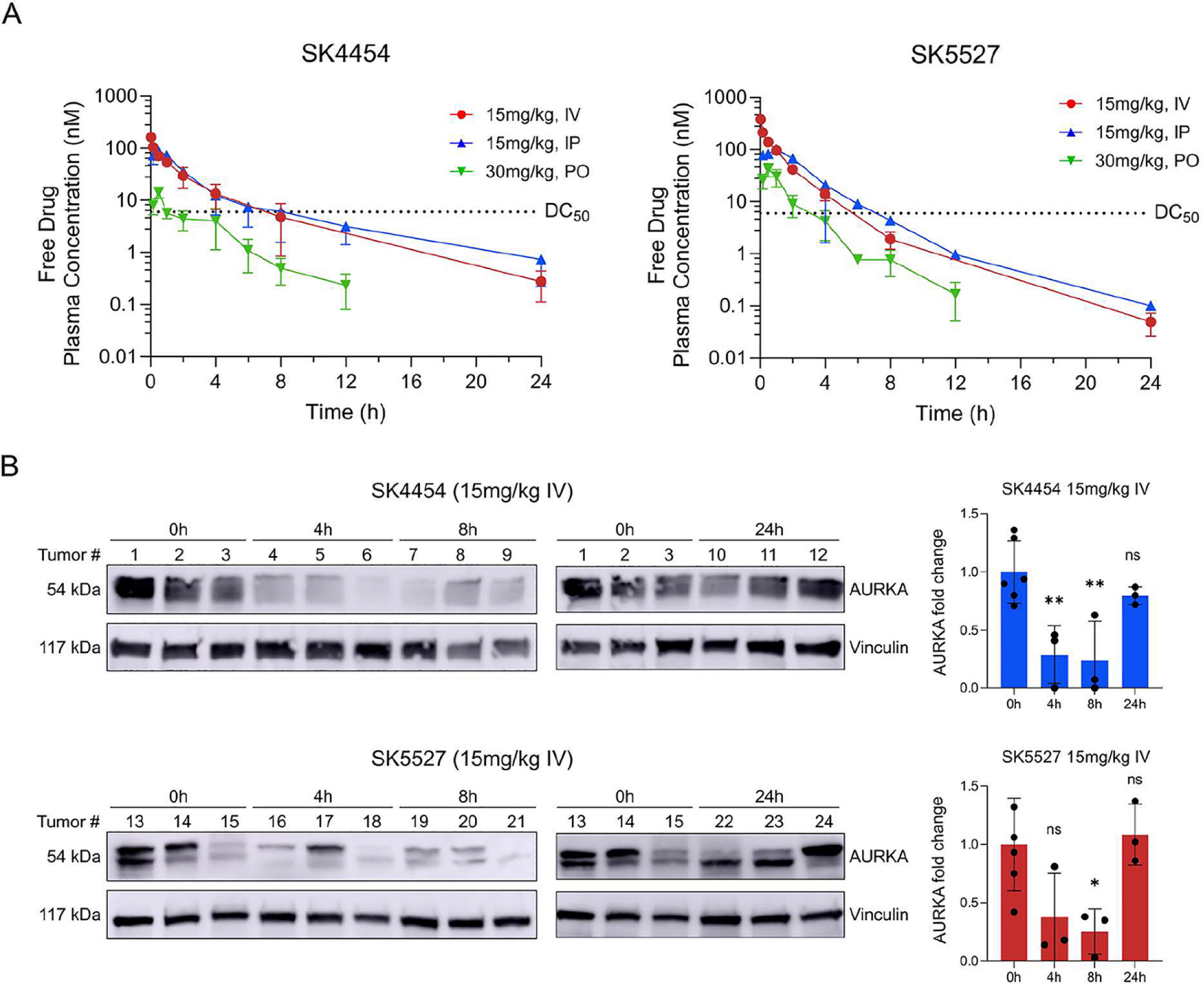
(A) In vivo PK curves depicting free drug plasma concentrations of PROTACs SK4454 and SK5527 over time, following a single IV (15 mg/kg), IP (15 mg/kg), and PO (30 mg/kg) administration in mice (mean ± SD) (*n* = 3 mice) (see also Supplementary Figure S8A,B for total drug PK curves along with key PK parameters). Dotted lines represent the in vitro DC_50_ values of each PROTAC in IMR-32. (B) Pharmacodynamic study of SK4454 and SK5527 in IMR-32 cell line-derived xenograft mice. Mice tumors were harvested at different time points following a single IV administration (15 mg/kg). Changes in AURKA protein levels were visualized using Western blot, with vinculin as a loading control. Bars represent the quantified Western blot data, representing AURKA fold change relative to the 0 h controls (mean ± SD) (*n* = 3 mice per time point). Statistical significance was calculated using an ordinary one-way ANOVA with Dunnett’s correction for multiple testing (ns = not significant, *p ≤ 0.05, **p ≤ 0.01).

To test this, **SK4454** or **SK5527** were administered (15 mg/kg, IV and IP) in IMR-32 neuroblastoma cell line-derived xenograft (CDX) mice. Tumor tissues were subsequently harvested at 4, 8, and 24 h after administration, and AURKA protein levels were visualized and compared to baseline levels at *t* = 0 h. Both PROTACs strongly reduced AURKA protein levels in vivo at 4 and 8 h post-treatment after a single IV dose, followed by a return to baseline levels at 24 h (Figure 5B). In contrast, these strong pharmacodynamic effects were not observed after a single IP dose (Supplementary Figure S8C), despite both routes showing highly comparable overall exposure. This discrepancy may be explained by the fact that free drug concentrations measured in plasma do not necessarily reflect those achieved within tumor tissue.^46^ Moreover, the higher initial plasma concentration (*C*_0_) achieved following IV administration, which is absent after IP dosing (Figure 5B, Supplementary Figure S8A,B), may help explain the stronger in vivo AURKA degradation observed in our study, as this transient peak could facilitate more rapid intracellular target engagement in tumors. This hypothesis, however, requires further investigation.

Overall, while our results demonstrate the strong in vivo degradation potential of both PROTACs following IV administration, further work is required to determine the minimum dose needed to achieve comparable AURKA degradation after IP administration. We also anticipate that repeated dosing (e.g., twice daily) may be necessary to sustain target depletion.

## Conclusions

Our previously reported AURKA-degrader, PROTAC **SK2188**, was suboptimal for further in vivo applications due to its poor plasma and metabolic stability and high in vivo clearance. In this optimization study, we report novel AURKA-targeting PROTACs with significantly improved pharmacokinetic profiles. By applying more rigid linkers, as well as exploring alternative CRBN- and AURKA-recruiting ligands, we discovered PROTACs **SK4454** and **SK5527**, which demonstrated superior plasma and metabolic stability, as well as reduced intravenous clearance compared to **SK2188.**

Both **SK4454** and **SK5527** retained potent and selective AURKA degradation capacity, effectively reducing MYCN levels in *MYCN* amplified neuroblastoma cells. Additionally, our investigations revealed that MDR1-mediated efflux may limit **SK4454** and **SK5527** activity, highlighting opportunities for further chemical modifications to reduce susceptibility to drug efflux and improve PROTAC activity and bioavailability.

Pharmacodynamic studies in a neuroblastoma xenograft mouse model confirmed that both SK4454 and SK5527 efficiently degraded AURKA in vivo following a single intravenous dose of 15 mg/kg. However, a single intraperitoneal dose at the same concentration did not result in detectable AURKA degradation, despite comparable systemic exposure. This difference may be attributed to the higher initial plasma concentration (*C*_0_) achieved after IV administration, which could facilitate faster and more effective target engagement in tumors. Further studies are needed to determine whether higher or repeated IP dosing can achieve comparable AURKA degradation in vivo to enable further preclinical efficacy and safety evaluation.

Overall, our results highlight that while rational design principles can guide PROTAC optimization and overcome key pharmacokinetic challenges, the discovery process ultimately remains highly empirical. Nonetheless, the optimized compounds **SK4454** and **SK5527** presented here provide a strong foundation for continued preclinical studies aimed at validating AURKA degradation as a therapeutic strategy in neuroblastoma and other AURKA-driven malignancies.

## Chemistry

Synthetic schemes of all final compounds can be found in the Supporting Information. In Scheme 1, we highlight the synthetic route toward PROTACs **SK4454** and **SK5527**. The synthesis of the 1-(6-(piperazin-1-yl)­pyridin-3-yl)­dihydrouracil-based CRBN ligand starts with a S_N_Ar of pyridine **40** with Boc-protected piperazine **39** to afford **41**. Reduction of the nitro group was achieved via catalytic hydrogenation. Treatment of aniline **42** with acrylamide afforded **43**, albeit in low yields. Ring closure with CDI was unsuccessful, but 4-nitrophenylchloroformate did the job. Next, deprotection and linker extension via reductive amination afforded intermediates **45a** and **45b**. The synthesis of AURKA ligand **55** starts with a bromination of methylpyridine **46** with NBS, followed by substitution with the lithium enolate of BOC-protected methylpiperidine-4-carboxylate (**48**). Buchwald-Hartwig amination with *tert*-butyl protected pyrazole **52** gave **53** in 66% yield. Pyrazole **52** itself was obtained by reaction of *tert*-butyl hydrazine **50** and 3-aminocrotonitrile **51** in aqueous NaOH.^47^ BOC removal with TFA and subsequent alkylation delivered intermediate **54**. Because an attempted dual pyrazole deprotection and ester hydrolysis in aqueous HCl resulted in a complex reaction mixture, a 2-step procedure was followed. Treatment of **54** with NaOH, followed by *tert*-butyl cleavage with 4 M HCl in dioxane at 100 °C afforded AURKA ligand **55** in 82% yield over 2 steps. This ligand was subsequently turned into PROTACs **SK4454** and **SK5527** via classical HATU-coupling with dihydrouracil-based intermediates **45a** and **45b** after BOC removal.

**Scheme 1:**
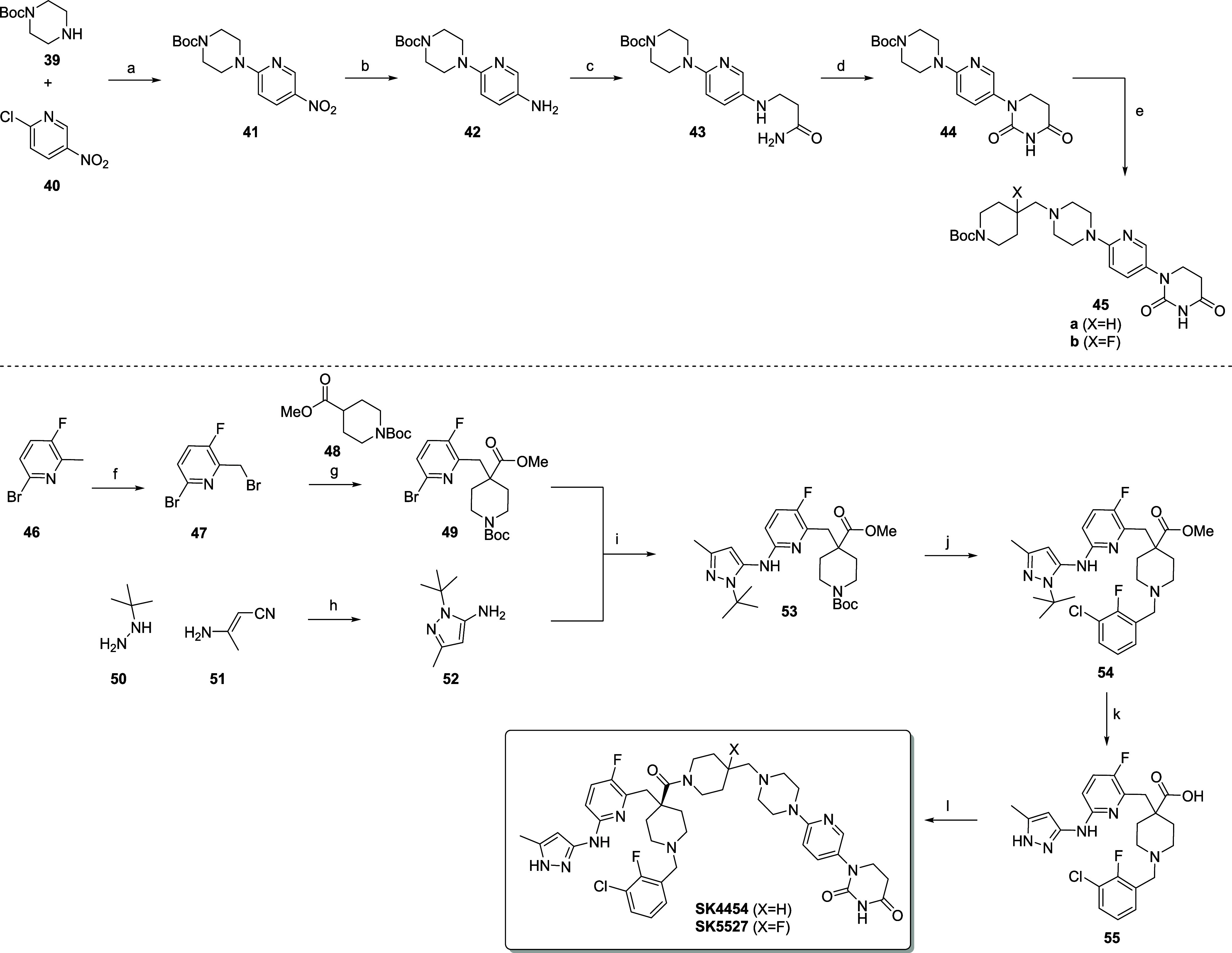
Synthesis of PROTACs SK4454 and SK5527 As an Example^a^

a Reagents and conditions: (a) DIPEA, MeCN, 80 °C, 98%; (b) H_2_, Pd/C 10%, 88%; (c) acrylamide, toluene, 100 °C, 30%; (d) 4-nitrophenyl chloroformate, K_2_CO_3_, DMF, 100 °C, 58%; (e) i. 4 M HCl in dioxane, 94%, ii. appropriate aldehyde, NaBH­(OAc)_3_, NaHCO_3_, DMF, 58% (a), 49% (b); (f) NBS, AIBN, CCl_4_, 89%; (g) LDA, THF, −78 °C, 78%; (h) NaOH, water, 90 °C, 73%; (i) Xantphos, Cs_2_CO_3_, Pd_2_(dba)_3_, toluene, 110 °C, 66%; (j) i. TFA, DCM, ii. 3-chloro-2-fluorobenzyl bromide, K_2_CO_3_, DMF, 87%; (k) i. 2 M NaOH, MeOH, 60 °C, ii. 4 M HCl in dioxane, 95 °C, 82%; (l) i. 45a or b, TFA, DCM, ii. 55, HATU, DIPEA, DMF, 43% (SK4454), 65% (SK5527).

## Experimental Section

### Compounds

All PROTACs, including inactive analogues, thalidomide and MK-5108 were synthesized in-house and are >95% pure by HPLC. Additional compounds used in this study were purchased commercially, including LY3295668 (MedChemExpress, #HY-114258), TAS-119 (MedChemExpress, #HY-137377), MG-132 (Sigma-Aldrich, #M7449) and tariquidar (Selleck Chemicals, #S8028).

### Cell Culture

The use of human cell lines in this study was approved by the Ethics Committee of Ghent University Hospital (EC/029-2022/sds). Human neuroblastoma cell lines NGP, IMR-32, SJNB-8, SK-N-BE(2)-C, SJNB-1, SK-N-SH and SK-N-AS, as well as benign immortalized cell lines RPE and HEK293T were either bought commercially or obtained through research laboratories. The neuroblastoma cell lines and HEK293T were cultured in RPMI 1640 medium (Gibco, #52400041) supplemented with 10% fetal bovine serum (Sigma-Aldrich, #F0804), 2 mM l-glutamine (Gibco, #11548876), and 100 IU/mL penicillin/streptomycin (Gibco, #11548876). RPE cells were cultured in DMEM-F12 (Gibco, #11594426), supplemented with 10% fetal bovine serum, 2 mM l-glutamine and 100 IU/mL penicillin/streptomycin. Screening for mycoplasma contamination occurred routinely on the cells. Human neuroblastoma cell lines SH-SY5Y_shABCB1 & SH-SY5Y_shNTC were generated by transduction with pLKO.1-ABCB1_TRCN0000059683 shRNA (Sigma MISSION SHCLND) or pLKO.1-puro NT shRNA (Sigma MISSION SHC002) vectors respectively (Supplementary Table S6), packaged using Lenti-X293T (Clontech). Transduced cells were selected with 2 μg/mL puromycin. These cells were cultured in DMEM 4.5 g/L d-Glucose, l-Glutamine and 110 mg/L Sodium Pyruvate (Gibco, #11995065) supplemented with 10% fetal bovine serum, 1× Antibiotic-Antimycotic solution (Sigma-Aldrich, #A5955) and 1× MEM nonessential amino acids (Sigma-Aldrich, #M7145). Incubation of all cells occurred at 37 °C and 5% CO_2_.

### IncuCyte Live-Cell Imaging

Neuroblastoma cells were seeded in 384-well plates at different densities ranging from 1200 to 5000 cells/well and allowed to adhere overnight. RPE and HEK293T were seeded at 200 and 600 cells/well, respectively. Compound treatment was performed using the D300e Digital Dispenser (Tecan) in duplicate or triplicate and cell confluency was monitored over time using the IncuCyte Live-cell imaging system (Sartorius). Percentage confluence data at 72 h was normalized to the DMSO negative control. Mean normalized confluences were calculated from 3 individual experiments.

### Alamar Blue Assay

SH-SY5Y_shABCB1 and SH-SY5Y_shNTC cells were seeded in 384-well plates at a density of 5500 cells/well and allowed to adhere overnight. Compound treatment was performed using the D300e Digital Dispenser (Tecan) in duplicate. At the 72 h time point, Alamar Blue reagent was added to each well. Fluorescence readouts were performed using the EnVision Multimode Plate Reader (PerkinElmer) immediately after reagent addition (baseline) and after 5 h incubation. Fluorescence intensities of the 5 h time point were subtracted by baseline levels and percent viability was calculated by normalizing to the negative DMSO control, applying a positive-control correction with Benzethonium Chloride-treated cells. Mean percent viability values were calculated from 3 individual experiments.

### Caspase-Glo 3/7 Assay

Neuroblastoma cells were seeded in 384-well plates at different densities ranging from 1200 to 5000 cells/well and allowed to adhere overnight. Compound treatment was performed using the D300e Digital Dispenser (Tecan) in duplicate. At the 48 h time point, a Caspase-Glo 3/7 Assay (Promega, #G8090) was performed following the manufacturer’s protocol, but adapted to add a 1:2 ratio of Caspase-Glo 3/7 reagent volume to sample volume. The readout was performed using the GloMax Discover Microplate Reader (Promega). Caspase 3/7 luminescence values were normalized to that of the DMSO negative control, and mean relative caspase 3/7 luminescence values were calculated from 3 individual experiments.

### Protein Extraction from Cells

Cells were seeded in T-25 flasks at different densities ranging from 1 to 2 million cells per flask and allowed to adhere for 1–2 days. In experiments involving compound treatment, this was done by medium replacement containing the compound(s) of interest in their corresponding concentrations. In competition binding experiments, cells were first pretreated with an excess of MK-5108, thalidomide or MG-132 for 1 h by shaking gently at room temperature prior to addition of SK4454 or SK5527. All samples were normalized to contain the same percentage of DMSO as the negative DMSO controls. Cells were harvested on ice at their respective time points by scraping, washed with ice-cold PBS to collect remaining cells, and centrifuged (2500 rpm, 4 °C). A second washing and centrifugation step was performed prior to collection of the final cell pellets which were then snap-frozen in liquid nitrogen and kept frozen at −80 °C until further processing. Lysis of cell pellets occurred in cold RIPA buffer (0.5% w/v Sodium Deoxycholate, 150 mM NaCl, 50 mM TrisHCl pH 7.5, 0.1% w/v SDS, 1% w/v NP-40) supplemented with protease and phosphatase inhibitors (Roche, #11836145001, #04906845001) by rotating gently at 4 °C for 1h, followed by a centrifugation step for 10 min (10,000 rpm, 4 °C). The supernatant was collected and concentrations of the obtained protein lysates were measured using the Pierce BCA Protein Assay Kit (Thermo Fisher Scientific, #23227).

### Simple Western

Simple Western analyses were performed with the Wes instrument (Bio-Techne) using the 12–230 kDa Wes Separation module and the EZ standard pack reagent 1 (Biotechne, #SM-W001, #PS-ST01EZ-8) following the manufacturer’s protocol. Protein lysates were loaded at 0.8 μg/μL under denaturing conditions (0.2 μg/μL for MYCN detection). Chemiluminescence peaks were generated by the Compass Software (Bio-Techne) and the area under the peaks calculated using the Gaussian fit method. Per lane, quantified data were always normalized to that of the loading control (Vinculin) prior to calculating fold changes relative to the negative DMSO control. Mean fold changes are calculated from 3 individual experiments. A list of the primary and secondary antibodies used can be found in the Supporting Information (Supplementary Table S7).

### Western Blot

Protein lysates were denatured through addition of Laemmli buffer supplemented with β-mercaptoethanol (Sigma-Aldrich, #M6250) heated at 95 °C for 10 min. Samples were subsequently loaded on 10% Mini-Protean TGX Precast Gels (Bio-Rad, #456-1034) with a total protein input mass of 35 μg and run at 120 V for 1 h in 10× Tris/glycine/SDS buffer (Bio-Rad, #161-0772). The ladder used was the PageRuler Prestained Protein Ladder (Thermo Fisher Scientific, #26617). Afterward, proteins were blotted on nitrocellulose membranes (Bio-Rad, #162-0233) by running at 100 V for 1 h with a buffer containing 10% 10× Tris/glycine (Bio-Rad, #161-0771) and 20% methanol (Thermo Fisher Scientific, #0390D). Ponceau staining was used for confirmation of protein transfer. Membranes were blocked in 5% milk/TBST or 5% BSA/TBST for 1 h at room temperature and subsequently incubated with primary antibodies overnight at 4 °C. Blots were washed 3× for 5 min each with TBST prior to incubation with secondary antibodies for 1 h at room temperature. Visualization of the blots was done using WestDura or WestFemto (Thermo Fisher Scientific, #34096, #34076) with the Amersham Imager 680 (BIOKÉ). Blots were stripped using the Restore Stripping Buffer (Thermo Fisher Scientific, #21059) prior to detection of additional proteins. Images were compiled and quantified using Sciugo (https://sciugo.com/). Per lane, quantified data were always normalized to that of the loading control (Vinculin or GAPDH), prior to calculating fold changes. Mean fold changes are calculated from 3 individual experiments. A list of the primary and secondary antibodies used can be found in the Supporting Information (Supplementary Table S7).

### AURKA/AURKB/CRBN Binding Assays

AURKA, AURKB and CRBN binding assays were performed by Eurofins Discovery using the KdELECT Kinase Assay Panel (AURKA and AURKB), or the E3scan Ligand Binding Assay Technology (CRBN). More information can be found on the Eurofins Discovery Web site.

### NanoBRET Assay

The NanoBRET Target Engagement Assay (Promega) was performed according to the recommended instructions from the manufacturer. Plasmids encoding full-length wild-type AURKA fused to Nano-Luc via AURKA’s C-terminus (Promega, #NV1041) were transfected into HEK293T cells using FuGENE HD (Promega, #E2312). Transfected cells were subsequently seeded in 96-well plates (Nunc, #136101) at a density of 20,000 cells/well in Opti-MEM medium without phenol red (Gibco, #11058-021) and incubated for 24h. Afterward, NanoBRET tracer K10 (Promega, #N2840) was added at a concentration of 7.8 nM for the assay in live-mode and 2.6 nM in permeabilized-mode. The plate was mixed at 900 rpm for 15 s, using an orbital shaker. For the permeabilized-mode, digitonin (Sigma-Aldrich, #D141) was additionally added at a final concentration of 50 μg/mL. Test compounds were subsequently administered in duplicate using the D300e digital dispenser (Tecan), followed by incubation for 2 h (37 °C, 5% CO2). To measure BRET, nanoglo substrate and extracellular nanoluc inhibitor (only in live-mode) (Promega #N2540) were added, and luminescence was measured using the GloMax Discover Microplate Reader (Promega) with a luminescence filter pair of 450 nm (BP filter, donor) and 600 nm (LP filter, acceptor). The NanoBRET ratio was calculated using the following formula: BRET ratio = 1000*­[(acceptor signal/donor signal)_with tracer_ – (acceptor signal/donor signal)_no tracer_]. Relative BRET ratios (%) were generated by normalizing to the negative DMSO control. The experiment was performed once with 2 replicate samples.

### KINOMEscan

The kinome-wide binding assay was performed by Eurofins Discovery using the scanMAX screening platform. More information can be found on the Eurofins Discovery Web site.

### LC–MS/MS Proteomics

IMR-32 cells were seeded in T-25 flasks at a density of 1.7 million cells/flask and treated the next day with DMSO (negative control), SK5527 (500 nM) or MK-5108 (500 nM) for 3 or 24 h (data for MK-5108 not discussed in this manuscript). Cell pellets were harvested at the respective time points, lysed, and the concentration of the obtained protein lysates measured as previously described. Details on further sample preparation, LC–MS/MS, and data analysis can be found in the Supporting Information. The LC–MS/MS proteomics data have been deposited to the ProteomeXchange Consortium via the PRIDE^48^ partner repository with the data set identifier PXD061539.

### In Vitro Physicochemical and ADME Determination for SK4454 and SK5527

In vitro physicochemical and ADME properties of PROTACs SK4454 and SK5527, including aqueous solubility, lipophilicity (LogD), plasma protein binding and permeability (in Caco-2 cells) were performed by Eurofins Villapharma. Detailed protocols can be found in the Supporting Information.

### Plasma and Metabolic Stability Assays

Plasma and metabolic stability tests were performed by Eurofins Discovery. For the plasma stability assay, test compounds were incubated in CD-1 mouse plasma for 0, 0.5, 1, 1.5, and 2 h at 37 °C at a concentration of 1 μM. For the metabolic stability assay, test compounds were incubated at 37 °C in either (1) CD-1 mouse liver microsomes (MLM) (0.1 mg/mL) at a concentration of 0.1 μM for 0, 15, 30, 45, and 60 min or (2) CD-1 mouse hepatocytes (MH) (0.7 million viable cells/mL) at a concentration of 1 μM for 0, 0.5, 1, 1.5, and 2 h. At the respective time points, acetonitrile was added to the incubation mixture followed by centrifugation. Samples were analyzed by HPLC-MS/MS and peak areas were recorded for each analyte. Compound stability, expressed as the percentage of parent compound remaining, was calculated by comparing the peak area of the compound at each time point relative to that of the 0h time point. Percentage compound remaining is plotted as a function of time as a logarithmic curve. Half-life (*t*_1/2_) is estimated from the slope of the initial linear range of the curve, assuming first-order kinetics. The apparent intrinsic clearance (CL_int_) in MLM (μL/min/mg) or MH (μL/min/10^6^ cells) was calculated according to the following formula: CL_int_ = 0.693/(*t*_1/2_*­(MLM protein concentration in mg/μL OR MH cell density in cells/μL)). Both plasma and metabolic assay experiments were performed once with 2 replicate samples each.

### General Statement for Animal Use

Both the in vivo pharmacokinetics and in vivo pharmacodynamics studies were performed by Eurofins Pharmacology Discovery Services. All mice were provided by BioLasco Taiwan (under Charles River Laboratories Licensee). Animals were acclimated for 3 days prior to use and were confirmed to be in good health. All aspects of this work including housing, experimentation, and animal disposal were performed in general accordance with the “Guide for the Care and Use of Laboratory Animals: Eighth Edition” (National Academies Press, Washington, DC. 2011) in a AAALAC-accredited laboratory animal facility. In addition, the animal care and use protocol was reviewed and approved by the IACUC at Eurofins Pharmacology Discovery Services; IN020-08202020 and ON002-07142023.

### In Vivo Pharmacokinetics Study

Male ICR (CD-1) mice, weighing 30 ± 5 g, were given intravenous (IV), intraperitoneal (IP) or oral gavage (PO) administrations of PROTACs at 15 mg/kg (IV and IP) or 30 mg/kg (PO). Dosing volumes used were 5 mL/kg (IV and IP) and 10 mL/kg (PO). The test compounds were dissolved in 5% DMSO/5% Solutol HS15/90% PBS. Serial blood samples were collected at 0.05, 0.167, 0.5, 1, 2, 4, 8, 12 h postadministration (an additional 24h sample was collected for IV-administered mice). Blood samples were collected from three animals at each time point via facial vein (±50 μL) for the first time points and cardiac puncture (±300 μL) for the final time point and stored in tubes coated with lithium heparin. Within 1h of collection, samples were mixed gently and centrifuged at 2500 g for 15 min at 4 °C. Plasma samples were then harvested and kept frozen at −70 °C until further processing. Samples were processed using acetonitrile precipitation and analyzed by LC–MS/MS. Mean plasma concentrations were plotted as a function of time and the fundamental PK parameters were obtained from a noncompartmental analysis (NCA) using WinNonlin. Free drug levels (*C*_p,u_) for SK4454 and SK5527 were calculated by factoring in their plasma protein binding (*f*_u_) values using the following formula: *C*_p,u_ = *C*_p_ * *f*_u_.

### In Vivo Pharmacodynamics Study

Six-week old female BALB/c nude mice were subcutaneously implanted with viable IMR-32 cells formulated in 50% Matrigel into the right flank (1 × 10^7^ cells/mouse, in 0.2 mL volume). Palpable tumor volumes were measured with a caliper and calculated according to the prolate ellipsoid formula: tumor volume = length × width^2^ × 0.5. When the mean tumor volume reached ∼250 mm^3^, animals were randomized into groups (0, 4, 8, 24 h) with 3 animals per group. The 0 h group, which serves as a baseline, did not receive treatment and tumors were harvested postrandomization. Other groups were given a single IV or IP administration of SK5527 or SK4454 at 15 mg/kg (dissolved in 5% DMSO/5% Solutol HS15/90% PBS). Tumors were subsequently harvested at the respective time points, snap-frozen in liquid nitrogen, and kept frozen at −70 °C until further processing. Upon receipt of the samples in our lab, we mechanically digested and lysed tumor pieces by adding cold RIPA lysis buffer (with protease and phosphatase inhibitors as previously described) and Stainless Steel Beads (Qiagen, #69989) followed by shaking for 2 min (twice) using TissueLyser II (Qiagen). Afterward, samples were centrifuged for 20 min (10,000 rpm, 4 °C). The supernatant was then collected and the concentration of the obtained protein lysates was measured using the Pierce BCA Protein Assay Kit (Thermo Fisher Scientific, #23227). Western blot was performed as previously described to visualize changes in AURKA protein levels.

### Data Analysis and Statistics

Graphs were generated using GraphPad Prism Software (version 10.1.2) or RStudio (R-4.3.2). All dose–response curves were computed on GraphPad Prism using the Variable Slope (four parameters) equation. Absolute half-maximal degradation (DC_50_), growth inhibitory (GI_50_) and enzymatic inhibitory (IC_50_) concentrations, as well as inhibition constants (*K*i) were derived from dose–response curves via interpolation. Statistical tests were performed on GraphPad Prism and detailed in the figure legends. Unless otherwise stated, “n” in the figure legends indicates the number of independent experiments conducted at different times.

## Supplementary Material







## Notes

Additional in vitro biological data, biochemical assays, and in vivo PK data; supplementary methods including LC–MS/MS proteomics, in vitro physicochemical and ADME determination; detailed experimental procedures of chemical reactions, characterization data, and spectra (HRMS, NMR, HPLC) of intermediates and final compounds (PDF)
Full KINOMEscan and LC–MS/MS data (XLSX)
Molecular-formula strings (SMILES) for all synthesized compounds (CSV)

## Abbreviations

ABCB1: ATP-binding cassette subfamily B member 1 (P-glycoprotein)
AURKA: Aurora kinase A
AURKB: Aurora kinase B
CDK12: Cyclin-dependent kinase 12
CDX: Cell line-derived xenograft
CRBN: Cereblon
ESCO2: Establishment of sister chromatid cohesion N-acetyltransferase 2
HAND2: Heart- and neural crest derivative-expressed protein 2
IP: Intraperitoneal
IV: Intravenous
MDR1: Multidrug resistance protein 1
MH: Mouse hepatocytes
MLM: Mouse liver microsomes
MYCN: V-myc avian myelocytomatosis viral oncogene neuroblastoma derived homologue
PEG: Polyethylene glycol
PK: Pharmacokinetics
PLK1: Polo-like kinase 1
PO: Per Os (oral administration)
PROTACs: Proteolysis targeting chimeras
shNTC: Nontargeting control shRNA
shRNA: short hairpin RNA
TPX2: Targeting protein for *Xenopus* Kinesin-like protein 2
TrKA: Tropomyosin receptor kinase A.

## References

[1] Matthay K. K., Maris J. M., Schleiermacher G., Nakagawara A., Mackall C. L., Diller L., Weiss W. A. (2016). Neuroblastoma. Nat. Rev. Dis. Primers.

[2] Brodeur G. M., Seeger R. C., Schwab M., Varmus H. E., Bishop J. M. (1984). Amplification
of N-myc in Untreated Human Neuroblastomas Correlates with Advanced
Disease Stage. Science (1979).

[3] Seeger R. C., Brodeur G. M., Sather H., Dalton A., Siegel S. E., Wong K. Y., Hammond D. (1985). Association of multiple
copies of
the N-myc oncogene with rapid progression of neuroblastomas. N Engl J. Med..

[4] Maris J. M., Hogarty M. D., Bagatell R., Cohn S. L. (2007). Neuroblastoma. Lancet.

[5] Wolpaw A. J., Bayliss R., Büchel G., Dang C. V., Eilers M., Gustafson W. Clay, Hansen G. H., Jura N., Knapp S., Lemmon M. A., Levens D., Maris J. M., Marmorstein R., Metallo S. J., Park J. R., Penn L. Z., Rape M., Roussel M. F., Shokat K. M., Tansey W. P., Verba K. A., Vos S. M., Weiss W. A., Wolf E., Mossé Y. P. (2021). Drugging
the “Undruggable” MYCN Oncogenic Transcription Factor:
Overcoming Previous Obstacles to Impact Childhood Cancers. Cancer Res..

[6] Shimomura T., Hasako S., Nakatsuru Y., Mita T., Ichikawa K., Kodera T., Sakai T., Nambu T., Miyamoto M., Takahashi I., Miki S., Kawanishi N., Ohkubo M., Kotani H., Iwasawa Y. (2010). MK-5108, a highly selective
Aurora-A kinase inhibitor, shows antitumor activity alone and in combination
with docetaxel. Mol. Cancer Ther.

[7] Du J., Yan L., Torres R., Gong X., Bian H., Marugan C., Boehnke K., Baquero C., Hui Y. H., Chapman S. C., Yang Y., Zeng Y., Bogner S. M., Foreman R. T., Capen A., Donoho G. P., Van Horn R. D., Barnard D. S., Dempsey J. A., Beckmann R. P., Marshall M. S., Chio L. C., Qian Y., Webster Y. W., Aggarwal A., Chu S., Bhattachar S., Stancato L. F., Dowless M. S., Iversen P. W., Manro J. R., Walgren J. L., Halstead B. W., Dieter M. Z., Martinez R., Bhagwat S. V., Kreklau E. L., Lallena M. J., Ye X. S., Patel B. K. R., Reinhard C., Plowman G. D., Barda D. A., Henry J. R., Buchanan S. G., Campbell R. M. (2019). Aurora
A-Selective Inhibitor LY3295668 Leads to Dominant Mitotic Arrest,
Apoptosis in Cancer Cells, and Shows Potent Preclinical Antitumor
Efficacy. Mol. Cancer Ther.

[8] Miura A., Sootome H., Fujita N., Suzuki T., Fukushima H., Mizuarai S., Masuko N., Ito K., Hashimoto A., Uto Y., Sugimoto T., Takahashi H., Mitsuya M., Hirai H. (2021). TAS-119, a
novel selective Aurora A and TRK inhibitor, exhibits antitumor efficacy
in preclinical models with deregulated activation of the Myc, β-Catenin,
and TRK pathways. Invest New Drugs.

[9] Otto T., Horn S., Brockmann M., Eilers U., Schüttrumpf L., Popov N., Kenney A. M., Schulte J. H., Beijersbergen R., Christiansen H., Berwanger B., Eilers M. (2009). Stabilization of N-Myc
is a critical function of Aurora A in human neuroblastoma. Cancer Cell.

[10] Richards M. W., Burgess S. G., Poon E., Carstensen A., Eilers M., Chesler L., Bayliss R. (2016). Structural basis of
N-Myc binding by Aurora-A and its destabilization by kinase inhibitors. Proc. Natl. Acad. Sci. U. S. A..

[11] Büchel G., Carstensen A., Mak K. Y., Roeschert I., Leen E., Sumara O., Hofstetter J., Herold S., Kalb J., Baluapuri A., Poon E., Kwok C., Chesler L., Maric H. M., Rickman D. S., Wolf E., Bayliss R., Walz S., Eilers M. (2017). Association with Aurora-A Controls N-MYC-Dependent
Promoter Escape and Pause Release of RNA Polymerase II during the
Cell Cycle. Cell Rep.

[12] Müller, M. ; Eing, L. ; Uhl, L. ; Schmitz, M. ; Kaneva, I. ; Byrne, D. P. ; Fleischhauer, D. ; Burgess, S. G. ; Fischer, S. ; Gebauer, N. ; Nancy-Portebois, V. ; Kaltheuner, I. H. ; Ha, S. A. ; Schülein-Völk, C. ; Solvie, D. ; Richards, M. W. ; Vidal, R. ; Brand, M. ; Papadopoulos, D. ; Bayliss, R. ; Eyers, P. A. ; Eyers, C. E. ; Geyer, M. ; Eilers, M. ; Büchel, G. , The MYCN/Aurora-A complex is a cyclin activating kinase for CDK12, BioRxiv (2025) 2025.01.15.633177. 10.1101/2025.01.15.633177.

[13] Byrum A. K., Carvajal-Maldonado D., Mudge M. C., Valle-Garcia D., Majid M. C., Patel R., Sowa M. E., Gygi S. P., Harper J. Wade, Shi Y., Vindigni A., Mosammaparast N. (2019). Mitotic regulators
TPX2 and Aurora A protect DNA forks during replication stress by counteracting
53BP1 function. J. Cell Biol..

[14] Adhikari B., Bozilovic J., Diebold M., Schwarz J. D., Hofstetter J., Schröder M., Wanior M., Narain A., Vogt M., Dudvarski Stankovic N., Baluapuri A., Schönemann L., Eing L., Bhandare P., Kuster B., Schlosser A., Heinzlmeir S., Sotriffer C., Knapp S., Wolf E. (2020). PROTAC-mediated
degradation reveals a non-catalytic function of AURORA-A kinase. Nat. Chem. Biol..

[15] Wang R., Ascanelli C., Abdelbaki A., Fung A., Rasmusson T., Michaelides I., Roberts K., Lindon C. (2021). Selective targeting
of non-centrosomal AURKA functions through use of a targeted protein
degradation tool. Commun. Biol..

[16] Liu F., Wang X., Duan J., Hou Z., Wu Z., Liu L., Lei H., Huang D., Ren Y., Wang Y., Li X., Zhuo J., Zhang Z., He B., Yan M., Yuan H., Zhang L., Yan J., Wen S., Wang Z., Liu Q. (2022). A Temporal PROTAC Cocktail-Mediated
Sequential Degradation of AURKA Abrogates Acute Myeloid Leukemia Stem
Cells. Adv. Sci. (Weinh).

[17] Bozilovic J., Eing L., Berger B.-T., Adhikari B., Weckesser J., Berner N. B., Wilhelm S., Kuster B., Wolf E., Knapp S. (2022). Novel, highly potent
PROTACs targeting AURORA-A kinase. Current Research
in Chemical Biology.

[18] Rishfi M., Krols S., Martens F., Bekaert S. L., Sanders E., Eggermont A., De Vloed F., Goulding J. R., Risseeuw M., Molenaar J., De Wilde B., Van Calenbergh S., Durinck K. (2023). Targeted AURKA degradation: Towards new therapeutic
agents for neuroblastoma. Eur. J. Med. Chem..

[19] Tang J., Moorthy R., Hirsch L. E., Demir Ö., Baker Z. D., Naumann J. A., Jones K. F. M., Grillo M. J., Haefner E. S., Shi K., Levy M. J., Gupta H. B., Aihara H., Harris R. S., Amaro R. E., Levinson N. M., Harki D. A. (2025). Targeting N-Myc
in neuroblastoma with selective Aurora kinase A degraders. Cell Chem. Biol..

[20] Sflakidou E., Adhikari B., Siokatas C., Wolf E., Sarli V. (2024). Development
of 2-Aminoadenine-Based Proteolysis-Targeting Chimeras (PROTACs) as
Novel Potent Degraders of Monopolar Spindle 1 and Aurora Kinases. ACS Pharmacol. Transl. Sci..

[21] Nelson S. E., Tucker J. R., Prado M. G., Tierney L. C., Quigley S. L., Lumpkin A. T., Dodd C. J., Hasko V., Howie S. L., Aastha, Ody B. K., Yin J., Heemstra J. M., Turlington M. (2025). Development of Dual Aurora-A and
Aurora-B Degrading PROTACs for MYCN-Amplified Neuroblastoma. ChemMedChem..

[22] Donovan K. A., Ferguson F. M., Bushman J. W., Eleuteri N. A., Bhunia D., Ryu S. S., Tan L., Shi K., Yue H., Liu X., Dobrovolsky D., Jiang B., Wang J., Hao M., You I., Teng M., Liang Y., Hatcher J., Li Z., Manz T. D., Groendyke B., Hu W., Nam Y., Sengupta S., Cho H., Shin I., Agius M. P., Ghobrial I. M., Ma M. W., Che J., Buhrlage S. J., Sim T., Gray N. S., Fischer E. S. (2020). Mapping the Degradable Kinome Provides
a Resource for Expedited Degrader Development. Cell.

[23] Sakamoto K. M., Kim K. B., Kumagai A., Mercurio F., Crews C. M., Deshaies R. J. (2001). Protacs: chimeric molecules that target proteins to
the Skp1-Cullin-F box complex for ubiquitination and degradation. Proc. Natl. Acad. Sci. U. S. A..

[24] Goracci L., Desantis J., Valeri A., Castellani B., Eleuteri M., Cruciani G. (2020). Understanding the Metabolism
of Proteolysis
Targeting Chimeras (PROTACs): The Next Step toward Pharmaceutical
Applications. J. Med. Chem..

[25] Sievers Q. L., Petzold G., Bunker R. D., Renneville A., Słabicki M., Liddicoat B. J., Abdulrahman W., Mikkelsen T., Ebert B. L., Thomä N. H. (2018). Defining
the human C2H2 zinc finger degrome targeted by thalidomide analogs
through CRBN. Science.

[26] Nguyen T. M., Sreekanth V., Deb A., Kokkonda P., Tiwari P. K., Donovan K. A., Shoba V., Chaudhary S. K., Mercer J. A. M., Lai S., Sadagopan A., Jan M., Fischer E. S., Liu D. R., Ebert B. L., Choudhary A. (2024). Proteolysis-targeting
chimeras with reduced off-targets. Nat. Chem..

[27] Norris S., Ba X., Rhodes J., Huang D., Khambatta G., Buenviaje J., Nayak S., Meiring J., Reiss S., Xu S., Shi L., Whitefield B., Alexander M., Horn E. J., Correa M., Tehrani L., Hansen J. D., Papa P., Mortensen D. S. (2023). Design
and Synthesis of Novel Cereblon
Binders for Use in Targeted Protein Degradation. J. Med. Chem..

[28] Murgai A., Sosič I., Gobec M., Lemnitzer P., Proj M., Wittenburg S., Voget R., Gütschow M., Krönke J., Steinebach C. (2022). Targeting the deubiquitinase USP7
for degradation with PROTACs. Chem. Commun.
(Camb).

[29] Rathje O.
H., Perryman L., Payne R. J., Hamprecht D. W. (2023). PROTACs
Targeting MLKL Protect Cells from Necroptosis. J. Med. Chem..

[30] Tsang T., Huerta F., Liu Y., Che J., Metivier R. J., Ferrao S., Donovan K. A., Jones L. H., Zerfas B. L., Nowak R. P. (2023). HiBiT-SpyTag: A Minimal Tag for Covalent
Protein Capture
and Degrader Development. ACS Chem. Biol..

[31] Yang Y., Ding L., Zhou Q., Fen L., Cao Y., Sun J., Zhou X., Liu A. (2020). Silencing
of AURKA augments the antitumor
efficacy of the AURKA inhibitor MLN8237 on neuroblastoma cells. Cancer Cell Int..

[32] Hornberger K. R., Araujo E. M. V. (2023). Physicochemical Property Determinants of Oral Absorption
for PROTAC Protein Degraders. J. Med. Chem..

[33] Fabian M. A., Biggs W. H., Treiber D. K., Atteridge C. E., Azimioara M. D., Benedetti M. G., Carter T. A., Ciceri P., Edeen P. T., Floyd M., Ford J. M., Galvin M., Gerlach J. L., Grotzfeld R. M., Herrgard S., Insko D. E., Insko M. A., Lai A. G., Lélias J. M., Mehta S. A., Milanov Z. V., Velasco A. M., Wodicka L. M., Patel H. K., Zarrinkar P. P., Lockhart D. J. (2005). A small molecule-kinase
interaction map for clinical kinase inhibitors. Nat. Biotechnol..

[34] Vasta J. D., Corona C. R., Wilkinson J., Zimprich C. A., Hartnett J. R., Ingold M. R., Zimmerman K., Machleidt T., Kirkland T. A., Huwiler K. G., Ohana R. F., Slater M., Otto P., Cong M., Wells C. I., Berger B. T., Hanke T., Glas C., Ding K., Drewry D. H., Huber K. V. M., Willson T. M., Knapp S., Müller S., Meisenheimer P. L., Fan F., Wood K. V., Robers M. B. (2018). Quantitative,
Wide-Spectrum Kinase Profiling in Live Cells for Assessing the Effect
of Cellular ATP on Target Engagement. Cell Chem.
Biol..

[35] Brockmann M., Poon E., Berry T., Carstensen A., Deubzer H. E., Rycak L., Jamin Y., Thway K., Robinson S. P., Roels F., Witt O., Fischer M., Chesler L., Eilers M. (2013). Small Molecule Inhibitors of Aurora-A
Induce Proteasomal Degradation of N-Myc in Childhood Neuroblastoma. Cancer Cell.

[36] Gustafson W. C., Meyerowitz J. G., Nekritz E. A., Chen J., Benes C., Charron E., Simonds E. F., Seeger R., Matthay K. K., Hertz N. T., Eilers M., Shokat K. M., Weiss W. A. (2014). Drugging
MYCN through an Allosteric Transition in Aurora Kinase A. Cancer Cell.

[37] Macůrek L., Lindqvist A., Lim D., Lampson M. A., Klompmaker R., Freire R., Clouin C., Taylor S. S., Yaffe M. B., Medema R. H. (2008). Polo-like kinase-1
is activated by aurora A to promote
checkpoint recovery. Nature.

[38] Xu M., Sun M., Zhang X., Nguyen R., Lei H., Shern J. F., Thiele C. J., Liu Z. (2023). HAND2 Assists MYCN Enhancer Invasion
to Regulate a Noradrenergic Neuroblastoma Phenotype. Cancer Res..

[39] Fu J., Zhou S., Xu H., Liao L., Shen H., Du P., Zheng X. (2023). ATM-ESCO2-SMC3 axis promotes 53BP1 recruitment in response
to DNA damage and safeguards genome integrity by stabilizing cohesin
complex. Nucleic Acids Res..

[40] Hartung I. V., Rudolph J., Mader M. M., Mulder M. P. C., Workman P. (2023). Expanding
Chemical Probe Space: Quality Criteria for Covalent and Degrader Probes. J. Med. Chem..

[41] Blagg J., Workman P. (2017). Choose and Use Your Chemical Probe
Wisely to Explore
Cancer Biology. Cancer Cell.

[42] Kurimchak A. M., Herrera-Montávez C., Montserrat-Sangrà S., Araiza-Olivera D., Hu J., Neumann-Domer R., Kuruvilla M., Bellacosa A., Testa J. R., Jin J., Duncan J. S. (2022). The drug
efflux pump MDR1 promotes intrinsic and acquired
resistance to PROTACs in cancer cells. Sci.
Signal.

[43] Wolf G., Craigon C., Teoh S. T., Essletzbichler P., Onstein S., Cassidy D., Uijttewaal E. C. H., Dvorak V., Cao Y., Bensimon A., Elling U., Ciulli A., Superti-Furga G. (2025). The efflux pump ABCC1/MRP1 constitutively
restricts PROTAC sensitivity in cancer cells. Cell Chem. Biol..

[44] Durbin A. D., Wang T., Wimalasena V. K., Zimmerman M. W., Li D., Dharia N. V., Mariani L., Shendy N. A. M., Nance S., Patel A. G., Shao Y., Mundada M., Maxham L., Park P. M. C., Sigua L. H., Morita K., Conway A. S., Robichaud A. L., Perez-Atayde A. R., Bikowitz M. J., Quinn T. R., Wiest O., Easton J., Schönbrunn E., Bulyk M. L., Abraham B. J., Stegmaier K., Look A. T., Qi J. (2022). EP300 Selectively Controls
the Enhancer
Landscape of MYCN-Amplified Neuroblastoma. Cancer
Discov.

[45] Dietrich C. G., Geier A., Oude Elferink R. P. J. (2003). ABC of oral bioavailability: transporters
as gatekeepers in the gut. Gut.

[46] Webborn P. J. H., Beaumont K., Martin I. J., Smith D. A. (2025). Free Drug Concepts:
A Lingering Problem in Drug Discovery. J. Med.
Chem..

[47] Mitchell D., Cole K. P., Pollock P. M., Coppert D. M., Burkholder T. P., Clayton J. R. (2012). Development and a Practical Synthesis of the JAK2 Inhibitor
LY2784544. Org. Process Res. Dev.

[48] Perez-Riverol Y., Bandla C., Kundu D. J., Kamatchinathan S., Bai J., Hewapathirana S., John N. S., Prakash A., Walzer M., Wang S., Vizcaíno J. A. (2025). The PRIDE
database at 20 years: 2025 update. Nucleic Acids
Res..

