# Supplementary material for: Second-Generation AURKA-Targeting PROTACs: Structural Optimization toward in Vivo Degradation in Neuroblastoma
Source: J Med Chem. 2025 Nov 18;68(22):23962–76. doi: 10.1021/acs.jmedchem.5c01271 (PMC12670405; doi:10.1021/acs.jmedchem.5c01271)
Supplement: Supplementary file 1 [file jm5c01271_si_001.pdf]

Supporting Information:

Second-generation AURKA-targeting PROTACs: structural optimization towards *in vivo* degradation in neuroblastoma

Simon Krols[1,3,#], Muhammad Rishfi[2,3,#], Fien Martens[2,3], Anouk Van Hauwermeiren[1], Ellen Sanders[2,3], Pieter-Jan De Sutter[4], An Vermeulen[4], Kaat De Wever[2,3], Sarah-Lee Bekaert[2,3], M. Emmy M. Dolman[5,6], Gabor Tax[5,6], Alvin Kamili[5,6], Jamie I. Fletcher[5,6], Lisa Depestel[2,3], Bram De Wilde[2,3,7], Kaat Durinck[2,3,*], Serge Van Calenbergh[1,3,*]

[1] Laboratory of Medicinal Chemistry, Department of Pharmaceutics, Faculty of Pharmaceutical Sciences, Ghent University, Ottergemsesteenweg 460, 9000 Ghent, Belgium

[2] Pediatric Precision Oncology Lab, Department of Biomolecular Medicine, Faculty of Medicine & Health Sciences, Ghent University, Corneel Heymanslaan 10, 9000 Ghent, Belgium

[3] Cancer Research Institute Ghent, Corneel Heymanslaan 10, 9000 Ghent, Belgium

[4] Laboratory of Medical Biochemistry and Clinical Analysis, Department of Bioanalysis, Ghent University, Ottergemsesteenweg 460, 9000 Ghent, Belgium

[5] Children's Cancer Institute, Lowy Cancer Research Centre, UNSW Sydney, Kensington, NSW, 2052 Sydney, Australia

[6] School of Clinical Medicine, UNSW Medicine & Health, UNSW Sydney, Kensington, NSW, 2052 Sydney, Australia

[7] Department of Internal Medicine and Pediatrics, Faculty of Medicine & Health Sciences, Ghent University, Corneel Heymanslaan 10, 9000 Ghent, Belgium

# These authors contributed equally as first authors

* These authors contributed equally as last authors

Corresponding authors: Serge.VanCalenbergh@UGent.be, Kaat.Durinck@UGent.be

TABLE OF CONTENTS

Supplementary Tables S2

Supplementary Figures S7

Supplementary Methods 1 – LC-MS/MS Proteomics S16

Supplementary Methods 2 – *In vitro* physicochemical and ADME properties S18

Supplementary Methods – Chemistry S20

SYNTHESIS OF SERIES 1 PROTACS S23

SYNTHESIS OF SERIES 2 PROTACS S40

SYNTHESIS OF SERIES 3 PROTACS S55

SYNTHESIS OF SERIES 4 PROTACS S70

References S109

Supplementary Tables

Supplementary Table S1. Summary of the structure/activity relationship of series 1 PROTACs. Linker rigidification of previously developed SK2188 and SK3277 [1].

SERIES 1

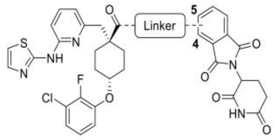

PROTAC Name	Linker	% Degradation 0.1 / 1 μM	DC_{50} (nM)	GI_{50} (nM)	pDC_{50} ± SEM	pGI_{50} ± SEM	Plasma $t_{1/2}$ (min)	CL_{int} MLM (μL/min/mg)	CL_{int} MH (μL/min/10^6cells)
SK2188[a]	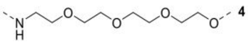	86 / 89	4	40	8.41 ± 0.05	7.40 ± 0.08	67	1390	
SK3277[a]	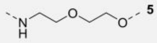	81 / 75	7	52	8.15 ± 0.16	7.28 ± 0.09	71	1085	
PROTAC 1	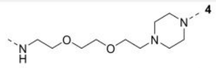	72 / 66		63		7.20 ± 0.09	> 120	1479	
PROTAC 2	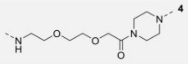	70 / 81		72		7.14 ± 0.09			
PROTAC 3	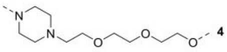	81 / 79	9	42	8.06 ± 0.11	7.38 ± 0.11	79	456	
PROTAC 4	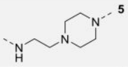	85 / 93	5	44	8.32 ± 0.06	7.35 ± 0.11	> 120	656	
PROTAC 5	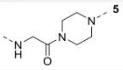	76 / 75		51		7.29 ± 0.11			
PROTAC 6	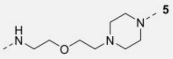	76 / 82		64		7.20 ± 0.12			
PROTAC 7	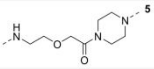	68 / 76		112		6.95 ± 0.08			
PROTAC 8	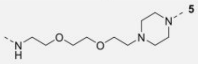	76 / 75		56		7.25 ± 0.12			
PROTAC 9	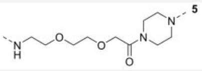	64 / 83		112		6.95 ± 0.05			
PROTAC 10	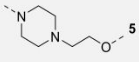	72 / 80		52		7.28 ± 0.11			
PROTAC 11	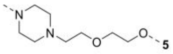	78 / 81	5	38	8.31 ± 0.13	7.42 ± 0.09			

blank = not determined
[a] *previously published*

Supplementary Table S2. Summary of the structure/activity relationship of series 2 PROTACs. Exploring arylaminoglutarimide-based ligands of cereblon.

SERIES 2

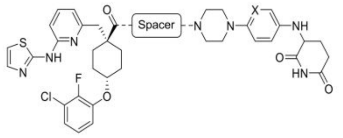

PROTAC Name	X	Spacer	% Degradation 0.1 / 1 µM	DC_{50} (nM)	GI_{50} (nM)	pDC_{50} ± SEM	pGI_{50} ± SEM	Plasma $t_{1/2}$ (min)	CL_{int} MLM (µL/min/mg)	CL_{int} MH (µL/min/10^6cells)
PROTAC 12	CH	none (direct connection)	12 / < 0		715		6.15 ± 0.12			
PROTAC 13	CH	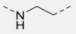	62 / 84	12	30	7.90 ± 0.13	7.53 ± 0.06			
PROTAC 14	CH	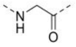	64 / 76	44	46	7.36 ± 0.14	7.34 ± 0.12	> 120	739	
PROTAC 15	CH	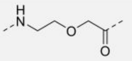	38 / 55		93		7.03 ± 0.07			
PROTAC 16	CH	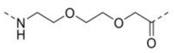	39 / 75		78		7.11 ± 0.10			
PROTAC 17	CH	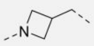	66 / 87	51	39	7.29 ± 0.09	7.41 ± 0.09			
PROTAC 18	CF	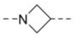	91 / 92	6	17	8.20 ± 0.10	7.77 ± 0.08	> 120	369	< 4.1
PROTAC 19	CF	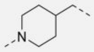	83 / 94		53		7.28 ± 0.18			
PROTAC 20	N	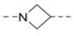	90 / 90	2	19	8.76 ± 0.08	7.72 ± 0.08	> 120	738	< 4.1

blank = not determined

Supplementary Table S3. Summary of the structure/activity relationship of series 3 PROTACs. Exploring dihydrouracil-based ligands of cereblon.

SERIES 3

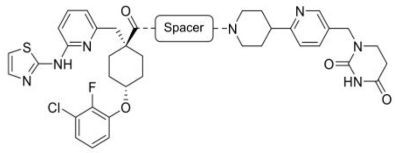

PROTAC Name	Spacer	% Degradation 0.1 / 1 μM	DC_{50} (nM)	GI_{50} (nM)	pDC_{50} ± SEM	pGI_{50} ± SEM	Plasma $t_{1/2}$ (min)	CL_{int} MLM (μL/min/mg)	CL_{int} MH (μL/min/10^6cells)
PROTAC 21	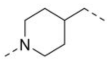	78 / 85	11	46	7.97 ± 0.10	7.34 ± 0.14	> 120	186	< 4.1
PROTAC 22	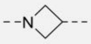	86 / 87		46		7.33 ± 0.10	> 120	693	< 4.1

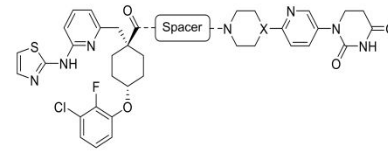

PROTAC Name	X	Spacer	% Degradation 0.1 / 1 μM	DC_{50} (nM)	GI_{50} (nM)	pDC_{50} ± SEM	pGI_{50} ± SEM	Plasma $t_{1/2}$ (min)	CL_{int} MLM (μL/min/mg)	CL_{int} MH (μL/min/10^6cells)
PROTAC 23	N	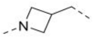	90 / 90	4	24	8.40 ± 0.12	7.63 ± 0.06	> 120	666	< 4.1
PROTAC 24	N	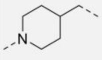	87 / 94	6	28	8.23 ± 0.07	7.56 ± 0.13	> 120	565	< 4.1
PROTAC 25	N	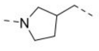	91 / 91	3	33	8.47 ± 0.06	7.49 ± 0.08	> 120	426	13.6
PROTAC 26	N	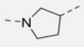	78 / 75	5	45	8.30 ± 0.15	7.35 ± 0.10			
PROTAC 27	N	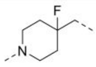	88 / 93		46		7.33 ± 0.12	> 120	1061	
PROTAC 28	CH	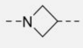	86 / 85	3	48	8.52 ± 0.10	7.32 ± 0.11	> 120	367	13

blank = not determined

Supplementary Table S4. Summary of the structure/activity relationship of series 4 PROTACs. Exploring the use of a novel AURKA ligand analogous to LY3295668.

SERIES 4

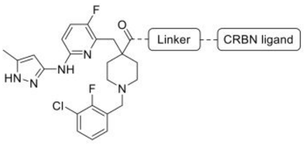

PROTAC Name	Linker - CRBN ligand	% Degradation 0.1 / 1 µM	DC_{50} (nM)	GI_{50} (nM)	pDC_{50} ± SEM	pGI_{50} ± SEM	Plasma $t_{1/2}$ (min)	CL_{int} MLM (µL/min/mg)	CL_{int} MH (µL/min/10^6cells)
PROTAC 29	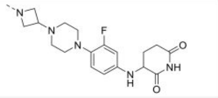	79 / 87	8	36	8.09 ± 0.06	7.44 ± 0.10	> 120	438	< 4.1
PROTAC 30	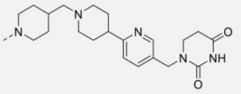	42 / 59		158		6.80 ± 0.08			

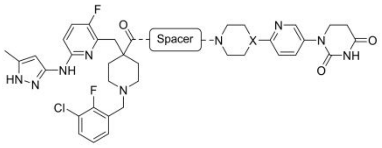

PROTAC Name	X	Spacer	% Degradation 0.1 / 1 µM	DC_{50} (nM)	GI_{50} (nM)	pDC_{50} ± SEM	pGI_{50} ± SEM	Plasma $t_{1/2}$ (min)	CL_{int} MLM (µL/min/mg)	CL_{int} MH (µL/min/10^6cells)
PROTAC 31	N	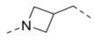	80 / 89		98		7.01 ± 0.07			
PROTAC 32 (**SK4454**)	N	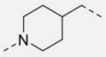	85 / 90	8	35	8.09 ± 0.10	7.46 ± 0.09	> 120	437	< 4.1
PROTAC 33 (**SK5527**)	N	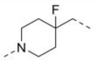	87 / 90	2	42	8.68 ± 0.10	7.38 ± 0.11	> 120	582	4.4
PROTAC 34	N	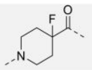	83 / 91		84		7.08 ± 0.08			
PROTAC 35	CH	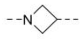	62 / 69		332		6.48 ± 0.07			
PROTAC 36	CH	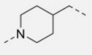	58 / 78		60		7.22 ± 0.13			
PROTAC 37	CH	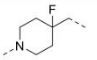	84 / 86		45		7.35 ± 0.10	> 120	617	4.5
PROTAC 38	CH	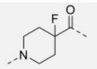	82 / 90		146		6.84 ± 0.05			

blank = not determined

Supplementary Table S5. ***In vitro*** **physicochemical and ADME properties of PROTACs SK4454 and SK5527.**

	SK4454	SK5527
**Aqueous Solubility** (µM)		
PBS, pH 7.4	19.2	4
Fasted simulated gastric fluid, pH 2.0	15.8	175
Fasted simulated intestinal fluid, pH 6.5	37.3	28.9
**LogD** (n-octanol/PBS, pH 7.4)	2.9	3.8
**Plasma protein binding** (%)		
Human	97.7	99.4
Rat	99.0	98.5
Mouse	98.6	98.6
**Permeability Caco-2,** pH 6.5/7.4 (10^{-6} cm/s)		
A -> B without/with verapamil (% recovery)	1.4 (49%) / -	0.7 (34%) / 0.7 (28%)
B -> A without/with verapamil (% recovery)	10.2 (135%)/ -	4.2 (29%) / 2.2 (27%)
Efflux ratio	7.3 / -	6.0- / 3.1
**Intrinsic clearance microsomes** (µL/min/mg)		
Human	207	630
Rat	< 120	149
Mouse	437	582
**Intrinsic clearance hepatocytes** (µL/min/10^6 cells)		
Human	-	14.8
Rat	-	27.1
Mouse	< 4.1	4.4

Supplementary Table S6. shRNA sequences used to target *ABCB1* and a non-targeting control.

**Target**	**Sequence**
ABCB1	CCGAACACATTGGAAGGAAAT
NT (non-targeting control)	CAACAAGATGAAGAGCACCAA

Supplementary Table S7. Antibodies used in this study.

**Antibodies used**	**Brand, Catalog#**
Human Aurora A Antibody	Bio-Techne, #AF3295
N-Myc Antibody (B8.4.B)	Santa Cruz Biotechnologies, #sc-53993
MDR1/ABCB1 (D3H1Q)	Cell Signaling Technologies, #12683
Anti-CRBN Antibody	Atlas Antibodies, #HPA045910
Vinculin (E1E9V) XP® Rabbit mAb	Cell Signaling Technologies, #13901
Anti-Vinculin Antibody	Sigma-Aldrich; #V9131
GAPDH Antibody (0411)	Santa Cruz Biotechnologies, #sc47724
Goat IgG HRP-conjugated Antibody	Bio-Techne, HAF109
Anti-rabbit IgG, HRP-linked Antibody	Cell Signaling Technologies, #7074
Anti-mouse IgG, HRP-linked Antibody	Cell Signaling Technologies, #7076
Anti-Rabbit Detection Module	Bio-Techne, #DM-001
Anti-Mouse Detection Module	Bio-Techne, #DM-002

Supplementary Figures

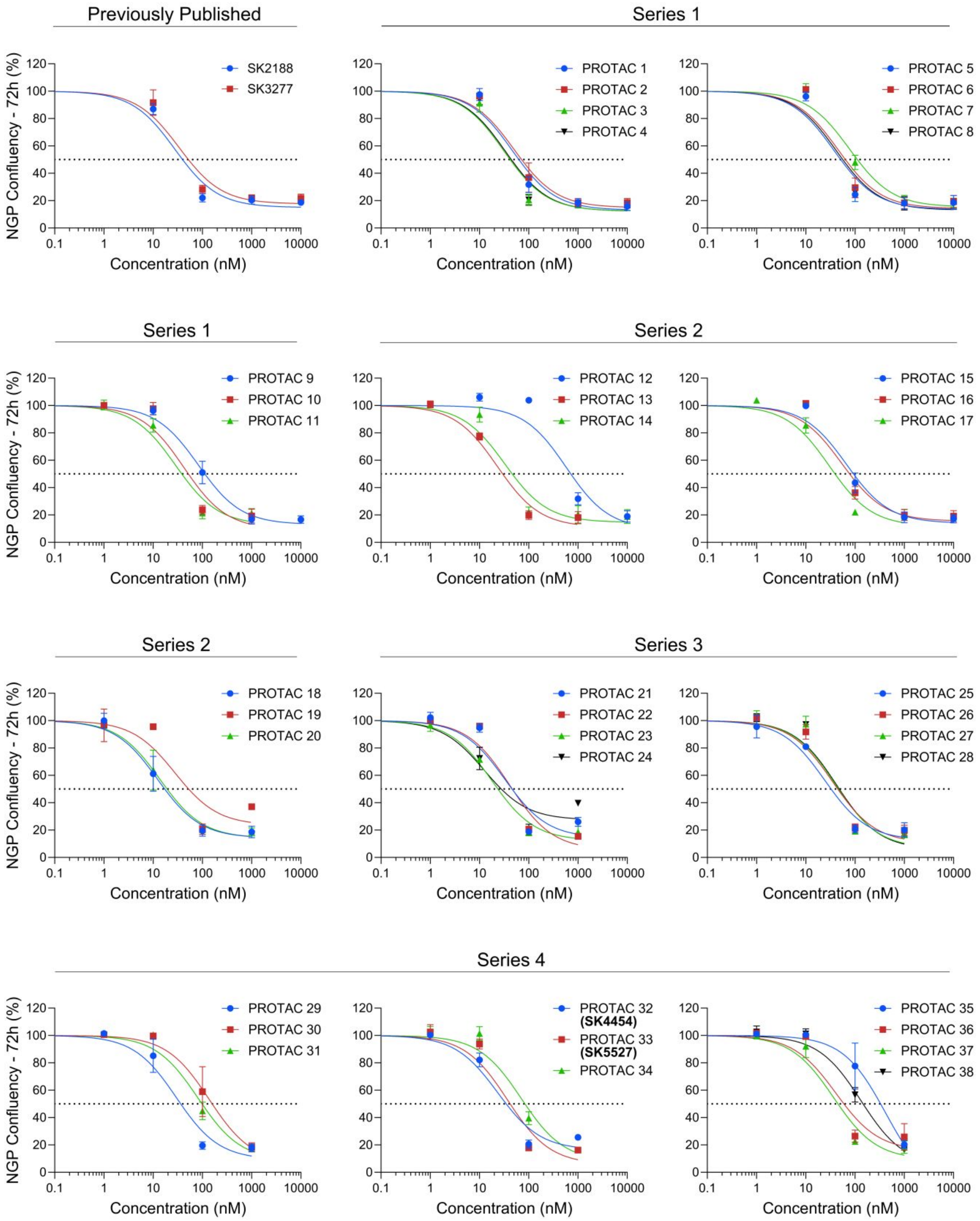

Supplementary Figure S1. ***In vitro*** **growth-inhibition screening of all PROTACs in NGP using IncuCyte® live-cell imaging.** Dose-response curves of all tested PROTACs in NGP at 72h post-treatment. Data represents percent cell confluency relative to the DMSO control (mean ± SD) (n = 3).

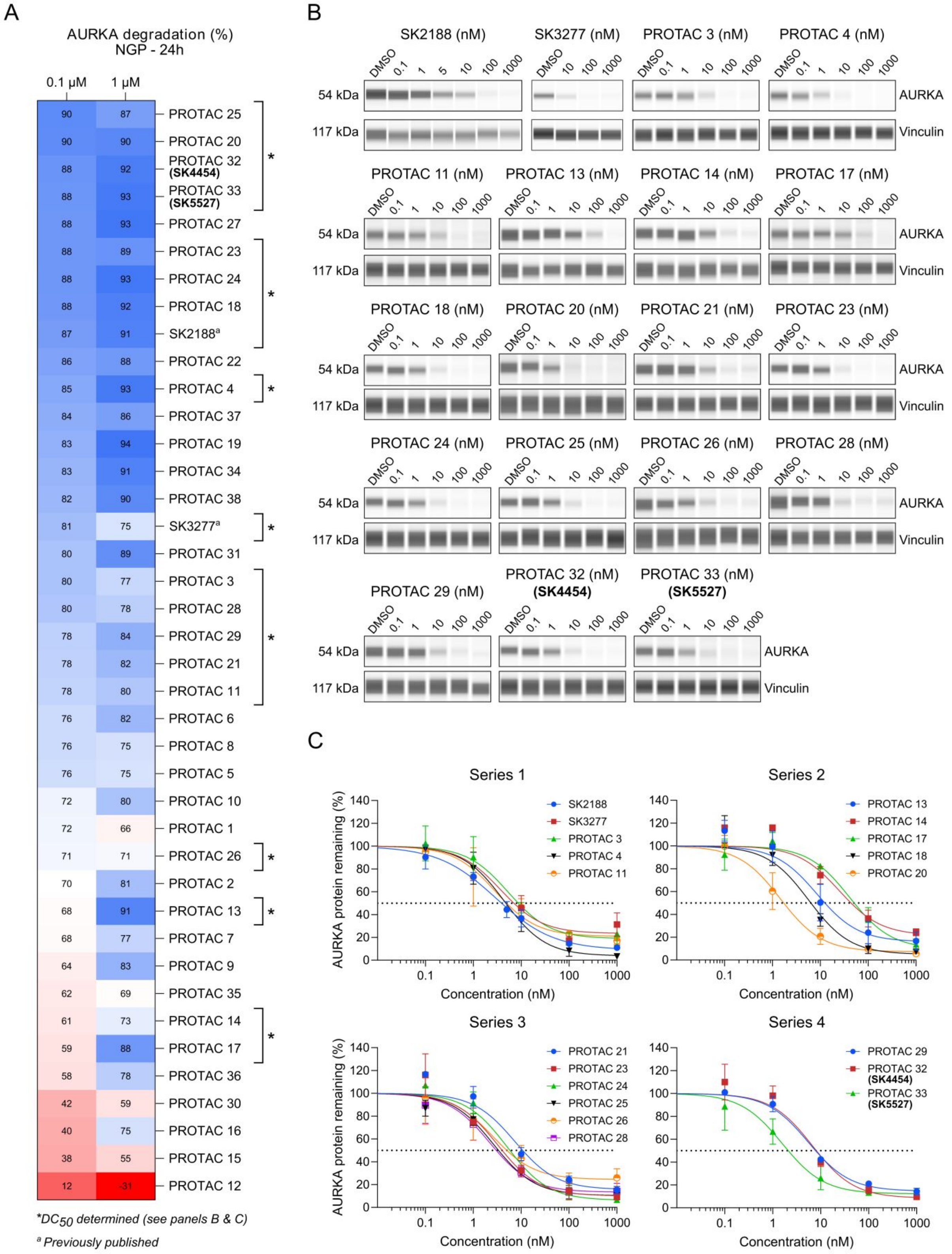

Supplementary Figure S2. ***In vitro*** **AURKA degradation screening of all PROTACs in NGP using Simple Western.** **(A)** Heat map depicting AURKA degradation (%) of all PROTACs following 24h treatment with 0.1µM and 1µM (n = 1). **(B)** Representative Simple Western lane-views of selected PROTACs, for which degradation potency (DC_{50} & D_{max}) was more accurately measured by testing a range of concentration points (n = 3). **(C)** Dose-response curves of the quantified Simple Western data used to derive DC_{50} and D_{max} values, showing percent AURKA protein remaining relative to a DMSO control (mean ± SD) (n = 3).

A

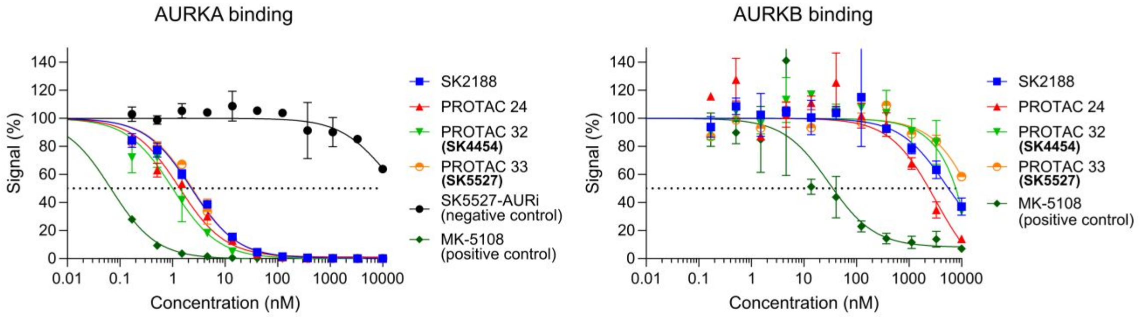

	K_i AURKA (nM)	pK_i AURKA ± SEM	K_i AURKB (nM)	pK_i AURKB ± SEM	Selectivity AURKA/AURKB
SK2188	2	8.64 ± 0.03	5606	5.25 ± 0.10	2803
PROTAC 24	1	8.86 ± 0.04	2390	5.62 ± 0.12	2390
PROTAC 32 (**SK4454**)	1	9.00 ± 0.05	7644	5.12 ± 0.05	7644
PROTAC 33 (**SK5527**)	2	8.62 ± 0.03	> 10000	< 5.00	>5000
SK5527-AURi	> 10000	< 5.00	NA	NA	NA
MK-5108	0.07	10.17 ± 0.02	36	7.45 ± 0.23	530

B

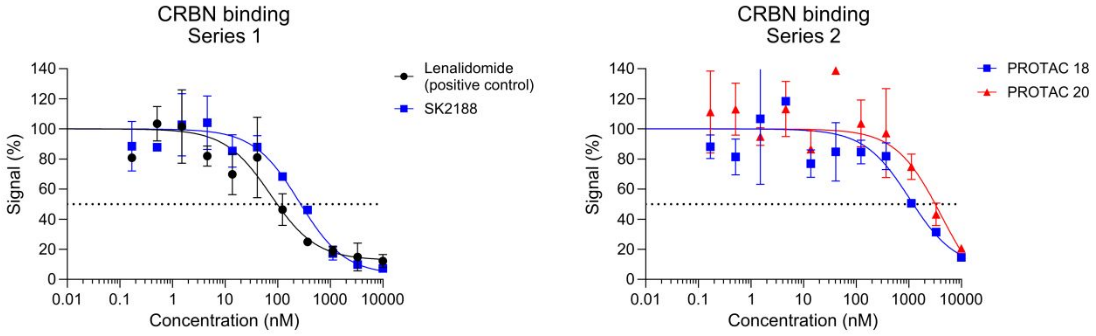

	K_i CRBN (nM)	pK_i CRBN ± SEM
Lenalidomide	98	7.01 ± 0.12
SK2188	273	6.56 ± 0.08

	K_i CRBN (nM)	pK_i CRBN ± SEM
PROTAC 18	1213	5.92 ± 0.14
PROTAC 20	3181	5.50 ± 0.15

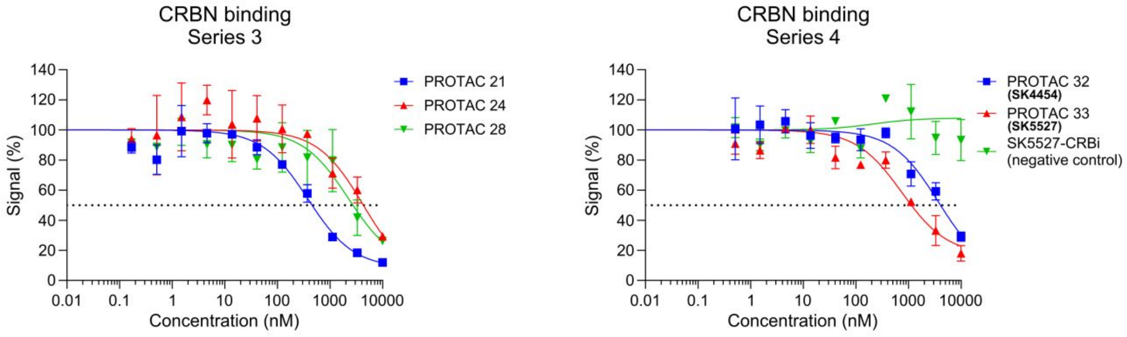

	K_i CRBN (nM)	pK_i CRBN ± SEM
PROTAC 21	455	6.34 ± 0.08
PROTAC 24	4304	5.37 ± 0.11
PROTAC 28	2724	5.56 ± 0.11

	K_i CRBN (nM)	pK_i CRBN ± SEM
PROTAC 32 (**SK4454**)	4060	5.39 ± 0.07
PROTAC 33 (**SK5527**)	1133	5.95 ± 0.09
SK5527-CRBi	NA	NA

Supplementary Figure S3. (A) Dose-response curves and K_i/pK_i values depicting AURKA and AURKB binding of selected PROTACs generated from a KINOMEscan binding assay (Eurofins Discovery). The non-AURKA binding inactive analogue SK5527-AURi and inhibitor MK-5108 were included as a negative and positive control, respectively (n = 2 replicate samples). **(B)** Dose-response curves and K_i/pK_i values depicting CRBN binding of selected PROTACs from series 1-4, measured using the E3scan ligand binding assay technology (Eurofins Discovery). The non-CRBN binding inactive analogue SK5527-CRBi and lenalidomide were included as a negative and positive control, respectively (n = 2 replicate samples).

A

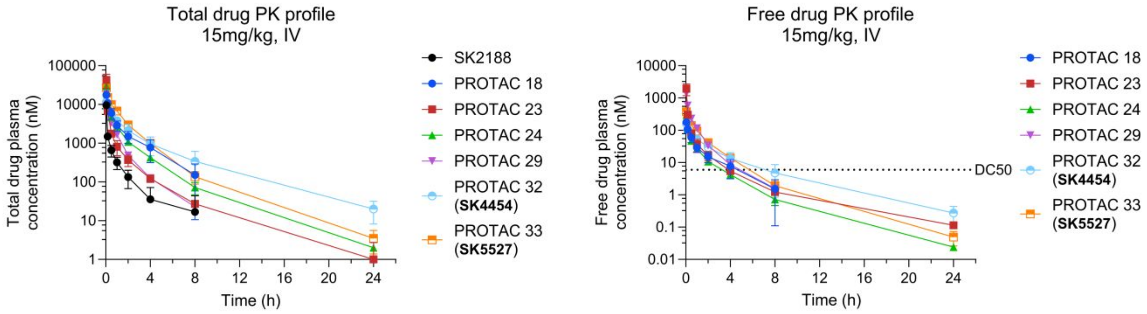

B

15mg/kg IV							
**PROTAC Name**	**Structure**	f_u	$t_{1/2}$ (h)	C_0 (ng/mL)	Vss (L/kg)	$AUC_{0\rightarrow\infty}$/D (ng*h*kg)/(ml*mg)	CL_{IV} (mL/min/kg)
SK2188	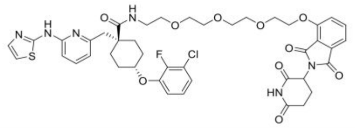	*	1.2 ± 0.7	18958 ± 2387	4.4 ± 2.2	153 ± 38	114 ± 27
PROTAC 18	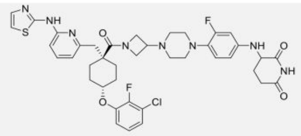	0.01	1.7 ± 0.4	17725 ± 3478	2.1 ± 0.5	773 ± 214	22.6 ± 5.8
PROTAC 23	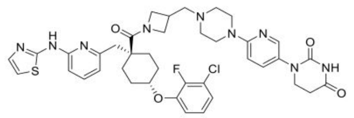	0.045	2.3 ± 1.2	77252 ± 38665	1.2 ± 0.6	528 ± 178	34.7 ± 14.1
PROTAC 24	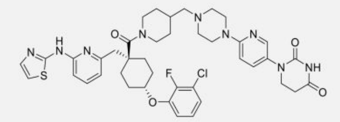	0.01	2.7 ± 0.1	37496 ± 6212	1.8 ± 0.4	747 ± 52	22.4 ± 1.5
PROTAC 29	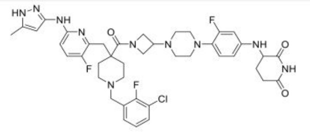	0.069	1.4 ± 0.1	23914 ± 3318	1.7 ± 0.3	446 ± 34	37.5 ± 3.0
PROTAC 32 (**SK4454**)	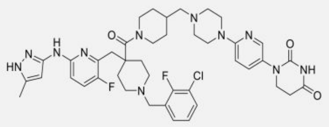	0.014	3.4 ± 0.3	11601 ± 1230	3.1 ± 0.4	978 ± 318	18.1 ± 5.1
PROTAC 33 (**SK5527**)	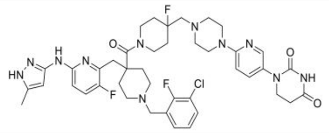	0.014	2.2 ± 0.8	29221 ± 4505	1.1 ± 0.1	1370 ± 18	12.2 ± 0.2

f_u = fraction unbound in plasma
$t_{1/2}$ = half-life
C_0 = Concentration at t = 0
V_{ss} = Volume of distribution at steady state
$AUC_{0\rightarrow\infty}$ = Area under the plasma concentration-time curve (extrapolated to infinity)
D = Dose
CL_{IV} = Clearance following intravenous administration

** = The plasma protein binding could not be determined due to significant reduced recovery.*

Supplementary Figure S4. ***In vivo* PK-parameters of various PROTACs after IV administration at a dose of 15 mg/kg in mice. (A)** Total and free drug plasma concentration curves as a function of time (mean ± SD) after IV administration at a dose of 15 mg/kg in mice (n = 3 mice). The dotted line represents the in vitro DC_{50} value in IMR-32. **(B)** Chemical structures and key PK-parameters corresponding to the total drug PK curves, obtained through non-compartmental analysis (NCA) using WinNonlin. Mouse plasma protein binding (PPB) was measured by equilibrium dialysis (*in vitro*).

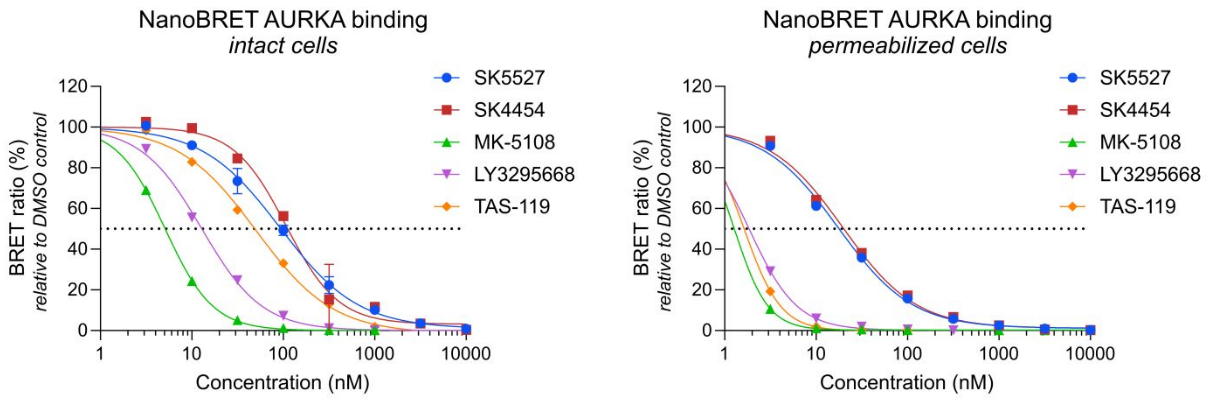

Compound	Intact Cells		Permeabilized Cells		IC_{50} Intact / IC_{50} Permeabilized
	**IC_{50} AURKA** (nM)	**pIC_{50} AURKA** ± SEM	**IC_{50} AURKA** (nM)	**pIC_{50} AURKA** ± SEM	
SK5527	94	7.03 ± 0.02	18	7.75 ± 0.02	5
SK4454	112	6.95 ± 0.04	20	7.70 ± 0.02	6
MK-5108	5	8.29 ± 0.01	1	8.89 ± 0.04	4
LY3295668	13	7.89 ± 0.01	2	8.73 ± 0.01	7
TAS-119	49	7.31 ± 0.02	2	8.79 ± 0.01	30

Supplementary Figure S5. *In vitro* AURKA binding measured using NanoBRET. Dose-response curves and AURKA IC_{50}/pIC_{50} values showing in-cell AURKA binding in both live (intact) and permeabilized HEK293T cells for PROTACs SK5527 and SK4454, as well as AURKA inhibitors MK-5108, LY3295668 and TAS-119, measured using a NanoBRET assay. Data is expressed as the average BRET ratio relative to the DMSO control (mean ± SD) (n = 2 replicate samples).

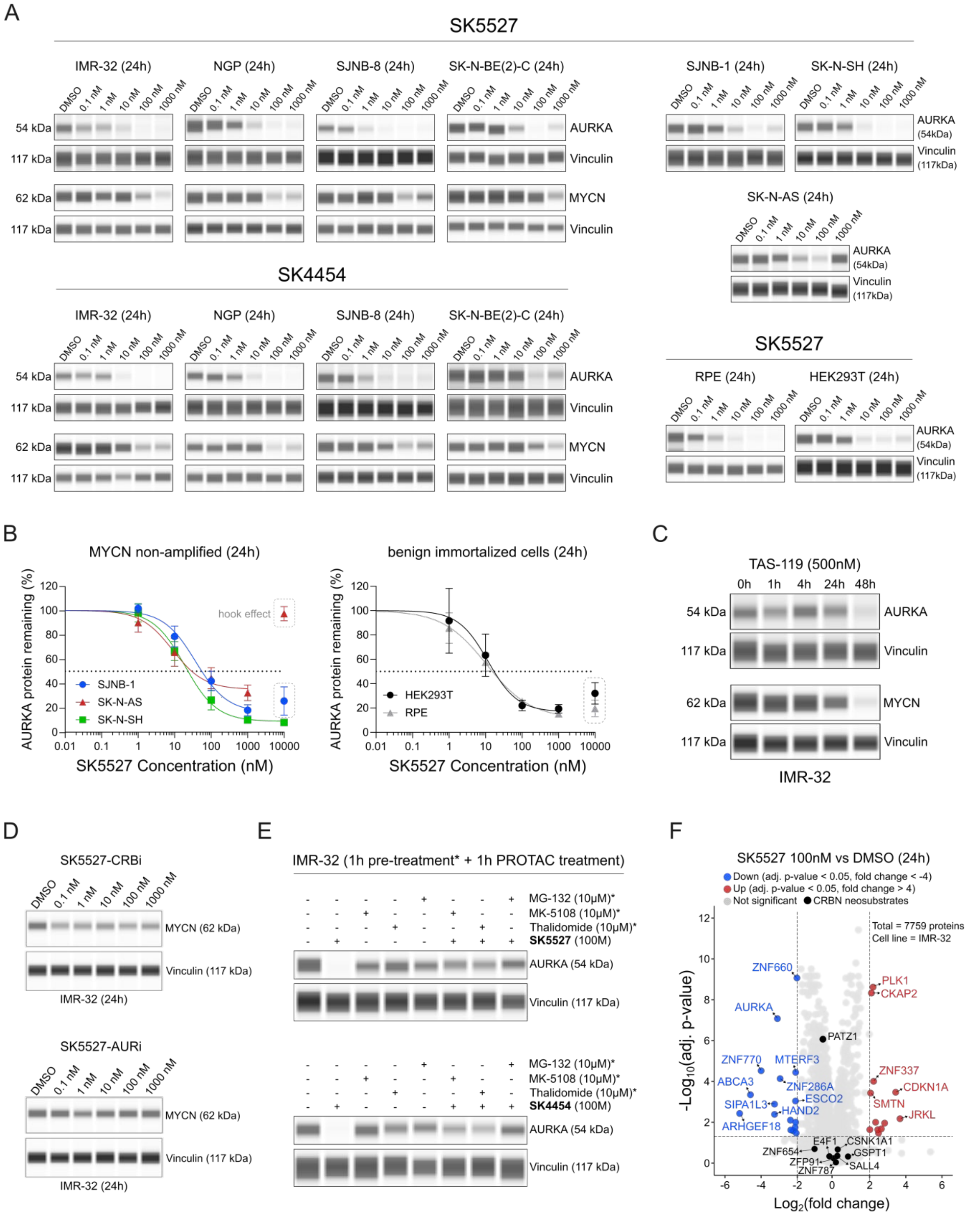

Supplementary Figure S6. **(A)** Representative Simple Western lane-views depicting AURKA protein levels across cell lines following 24h treatment with SK5527 or SK4454, using vinculin as loading control (n = 3). **(B)** Dose-response curves representing quantified Simple Western data of SK5527 treatment in *MYCN* non-amplified neuroblastoma and benign immortalized cell lines, expressed as changes in AURKA protein relative to DMSO control (mean ± SD) (n = 3). **(C)** Representative Simple Western lane-view showing changes in AURKA protein levels following treatment with TAS-119 (500nM) in IMR-32 at different timepoints, using vinculin as loading control (n = 3). **(D)** Representative Simple Western lane-views showing MYCN protein levels in IMR-32 following 24h treatment with the inactive analogues SK5527-CRBi and SK5527-AURi, using vinculin as loading control (n=3) **(E)** Representative Simple Western lane-views of IMR-32 in a competition binding assay through co-treatment with various compounds as displayed, using vinculin as loading control (n = 3). **(F)** LC-MS/MS proteome profiling of IMR-32 following 24h treatment with SK5527 (100nM) (n = 4).

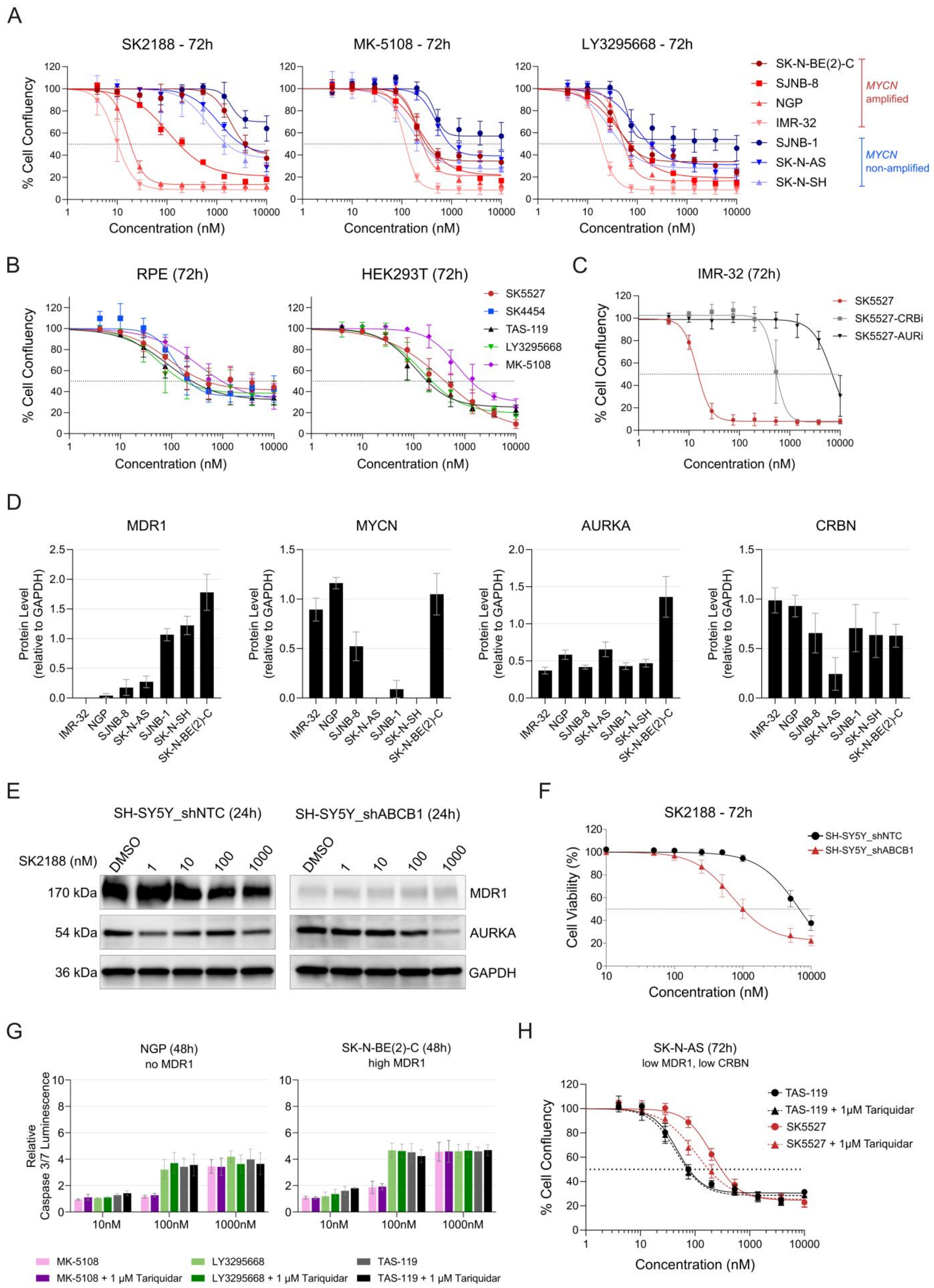

Supplementary Figure S7. (A) Dose-response curves representing percent cell confluency relative to a DMSO control (mean ± SD) as measured by IncuCyte® live-cell imaging at 72h after treatment with SK2188, and AURKA inhibitors MK-5108 and LY3295668 (n = 3). **(B)** Additional cell confluency dose-response curves in benign immortalized cell lines RPE and HEK293T cells for SK4454, SK5527 and AURKA inhibitors MK-5108, LY3295668 and TAS119, as well as **(C)** inactive analogues SK5527-CRBi and SK5527-AURi in IMR-32 (n = 3). **(D)** Quantification

of western blot data from Figure 4C, expressed as protein levels relative to loading control GAPDH (mean ± SD) (n = 3). **(E)** Representative western blot of SH-SY5Y with a stable knockdown of *ABCB1* (along with a Non-Targeting-Control model) showing changes in AURKA protein following treatment with SK2188 for 24h, using GAPDH as loading control. **(F)** Dose-response curve showing changes in cell viability of the same SH-SY5Y cell lines as in (E), following treatment with SK2188. Data was generated using the Alamar Blue Assay and is expressed as percent cell viability relative to a DMSO control (mean ± SD) (n = 3). **(G)** Caspase-Glo® 3/7 Assay showing induction of apoptosis in NGP and SK-N-BE(2)-C following 48h treatment with AURKA inhibitors (10, 100 & 1000 nM) with or without the addition of tariquidar (1μM). Bars represent caspase 3/7 luminescence relative to a DMSO control (mean ± SD) (n = 3). Statistical significance was calculated using multiple unpaired t-tests with correction for multiple testing using the Bonferroni-Dunn method (blank = non-significant). **(H)** Dose-response curves representing percent cell confluency relative to a DMSO control in SK-N-AS as measured by IncuCyte® live-cell imaging at 72h, following treatment with TAS-119 or SK5527 with or without the addition of tariquidar (1μM) (mean ± SD) (n = 3).

A

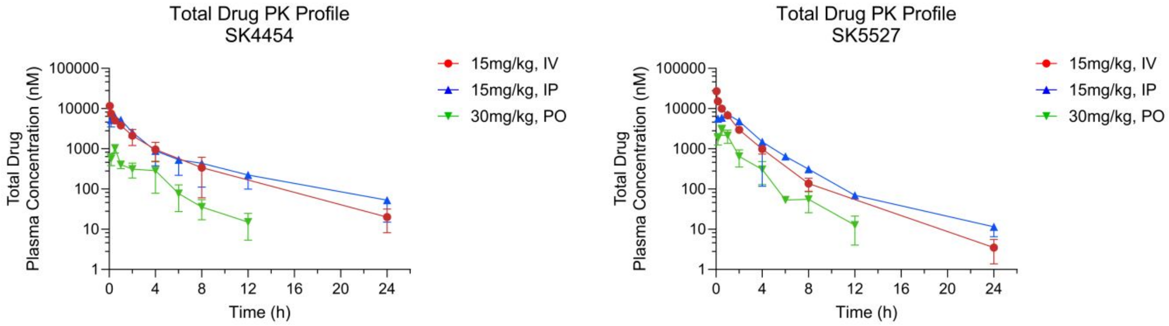

B

PROTAC Name	Mode of Administration & Dose	$t_{1/2}$ (h)	C_0 (ng/mL)	V_{ss} (L/kg)	$AUC_{0\rightarrow\infty}/D$ (ng*h*kg)/(mL*mg)	CL_{IV} (mL/min/kg)	t_{max} (h)	C_{max} (ng/mL)	F (%)
SK4454	IV, 15 mg/kg	3.4 ± 0.3	11601 ± 1230	3.1 ± 0.4	978 ± 318	18.1 ± 5.1	NA	NA	NA
	IP, 15 mg/kg	5.3 ± 0.3	NA	NA	1017 ± 390	NA	0.5	5178 ± 1686	104
	PO, 30 mg/kg	2.5 ± 0.2	NA	NA	64 ± 16	NA	0.5	856 ± 206	7
SK5527	IV, 15 mg/kg	2.2 ± 0.8	29221 ± 4505	1.1 ± 0.1	1370 ± 18	12.2 ± 0.2	NA	NA	NA
	IP, 15 mg/kg	2.7 ± 0.6	NA	NA	1264 ± 418	NA	1.0	6106 ± 347	92
	PO, 30 mg/kg	2.5 ± 1.6	NA	NA	152 ± 49	NA	0.5	2543 ± 715	11

$t_{1/2}$ = half-life
C_0 = Concentration at t = 0
V_{ss} = Volume of distribution at steady state
$AUC_{0\rightarrow\infty}$ = Area under the plasma concentration-time curve (extrapolated to infinity)
D = Dose
CL_{IV} = Clearance following IV
t_{max} = time to reach C_{max}
C_{max} = maximum concentration reached
F = bioavailability
NA = not applicable

C

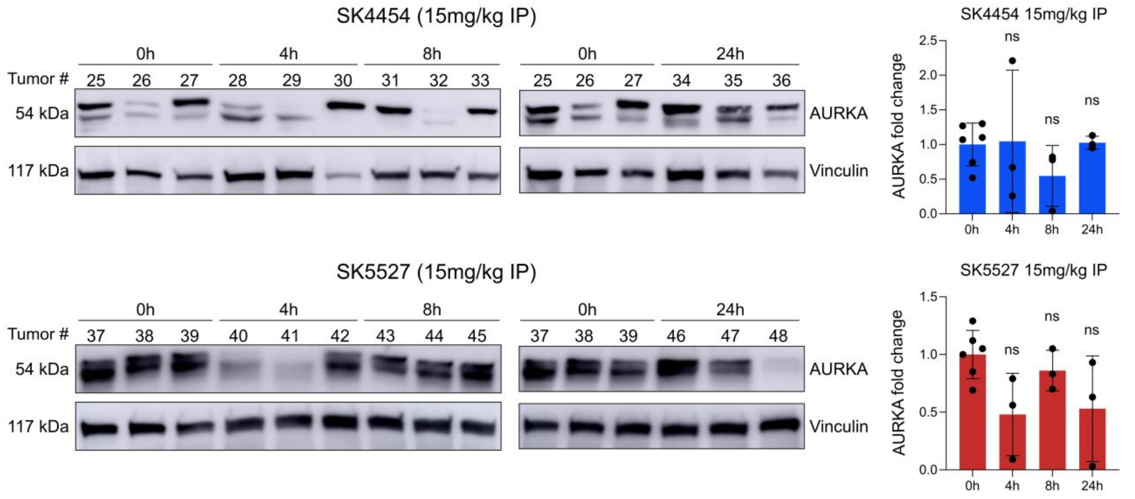

Supplementary Figure S8. (A) Total drug plasma concentration curves as a function of time (mean ± SD) for SK4454 & SK5527 following a single IV (15 mg/kg), IP (15 mg/kg) and PO (30 mg/kg) administration in mice (n = 3 mice) and **(B)** their corresponding key PK parameters. **(C)** Pharmacodynamics study of SK4454 and SK5527 in IMR-32 cell line-derived xenograft mice. Mice tumors were harvested at different timepoints following a single IP administration (15mg/kg). Changes in AURKA protein levels were visualized using western blot, with vinculin as loading control. Bars represent the quantified western blot data, representing AURKA fold change relative to the 0h controls (mean ± SD) (n = 3 mice per timepoint). Statistical significance was calculated using an Ordinary One-Way ANOVA with Dunnet's correction for multiple testing (ns = not significant).

Supplementary Methods 1 – LC-MS/MS proteomics

Sample Preparation.

From each sample, 100 µg of protein was isolated, the volumes were equalized with water and SDS and TEAB were added to a final concentration of 5% and 50 mM, respectively. Proteins were reduced and alkylated by addition of 10 mM Tris(2-carboxyethyl)phosphine hydrochloride and 40 mM chloroacetamide and incubation for 30 minutes at 25°C in the dark. Phosphoric acid was added to a final concentration of 1.2%, and samples were subsequently diluted 7-fold with binding buffer containing 90% methanol in 100 mM TEAB, pH 7.55. The samples were loaded on the 96-well S-Trap™ plate (Protifi), placed on top of a deepwell plate, and centrifuged for 2 min at 1,500 x g at RT. After protein binding, the S-trap™ plate was washed three times by adding 200 µl binding buffer and centrifugation for 2 min at 1,500 x g at RT. A new deepwell receiver plate was placed below the 96-well S-Trap™ plate and 50 mM TEAB containing trypsin (1/100, w/w) was added for digestion overnight at 37°C. Using centrifugation for 2 min at 1,500 x g, peptides were eluted in three steps, first with 80 µl 50 mM TEAB, then with 80 µl 0.2% formic acid (FA) in water and finally with 80 µl 0.2% FA in water/acetonitrile (ACN) (50/50, v/v). Eluted peptides were dried completely by vacuum centrifugation.

LC-MS/MS.

Peptides were re-dissolved in 20 µl loading solvent A (0.1% trifluoroacetic acid in water/acetonitrile (ACN) (99.5:0.5, v/v)) of which 2 µl of sample was injected for LC-MS/MS analysis on an Ultimate 3000 ProFlow nanoLC system in-line connected to a Orbitrap Exploris 240 mass spectrometer (Thermo) equipped with pneu-Nimbus dual ion source (Phoenix S&T). Trapping was performed at 20 µl/min for 2 min in loading solvent A on a PepMap™ Neo Trap column (Thermo scientific, 300 µm internal diameter (I.D.), 5 µm beads). The peptides were separated on a 250 mm Odyssey Ultimate, 1.7µm C18, 75 µm inner diameter (Ionopticks) kept at a constant temperature of 45°C. Peptides were eluted by a gradient reaching 26,4 % MS solvent B (0.1% FA in acetonitrile) after 80 min, 44% MS solvent B at 95 min, 56% MS solvent B at 100min followed by a 5-minutes wash at 56% MS solvent B and re-equilibration with MS solvent A (0.1% FA in water). The flow rate was set to 250 nl/min. The mass spectrometer was operated in data-independent mode, automatically switching between MS and MS/MS acquisition. Full-scan MS spectra ranging from 375-1500 m/z with a normalized target value of 300%, a maximum fill time of 25 ms and a resolution at of 60,000 were followed by 30 quadrupole isolations with a precursor isolation width of 10 m/z for HCD fragmentation at an NCE of 30% after filling the trap at a normalized target value of 2000% for maximum injection time of 45 ms. MS2 spectra were acquired at a resolution of 15,000 with a scan range of 200-1800 m/z in the Orbitrap analyser without multiplexing. The isolation intervals were set from 400 – 900 m/z with a width of 10 m/z using window placement optimization. The polydimethylcyclosiloxane background ion at 445.120028 Da was used for internal calibration (lock mass) and QCloud has been used to control instrument longitudinal performance during the project [2,3].

Data Analysis.

Analysis of the mass spectrometry data was performed in DiaNN (version 1.9). Precursor false discovery rate was set at 1%. Spectra were searched against the the Homo sapiens protein sequences in the Uniprot database (database release version of 2024_01), containing 20,597 sequences (www.uniprot.org) and the imbedded protein contaminant list. Enzyme specificity was set as C-terminal to arginine and lysine, also allowing cleavage at proline bonds with a maximum of 2 missed cleavages. Variable modifications were set to oxidation of methionine residues and acetylation of protein N-termini while fixed modification was set to carbamidomethylation of cystein residues. Matching between runs was enabled. Mainly default settings were used, except for the addition of a 400-900 m/z precursor mass range filter and MS1 and MS2 mass tolerance was set to 10 and 20 ppm respectively.

Further data analysis of the results was performed with an in-house script in the R programming language. Protein expression matrices were prepared as follows: the DIA-NN main report output table was filtered at a precursor and protein library q-value cut-off of 1 % and only proteins identified by at least one proteotypic peptide were retained. After pivoting into a wide format, iBAQ intensity columns were then added to the matrix using the DIAgui's R package get_IBAQ function. PG MaxLFQ intensities were log2 transformed and replicate samples were grouped. Proteins with less than 3 valid values in at least one group were removed and missing values were imputed from a normal distribution centered around the detection limit (package DEP) leading to a list of 7,759 quantified proteins in the experiment, used for further data analysis. To compare protein abundance between pairs of sample groups3h SK5527 vs 3h DMSO; 3h MK-5108 vs 3h DMSO; 24h SK5527 vs 24h DMSO; 24h MK-5108 vs 24h DMSO sample groups), statistical testing for differences between two group means was performed, using the package limma. Statistical significance for differential regulation was set to a false discovery rate (FDR) of < 0.05 and $|\log_2 FC| = 2$.

Supplementary Methods 2 – *in vitro* physicochemical and ADME properties

General

All *in vitro* physicochemical and ADME determinations were performed by Eurofins Villapharma.

Aqueous solubility

The test compound is prepared at 200 µM in the corresponding buffer from a 10 mM DMSO stock solution. The final DMSO concentration is 2 %. The buffer samples are mixed thoroughly followed by incubation at room temperature for 24 h. At the end of the incubation, the buffer samples are centrifuged for 30 min and supernatants are then injected for the LC/UV/MS analysis. The used buffers were phosphate buffered saline (PBS) pH 7.4 (Gibco™ 10010031 (without Ca, without Mg)), simulated gastric fluid (NaCl 34.2 mM, HCl 84.7 mM, pepsin 3.2 g/L, pH ± 2) and simulated intestinal fluid (KH_2PO_4 50 mM, NaOH 38 mM, pancreatin 10 g/L, pH 7.5). Aqueous solubility (µM) was determined by comparing the peak area of the principal peak in a calibration standard (200 µM) containing organic solvent (methanol/water, 60/40, v/v) with the peak area of the corresponding peak in a buffer sample. In addition, chromatographic purity (%) was defined as the peak area of the principal peak relative to the total integrated peak area in the HPLC chromatogram of the calibration standard.

Lipophilicity (LogD).

The partition coefficient between n-octanol and PBS buffer (pH 7.4) was determined using the shake-flask method in a 96-well plate format by LC/UV/MS analysis. The samples are mixed for 120min at room temperature and centrifuged for 30 minutes prior to LC-MS analysis. The test compound is prepared at 100 µM from a 10 mM DMSO stock solution. The total amount of compound was determined as the peak area of the principal peak in a calibration standard (100 µM) containing organic solvent (methanol/water, 60/40, v/v). The amount of compound in buffer was determined as the combined, volume-corrected, and weighted areas of the corresponding peaks in the aqueous phases of three organic-aqueous samples of different composition (4 µL of 1/1 v/v n-octanol/PBS, 20 µL of 1/1 v/v n-octanol/PBS and 20 µL of 6/55 v/v n-octanol/PBS). An automated weighting system was used to ensure the preferred use of raw data from those samples with well quantifiable peak signals. The amount of compound in the organic phase was calculated by subtraction. Subsequently, Log D was calculated as the Log_{10} of the amount of compound in the organic phase divided by the amount of compound in the aqueous phase.

Plasma protein binding (PPB).

PPB was determined via equilibrium dialysis in a 96-well plate in a dialysis block constructed from Teflon. Plasma from human or animal species (rat or mouse) is used as the protein containing matrix. The protein matrix is spiked with the test compound at 10 µM (n=2) with a final DMSO concentration of 1%. The dialysate compartment is loaded with phosphate buffered saline (PBS, pH 7.4) and the sample side is loaded with equal volume of the spiked protein matrix. The dialysis plate is then sealed and incubated at 37°C for 4 h. After the incubation, samples are taken from each compartment, diluted with the phosphate buffer followed by addition of acetonitrile and centrifugation. The supernatants are then used for LC-MS/MS analysis. A control sample (n=2) is prepared from the spiked protein matrix in the same manner as the assay samples (without dialysis). This control sample serves as the basis for the recovery determination. The percent bound and recovery are calculated as follow:

$$PPB\ (\%) = \frac{Area_p - Area_b}{Area_p} * 100$$

$$Recovery\ (\%) = \frac{Area_p + Area_b}{Area_c} * 100$$

$Area_p$ = peak area of analyte in the protein matrix; $Area_b$ = peak area of analyte in the assay buffer; $Area_c$ = peak area of the analyte in the control sample.

Caco-2 permeability assay.

Cells are seeded at 1.2 x 10^5 cells/cm^2 in 96-well Multiscreen™ plates (Corning). Permeability assays are performed with the cells at days 21-25 post seeding. Cells are typically used for 15 consecutive passages in culture. This assay is performed in both the apical to basolateral (A-B) and B-A direction. The test compound is prepared at 10 µM in HBSS-MES (pH 6.5) or HBSS-HEPES (pH 7.4) with a final DMSO concentration of 1 %. The assay plate is incubated at 37°C with gentle shaking for 60 min or 40 min for the A-B or B-A assay, respectively. For assays tested in the presence of verapamil, 100 µM verapamil is included on both the A and B sides. Samples are aliquoted from the donor side at time zero and the end point, and from the receiver side at the end point. Samples are analyzed by HPLC-MS/MS using selected reaction monitoring. Fluorescein permeability is assessed in the A-B direction at pH 7.4 on both sides after the permeability assay with the test compound. The cell monolayer with a fluorescein permeability of less than 1.5 x 10^{-6} cm/s was considered intact. The apparent permeability coefficient (P_{app}) of the test compound and its recovery (%) are calculated as follows:

$$P_{app} = \frac{V_R * C_{R,end}}{\Delta T * A * (C_{D,mid} - C_{R,mid})}$$

$$Recovery\ (\%) = \frac{V_D * C_{D,end} + V_R * C_{R,end}}{V_D * C_{D,t=0}} * 100$$

Supplementary Methods – Chemistry

GENERAL EXPERIMENTAL DETAILS

All reactions described were performed in a fume hood and were carried out under a nitrogen atmosphere at room temperature (21 °C) unless explicitly stated otherwise.

Reactions were monitored by TLC analysis using Machery Nagel ALUGRAM Xtra SIL G UV254 sheets. Visualization of material on the TLC plate was carried out by inspection under UV light (254 nm) and/or staining the plates with one of the following spray reagents: A: a solution of $(NH_4)_6Mo_7O_{24}{\cdot}4H_2O$ (25 gL^{-1}) and $(NH_4)_4Ce(SO_4)_4{\cdot}2H_2O$ (10 gL^{-1}) in 10% aqueous sulfuric acid (v/v) followed by heating with a heat gun; B: an aqueous solution of KMn04 (20 gL^{-1}) and K_2CO_3 (10 gL^{-1}).

LC-MS analysis for reaction monitoring and purity analyses were carried out on a Waters Autopurification system equipped with a Waters CORTECS column (4.6x100 mm, C18, 2.7µm) and using a water/acetonitrile/formic acid linear gradient. Peak detection was achieved using mass spectrometry (ESI) and a photo-diode-array detector (PDA)

Column chromatography was performed on a Reveleris X2 automated flash chromatography system (Grace/Büchi) using disposable 60Å silica gel cartridges (Agela).

Nuclear magnetic resonance (NMR) analyses including ^1H- and ^{13}C- spectra were carried out on a Bruker Avance Neo 400MHz spectrometer equipped with an autosampler and using TOPSPIN/ICON-NMR. Chemical shifts are given in ppm (δ) relative to the solvent peak. NMR solvents included $CDCl_3$ (7.26 ppm in ^1H NMR, 77.16 ppm in ^{13}C NMR) or DMSO-d6 (2.50 ppm in ^1H NMR and 39.52 ppm in ^{13}C NMR) and were all purchased from Euriso-Top (Saint Aubin, France).

High-resolution mass spectrometry was performed on a Waters Premier XE HRMS system that is calibrated using a solution of Le-enkephalin. Infusion of the analyte into the HRMS system was done as a solution (0.5 $ngmL^{-1}$) in UPLC grade water and acetonitrile.

All solvents utilized were used as received, were purchased from Chemlab (Zedelgem, Belgium), and were of HPLC grade or equivalent or superior purity. All building blocks and reagents were used as received and were purchased from common chemical suppliers including but not limited to: Fluorochem, Apollo Scientific, Sigma-Aldrich, and Fisher Scientific.

All obtained final compounds had purity > 95%, as assayed by analytical HPLC (UV) using a linear gradient Water/ MeCN 0 -> 98 % + 0.02 % FA in 10 min on a Waters CORTECS column (4.6x100 mm, C18, 2.7µm).

GENERAL PROCEDURES

General procedure 1 for linker alkylation with ethyl bromoacetate

The NHBoc-Linker-OH (1 eq) was dissolved in THF, cooled down to 0°C, followed by step wise addition of NaH (1.1 eq, 60% dispersion). After 30 minutes of stirring, ethyl bromoacetate (1.1 eq) was added. After 4h, the RM was quenched by addition of sat. NH_4Cl solution until pH 7 and extracted twice with EA. The organic phase was dried over Na_2SO_4, filtered and evaporated. Flash column chromatography delivered the intermediates.

General procedure 2 for ester hydrolysis

The ethyl esters (1 eq) were treated with 4M NaOH solution (5 eq) in MeOH for 24h. The reaction was quenched with 4M HCl until pH of around 2 to 3 was reached, followed by an extraction with DCM (2 to 3 times). The organic phase was dried over Na_2SO_4, filtered and evaporated. Drying *in vacuo* delivered the carboxylic acid intermediates.

General procedure 3 for HATU-mediated amide coupling

The acid (1.0 eq) and HATU (1.2 eq) were dissolved in DMF, DIPEA (5 eq) was added and stirred for 5 minutes. Then, the amine was dissolved in DMF and added to the activated acid. Upon completion of the reaction, the RM was transferred to a separation funnel with DCM and washed with sat. $NaHCO_3$ and brine. The organic phase was dried over Na_2SO_4, filtered and evaporated. Flash column chromatography delivered the amides.

General procedure 4 for S_N2 substitution of 5-piperazine-thalidomide

The appropriate linker (1.5 eq) was dissolved in DCM, Et_3N (2 eq) was added and the solution cooled to 0°C. Next, methanesulfonyl chloride (1.7 eq) was added and stirred for 1h at RT. The mixture was evaporated, redissolved in EA and filtered. The filtrate was collected, evaporated and redissolved in DMF. 5-(*N*-Boc-piperazine)thalidomide was treated with TFA/ DCM (1/2) for 1h, evaporated, redissolved in DMF and added to the mesylated linker together with DIPEA (4 eq) and NaI (0.1 eq). After heating for 16h at 70-80°C, sat. $NaHCO_3$ was added and extracted 2 times with EA. The organic layers were dried over Na_2SO_4, filtered and evaporated. Flash column chromatography (DCM/MeOH 0 -> 5% + 0.1% NH_4OH) delivered the products.

General alcohol oxidation procedure 5

The alcohol (1.0 eq) was dissolved in DCM and cooled down to 0°C, followed by addition of Dess-Martin periodinane (1.2 eq). After 5h, sat $NaHCO_3$ and 2M NaS_2O_4 were added and extracted twice with DCM. The organic layer was dried over Na_2SO_4, filtered and evaporated. Flash column chromatography (PE/EA 10 -> 60%) delivered the aldehydes

General reductive amination procedure 6

The amine (1 eq) and the appropriate aldehyde or keton (1.2 eq) was dissolved in DMF. The pH is adjusted to +/- 6 with either $NaHCO_3$ or AcOH, followed by addition of STAB (1.3 eq). When LC-MS indicated completion of the reaction (often after 4 to 6h), sat $NaHCO_3$ was added, followed by extraction with DCM. The organic layer was dried over Na_2SO_4, filtered and evaporated. Flash column chromatography (DCM/MeOH 0 -> 5% + 0.1% NH_4OH) delivered the intermediates.

General procedure 7 for PROTAC synthesis (HATU coupling) :

The Boc-protected amine (0.12 mmol, 1.2 eq) was dissolved in DCM (2 mL) and TFA (1 mL) and stirred for 1.5 h. Then, the volatiles were evaporated. MK-5108 (50 mg, 0.10 mmol, 1.0 eq) and HATU (46 mg, 0.12 mmol, 1.2 eq) were dissolved in DMF (2 mL), DIPEA (0.087 mL, 5 eq) was added and stirred for 5 minutes. Then, the amine was dissolved in DMF (2 mL) and added to the activated acid. After approximate 30 min, the RM was transferred to a separation funnel with DCM and washed with sat. $NaHCO_3$ (x2) and Brine (x1). The organic phase was dried over Na_2SO_4, filtered and evaporated. Flash column chromatography using DCM/MeOH 0 -> 10% delivered the final products. All PROTACs showed purity of > 95% as measured by HPLC analysis.

SYNTHESIS OF SERIES 1 PROTACS

Scheme S1. Synthesis of piperazine-containing PROTAC analogues of SK2188

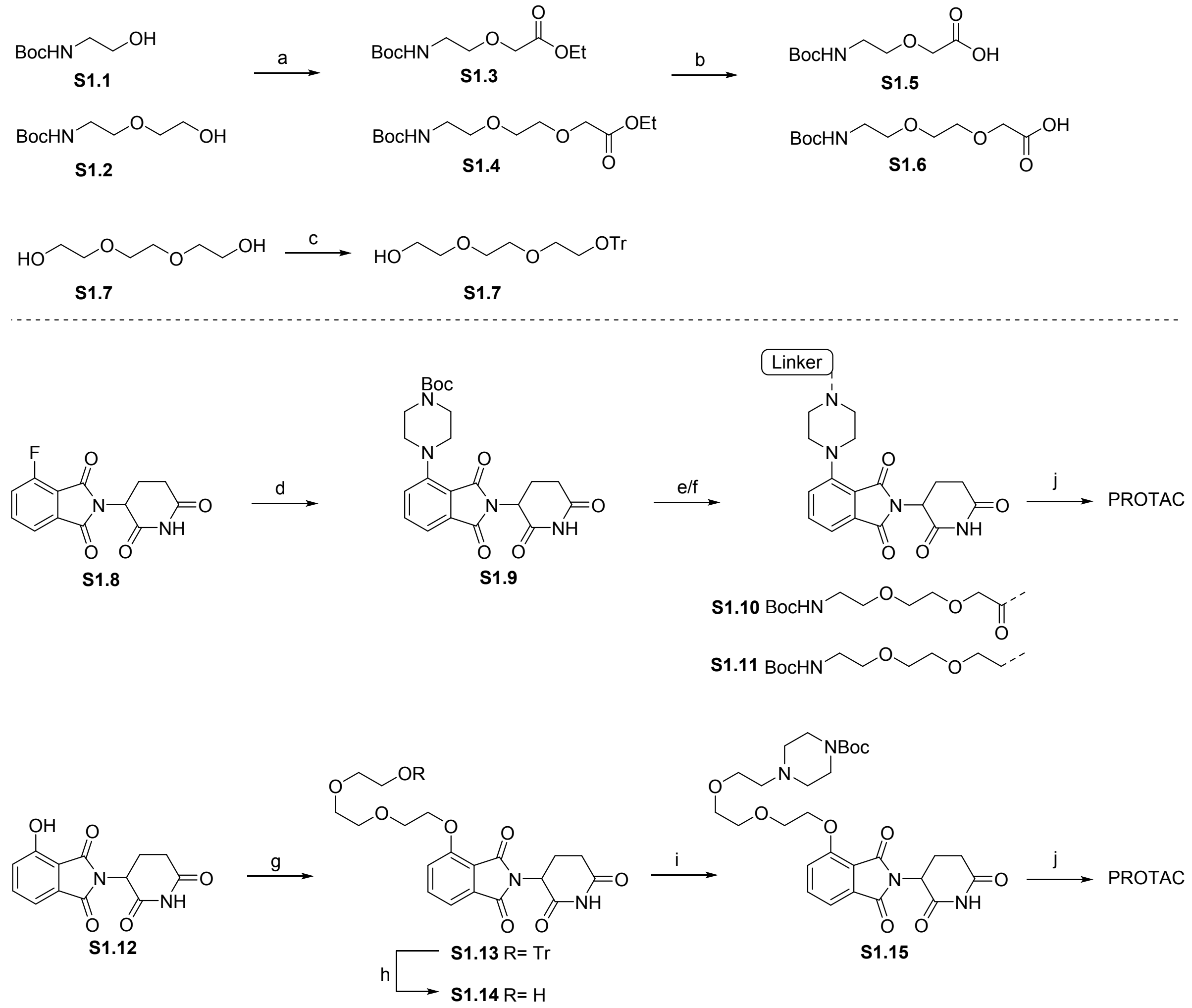

Reagents and conditions: **a)** NaH, ethyl bromoacetate, THF, 0 °C, 42% (S1.3), 38% (S1.4); **b)** NaOH, MeOH/Water, 80% (S1.5), 97% (S.1.6); **c)** Trityl chloride, Et_3N, DMAP, DCM, 61%; **d)** Boc-piperazine, DIPEA, dioxane, 100 °C, 63%, **e)** for (S2.10) i. TFA, DCM, ii. Appropriate linker, HATU, DIPEA, DMF, 98%; **f)** for (S2.11) i. TFA, DCM, ii. Appropriate linker, Ms_2O, Et_3N, DCM, iii. Et_3N, DMF, 80 °C, 35%; **g)** Appropriate linker-OH, DBAD, PPh_3, THF; **h)** Camphorsulfonic acid, MeOH, 76% over 2 steps; **i)** i. Ms_2O, Et_3N, DCM, ii. Boc-piperazine, Et_3N, DMF, 80 °C, 29%; **j)** i. TFA, DCM, ii. MK-5108, HATU, DIPEA, DMF.

Scheme S2. Synthesis of piperazine-containing PROTAC analogues of SK3277

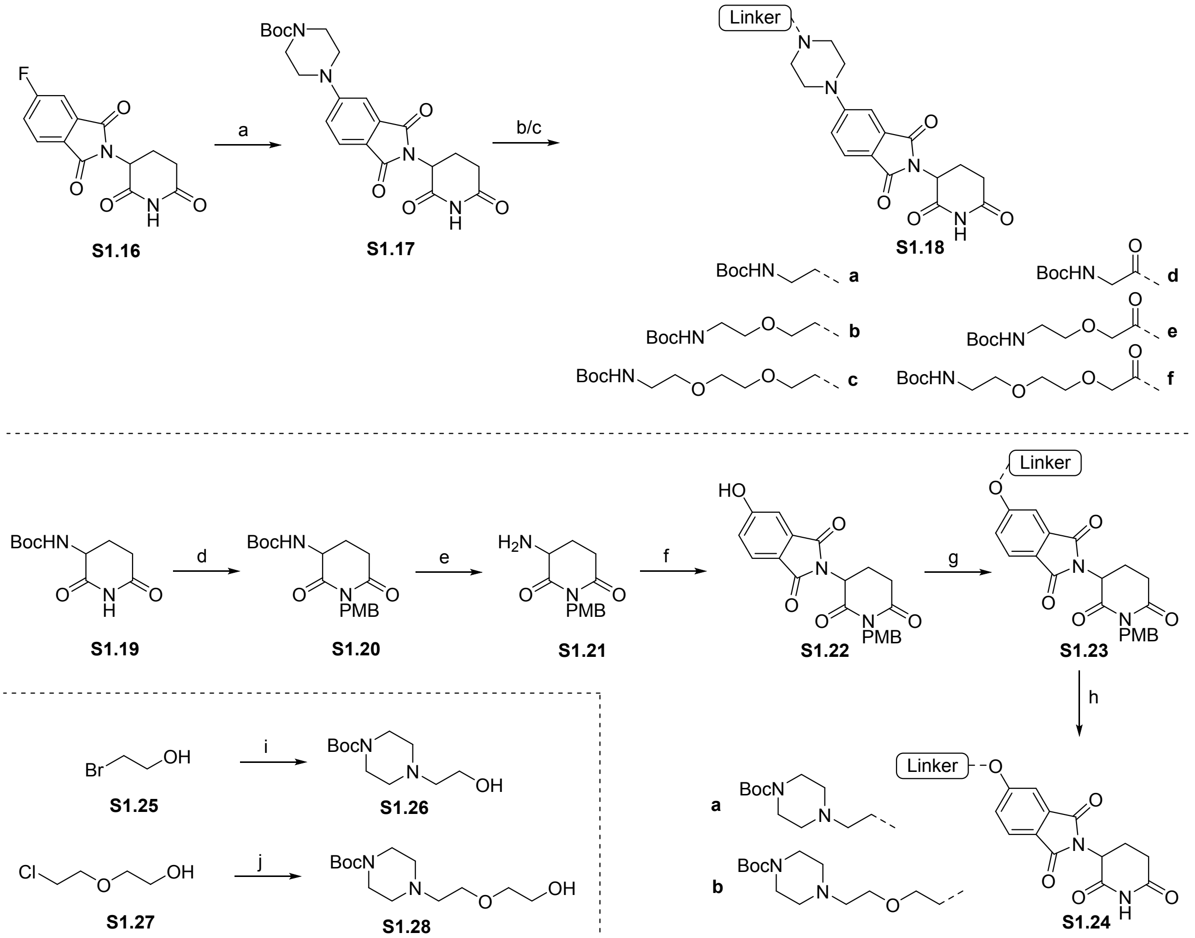

Reagents and conditions: ***a)*** *Boc-piperazine, DIPEA, DMSO, 74%;* ***b)*** *Mesylated linker or* tert-*butyl (2-bromoethyl)carbamate, DIPEA, DMF, 70 °C, 58% (a), 29% (b), 17% (c),* ***c)*** *HATU, DIPEA, DMF, 86% (d); 54% (e), 70% (f);* ***d)*** *4-methoxybenzyl alcohol, DBAD, PPh_3, THF, 67%,* ***e)*** *4M HCl in dioxane, 93%;* ***f)*** *4-hydroxyphtalic acid, pyridine, 140°C, 66%;* ***g)*** *Appropriate linker-OH, DBAD, PPh_3, THF, 91% (a), 45% (b);* ***h)*** *CAN, MeCN/ H_2O, 50% (a), 48% (b); i)Boc-piperazine, K_2CO_3, DMF, 80 °C, 49%; j)Boc-piperazine, K_2CO_3, NaI, DMF, 80 °C, 55%.*

Synthesis of ethyl 2-(2-((*tert*-butoxycarbonyl)amino)ethoxy)acetate

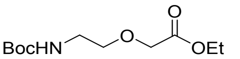

Following general procedure 1, 3.24 g of the product was obtained in 42% yield.

^{1}H NMR ($CDCl_3$, 400MHz): δ = 1.29 (t, J = 7.2 Hz, 3H), 1.44 (s, 9H), 3.34 (t, J = 4.7 Hz, 2H), 3.61 (t, J = 5.1 Hz, 2H), 4.08 (s, 2H), 4.22 (q, J = 7.1 Hz, 2H) ppm.

^{13}C NMR ($CDCl_3$, 100MHz): δ = 14.3 (CH3), 28.5 (CH3), 40.6 (CH2), 61.1 (CH2), 69.4 (CH2), 70.9 (CH2), 79.5 (C), 156.1 (C), 170.6 (C) ppm.

Synthesis of ethyl 2,2-dimethyl-4-oxo-3,8,11-trioxa-5-azatridecan-13-oate

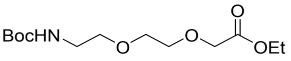

Following general procedure 1, 2.18 g of the product was obtained in 38% yield.

^{1}H NMR ($CDCl_3$, 400MHz): δ = 1.28 (t, J = 7.2 Hz, 3H), 1.44 (s, 9H), 3.31 (t, J = 5.0 Hz, 2H), 3.54 (t, J= 5.1 Hz, 2H), 3.63-3.68 (m, 2H), 3.69-3.74 (m, 2H), 4.13 (s, 2H), 4.22 (q, J = 7.1 Hz, 2H) ppm.

^{13}C NMR ($CDCl_3$, 100MHz): δ = 14.3 (CH3), 28.5 (CH3), 40.6 (CH2), 61.0 (CH2), 68.1 (CH2), 70.5 (CH2), 70.5 (CH2), 71.0 (CH2), 79.4 (C), 156.1 (C), 170.6 (C) ppm.

Synthesis of 2-(2-((*tert*-butoxycarbonyl)amino)ethoxy)acetic acid

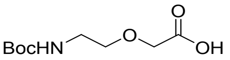

Following general procedure 2 for ester hydrolysis, 2.31 g of the product was obtained in 80% yield.

^{1}H NMR ($CDCl_3$, 400MHz): δ = 1.44 (s, 9H), 3.34 (t, J = 4.7 Hz, 2H), 3.63 (t, J = 5.1 Hz, 2H), 4.13 (s, 2H) ppm.

^{13}C NMR ($CDCl_3$, 100MHz): δ = 28.5 (CH3), 40.5 (CH2), 68.1 (CH2), 71.1 (CH2), 172.8 (C) ppm.

Quaternary carbons from Boc group missing

Synthesis of 2,2-dimethyl-4-oxo-3,8,11-trioxa-5-azatridecan-13-oic acid

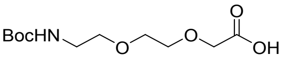

Following general procedure 2 for ester hydrolysis, 1.91 g of the product was obtained in 97% yield.

^{1}H NMR ($CDCl_3$, 400MHz): δ = 1.44 (s, 9H), 3.34 (t, J = 4.7 Hz, 2H), 3.57 (t, J = 5.1 Hz, 2H), 3.63-3.68 (m, 2H), 3.73-3.78 (m, 2H), 4.17 (s, 2H) ppm.

^{13}C NMR ($CDCl_3$, 100MHz): δ = 28.5 (CH3), 40.5 (CH2), 68.8 (CH2), 70.2 (CH2), 70.6 (CH2), 71.4 (CH2), 172.8 (C) ppm.

Quaternary carbons from Boc group missing

Synthesis of 2-(2-(2-(trityloxy)ethoxy)ethoxy)ethan-1-ol

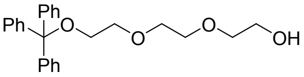

Triethylene glycol (18.0 g, 120 mmol, 6 eq) was dissolved in DCM (100 mL), followed by the addition of Et_3N (5.6 mL, 40 mmol, 2 eq), DMAP (244 mg, 2.0 mmol, 0.1 eq), and Trityl chloride (5.58 g, 20 mmol, 1 eq). After 16h refluxing, brine was added and extracted with DCM twice. Organic layers were dried over Na_2SO_4 and evaporated. Flash column chromatography (PE/EA 10 -> 60%) delivered 4.76 g of the product in 61% yield.

^{1}H NMR ($CDCl_3$, 400MHz): δ = 2.29 (br s, 1H), 3.23-3.28 (m, 2H), 3.60-3.65 (m, 2H), 3.66-3.75 (m, 8H), 7.19-7.25 (m, 3H), 7.27-7.32 (m, 6H), 7.43-7.48 (m, 6H) ppm.

^{13}C NMR ($CDCl_3$, 100MHz): δ = 62.0 (CH2), 63.4 (CH2), 70.7(CH2), 70.9 (CH2), 71.0 (CH2), 72.6 (CH2), 86.8 (C), 127.1 (CH), 127.9 (CH), 128.9 (CH), 144.2 (C) ppm.

Synthesis of *tert*-butyl 4-(2-(2,6-dioxopiperidin-3-yl)-1,3-dioxoisoindolin-4-yl)piperazine-1-carboxylate

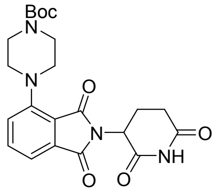

4-fluoro thalidomide (1.0 g, 3.62 mmol, 1 eq) and Boc-piperazine (0.74 g, 3.98 mmol, 1.1 eq) were dissolved in 1,4-dioxane followed by the addition of DIPEA (2.5 mL, 14.5 mmol, 4 eq). After 18h of heating at 100°C, EA was

added and washed twice with brine. The organic layers were dried over Na_2SO_4, filtered and evaporated. Flash column chromatography (DCM/MeOH 0 -> 5%) delivered the product (1.0 g) in 63% yield.

^{1}H NMR ($CDCl_3$, 400MHz): δ = 1.48 (s, 9H), 2.09-2.19 (m, 1H), 2.96-2.95 (m, 3H), 3.30-3.34 (m, 4H), 3.68 (t, J= 4.7 Hz, 4H), 4.97 (dd, J= 5.2; 12.3 Hz, 1H), 7.25 (d, J= 7.3 Hz, 1H), 7.47 (d, J= 7.2 Hz, 1H), 7.63 (t, J= 7.7 Hz, 1H), 8.13 (s, 1H) ppm.

^{13}C NMR ($CDCl_3$, 100MHz): δ = 22.8 (CH2), 28.5 (CH3), 31.5 (CH2), 43.5 (CH2), 49.4 (CH), 51.4 (CH2), 80.4 (C), 117.6 (CH), 124.5 (CH), 134.4 (C), 136.1 (CH), 154.8 (C), 166.6 (C), 167.1 (C), 168.2 (C), 170.8 (C) ppm.

2 quaternary carbons missing.

HRMS: ESI$^+$, *m/z* 443.1926 [M+H]$^+$, Error: 0.21 ppm

Synthesis of *tert*-butyl (2-(2-(2-(4-(2-(2,6-dioxopiperidin-3-yl)-1,3-dioxoisoindolin-4-yl)piperazin-1-yl)-2-oxoethoxy) ethoxy)ethyl)carbamate

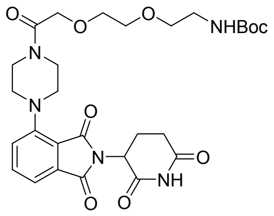

Following the standard HATU coupling procedure 3, 650 mg of the product was delivered in 98% yield after flash column chromatography (DCM/MeOH 0 -> 10%)

^{1}H NMR (DMSO-d6, 400MHz): δ = 1.36 (s, 9H), 1.98-2.04 (m, 1H), 2.52-2.68 (m, 2H), 2.82-2.94 (m, 1H), 3.03-3.10 (m, 2H), 3.28 (s, 4H), 3.39 (t, J= 6.1 Hz, 2H), 3.51-3.60 (m, 4H), 3.62 (s, 4H), 4.20 (s, 2H), 5.11 (dd, J= 5.4; 12.9 Hz, 1H), 6.77 (t, J= 5.4 Hz, 1H), 7.35 (d, J= 8.4 Hz, 1H), 7.41 (d, J= 7.1 Hz, 1H), 7.72 (t, J= 8.0 Hz, 1H), 11.1 (s, 1H) ppm.

^{13}C NMR (DMSO-d6, 100MHz): δ = 22.0 (CH2), 28.2 (CH3), 30.9 (CH2), 39.1 (CH2), 41.1 (CH2), 44.3 (CH2), 48.8 (CH), 50.3 (CH2), 50.7 (CH2), 69.1 (CH2), 69.3 (CH2), 69.5 (CH2), 69.8 (CH2), 77.6 (C), 115.2 (CH), 116.9 (C), 123.9 (CH), 133.6 (C), 135.9 (CH), 149.4 (C), 155.6 (C), 166.3 (C), 167.0 (C), 167.5 (C), 169.9 (C), 172.8 (C) ppm.

HRMS: ESI$^+$, *m/z* 588.2659 [M+H]$^+$, Error: 0.86 ppm

Synthesis of *tert*-butyl (2-(2-(2-(4-(2-(2,6-dioxopiperidin-3-yl)-1,3-dioxoisoindolin-4-yl)piperazin-1-yl)ethoxy)ethoxy) ethyl)carbamate

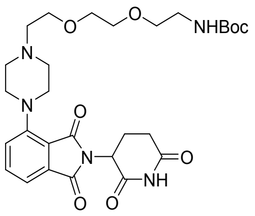

Tert-butyl 4-(2-(2,6-dioxopiperidin-3-yl)-1,3-dioxoisoindolin-4-yl)piperazine-1-carboxylate (470 mg, 1.1 mmol, 1.0 eq) was deprotected by adding TFA/DCM (3/6 mL). The appropriate linker (395 mg, 1.59 mmol, 1.5 eq) was mesylated by dissolving it in DCM and addition of Et_3N (0.44 mL, 3.18 mmol, 3.0 eq) and methanesulfonic anhydride (0.55 g, 3.18 mmol, 3.0 eq). After 1h, water was added and extracted twice with EA. After evaporation, it was redissolved in DMF and Et_3N (0.74 mL, 5.3 mmol, 5 eq) and the deprotected amine were subsequently added and heated for 20h at 80 °C. The reaction mixture was washed twice with brine and organic layers were

dried over Na_2SO_4, filtered and evaporated. Flash column chromatography (DCM/MeOH 0 -> 5%) delivered the product (0.21 g) in 35% yield.

Due to peak broadening, no NMR is reported.

HRMS: ESI$^+$, *m/z* 574.2838 [M+H]$^+$, Error: 5.82 ppm

Synthesis of 2-(2,6-dioxopiperidin-3-yl)-4-(2-(2-(2-(trityloxy)ethoxy)ethoxy)ethoxy)isoindoline-1,3-dione

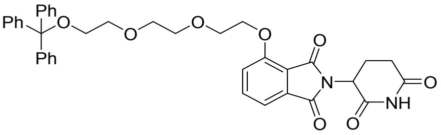

The Linker (1.0eq), 4-hydroxythalidomide (1 g, 3.6 mmol, 1.1eq) and PPh_3 (1.04 g, 3.98 mmol, 1.2 eq) were dissolved in dry THF and cooled down to 0°C. DBAD (0.92 g, 3.98 mmol 1.2 eq) was added and the reaction mixture is stirred for 5h-7h. H_2O was added and extracted with EA. The organic phase was washed 2 times with sat. $NaHCO_3$ to remove excess 4-hydroxythalidomide, followed by drying over Na_2SO_4, filtration and evaporation. The product was obtained after flash column chromatography (PE/EA 30 -> 80%). Since considerable amounts of $OPPh_3$ were present, it was directly used in the next step.

Synthesis of 2-(2,6-dioxopiperidin-3-yl)-4-(2-(2-(2-hydroxyethoxy)ethoxy)ethoxy)isoindoline-1,3-dione

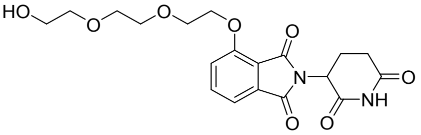

The trityl protected intermediate (3.08 mmol, 1 eq) was dissolved in MeOH and camphorsulfonic acid was added (72 mg, 0.31 mmol, 0.1 eq). The reaction was quenched by addition of Et_3N (0.1 eq) and evaporated on celite. Flash column chromatography delivered 1 g of the product in 76% over 2 steps.

^{1}H NMR ($CDCl_3$, 400MHz): δ = 2.08-2.16 (m, 2H), 2.67-2.92 (m 3H), 3.60-3.63 (m, 2H), 3.66-3.74 (m, 4H), 3.79-3.85 (m, 2H), 3.94-4.00 (m, 2H), 4.34-4.38 (m, 2H), 4.73 (br s, 1H), 4.95 (dd, J= 5.3; 12.2 Hz, 1H), 7.25 (d, J= 8.4 Hz, 1H), 7.47 (d, J= 7.0Hz, 1H), 7.67 (dd, J= 7.4; 8.4 Hz, 1H), 8.23 (s, 1H) ppm.

^{13}C NMR ($CDCl_3$, 100MHz): δ = 22.8 (CH2), 31.5 (CH2), 49.3 (CH), 61.9 (CH2), 69.4 (2x CH2), 70.5 (CH2), 71.2 (CH2), 72.6 (CH2), 116.3 (CH), 117.4 (C), 119.5 (CH), 133.9 (C), 136.6 (CH), 156.5 (C), 165.8 (C), 167.1 (C), 168.2 (C), 171.1 (C) ppm.

Synthesis of *tert*-butyl 4-(2-(2-(2-((2-(2,6-dioxopiperidin-3-yl)-1,3-dioxoisoindolin-4-yl)oxy)ethoxy)ethoxy)ethyl) piperazine-1-carboxylate

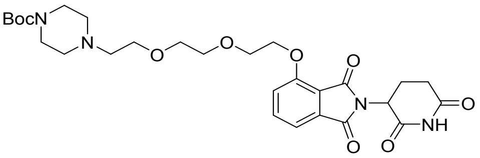

2-(2,6-dioxopiperidin-3-yl)-4-(2-(2-(2-hydroxyethoxy)ethoxy)ethoxy)isoindoline-1,3-dione (500 mg, 1.23 mmol, 1 eq) was mesylated by dissolving it in DCM and addition of Et_3N (0.34 mL, 2.46 mmol, 2.0 eq) and methanesulfonic anhydride (0.32 g, 1.85 mmol, 1.5 eq). After 1h, water was added and extracted twice with EA. After evaporation, it was redissolved in DMF and Et_3N (0.34 mL, 2.46 mmol, 2 eq) and the Boc-piperazine (0.46 g, 2.46 mmol, 2 eq) were subsequently added and heated for 20h at 80 °C. DCM was added, the reaction mixture was washed twice

with brine and organic layers were dried over Na_2SO_4, filtered and evaporated. Flash column chromatography (DCM/MeOH 0->10% +0.1% NH_4OH) delivered 202 mg of the product in 29% yield.

Due to peak broadening, no NMR is reported.

HRMS: ESI$^+$, *m/z* 575.2702 [M+H]$^+$, Error: 1.66 ppm

Synthesis of *tert*-butyl 4-(2-(2,6-dioxopiperidin-3-yl)-1,3-dioxoisoindolin-5-yl)piperazine-1-carboxylate (S1.17)

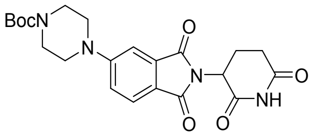

5-fluoro thalidomide (1.94 g, 7.0 mmol, 1 eq) and Boc-piperazine (1.56 g, 8.4 mmol, 1.2 eq) were dissolved in DMSO followed by the addition of DIPEA (2.4 mL, 14 mmol, 2 eq). After 18h of heating at 80 °C, the reaction mixture was allowed to cool to rt and EA was added and washed three times with brine. The organic layers were dried over Na_2SO_4, filtered and evaporated. Flash column chromatography (DCM/MeOH 0 -> 5%) delivered the product (2.29 g) in 74% yield.

^{1}H NMR ($CDCl_3$, 400MHz): δ = 1.48 (s, 9H), 2.09-2.19 (m, 1H), 2.72-2.95 (m, 3H), 3.40-3.44 (m, 4H), 3.61-3.66 (m, 4H), 4.94 (dd, J= 5.2; 12.3 Hz, 1H), 7.13 (dd, J= 2.3, 8.5 Hz, 1H), 7.32 (d, J= 2.2 Hz, 1H), 7.73 (t, J= 8.5 Hz, 1H), 8.12 (s, 1H) ppm.

^{13}C NMR ($CDCl_3$, 100MHz): δ = 22.8 (CH2), 28.5 (CH3), 31.5 (CH2), 42.9 (CH2), 47.9 (CH2), 49.4 (CH), 80.6 (C), 109.4 (CH), 118.9 (CH), 120.8 (C), 126.7 (CH), 134.4 (C), 154.6 (C), 155.0 (C), 167.2 (C), 167.7 (C), 168.3 (C), 171.1 (C) ppm.

HRMS: ESI$^+$, *m/z* 443.1926 [M+H]$^+$, Error: 0.20 ppm

Synthesis of *tert*-butyl (2-(4-(2-(2,6-dioxopiperidin-3-yl)-1,3-dioxoisoindolin-5-yl)piperazin-1-yl)ethyl)carbamate (S1.18a)

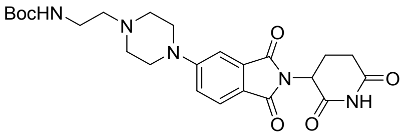

Following the general procedure 4, the product was obtained (190 mg) with a 58% yield. In stead of a mesylated linker, commercially available *tert*-butyl (2-bromoethyl)carbamate was used.

^{1}H NMR (DMSO-d6, 400MHz): δ = 1.38 (s, 9H), 1.96-2.04 (m, 1H), 2.37 (t, J= 6.8Hz, 2H), 2.50-2.60 (m, 6H), 2.83-2.94 (m, 1H), 3.04-3.06 (m, 2H), 3.40-3.44 (m, 4H), 3.61-3.66 (m, 4H), 5.07 (dd, J= 5.2; 12.3 Hz, 1H), 6.69 (t, J= 5.5 Hz, 1H), 7.25 (dd, J= 2.1, 8.5 Hz, 1H), 7.34 (d, J= 2.0 Hz, 1H), 7.67 (d, J= 8.5 Hz, 1H), 11.07 (s, 1H) ppm.

^{13}C NMR (DMSO-d6, 100MHz): δ = 22.2 (CH2), 28.3 (CH3), 30.9 (CH2), 37.3 (CH2), 46.8 (CH2), 47.8 (CH), 52.2 (CH2), 57.2 (CH2), 77.5 (C), 107.9 (CH), 117.8 (CH), 118.3 (C), 124.9 (CH), 133.8 (C), 155.2 (C), 155.6 (C), 167.0 (C), 167.5 (C), 170.1 (C), 172.8 (C) ppm.

HRMS: ESI$^+$, *m/z* 486.2349 [M+H]$^+$, Error: 0.39 ppm

Synthesis of *tert*-butyl (2-(2-(4-(2-(2,6-dioxopiperidin-3-yl)-1,3-dioxoisoindolin-5-yl)piperazin-1-yl)ethoxy)ethyl) carbamate (S1.18b)

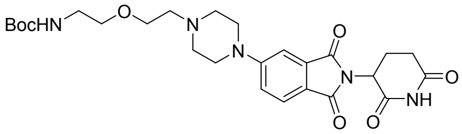

Following the general procedure 4, the product was obtained (140 mg) with a 29% yield.

^{1}H NMR (DMSO-d6, 400MHz): δ = 1.36 (s, 9H), 1.96-2.04 (m, 1H), 2.50-2.60 (m, 8H), 2.83-2.94 (m, 1H), 3.04-3.06 (m, 2H), 3.39 (t, J= 6.0 Hz, 2H), 3.43 (s, 4H), 3.53 (t, J= 5.7 Hz, 2H), 5.07 (dd, J= 5.2; 12.3 Hz, 1H), 6.78 (t, J= 5.5 Hz, 1H), 7.25 (dd, J= 2.1, 8.5 Hz, 1H), 7.33 (d, J= 2.0 Hz, 1H), 7.67 (d, J= 8.5 Hz, 1H), 11.07 (s, 1H) ppm.

^{13}C NMR (DMSO-d6, 100MHz): δ = 22.2 (CH2), 28.3 (CH3), 30.9 (CH2), 38.9 (CH2), 46.8 (CH2), 48.8 (CH), 52.2 (CH2), 57.0 (CH2), 67.8 (CH2), 68.9 (CH2), 77.5 (C), 107.9 (CH), 117.8 (CH), 118.3 (C), 124.9 (CH), 133.8 (C), 155.2 (C), 155.6 (C), 167.0 (C), 167.5 (C), 170.1 (C), 172.8 (C) ppm.

HRMS: ESI$^+$, *m/z* 530.2602 [M+H]$^+$, Error: 1.38 ppm

Synthesis of *tert*-butyl (2-(2-(2-(4-(2-(2,6-dioxopiperidin-3-yl)-1,3-dioxoisoindolin-5-yl)piperazin-1-yl)ethoxy)ethoxy) ethyl)carbamate (S1.18c)

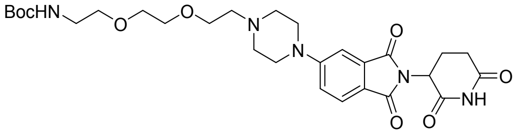

Following the general procedure 4, the product was obtained (90 mg) in 17% yield.

^{1}H NMR (DMSO-d6, 400MHz): δ = 1.36 (s, 9H), 1.96-2.04 (m, 1H), 2.50-2.60 (m, 8H), 2.83-2.94 (m, 1H), 3.04-3.06 (m, 2H), 3.39 (t, J= 6.0 Hz, 2H), 3.43 (m, 4H), 3.49-3.53 (m, 4H), 3.55 (t, J= 5.7 Hz, 2H), 5.07 (dd, J= 5.2; 12.3 Hz, 1H), 6.75 (t, J= 5.5 Hz, 1H), 7.25 (dd, J= 2.1, 8.5 Hz, 1H), 7.33 (d, J= 2.0 Hz, 1H), 7.67 (d, J= 8.5 Hz, 1H), 11.07 (s, 1H) ppm.

^{13}C NMR (DMSO-d6, 100MHz): δ = 22.2 (CH2), 28.3 (CH3), 30.9 (CH2), 38.9 (CH2), 46.8 (CH2), 48.8 (CH), 52.7 (CH2), 57.0 (CH2), 68.4 (CH2), 69.2 (CH2), 69.5 (CH2), 69.6 (CH2), 77.5 (C), 107.9 (CH), 117.8 (CH), 118.3 (C), 124.9 (CH), 133.8 (C), 155.2 (C), 155.6 (C), 167.0 (C), 167.5 (C), 170.1 (C), 172.8 (C) ppm.

HRMS: ESI$^+$, *m/z* 574.2872 [M+H]$^+$, Error: 0.10 ppm

Synthesis of *tert*-butyl (2-(4-(2-(2,6-dioxopiperidin-3-yl)-1,3-dioxoisoindolin-5-yl)piperazin-1-yl)-2-oxoethyl)carbamate (S1.18d)

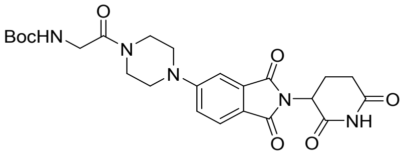

Following the general amide coupling procedure 3, the product was obtained (290 mg) in 86% yield.

^{1}H NMR ($CDCl_3$, 400MHz): δ = 1.45 (s, 9H), 2.10-2.17 (m, 1H), 2.67-2.95 (m, 3H), 3.43-3.49 (m, 4H), 3.61-3.65 (m, 2H), 3.82-3.86 (m, 2H), 4.01 (s, 2H), 4.95 (dd, J= 5.3; 12.3 Hz, 1H), 7.11 (dd, J= 2.2, 8.5 Hz, 1H), 7.30 (d, J= 2.2 Hz, 1H), 7.74 (d, J= 8.4 Hz, 1H), 8.16 (s, 1H) ppm.

^{13}C NMR ($CDCl_3$, 100MHz): δ = 22.7 (CH2), 28.5 (CH3), 31.6 (CH2), 41.4 (CH2), 42.0 (CH2), 43.8 (CH2), 47.6 (CH2), 49.9 (CH), 80.2 (C), 109.3 (CH), 118.8 (CH), 121.0 (C), 125.6 (CH), 134.4 (C), 154.9 (C), 156.0 (C), 167.2 (C), 167.5 (C), 167.7 (C), 168.4 (C), 171.1 (C) ppm.

HRMS: ESI$^+$, *m/z* 500.2133 [M+H]$^+$, Error: 1.36 ppm

Synthesis of *tert*-butyl (2-(2-(4-(2-(2,6-dioxopiperidin-3-yl)-1,3-dioxoisoindolin-5-yl)piperazin-1-yl)-2-oxoethoxy) ethyl)carbamate (S1.18e)

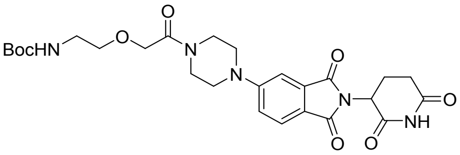

Following the general amide coupling procedure 3, the product was obtained (200 mg) in 54% yield.

^1H NMR ($CDCl_3$, 400MHz): δ = 1.43 (s, 9H), 2.10-2.17 (m, 1H), 2.67-2.95 (m, 3H), 3.35 (t, J= 5.2 Hz, 2H), 3.46 (s, 4H), 3.61 (t, J= 5.2 Hz, 2H), 3.70 (s, 2H), 3.82 (s, 2H), 4.22 (s, 2H), 4.95 (dd, J= 5.3; 12.3 Hz, 1H), 7.14 (dd, J= 1.7; 8.5 Hz, 1H), 7.33 (d, J= 1.7 Hz, 1H), 7.73 (d, J= 8.4 Hz, 1H), 8.21 (s, 1H) ppm.

^{13}C NMR ($CDCl_3$, 100MHz): δ = 22.7 (CH2), 28.5 (CH3), 31.6 (CH2), 40.7 (CH2), 41.2 (CH2), 44.2 (CH2), 47.8 (CH2), 48.1 (CH2), 49.4 (CH), 70.2 (CH2), 70.8 (CH2), 79.7 (C), 109.3 (CH), 119.0 (CH), 121.2 (C), 125.6 (CH), 134.4 (C), 154.9 (C), 156.1 (C), 167.1 (C), 167.7 (C), 168.0 (C), 168.3 (C), 171.1 (C) ppm.

HRMS: ESI$^+$, *m/z* 544.2402 [M+H]$^+$, Error: 0.02 ppm

Synthesis of *tert*-butyl (2-(2-(2-(4-(2-(2,6-dioxopiperidin-3-yl)-1,3-dioxoisoindolin-5-yl)piperazin-1-yl)-2-oxoethoxy) ethoxy)ethyl)carbamate (S1.18f)

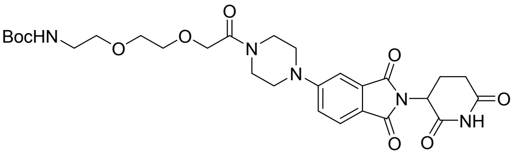

Following the general amide coupling procedure 3, the product was obtained (280 mg) in 70% yield.

^1H NMR ($CDCl_3$, 400MHz): δ = 1.43 (s, 9H), 2.10-2.17 (m, 1H), 2.67-2.95 (m, 3H), 3.31 (t, J= 5.2 Hz, 2H), 3.47 (s, 4H), 3.54 (t, J= 5.2 Hz, 2H), 3.64-3.73 (m, 4H), 3.79 (s, 2H), 3.83 (s, 2H), 4.27 (s, 2H), 4.95 (dd, J= 5.3; 12.3 Hz, 1H), 7.14 (d, J=8.5 Hz, 1H), 7.33 (s, 1H), 7.74 (d, J= 8.4 Hz, 1H), 8.16 (s, 1H) ppm.

^{13}C NMR ($CDCl_3$, 100MHz): δ = 22.7 (CH2), 28.5 (CH3), 31.6 (CH2), 40.7 (CH2), 41.2 (CH2), 44.2 (CH2), 48.2 (CH2), 48.5 (CH2), 49.4 (CH), 70.3 (CH2), 70.4 (CH2), 70.8 (CH2), 70.9 (CH2), 79.6 (C), 109.7 (CH), 119.4 (CH), 121.5 (C), 125.6 (CH), 134.4 (C), 154.6 (C), 156.1 (C), 167.1 (C), 167.7 (C), 168.0 (C), 168.3 (C), 171.0 (C) ppm.

HRMS: ESI$^+$, *m/z* 588.2664 [M+H]$^+$, Error: 0.2 ppm

Synthesis of *tert*-butyl (1-(4-methoxybenzyl)-2,6-dioxopiperidin-3-yl)carbamate (S1.20)

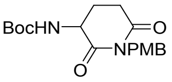

3-Boc-amino-2,6-dioxopiperidine (4.0 g, 17.5 mmol, 1.1 eq), 4-methoxybenzyl alcohol (1.95 mL, 15.9 mmol, 1 eq) and PPh_3 (6.3 g, 23.9 mmol, 1.5 eq) were dissolved in dry THF, followed by addition of DBAD (5.5 g, 23.9 mmol, 1.5 eq). After 6h, water was added and extracted twice with EA. Flash column chromatography (PE/EA 5 -> 50%) delivered the product (3.7 g) in 67% yield.

^{1}H NMR ($CDCl_3$, 400MHz): δ = 1.45 (s, 1H), 1.77 (qd, J= 4.6; 13.1 Hz, 1H), 2.44-2.49 (m, 1H), 2.64-2.76 (m, 1H), 2.87 (ddd, J= 2.1; 4.6; 18.0 Hz, 1H), 3.77 (s, 3H), 4.23-4.28 (m, 1H), 4.85 (d, J= 13.4 Hz, 1H), 4.90 (d, J= 13.7 Hz, 1H), 5.41 (br s, 1H, NH), 6.81 (d, J= 8.7 Hz, 2H), 7.30 (d, J= 8.6 Hz, 2H) ppm.

^{13}C NMR ($CDCl_3$, 100MHz): δ = 25.0 (CH2), 28.4 (CH3), 32.0 (CH2), 43.3 (CH2), 52.8 (CH), 55.4 (CH3), 80.5 (C), 113.9 (CH), 128.8 (C), 130.6 (CH), 155.6 (C), 159.2 (C), 171.3 (C), 172.0 (C) ppm.

Synthesis of 3-amino-1-(4-methoxybenzyl)piperidine-2,6-dione (S1.21)

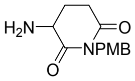

Tert-butyl (1-(4-methoxybenzyl)-2,6-dioxopiperidin-3-yl)carbamate (3.7 g, 10.6 mmol), was dissolved in 4M HCl in dioxane (10 mL) and stirred for 16h. The solids were filtered, washed once with dioxane and delivered the product (2.8 g) as HCl salt in 93% yield after drying *in vacuo*.

^{1}H NMR (DMSO-d6, 400MHz): δ = 2.00-2.13 (m, 1H), 2.25-2.32 (m, 1H), 2.75-2.95 (m, 2H), 3.71 (s, 3H), 4.34-4.40 (m, 1H), 6.85 (d, J= 8.7 Hz, 2H), 7.20 (d, J= 8.6 Hz, 2H), 8.83 (s, 3H) ppm.

^{13}C NMR (DMSO-d6, 100MHz): δ = 21.2 (CH2), 30.2 (CH2), 42.1 (CH2), 49.6 (CH), 55.1 (CH3), 113.7 (CH), 128.7 (C), 129.2 (CH), 158.5 (C), 169.8 (C), 171.1 (C) ppm.

Synthesis of 5-hydroxy-2-(1-(4-methoxybenzyl)-2,6-dioxopiperidin-3-yl)isoindoline-1,3-dione (S1.22)

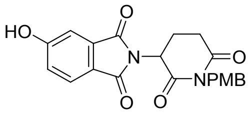

4-hydroxyphtalic acid (544 mg, 2.97 mmol, 1.0 eq) and 3-amino-1-(4-methoxybenzyl)piperidine-2,6-dione (850 mg, 2.97 mmol, 1.0 eq) were dissolved in pyridine (7 ml) and subsequently heated to 140 °C. After 16h, DCM was added and the organic layer was washed twice with water. The organic layers were dried over Na_2SO_4, filtered and evaporated. Flash column chromatography (DCM/MeOH 0 -> 4%) delivered the product (780 mg) in 66% yield

^{1}H NMR (DMSO-d6, 400MHz): δ = 2.02-2.13 (m, 1H), 2.52-2.63 (m, 1H), 2.74-2.81 (m, 1H), 2.99-3.10 (m, 1H), 3.72 (s, 3H), 4.70 (d, J= 14.4 Hz, 1H), 4.84 (d, J= 14.4 Hz, 1H), 5.26 (dd, J= 5.4; 13.1 Hz, 1H), 6.86 (d, J= 8.7 Hz, 2H), 7.15-7.21 (m, 4H), 7.76 (d, J= 8.4 Hz, 1H), 11.1 (s, 1H) ppm.

^{13}C NMR (DMSO-d6, 100MHz): δ = 21.3 (CH2), 31.2 (CH2), 42.2 (CH2), 49.5 (CH), 55.0 (CH3), 110.0 (CH), 113.7 (CH), 120.8 (CH), 121.4 (C), 125.7 (CH), 128.9 (CH), 129.0 (C), 134.1 (C), 158.3 (C), 163.6 (C), 166.9 (C), 167.0 (C), 169.7 (C), 171.6 (C) ppm.

Synthesis of *tert*-butyl 4-(2-hydroxyethyl)piperazine-1-carboxylate (S1.26)

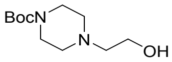

DMF was added to a flask with 2-bromoethan-1-ol (4 g, 32 mmol, 1.0 eq), Boc-piperazine (6 g, 32mmol, 1 eq), and K_2CO_3 (6.6 g, 48 mmol, 1.5 eq) and stirred for 2 days at 80°C. Water was added and extracted twice with DCM. Organic layers were dried over Na_2SO_4 and evaporated. Flash column chromatography (DCM/MeOH 0 -> 10 % + 0.1% NH_4OH) delivered 3.6 g of the product in 49% yield.

^{1}H NMR ($CDCl_3$, 400MHz): δ = 1.44 (s, 9H), 2.52-2.56 (m, 4H), 2.60-2.64 (m, 2H), 3.47-3.51 (m, 4H), 3.65-3.68 (m, 2H) ppm.

^{13}C NMR ($CDCl_3$, 100MHz): δ = 28.5 (CH3), 43.1 (CH2), 43.8 (CH2), 52.9 (CH2), 57.7 (CH2), 59.7 (CH2), 80.0 (C), 154.7 (C) ppm.

HRMS: ESI$^+$, *m/z* 231.1712 [M+H]$^+$, Error: 3.81 ppm

Synthesis of *tert*-butyl 4-(2-(2-hydroxyethoxy)ethyl)piperazine-1-carboxylate (S1.28)

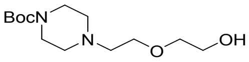

DMF was added to a flask with 2-(2-chloroethoxy)ethan-1-ol (3 g, 24.1 mmol, 1.0 eq), Boc-piperazine (4.5 g, 24.1 mmol, 1 eq), K_2CO_3 (5.0 g, 36.1 mmol, 1.5 eq) and NaI (0.36 g, 2.4 mmol, 0.1 eq) and stirred for 2 days at 80°C. Water was added and extracted twice with DCM. Organic layers were dried over Na_2SO_4 and evaporated. Flash column chromatography (DCM/MeOH 0 -> 10 % + 0.1% NH_4OH) delivered 3.6 g of the product in 55% yield.

^{1}H NMR ($CDCl_3$, 400MHz): δ = 1.44 (s, 9H), 2.49-2.54 (m, 4H), 2.63 (t, J= 5.7 Hz, 2H), 3.41-3.45 (m, 4H), 3.58-3.62 (m, 2H), 3.65-3.71 (m, 4H) ppm.

^{13}C NMR ($CDCl_3$, 100MHz): δ = 28.5 (CH3), 42.6 (CH2), 43.7 (CH2), 53.2 (CH2), 58.0 (CH2), 61.8 (CH2), 67.5 (CH2), 72.6 (CH2), 79.9 (C), 154.9 (C) ppm.

HRMS: ESI$^+$, *m/z* 275.1982 [M+H]$^+$, Error: 6.03 ppm

Synthesis of *tert*-butyl 4-(2-((2-(2,6-dioxopiperidin-3-yl)-1,3-dioxoisoindolin-5-yl)oxy)ethyl)piperazine-1-carboxylate (S1.24a)

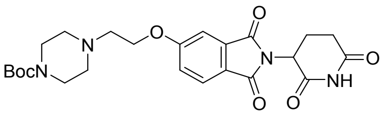

Linker **S1.26** (288 mg, 1.25 mmol, 1.1 eq), **S1.22** (450 mg, 1.14 mmol, 1 eq) and PPh_3 (359 mg, 1.37 mmol, 1.2 eq) were dissolved in dry THF and cooled down to 0 °C. DBAD (315 mg, 1.37 mmol, 1.2 eq) was added and the reaction mixture was stirred for 5h at RT. H_2O was added and extracted with EA. The organic phase was washed 2 times with sat. $NaHCO_3$, followed by drying over Na_2SO_4, filtration and evaporation. Flash column chromatography (DCM/MeOH 0 -> 5/10%) delivered the PMB-protected intermediate (630 mg) in 91 % yield. The intermediate (530 mg, 0.87 mmol, 1.0 eq) was redissolved in MeCN/Water (9 / 3 mL) and Cerium Ammonium Nitrate (CAN) added (1.4 g, 2.61 mmol, 3 eq). After 3h, brine was added and extracted with EA twice. Purification using flash column chromatography (DCM/MeOH 0 -> 5% + 0.1% NH_4OH) delivered the product (210 mg) in 50% yield.

^{1}H NMR ($CDCl_3$, 400MHz): δ = 1.46 (s, 9H), 2.11-2.19 (m, 1H), 2.61 (s, 4H), 2.68-2.97 (m 5H), 3.51 (s, 4H), 4.28 (s, 2H), 4.96 (dd, J= 5.3; 12.3 Hz, 1H), 7.21 (dd, J= 2.3; 8.3 Hz, 1H), 7.36 (d, J= 2.2 Hz, 1H), 7.78 (d, J= 8.3 Hz, 1H), 8.42 (s, 1H) ppm.

^{13}C NMR ($CDCl_3$, 100MHz): δ = 22.8 (CH2), 28.6 (CH3), 31.6 (CH2), 43.2 (CH2, via HSQC), 49.5 (CH), 53.5 (CH2), 56.8 (CH2), 66.4 (CH2), 80.3 (C), 109.3 (CH), 120.8 (CH), 124.1 (C), 125.8 (CH), 134.5 (C), 154.6 (C), 163.8 (C), 167.0 (C), 167.1 (C), 168.2 (C), 171.1 (C) ppm.

HRMS: ESI$^+$, *m/z* 487.2179 [M+H]$^+$, Error: 1.70 ppm

Synthesis of *tert*-butyl 4-(2-(2-((2-(2,6-dioxopiperidin-3-yl)-1,3-dioxoisoindolin-5-yl)oxy)ethoxy)ethyl)piperazine-1-carboxylate (S1.24b)

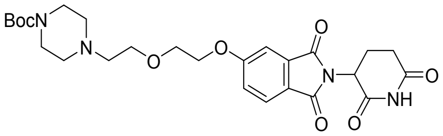

Following the same procedure as described for **S1.24a**, delivered the PMB-protected intermediate (330 mg) in 45% yield. The intermediate (330 mg, 0.51 mmol, 1.0 eq) was redissolved in MeCN/Water (6 / 2 mL) and Cerium Ammonium Nitrate (CAN) added (833 mg, 1.52 mmol, 3 eq). After 3h, brine was added and extracted with EA twice. Purification using flash column chromatography (DCM/MeOH 0 -> 5% + 0.1% NH_4OH) delivered the product (130 mg) in 48% yield.

^{1}H NMR ($CDCl_3$, 400MHz): δ = 1.46 (s, 9H), 2.11-2.19 (m, 1H), 2.45 (t, J= 4.7 Hz, 4H), 2.62 (t, J= 5.7 Hz, 2H), 2.68-2.93 (m 3H), 3.43 (t, J= 4.9 Hz, 4H), 3.69 (t, J= 5.7 Hz, 2H), 3.83-3.88 (m, 2H), 4.22-4.26 (m, 2H), 4.96 (dd, J= 5.3; 12.3 Hz, 1H), 7.21 (dd, J= 2.3; 8.3 Hz, 1H), 7.36 (d, J= 2.2 Hz, 1H), 7.77 (d, J= 8.3 Hz, 1H), 8.31 (s, 1H) ppm.

^{13}C NMR ($CDCl_3$, 100MHz): δ = 22.8 (CH2), 28.6 (CH3), 31.6 (CH2), 43.4 (CH2, via HSQC), 49.5 (CH), 53.5 (CH2), 57.9 (CH2), 68.6 (CH2), 69.3 (CH2), 69.3 (CH2), 79.8 (C), 109.1 (CH), 121.1 (CH), 123.8(C), 125.7 (CH), 134.4 (C), 154.9 (C), 164.4 (C), 167.1 (C), 167.2 (C), 168.2 (C), 171.0 (C) ppm.

HRMS: ESI$^+$, *m/z* 531.2445 [M+H]$^+$, Error: 0.82 ppm

PROTAC synthesis

Synthesis of PROTAC 1

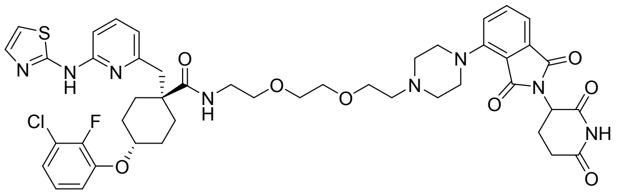

Following the general PROTAC synthesis procedure, 63 mg of the product was obtained with a yield of 69%.

^{1}H NMR (DMSO-d6, 400MHz): δ = 1.58- 1.93 (m, 8H), 1.97- 2.05 (m, 1H), 2.50 – 2.60 (m, 8H, overlap with solvent signal), 2.80-2.93 (m, 3H), 3.16-3.30 (m, 6H), 3.38 (t, J= 6.0Hz, 2H), 3.46-3.57 (m, 6H), 4.54 (m, 1H), 5.09 (dd, J= 5.4; 12.9 Hz, 1H), 6.64 (d, J= 7.3Hz, 1H), 6.86 (d, J= 8.2Hz, 1H), 6.95 (d, J= 3.6Hz, 1H), 7.06-7.18 (m, 3H), 7.26-7.37 (m, 3H), 7.54 (t, J= 7.8 Hz, 1H), 7.62 (t, J= 5.5Hz, 1H), 7.67 (t, J= 8.0, 1H), 11.08 (s, 1H), 11.11 (s, 1H) ppm.

^{13}C NMR (DMSO-d6, 100MHz): δ = 22.1 (CH2), 26.6 (CH2), 28.4 (CH2), 31.0 (CH2), 38.8 (CH2), 46.6 (C), 46.8 (CH2), 48.8 (CH), 50.5 (CH2), 53.0 (CH2), 57.2 (CH2), 68.3 (CH2), 68.9 (CH2), 69.5 (CH2), 69.7 (CH2), 74.4 (CH), 108.1 (CH), 110.7 (CH), 114.8 (CH), 116.2 (CH), 116.5 (C), 116.7 (CH), 120.4 (d, $^{2}J(C,F)$ = 15.1Hz, C), 121.9 (CH), 123.7 (CH), 124.9 (d, $^{3}J(C,F)$= 4.9Hz, CH), 133.7 (C), 135.8 (CH), 137.4 (CH), 137.6 (CH), 146.2 (d, $^{2}J(C,F)$= 10.1Hz, C), 148.9 (d, $^{1}J(C,F)$= 245.7Hz, C), 149.7 (C), 150.7 (C), 155.2 (C), 159.8 (C), 166.3 (C), 167.0 (C), 170.0 (C), 172.8 (C), 174.1 (C) ppm.

^{19}F NMR (DMSO-d6, 377MHz): δ = -136.2 ppm

HRMS: ESI$^+$, *m/z* 917.3205 [M+H]$^+$, Error: 1.38 ppm

Synthesis of PROTAC 2

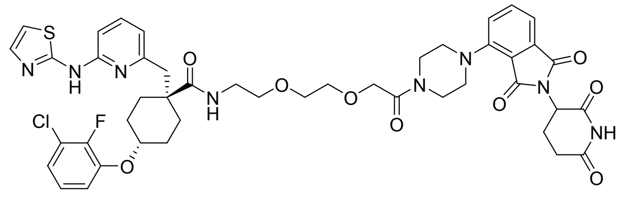

Following the general PROTAC synthesis procedure, 58 mg of the product was obtained with a yield of 62%.

^{1}H NMR (DMSO-d6, 400MHz): δ = 1.58- 1.93 (m, 8H), 1.97- 2.05 (m, 1H), 2.50 – 2.60 (m, 2H, overlap with solvent signal), 2.80-2.93 (m, 3H), 3.16-3.30 (m, 6H), 3.39 (t, J= 6.0Hz, 2H), 3.49-3.65 (m, 8H), 4.20 (s, 2H), 4.54 (m, 1H), 5.11 (dd, J= 5.4; 12.9 Hz, 1H), 6.63 (d, J= 7.3Hz, 1H), 6.86 (d, J= 8.2Hz, 1H), 6.95 (d, J= 3.6Hz, 1H), 7.06-7.18 (m, 3H), 7.32 (d, J= 8.4Hz, 1H), 7.35 (d, J= 3.6Hz, 1H), 7.38 (d, J= 7.1Hz, 1H), 7.54 (t, J= 7.9Hz, 1H), 7.62 (t, J= 5.5Hz, 1H), 7.70 (dd, J= 7.4; 8.1Hz, 1H), 11.09 (s, 1H), 11.11 (s, 1H) ppm.

^{13}C NMR (DMSO-d6, 100MHz): δ = 22.0 (CH2), 26.6 (CH2), 28.4 (CH2), 31.0 (CH2), 38.8 (CH2), 41.1 (CH2), 44.3 (CH2), 46.6 (C), 46.8 (CH2), 48.8 (CH), 50.3 (CH2), 50.7 (CH2), 68.8 (CH2), 69.3 (CH2), 69.4 (CH2), 69.8 (CH2), 74.3 (CH), 108.1 (CH), 110.7 (CH), 115.3 (CH), 116.2 (CH), 116.7 (CH), 116.9 (C), 120.41 (d, ^{2}J(C,F) = 15.1Hz, C), 121.8 (CH), 123.8 (CH), 124.9 (d, ^{3}J(C,F)= 4.9Hz, CH), 133.6 (C), 135.9 (CH), 137.4 (CH), 137.6 (CH), 146.2 (d, ^{2}J(C,F)= 10.1Hz, C), 148.9 (d, ^{1}J(C,F)= 245.7Hz, C), 149.4 (C), 150.7 (C), 155.2 (C), 159.8 (C), 166.4 (C), 166.7 (C), 167.5 (C), 167.0 (C), 172.8 (C), 174.1 (C) ppm.

^{19}F NMR (DMSO-d6, 377MHz): δ = -136.3 ppm

HRMS: ESI^{+}, *m/z* 931.3003 [M+H]$^{+}$, Error: 0.78 ppm

Synthesis of PROTAC 3

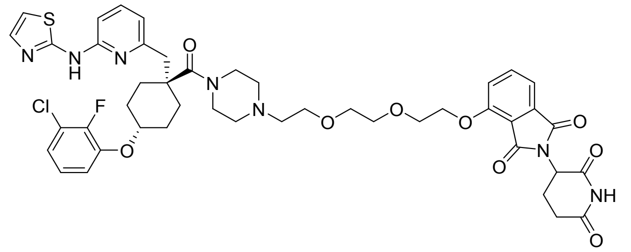

Following the general PROTAC synthesis procedure, 23 mg of the product was obtained with a yield of 25%.

^{1}H NMR (DMSO-d6, 400MHz): δ = 1.63- 1.83 (m, 6H), 1.87- 2.05 (m, 3H), 2.40 (s, 4H), 2.43-2.50 (m, 2H, overlap with solvent signal), 2.50 – 2.60 (m, 2H, overlap with solvent signal), 2.80-2.93 (m, 1H), 3.03 (s, 2H), 3.48-3.69 (m, 10H), 3.81 (m, 2H), 4.33 (m, 2H), 4.54 (m, 1H), 5.08 (dd, J= 5.4; 12.9 Hz, 1H), 6.66 (d, J= 7.3Hz, 1H), 6.89 (d, J= 8.2Hz, 1H), 6.97 (d, J= 3.6Hz, 1H), 7.05-7.15 (m, 3H), 7.35 (d, J= 3.6Hz, 1H), 7.44 (d, J= 7.2 Hz, 1H), 7.52 (d, J= 8.6 Hz, 1H), 7.56 (t, J= 7.9 Hz, 1H), 7.79 (dd, J= 7.4; 8.4 Hz, 1H), 11.10 (s, 1H), 11.15 (s, 1H) ppm.

^{13}C NMR (DMSO-d6, 100MHz): δ = 22.0 (CH2), 26.9 (CH2), 29.5 (CH2), 30.9 (CH2), 44.8 (CH2), 47.1 (C), 48.7 (CH), 53.3 (CH2), 57.0 (CH2), 68.2 (CH2), 68.7 (CH2), 68.9 (CH2), 69.7 (CH2), 70.1 (CH2), 73.8 (CH), 108.3 (CH), 110.7 (CH), 115.4 (CH), 116.2 (CH), 116.3 (C), 116.5 (CH), 120.0 (CH), 120.4 (d, ^{2}J(C,F) = 15.1Hz, C), 121.8 (CH), 124.9 (d, ^{3}J(C,F)= 4.9Hz, CH), 133.2 (C), 137.0 (CH), 137.4 (CH), 137.7 (CH), 146.1 (d, ^{2}J(C,F)= 10.1Hz, C), 148.9 (d, ^{1}J(C,F)= 245.7Hz, C), 150.9 (C), 154.9 (C), 155.8 (C), 159.6 (C), 165.3 (C), 166.8 (C), 169.9 (C), 171.8 (C), 172.8 (C) ppm.

One CH2 from piperazine missing.

<u>^{19}F NMR (DMSO-d6, 377MHz):</u> δ = -136.4 ppm

<u>HRMS</u>: ESI^{+}, *m/z* 918.3056 [M+H]$^{+}$, Error: 0.19 ppm

Synthesis of PROTAC 4

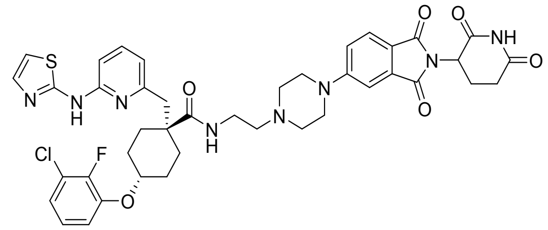

Following the general PROTAC synthesis procedure, 40 mg of the product was obtained with a yield of 49%.

<u>^{1}H NMR (DMSO-d6, 400MHz):</u> δ = 1.62-1.95 (m, 8H), 1.96-2.07 (m, 1H), 2.36 (t, J = 6.3 Hz, 2H), 2.46- 2.69 (m, 6H, overlap with solvent peak), 2.80-2.97 (m, 3H), 3.14-3.25 (m, 2H), 3.39 (s, 4H), 4.56 (s, 1H), 5.07 (dd, J = 5.3; 12.7 Hz, 1H), 6.65 (d, J = 7.3 Hz, 1H), 6.87 (d, J = 8.2 Hz, 1H), 6.95 (d, J = 3.6Hz, 1H), 7.06-7.18 (m, 3H), 7.24 (dd, J = 1.5; 8.5 Hz, 1H), 7.31-7.38 (m, 2H), 7.46 (t, J = 5.0 Hz, 1H), 7.55 (t, J = 7.8 Hz, 1H), 7.67 (d, J = 8.5 Hz, 1H), 11.07 (s, 1H), 11.13 (s, 1H) ppm

<u>^{13}C NMR (DMSO-d6, 100MHz)</u>: δ = 22.2 (CH2), 26.6 (CH2), 28.4 (CH2), 31.0 (CH2), 36.2 (CH2), 46.6 (C), 47.0 (2 x CH2), 48.8 (CH), 52.1 (CH2), 56.6 (CH2), 74.3 (CH), 107.9 (CH), 108.1 (CH), 110.7 (CH), 116.2 (CH), 116.7 (CH), 117.8 (CH), 118.3 (C), 120.4 (d, ^{2}J(C,F) = 15.3 Hz, C), 121.8 (CH), 124.9 (CH), 124.9 (CH), 133.8 (C), 137.4 (CH), 137.5 (CH), 146.2 (d, ^{2}J(C,F) = 10.0 Hz, C), 148.9 (d, ^{1}J(C,F) = 246.0 Hz, C), 150.8 (C), 155.2 (C), 159.8 (C), 166.9 (C), 167.5 (C), 170.1 (C), 172.8 (C), 173.8 (C) ppm

1 quaternary carbon missing

<u>^{19}F NMR (DMSO-d6, 377MHz):</u> δ = -136.30 ppm

1 quaternary carbon is missing.

<u>HRMS</u>: ESI^{+}, *m/z* 829.2691 [M+H]$^{+}$, Error: 0.29 ppm

Synthesis of PROTAC 5

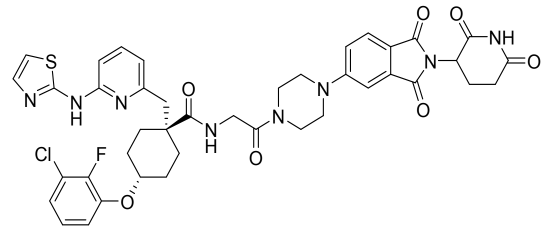

Following the general PROTAC synthesis procedure, 52 mg of the product was obtained with a yield of 61%.

<u>^{1}H NMR (DMSO-d6, 400MHz):</u> δ = 1.72-1.96 (m, 8H), 1.97-2.08 (m, 1H), 2.46-2.69 (m, 2H, overlap with solvent peak), 2.82-2.99 (m, 3H), 3.44-3.68 (m, 8H), 3.97 (d, J = 5.1 Hz, 2H), 4.59 (s, 1H), 5.08 (dd, J = 5.4; 12.9 Hz, 1H), 6.72 (d, J = 7.3 Hz, 1H), 6.86 (d, J = 8.2 Hz, 1H), 6.95 (d, J = 3.6 Hz, 1H), 7.06-7.19 (m, 3H), 7.26 (dd, J = 2.0; 8.6 Hz, 1H), 7.34-7.39 (m, 2H), 7.54 (t, J = 7.8 Hz, 1H), 7.67-7.76 (m, 2H), 11.08 (s, 1H), 11.11 (s, 1 H) ppm.

<u>^{13}C NMR (DMSO-d6, 100MHz):</u> δ = 22.2 (CH2), 26.4 (CH2), 28.2 (CH2), 31.0 (CH2), 40.7 (2 x CH2), 43.2 (CH2), 46.45 (C), 46.7 (CH2), 47.3 (CH2), 48.8 (CH), 74.0 (CH), 108.0 (CH), 108.1 (CH), 110.7 (CH), 116.3 (CH), 116.7 (CH), 117.8 (CH), 118.5 (C), 120.4 (d, ^{2}J (C,F) = 15.1 Hz, C), 121.7 (CH), 124.9 (CH), 124.9 (CH), 133.9 (C), 137.4 (CH), 137.6

(CH), 146.2 (d, ^{2}J (C,F) = 10.1 Hz, C), 148.9 (d, ^{1}J(C,F) = 245.8 Hz, C), 150.7 (C), 154,8 (C), 155.0 (C), 159.8 (C), 167.0 (C), 167.5 (C), 167.5 (C), 170.1 (C), 172.8 (C), 174.3 (C) ppm.

^{19}F NMR (DMSO-d6, 377MHz): δ = -136.3 ppm

HRMS: ESI^{+}, *m/z* 843.2492 [M+H]$^{+}$, Error: 0.71 ppm

Synthesis of PROTAC 6

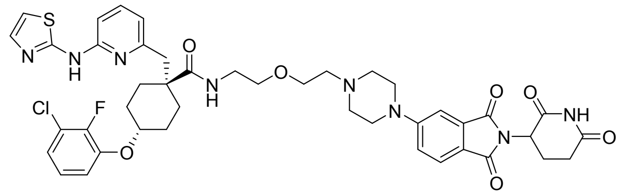

Following the general PROTAC synthesis procedure, 51 mg of the product was obtained with a yield of 58%.

^{1}H NMR (DMSO-d6, 400MHz): δ = 1.58-1.95 (m, 8H), 1.96-2.06 (m, 1H), 2.45-2.70 (m, 8H, overlap with solvent peak), 2.82-2.97 (m, 3H), 3.16-3.26 (m, 2H), 3.29-3.44 (m, 6H), 3.48-3.57 (m,2H), 4.54 (s, 1H), 5.07 (dd, J = 5.4; 12.9 Hz, 1H), 6.64 (d, J = 7.3 Hz, 1H), 6.87 (d, J = 8.2 Hz, 1H), 6.95 (d, J = 3.6 Hz, 1H), 7.03-7.16 (m, 3H), 7.19 (dd, J = 2.0; 8.7 Hz, 1H), 7.31 (d, J = 1.4 Hz, 2H), 7.36 (d, J = 3.6 Hz, 1H), 7.51-7.68 (m, 3H), 11.08 (s, 1H), 11.13 (s, 1H) ppm

^{13}C NMR (DMSO-d6, 100MHz): δ = 22.2 (CH2), 26.6 (CH2), 28.4 (CH2), 31.0 (CH2), 38.8 (CH2), 46.6 (C), 46.9 (2 x CH2), 48.8 (CH), 52.6 (CH2), 57.1 (CH2), 68.0 (CH2), 68.8 (CH2), 74.4 (CH), 107.9 (CH), 108.1 (CH), 110.7 (CH), 116.2 (CH), 116.7 (CH), 117.8 (CH), 118.4 (C), 120.42 (d, ^{2}J(C,F) = 15.1 Hz, C), 121.9 (CH), 124.9 (CH), 124.9 (d, ^{3}J(C,F)= 4.8 Hz, CH), 133.8 (C), 137.4 (CH), 137.6 (CH), 146.2 (d, ^{2}J(C,F) = 10.1 Hz, C), 148.9 (d, ^{1}J(C,F) = 245.8 Hz, C), 150.8 (C), 155.2 (2 x C), 159.8 (C), 167.0 (C), 167.5 (C), 170.1 (C), 172.8 (C), 174.2 (C) ppm

^{19}F NMR (DMSO-d6, 377MHz): δ = -136.30 ppm

HRMS: ESI^{+}, *m/z* 873.2951 [M+H]$^{+}$, Error: 0.52 ppm

Synthesis of PROTAC 7

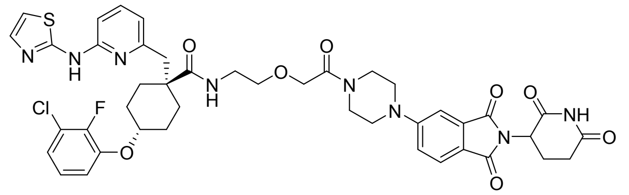

Following the general PROTAC synthesis procedure, 56 mg of the product was obtained with a yield of 64%.

^{1}H NMR (DMSO-d6, 400MHz): δ = 1.59-1.96 (m, 8H), 1.97-2.08 (m, 1H), 2.46-2.69 (m, 2H, overlap with solvent peak), 2.82-2.96 (m, 3H), 3.18-3.24 (m, 2H), 3.41-3.62 (m, 10H), 4.20 (s, 2H), 4.54 (s, 1H), 5.08 (dd, J = 5.4; 12.9 Hz, 1H), 6.62 (d, J = 7.3 Hz, 1H), 6.86 (d, J = 8.2 Hz, 1H), 6.95 (d, J = 3.6 Hz, 1H), 7.06-7.19 (m, 3H), 7.26 (dd, J = 2.0; 8.6 Hz, 1H), 7.34-7.39 (m, 2H), 7.54 (t, J = 7.8 Hz, 1H), 7.69 (d, J= 8.5Hz, 1H), 7.80 (t, J= 4.9 Hz, 1H), 11.08 (s, 1H), 11.11 (s, 1 H) ppm.

^{13}C NMR (DMSO-d6, 100MHz): δ = 22.2 (CH2), 26.7 (CH2), 28.3 (CH2), 31.0 (CH2), 38.8 (CH2), 40.5 (CH2), 43.2 (CH2), 46.4 (C), 46.6 (CH2), 46.7 (CH2), 48.8 (CH), 68.7 (CH2), 69.1 (CH2), 74.4 (CH), 108.0 (CH), 108.1 (CH), 110.7 (CH), 116.2 (CH), 116.7 (CH), 117.8 (CH), 118.5 (C), 120.4 (d, ^{2}J (C,F) = 15.1 Hz, C), 121.8 (CH), 124.9 (CH), 124.9

(CH), 133.9 (C), 137.4 (CH), 137.5 (CH), 146.2 (d, ^{2}J (C,F) = 10.1 Hz, C), 148.9 (d, ^{1}J(C,F) = 245.9 Hz, C), 150.7 (C), 154,8 (C), 155.1 (C), 159.8 (C), 166.9 (C), 167.5 (C), 167.8 (C), 170.1 (C), 172.8 (C), 174.1 (C) ppm.

<u>^{19}F NMR (DMSO-d6, 377MHz):</u> δ = -136.3 ppm

<u>HRMS</u>: ESI^{+}, *m/z* 887.2758 [M+H]$^{+}$, Error: 1.10 ppm

Synthesis of PROTAC 8

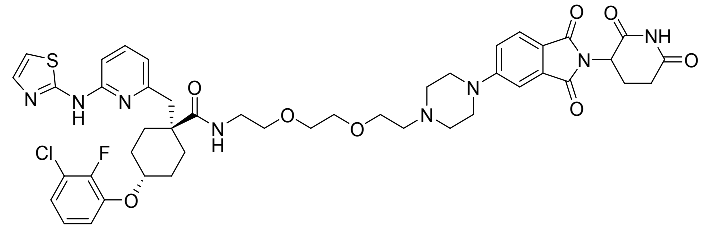

Following the general PROTAC synthesis procedure, 37 mg of the product was obtained with a yield of 40 %.

<u>^{1}H NMR (DMSO-d6, 400MHz):</u> δ = 1.57-1.95 (m, 8H), 1.96-2.06 (m, 1H), 2.45-2.70 (m, 8H, overlap with solvent peak), 2.81-2.98 (m, 3H), 3.16-3.24 (m,2H), 3.27-3.44 (m, 6H, overlap with H_2O peak), 3.46-3.57 (m, 6H), 4.54 (s, 1H), 5.07 (dd, J = 5.3; 12.9 Hz, 1H), 6.64 (d, J = 7.3 Hz, 1H), 6.86 (d, J = 8.2 Hz, 1H), 6.95 (d, J = 3.6 Hz, 1H), 7.05-7.17 (m, 3H), 7.21 (dd, J = 1.6; 8.6 Hz, 1H), 7.31 (d, J = 1.3 Hz, 1H), 7.35 (d, J = 3.6 Hz, 1H), 7.55 (t, J = 7.8 Hz, 1H), 7.59-7.68 (m, 2H), 11.08 (s, 1H), 11.12 (s, 1H) ppm

<u>^{13}C NMR (DMSO-d6, 100MHz)</u>: δ = 22.2 (CH2), 26.6 (CH2), 28.4 (CH2), 31.0 (CH2), 38.8 (CH2), 46.6 (C), 46.8 (2 x CH2), 48.8 (CH), 52.7 (CH2), 57.1 (CH2), 68.4 (CH2), 68.9 (CH2), 69.5 (CH2), 69.7 (CH2), 74.4 (CH), 107.9 (CH), 108.1 (CH), 110.7 (CH), 116.2 (CH), 116.7 (CH), 117.7 (CH), 118.3 (C), 120.43 (d, ^{2}J(C,F) = 15.2 Hz, C), 121.9 (CH), 124,9 (CH), 124.9 (d, ^{3}J(C,F)= 4.8 Hz, CH), 133.8 (C), 137.4 (CH), 137.6 (CH), 146.2 (d, ^{2}J(C,F) = 10.2 Hz, C), 148.92 (d, ^{1}J(C,F) = 246.0 Hz, C), 150.7 (C), 155.2 (C), 155.2 (C), 159.8 (C), 167.0 (C), 167.6 (C), 170.1 (C), 172.8 (C), 174.1 (C) ppm.

<u>^{19}F NMR (DMSO-d6, 377MHz):</u> δ = -136.30 ppm

<u>HRMS</u>: ESI^{+}, *m/z* 917.3209 [M+H]$^{+}$, Error: 0.95 ppm

Synthesis of PROTAC 9

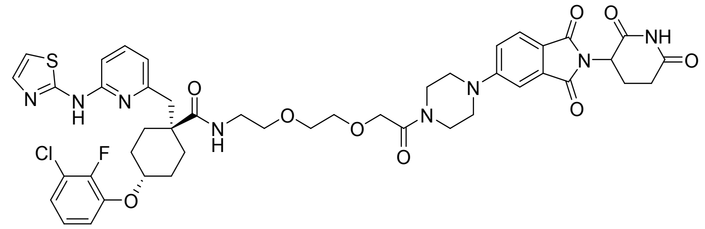

Following the general PROTAC synthesis procedure, 51 mg of the product was obtained with a yield of 54%.

<u>^{1}H NMR (DMSO-d6, 400MHz):</u> δ = 1.59-1.96 (m, 8H), 1.97-2.08 (m, 1H), 2.46-2.69 (m, 2H, overlap with solvent peak), 2.82-2.96 (m, 3H), 3.18-3.24 (m, 2H), 3.38 (t, J= 6.1 Hz, 2H), 3.41-3.62 (m, 12H), 4.19 (s, 2H), 4.54 (s, 1H), 5.08 (dd, J = 5.4; 12.9 Hz, 1H), 6.62 (d, J = 7.3 Hz, 1H), 6.86 (d, J = 8.2 Hz, 1H), 6.95 (d, J = 3.6 Hz, 1H), 7.06-7.19 (m, 3H), 7.23 (dd, J = 2.0; 8.6 Hz, 1H), 7.34-7.39 (m, 2H), 7.54 (t, J = 7.8 Hz, 1H), 7.62 (t, J= 5.5 Hz, 1H), 7.68 (d, J= 8.5Hz, 1H), 11.08 (s, 1H), 11.11 (s, 1 H) ppm.

<u>^{13}C NMR (DMSO-d6, 100MHz):</u> δ = 22.2 (CH2), 26.7 (CH2), 28.3 (CH2), 31.0 (CH2), 38.8 (CH2), 40.5 (CH2), 43.5 (CH2), 46.4 (CH2), 46.6 (C), 46.8 (CH2), 48.8 (CH), 68.8 (CH2), 69.3 (CH2), 69.4 (CH2), 69.8 (CH2), 74.3 (CH), 108.0

(CH), 108.1 (CH), 110.7 (CH), 116.2 (CH), 116.7 (CH), 117.8 (CH), 118.5 (C), 120.4 (d, ^{2}J (C,F) = 15.1 Hz, C), 121.8 (CH), 124.9 (CH), 124.9 (CH), 133.9 (C), 137.4 (CH), 137.5 (CH), 146.2 (d, ^{2}J (C,F) = 10.1 Hz, C), 148.9 (d, ^{1}J(C,F) = 245.9 Hz, C), 150.7 (C), 154,8 (C), 155.1 (C), 159.8 (C), 166.9 (C), 167.5 (C), 167.5 (C), 170.1 (C), 172.8 (C), 174.1 (C) ppm.

^{19}F NMR (DMSO-d6, 377MHz): δ = -136.3 ppm

HRMS: ESI$^+$, *m/z* 931.3019 [M+H]$^+$, Error: 0.93 ppm

Synthesis of PROTAC 10

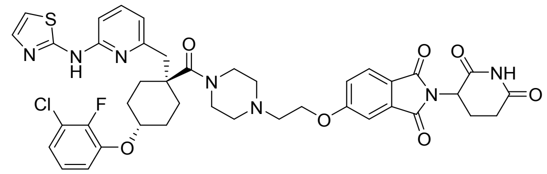

Following the general PROTAC synthesis procedure, 37 mg of the product was obtained with a yield of 45%.

^{1}H NMR (DMSO-d6, 400MHz): δ = 1.65-1.86 (m, 6H), 1.91-2.10 (m, 3H), 2.52-2.65 (m, 6H), 2.77 (s, 2H), 2.83-2.95 (m, 1H), 3.06 (s, 2H), 3.66 (s, 4H), 4.32 (m, 2H), 4.55 (s, 1H), 5.12 (dd, J = 5.3; 12.9 Hz, 1H), 6.67 (d, J = 7.3 Hz, 1H), 6.88 (d, J = 8.2 Hz, 1H), 6.96 (d, J = 3.1 Hz, 1H), 7.02-7.16 (m, 3H), 7.33-7.39 (m, 2H), 7.47 (s, 1H), 7.57 (t, J=7.7 Hz, 1H), 7.83 (d, J= 8.2 Hz, 1H), 11.11 (s, 1H), 11,15 (s, 1H) ppm

^{13}C NMR (DMSO-d6, 100MHz): δ = 22.0 (CH2), 26.9 (CH2), 29.5 (CH2), 31.0 (CH2), 44.9 (2x CH2), 47.1 (C), 49.0 (CH), 53.1 (CH2), 56.1 (CH2), 66.8 (CH2), 73.8 (CH), 108.3 (CH), 109.0 (CH), 110.7 (CH), 116.2 (CH), 116.5 (CH), 120.4 (d, ^{2}J(C,F) = 15.1 Hz, C), 120.9 (CH), 121.8 (CH), 123.0 (C), 124.9 (d, ^{3}J(C,F)= 4.9 Hz, CH), 125.3 (CH), 133.9 (C), 137.4 (CH), 137.7 (CH), 146.2 (d, ^{2}J(C,F) = 10.1 Hz, C), 148.8 (d, ^{1}J(C,F) = 245.8 Hz, C), 150.9 (C), 154.9 (C), 159.7 (C), 163.9 (C), 166.8 (C), 166.9 (C), 169.9 (C), 171.8 (C), 172.8 (C) ppm.

^{19}F NMR (DMSO-d6, 377MHz): δ = -136.4 ppm

HRMS: ESI$^+$, *m/z* 830.2532 [M+H]$^+$, Error: 0.18 ppm

Synthesis of PROTAC 11

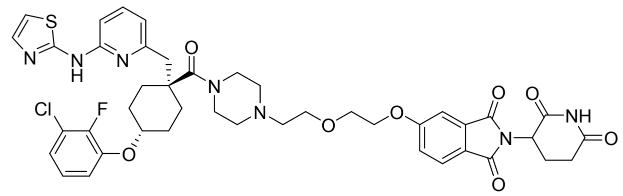

Following the general PROTAC synthesis procedure, 42 mg of the product was obtained with a yield of 48%.

^{1}H NMR (DMSO-d6, 400MHz): δ = 1.65-1.86 (m, 6H), 1.91-2.10 (m, 3H), 2.42 (s, 4H), 2.52-2.65 (m, 4H), 2.83-2.95 (m, 1H), 3.04 (s, 2H), 3.57-3.65 (m, 6H), 3.77 (m, 2H), 4.32 (m, 2H), 4.55 (s, 1H), 5.12 (dd, J = 5.3; 12.9 Hz, 1H), 6.67 (d, J = 7.3 Hz, 1H), 6.88 (d, J = 8.2 Hz, 1H), 6.96 (d, J = 3.4 Hz, 1H), 7.02-7.16 (m, 3H), 7.33-7.39 (m, 2H), 7.45 (m, 1H), 7.57 (t, J=7.7 Hz, 1H), 7.83 (d, J= 8.2 Hz, 1H), 11.11 (s, 1H), 11,15 (s, 1H) ppm

^{13}C NMR (DMSO-d6, 100MHz): δ = 22.1 (CH2), 26.9 (CH2), 29.5 (CH2), 31.0 (CH2), 44.9 (2x CH2), 47.1 (C), 49.0 (CH), 53.2 (CH2), 56.9 (CH2), 68.4 (2x CH2), 68.5 (CH2), 73.8 (CH), 108.3 (CH), 108.9 (CH), 110.7 (CH), 116.2 (CH), 116.5 (CH), 120.4 (d, ^{2}J(C,F) = 15.1 Hz, C), 120.9 (CH), 121.8 (CH), 123.1 (C), 124.9 (d, ^{3}J(C,F)= 4.9 Hz, CH), 125.3

(CH), 133.9 (C), 137.4 (CH), 137.7 (CH), 146.2 (d, ^2J(C,F) = 10.1 Hz, C), 148.8 (d, ^1J(C,F) = 245.8 Hz, C), 150.9 (C), 154.9 (C), 159.7 (C), 163.9 (C), 166.8 (C), 166.9 (C), 169.9 (C), 171.8 (C), 172.8 (C) ppm

^{19}F NMR (DMSO-d6, 377MHz): δ = -136.4 ppm

HRMS: ESI$^+$, *m/z* 874.2795 [M+H]$^+$, Error: 0.08 ppm

SYNTHESIS OF SERIES 2 PROTACS

Synthesis of rigid linker units

Scheme S3. *Synthesis of various rigid linker units*

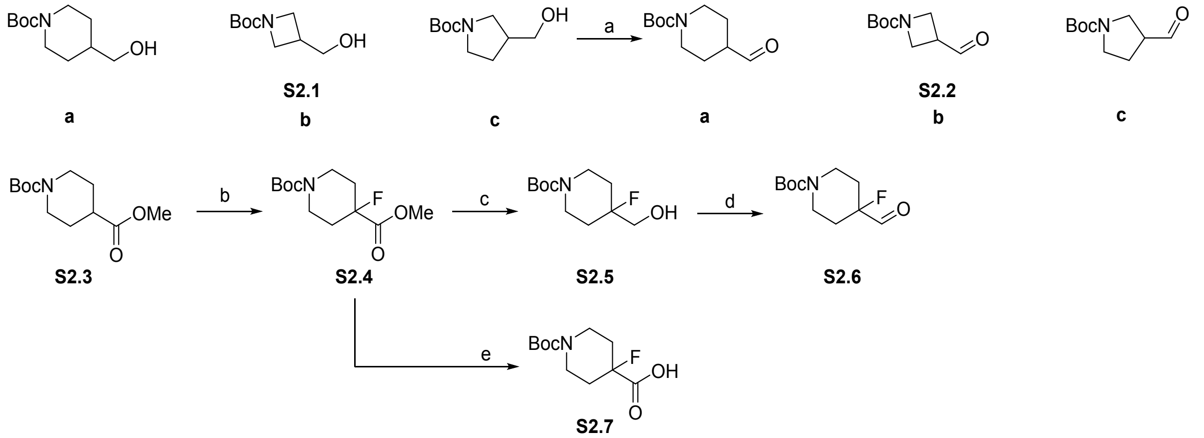

Reagents and conditions: ***a)*** *DMP, DCM, 0°C -> RT, 76% - 86%;* ***b)*** *LDA, NFSI, THF, -78 °C, 61%;* ***c)*** *$LiAlH_4$, THF, 0°C, 80%;* ***d)*** *Oxalyl chloride, DMSO, Et_3N, DCM, 81%;* ***e)*** *2M NaOH, MeOH, 92%.*

Synthesis of *tert*-butyl 4-formylpiperidine-1-carboxylate (S2.2a)

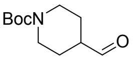

Following the general alcohol oxidation procedure 5, 800 mg of the product was obtained with a yield of 81%.

^{1}H NMR ($CDCl_3$, 400MHz): δ = 1.45 (s, 9H), 1.49-1.62 (m, 2H), 1.84-1.93 (m, 2H), 2.35-2.46 (m, 1H), 2.86-2.97 (m, 2H), 3.91-4.03 (m, 2H), 9.66 (d, J= 0.7 Hz, 1H) ppm.

^{13}C NMR ($CDCl_3$, 100 MHz): δ = 25.3 (CH2), 28.6 (CH3), 42.9 (CH2), 48.1 (CH), 79.9 (C), 154.8 (C), 203.1 (CH) ppm.

Synthesis of *tert*-butyl 3-formylazetidine-1-carboxylate (S2.2b)

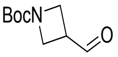

Following the general alcohol oxidation procedure 5, 760 mg of the product was obtained with a yield of 77%. $NaHCO_3$ (5 eq) were added to the RM to reduce formation of aldol by-products.

^{1}H NMR ($CDCl_3$, 400MHz): δ = 1.43 (s, 9H), 3.30-3.40 (m, 1H), 4.04-4.15 (m, 4H), 9.84 (d, J= 2.1 Hz, 1H) ppm.

^{13}C NMR ($CDCl_3$, 100 MHz): δ = 28.5 (CH3), 38.9 (CH), 49.0 (CH2), 80.2 (C), 156.2 (C), 199.2 (CH) ppm.

Synthesis of *tert*-butyl 3-formylpyrrolidine-1-carboxylate (S2.2c)

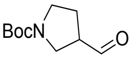

Following the general alcohol oxidation procedure 5, 850 mg of the product was obtained with a yield of 86%.

^{1}H NMR ($CDCl_3$, 400MHz): δ = 1.45 (s, 9H), 2.01-2.22 (m, 2H), 3.02 (br s, 1H), 3.28-3.61 (m, 3H), 3.62-3.73 (m, 1H), 9.69 (d, J= 1.6 Hz, 1H) ppm.

^{13}C NMR ($CDCl_3$, 100 MHz): δ = 25.5 (CH2), 26.0 (CH2), 28.6 (CH3), 44.9 (CH2), 45.2 (2 x CH2), 49.8 (CH), 50.7 (CH), 79.8 (C), 154.4 (C), 200.8 (CH) ppm.

Synthesis of 1-(*tert*-butyl) 4-methyl 4-fluoropiperidine-1,4-dicarboxylate (S2.4)

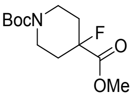

An oven-dried 2-necked flask was cooled down to -78°C and THF (100 mL), diisopropylamine (7.0 mL, 49.3 mmol, 1.2 eq) and butyllithium (30.8 mL of a 1.6 M solution in hexanes, 49.3 mmol, 1.2 eq) were subsequently added. Next, 1-(*tert*-butyl) 4-methyl piperidine-1,4-dicarboxylate (10 g, 41.1 mmol, 1.0 eq) was added, followed by N-fluorobenzenesulfonimide (NFSI) (15.6 g, 49.3 mmol, 1.2 eq) 30 minutes later. The cooling bath was removed and the reaction was stirred overnight for 16h. Sat. NH_4Cl was added and extracted twice with EA. The organic layer was dried over Na_2SO_4, filtered and evaporated. Flash column chromatography (PE/EA 0 -> 20%) delivered the product (6.5 g) in 61%.

^{1}H NMR ($CDCl_3$, 400MHz): δ = 1.46 (s, 9H), 1.82-2.11 (m, 4H), 3.10 (br s, 2H), 3.80 (s, 3H), 4.01 (br s, 2H) ppm.

^{13}C NMR ($CDCl_3$, 100 MHz): δ = 28.5 (CH3), 32.3 (d, ^{2}J= 21.7 Hz, CH2), 38.6 (CH2, br s), 52.9 (CH3), 80.1 (C), 92.3 (d, ^{1}J= 186.3 Hz, C), 154.7 (C), 171.5 (d, ^{2}J= 24.5 Hz, C) ppm.

^{19}F NMR ($CDCl_3$, 370 MHz): δ = -168.4 ppm

Synthesis of *tert*-butyl 4-fluoro-4-(hydroxymethyl)piperidine-1-carboxylate (S2.5)

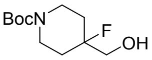

1-(*tert*-butyl) 4-methyl 4-fluoropiperidine-1,4-dicarboxylate (7.7g, 29.5 mmol, 1.0 eq) was treated with $LiAlH_4$ (1.3g, 35.4 mmol, 1.2 eq) in THF at 0°C. Next, a Fieser work-up was employed to quench the reaction: 1.3 mL water, 1.3 mL 4M NaOH and 3.9 mL water were subsequently added. The solids were filtered and flushed with DCM. The filtrate dried over Na_2SO_4, filtered and evaporated. Flash column chromatography (DCM/MeOH 0 -> 5%) delivered the product (5.5 g) in 80% yield.

^{1}H NMR ($CDCl_3$, 400MHz): δ = 1.46 (s, 9H), 1.46-1.63 (m, 2H), 1.83-1.93 (m, 3H), 3.10 (t, J= 12.3 Hz, 2H), 3.60 (dd, J= 6.6; 20.2 Hz, 2H), 3.93 (br s, 2H) ppm.

^{13}C NMR ($CDCl_3$, 100 MHz): δ = 28.6 (CH3), 31.4 (d, ^{2}J= 21.4 Hz, CH2), 39.4 (CH2, br s), 68.5 (d, ^{2}J= 23.3 Hz, CH2), 79.9 (C), 94.6 (d, ^{1}J= 171.0 Hz, C), 154.9 (C) ppm.

^{19}F NMR ($CDCl_3$, 370 MHz): δ = -171.0 ppm

Synthesis of *tert*-butyl 4-fluoro-4-formylpiperidine-1-carboxylate (S2.6)

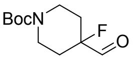

An oven-dried 2-necked flask was cooled down to -78°C and DCM (70 mL), oxalyl chloride (2.2 mL, 25.2 mmol, 1.2 eq) and DMSO (3.6 mL, 50.4 mmol, 2.4 eq) were subsequently added. After 30 min, the alcohol (4.9 g, 21.0 mmol, 1.0 eq) was added, followed by addition of Et_3N (11.7 mL, 84.0 mmol, 4.0 eq) another 30 minutes later. The cooling bath was removed and the reaction was allowed to come to RT for 1h. Water was added and extracted twice with DCM. The organic layer was dried over Na_2SO_4, filtered and evaporated. Flash column chromatography (cyclohexane/EA 10 -> 70%) delivered the aldehyde (3.9 g) in 81%.

^{1}H NMR ($CDCl_3$, 400MHz): δ = 1.46 (s, 9H), 1.72-1.87 (m, 4H), 3.10 (br s, 2H), 4.02 (br s, 2H), 9.66 (d, J= 4.9 Hz, 1H) ppm.

^{13}C NMR ($CDCl_3$, 100 MHz): δ = 28.5 (CH3), 29.8 (d, ^{2}J= 21.3 Hz, CH2), 38.2 (CH2, br s), 79.9 (C), 96.4 (d, ^{1}J= 179.3 Hz, C), 154.6 (C), 200.0 (d, ^{2}J= 39.8 Hz, CH) ppm.

^{19}F NMR ($CDCl_3$, 370 MHz): δ = -173.6 ppm.

Synthesis of 1-(*tert*-butoxycarbonyl)-4-fluoropiperidine-4-carboxylic acid (S2.7)

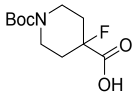

1-(*tert*-butyl) 4-methyl 4-fluoropiperidine-1,4-dicarboxylate (1.5g, 5.74 mmol, 1.0 eq) was treated with 2 M NaOH (14.4 mL, 28.8 mmol, 5 eq) in MeOH (10 mL) for 4h. The RM was cooled down to 0°C, quenched with 2M HCl until pH 1 to 2 was reached, followed by an extraction with DCM (x 2). This delivered the product (1.3 g) in 92% after drying *in vacuo*.

^{1}H NMR ($CDCl_3$, 400MHz): δ = 1.46 (s, 9H), 1.90-2.16 (m, 4H), 3.08 (br s, 2H), 4.01 (br s, 2H), 10.06 (br s, 1H) ppm.

^{13}C NMR ($CDCl_3$, 100 MHz): δ = 28.5 (CH3), 32.0 (d, ^{2}J= 21.7 Hz, CH2), 39.2 (CH2, br s), 80.7 (C), 91.9 (d, ^{1}J= 186.4 Hz, C), 154.9 (C), 175.1 (d, ^{2}J= 25.2 Hz, C) ppm.

^{19}F NMR ($CDCl_3$, 370 MHz): δ = -168.5 ppm

Synthesis of arylaminoglutaramide-based CRBN intermediates

Scheme S4. *Synthesis of arylaminoglutarimide-based PROTACs*

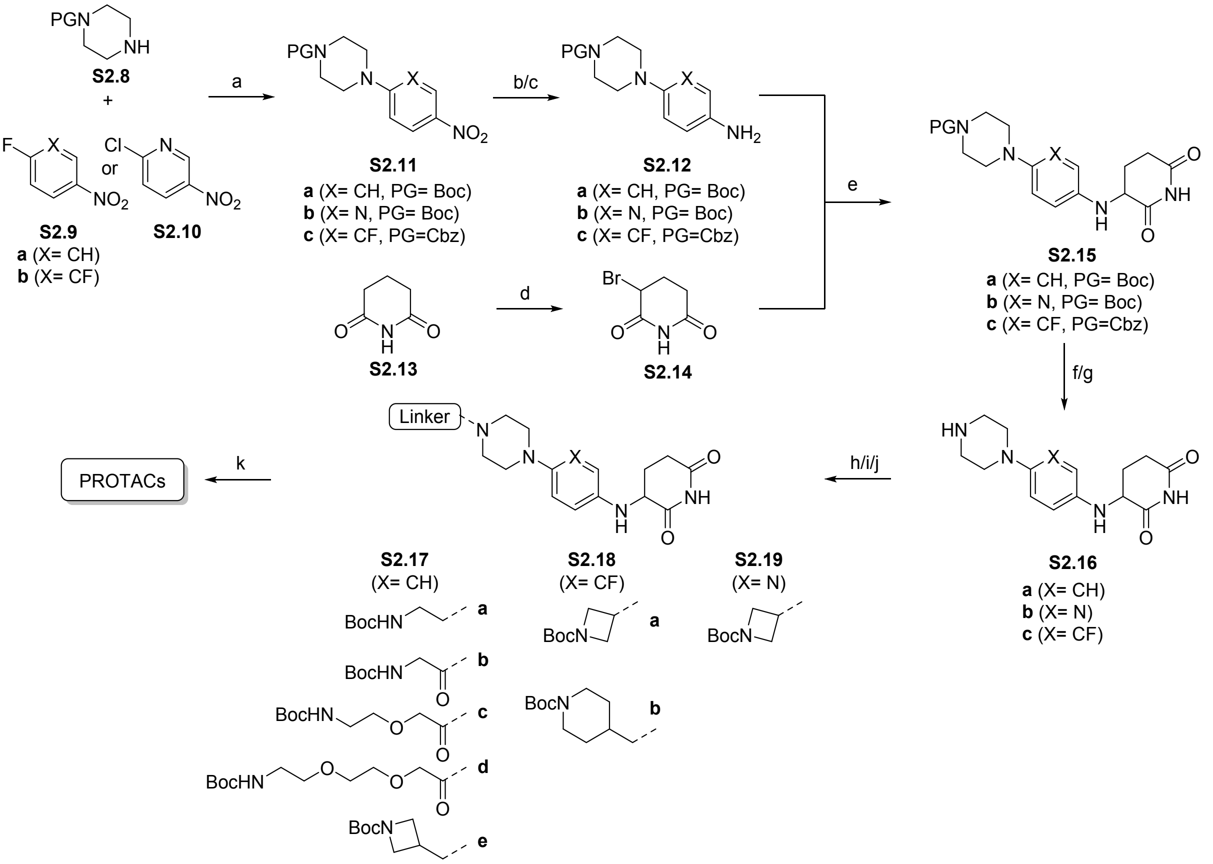

<u>Reagents and conditions:</u> ***a)*** *DIPEA, MeCN, 80 °C, 83% (a), 98% (b), 89% (c);* ***b)*** *H_2, Pd/C 10%, 98% (a), 88% (b);* ***c)*** *$SnCl_2$, EtOH, 80 °C, 40% (c),* ***d)*** *Br_2, $CHCl_3$, 110 °C, 55%,* ***e)*** *DIPEA, DMF, 80 °C, 60% (a), 61% (b), 60% (c);* ***f)*** *TFA, DCM (a) or 4M HCl in dioxane (b), quantitative yield;* ***g)*** *H_2, Pd/C 10%, 75% (c);* ***h)*** *tert-butyl (2-bromoethyl)carbamate or tert-butyl 3-(((methylsulfonyl)oxy)methyl)azetidine-1-carboxylate, $NaHCO_3$, DMF, 70 °C, 60% (a), 31% (e);* ***i)*** *HATU, DIPEA, DMF, 78% (b), 56% (c), 75% (d);* ***j)*** *appropriate aldehyde or ketone, STAB, $NaHCO_3$, DMF, 47% (3.49 a), 37% (3.49 b), 65% (3.50);* ***k)*** *HATU, DIPEA, DMF, 27- 69%.*

Synthesis of *tert*-butyl 4-(4-nitrophenyl)piperazine-1-carboxylate (S2.11a)

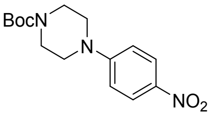

1-fluoro-4-nitrobenzene (5g, 35.4 mmol, 1 eq) and *N*-Boc-piperazine (6.9 g, 37.2 mmol, 1.05 eq) were dissolved in MeCN, followed by addition of DIPEA (6.8 mL, 39.0 mmol, 1.1 eq) and subsequent heating to 80°C for 18h. Then the reaction was slowly cooled down to RT. The precipitate was filtered and washed once with ice cold MeCN. After dying in vacuo, 9.0 g of the product was obtained in 83% yield.

^{1}H NMR ($CDCl_3$, 400MHz): δ = 1.46 (s, 9H), 3.39-3.43 (m, 4H), 3.58-3.63 (m, 4H), 6.83 (d, J= 9.4 Hz, 2H), 8.12 (d, J= 9.4 Hz, 2H) ppm.

^{13}C NMR ($CDCl_3$, 100 MHz): δ = 28.5 (CH3), 43.0 (CH2), 47.2 (CH2), 80.5 (C), 113.2 (CH), 126.1 (CH), 139.1 (C), 154.6 (C), 154.7 (C) ppm.

HRMS: ESI$^+$, *m/z* 308.1610 $[M+H]^+$, Error: 1.65 ppm

Synthesis of *tert*-butyl 4-(5-nitropyridin-2-yl)piperazine-1-carboxylate (S.2.11 b)

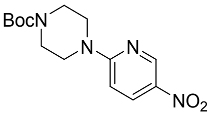

Following the same synthetic procedure as for *tert*-butyl 4-(4-nitrophenyl)piperazine-1-carboxylate, however with 2-chloro-5-nitropyridine as starting material, delivered 19.1 g of the product in 98% yield.

^{1}H NMR ($CDCl_3$, 400MHz): δ = 1.48 (s, 9H), 3.53-3.58 (m, 4H), 3.74-3.78 (m, 4H), 6.56 (d, J= 9.5 Hz, 1H), 8.20 (dt, J= Hz, 1H), 9.02 (t, J= Hz, 1H) ppm.

^{13}C NMR ($CDCl_3$, 100 MHz): δ = 28.5 (CH3), 42.6 (CH2), 44.7 (CH2), 80.5 (C), 104.7 (CH), 133.2 (CH), 135.4 (C), 146.5 (CH), 154.7 (C), 160.4 (C) ppm.

HRMS: ESI$^+$, *m/z* 309.1559 $[M+H]^+$, Error: 0.53 ppm

Synthesis of benzyl 4-(2-fluoro-4-nitrophenyl)piperazine-1-carboxylate (S.2.11 c)

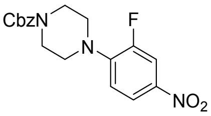

Following the same synthetic procedure as for *tert*-butyl 4-(4-nitrophenyl)piperazine-1-carboxylate, however with 1,2-difluoro-4-nitrobenzene and *N*-Cbz piperazine as starting materials, delivered 10.0 g of the product in 89% yield.

^{1}H NMR ($CDCl_3$, 400MHz): δ = 3.26 (br s, 4H), 3.67-3.72 (m, 4H), 5.17 (s, 2H), 6.91 (t, J= 8.8 Hz, 1H), 7.30-7.41 (m, 5H), 7.92 (dd, J= 2.6; 12.8 Hz, 1H), 7.99 (ddd, J= 0.9; 2.5; 8.9 Hz, 1H) ppm.

^{13}C NMR ($CDCl_3$, 100 MHz): δ = 43.7 (CH2), 49.6 (CH2), 49.7 (CH2), 67.6 (CH2), 112.8 (d, J= 26.2 Hz, CH), 117.5 (d, J= 3.6 Hz, CH), 121.1 (d, J= 2.9 Hz, CH), 128.2 (CH), 128.5 (CH), 128.7 (CH), 136.5 (C), 141.2 (d, J= 8.7 Hz, C), 145.5 (d, J= 7.7 Hz, C), 153.3 (d, J= 249.5 Hz, C), 155.3 (C) ppm.

^{19}F NMR ($CDCl_3$, 370 MHz): δ = -118.7 ppm

Synthesis of *tert*-butyl 4-(4-aminophenyl)piperazine-1-carboxylate (S2.12a)

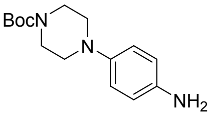

Tert-butyl 4-(4-nitrophenyl)piperazine-1-carboxylate (9.0 g, 29.3 mmol) was dissolved in MeOH and Pd/C 10% was added. The reaction mixture was purged with N_2, followed by gas exchange with H_2 (2 balloons). After 18h of stirring, the reaction mixture was filtered over celite and the filtrate evaporated. This delivered the product (8 g) in 98% yield.

<u>^{1}H NMR (CD_3OD, 400MHz):</u> δ = 1.47 (s, 9H), 2.91-2.95 (m, 4H), 3.51-3.56 (m, 4H), 6.71 (d, J= 8.8 Hz, 2H), 6.84 (d, J= 8.8 Hz, 2H) ppm.

<u>^{13}C NMR (CD_3OD, 100 MHz):</u> δ = 28.5 (CH3), 44.7 (CH2), 52.6 (CH2), 81.3 (C), 117.9 (CH), 120.5 (CH), 142.7 (C), 145.6 (C), 156.4 (C) ppm.

<u>HRMS</u>: ESI$^+$, *m/z* 278.1859 [M+H]$^+$, Error: 1.46 ppm

Synthesis of *tert*-butyl 4-(5-aminopyridin-2-yl)piperazine-1-carboxylate (S2.12 b)

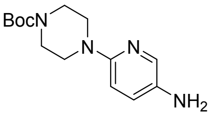

Tert-butyl 4-(5-nitropyridin-2-yl)piperazine-1-carboxylate (19.1 g, 61.9 mmol) was dissolved in MeOH and Pd/C 10% was added. The reaction mixture was purged with N_2, followed by gas exchange with H_2 (2 balloons). After 18h of stirring, the reaction mixture was filtered over celite and the filtrate evaporated. This delivered the product (15.1 g) in 88% yield after purification (DCM/MeOH 0 -> 5% +0.1% NH_4OH).

<u>^{1}H NMR ($CDCl_3$, 400MHz):</u> δ = 1.48 (s, 9H), 3.29-3.34 (m, 4H), 3.49-3.56 (m, 4H), 6.56 (d, J= 8.7 Hz, 1H), 6.98 (dd, J= 2.9; 8.8 Hz, 1H), 7.78 (d, J= 2.9 Hz, 1H) ppm.

<u>^{13}C NMR ($CDCl_3$, 100 MHz):</u> δ = 28.5 (CH3), 43.0 (CH2), 44.1 (CH2), 46.9 (CH2), 79.9 (C), 108.9 (CH), 126.2 (CH), 135.1 (C), 135.4 (CH), 154.4 (C), 155.0 (C) ppm.

<u>HRMS</u>: ESI$^+$, *m/z* 279.1813 [M+H]$^+$, Error: 0.92 ppm

Synthesis of benzyl 4-(4-amino-2-fluorophenyl)piperazine-1-carboxylate (S2.12c)

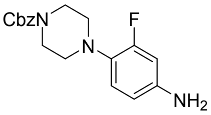

Benzyl 4-(2-fluoro-4-nitrophenyl)piperazine-1-carboxylate (10.0 g, 27.8 mmol, 1.0 eq), $SnCl_2$ (26g, 139 mmol, 5.0 eq) and EtOH (100 mL) were heated at 80°C for 3h. Then, the reaction was cooled down to 0°C and sat. Na_2CO_3 was added, followed by extraction with DCM. Drying over Na_2SO_4, and evaporation of the organic layer delivered 3.62 g of the product in 40% yield after column chromatography (DCM/MeOH 0 -> 5%).

<u>^{1}H NMR ($CDCl_3$, 400MHz):</u> δ = 2.92 (s, 4H), 3.57 (s, 2H), 3.63-3.67 (m, 4H), 5.16 (s, 2H), 6.35-6.45 (m, 2H), 6.77 (t, J= 9.1 Hz, 1H), 7.29-7.41 (m, 5H) ppm.

<u>^{13}C NMR ($CDCl_3$, 100 MHz):</u> δ = 44.3 (CH2), 51.4 (CH2), 67.3 (CH2), 103.9 (d, J= 23.7 Hz, CH), 110.7 (d, J= 2.9 Hz, CH), 120.9 (d, J= 4.3 Hz, CH), 128.0 (CH), 128.2 (CH), 128.6 (CH), 131.6 (d, J= 9.6 Hz, C), 136.8 (C), 143.3 (d, J= 10.6 Hz, C), 155.4 (C), 156.9 (d, J= 245.2 Hz, C) ppm.

^{19}F NMR ($CDCl_3$, 370 MHz): δ = -122.8 ppm

HRMS: ESI$^+$, *m/z* 330.1608 $[M+H]^+$, Error: 1.32 ppm

Synthesis of 3-bromopiperidine-2,6-dione (S2.14)

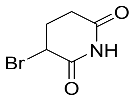

Br_2 (2.15 mL, 41.6 mmol, 1.1 eq) was added to a pressure tube with glutarimide (5g, 37.8 mmol, 1 eq) and $CHCl_3$ (15mL), flushed with Argon. The mixture was heated to 110°C for 1h30 minutes (reaction turns from brown to pale yellow). After cooling down, DCM was added. The organic layers were washed with sat. $NaHCO_3$, dried over Na_2SO_4, and evaporated. Flash column chromatography (PE/EA 10 -> 60%) delivered 4g of the product in 55% yield.

^1H NMR (DMSO-d6, 400MHz): δ = 2.10-2.20 (m, 1H), 2.38-2.50 (m, 1H), 2.51-2.67 (m, 2H), 4.88 (t, J= 4.5 Hz, 1H), 11.05 (s, 1H) ppm.

^{13}C NMR (DMSO-d6, 100 MHz): δ = 26.8 (CH2), 28.7 (CH2), 44.4 (CH) ppm.

2 quaternary carbons missing.

Synthesis of *tert*-butyl 4-(4-((2,6-dioxopiperidin-3-yl)amino)phenyl)piperazine-1-carboxylate (S2.15a)

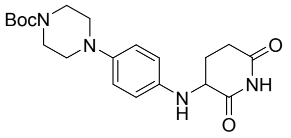

Tert-butyl 4-(4-aminophenyl)piperazine-1-carboxylate (2.18 g, 11.4 mmol, 1.05 eq) and 3-bromopiperidine-2,6-dione (3.0 g, 10.8 mmol, 1.0 eq) were dissolved in DMF and DIPEA (2.45 mL, 14.1 mmol, 1.3 eq) was added. After 2h of stirring at 80°C, the reaction mixture was evaporated on celite and purified via flash column chromatography (DCM/MeOH 0 -> 4 %). This delivered the product (2.5 g) in 60% yield.

^1H NMR ($CDCl_3$, 400MHz): δ = 1.48 (s, 9H), 1.87-1.93 (m, 1H), 2.49-2.56 (m, 1H), 2.67-2.76 (m, 1H), 2.82-2.89 (m, 1H), 2.99 (t, J= 4.7 Hz, 4H), 3.58 (t, J= 4.9Hz, 4H), 4.01 (dd, J= 4.8; 12.4 Hz, 1H), 4.53 (br s, 1H), 6.66 (d, J= 8.8 Hz, 2H), 6.88 (d, J= 8.6 Hz, 2H), 8.17 (s, 1H) ppm.

^{13}C NMR ($CDCl_3$, 100 MHz): δ = 25.9 (CH2), 28.5 (CH3), 31.3 (CH2), 43.4 (CH2, broad peak), 44.4 (CH2, broad peak), 51.2 (CH2), 55.5 (CH), 80.0 (C), 115.1 (CH), 119.4 (CH), 140.9 (C), 145.1 (C, via HMBC), 154.9 (C), 171.5 (C), 172.6 (C) ppm.

Synthesis of *tert*-butyl 4-(5-((2,6-dioxopiperidin-3-yl)amino)pyridin-2-yl)piperazine-1-carboxylate (S2.15b)

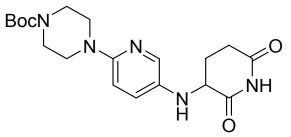

Following the same procedure as for *tert*-butyl 4-(4-((2,6-dioxopiperidin-3-yl)amino)phenyl)piperazine-1-carboxylate, delivered the product (0.85 g) in 61% yield.

^1H NMR ($CDCl_3$, 400MHz): δ = 1.48 (s, 9H), 1.87-1.97 (m, 1H), 2.46-2.52 (m, 1H), 2.64-2.75 (m, 1H), 2.81-2.90 (m, 1H), 3.34-3.38 (m, 4H), 3.51-3.56 (m, 4H), 3.96 (ddd, J= 3.4; 4.6; 12.3 Hz, 1H), 4.35 (d, J= 3.3 Hz, 1H), 6.63 (d, J= 8.9 Hz, 1H), 7.04 (dd, J= 2.9; 8.8 Hz, 1H), 7.79 (d, J= 2.9 Hz, 1H), 8.20 (s, 1H) ppm.

13C NMR ($CDCl_3$, 100 MHz): δ = 25.7 (CH2), 28.5 (CH3), 31.2 (CH2), 43.1 (CH2, broad peak), 44.1 (CH2, broad peak), 46.6 (CH2), 55.8 (CH), 80.0 (C), 108.9 (CH), 126.5 (CH), 134.6 (CH), 135.1 (C), 154.4 (C), 154.9 (C), 171.4 (C), 172.5 (C) ppm.

HRMS: ESI+, *m/z* 390.2132 [M+H]+, Error: 0.97 ppm

Synthesis of benzyl 4-(4-((2,6-dioxopiperidin-3-yl)amino)-2-fluorophenyl)piperazine-1-carboxylate (S2.15c)

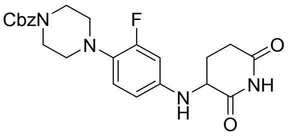

Following the same procedure as for *tert*-butyl 4-(4-((2,6-dioxopiperidin-3-yl)amino)phenyl)piperazine-1-carboxylate, delivered the product (2.9 g) in 60% yield.

1H NMR ($CDCl_3$, 400MHz): δ = 1.83-1.99 (m, 1H), 2.47-2.56 (m, 1H), 2.70-2.81 (m, 1H), 2.84-3.03 (m, 5H), 3.66 (t, J= 5.1 Hz, 4H), 3.99 (dt, J= 4.3; 12.3 Hz, 1H), 4.63 (d, J= 3.8 Hz, 1H), 5.16 (s, 2H), 6.35-6.45 (m, 2H), 6.84 (t, J= 9.1 Hz, 1H), 7.29-7.40 (m, 5H), 8.10 (s, 1H) ppm.

13C NMR ($CDCl_3$, 100 MHz): δ = 25.7 (CH2), 31.6 (CH2), 44.2 (CH2), 51.4 (CH2), 55.1 (CH), 67.4 (CH2), 102.5 (d, J= 24.2 Hz, CH), 109.3 (d, J= 2.8 Hz, CH), 121.1 (d, J= 4.3 Hz, CH), 128.1 (CH), 128.2 (CH), 128.7 (CH), 132.0 (d, J= 9.9 Hz, C), 136.8 (C), 143.1 (d, J= 10.1 Hz, C), 153.3 (C), 157.1 (d, J= 245.7 Hz, C), 171.2 (C), 172.2 (C) ppm.

19F NMR ($CDCl_3$, 370 MHz): δ = -121.8 ppm

HRMS: ESI+, *m/z* 441.1933 [M+H]+, Error: 0.10 ppm

Synthesis of 3-((4-(piperazin-1-yl)phenyl)amino)piperidine-2,6-dione (S2.16a)

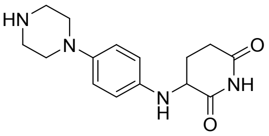

Tert-butyl 4-(4-((2,6-dioxopiperidin-3-yl)amino)phenyl)piperazine-1-carboxylate was dissolved in a mixture of DCM/TFA (2/1) and stirred for 1 to 2h. Next, the solvent was evaporated and was immediately used as such in the next step.

Synthesis of 3-((6-(piperazin-1-yl)pyridin-3-yl)amino)piperidine-2,6-dione (S2.16b)

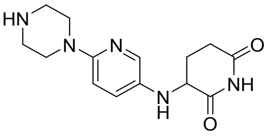

Tert-butyl 4-(5-((2,6-dioxopiperidin-3-yl)amino)pyridin-2-yl)piperazine-1-carboxylate (0.85 g, 2.2 mmol) was dissolved in 4M HCl in dioxane and stirred for 18h at room temperature. The precipitate was filtered, washed once with diethyl ether, and dried *in vacuo*. 0.79 g of the product as 2 x HCl salt was obtained in quantitative yield. The product was used as such in the next step without further characterization.

Synthesis of 3-((3-fluoro-4-(piperazin-1-yl)phenyl)amino)piperidine-2,6-dione (S2.16c)

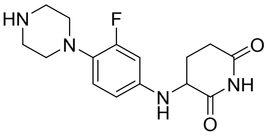

Benzyl 4-(4-((2,6-dioxopiperidin-3-yl)amino)-2-fluorophenyl)piperazine-1-carboxylate (2.9 g, 6.6 mmol) was dissolved in MeOH and Pd/C 10% was added. The reaction mixture was purged with N_2, followed by gas exchange with H_2 (2 balloons). After 18h of stirring, the reaction mixture was filtered over celite, extensively washed with MeOH and DCM, and then evaporated. This delivered the product (1.5 g) in 75% yield and was used as such in the next step without further characterization.

HRMS: ESI$^+$, *m/z* 307.1564 [M+H]$^+$, Error: 0.25 ppm

Synthesis of tert-butyl (2-(4-(4-((2,6-dioxopiperidin-3-yl)amino)phenyl)piperazin-1-yl)ethyl)carbamate (S2.17a)

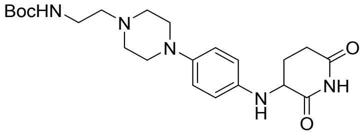

DMF (4 mL) was added to a flask with 3-((4-(piperazin-1-yl)phenyl)amino)piperidine-2,6-dione (0.77 mmol, directly from previous step, 1 eq), *tert*-butyl (2-bromoethyl)carbamate (191 mg, 0.85 mmol, 1.1 eq) and $NaHCO_3$ (650 mg, 7.7 mmol, 10 eq), and was subsequently heated to 70°C for 8h. Next, sat. $NaHCO_3$ was added, followed by extraction with DCM. The organic layer was dried over Na_2SO_4, filtered and evaporated. Purification using flash column chromatography (DCM/MeOH 0 -> 5% + 0.1% NH_4OH) delivered the intermediate (200 mg) in 60% yield.

^{1}H NMR (DMSO-d6, 400MHz): δ = 1.38 (s, 9H), 1.79-1.90 (m, 1H), 2.06-2.14 (m, 1H), 2.35 (t, J= 6.7 Hz, 2H), 2.48-2.61 (m, 5H), 2.65-2.78 (m, 1H), 2.85-2.95 (m, 4H), 3.06 (q, J= 6.2 Hz, 2H), 4.14-4.22 (m, 1H), 5.36 (d, J= 7.2 Hz, 1H), 6.60 (d, J= 8.0 Hz, 2H), 6.65 (t, J= 5.3 Hz, 1H), 6.74 (d, J= 8.8 Hz, 2H), 10.75 (s, 1H) ppm.

^{13}C NMR (DMSO-d6, 100 MHz): δ = 24.8 (CH2), 28.2 (CH3), 30.7 (CH2), 37.4 (CH2), 50.1 (CH2), 52.9 (CH2), 53.1 (CH), 57.4 (CH2), 77.8 (C), 113.5 (CH), 117.5 (CH), 141.5 (C), 142.3 (C), 155.5 (C), 173.1 (C), 173.9 (C) ppm.

HRMS: ESI$^+$, *m/z* 432.2600 [M+H]$^+$, Error: 1.22 ppm

Synthesis of tert-butyl (2-(4-(4-((2,6-dioxopiperidin-3-yl)amino)phenyl)piperazin-1-yl)-2-oxoethyl)carbamate (S2.17b)

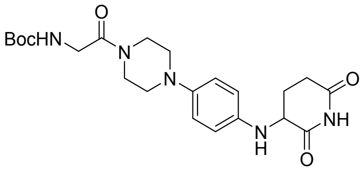

Classical amide coupling (general procedure 3) delivered the product (360 mg) in 78% yield.

^{1}H NMR ($CDCl_3$, 400MHz): δ = 1.45 (s, 9H), 1.84-1.97 (m, 1H), 2.49-2.56 (m, 1H), 2.67-2.76 (m, 1H), 2.82-2.89 (m, 1H), 2.98-3.08 (m 4H), 3.56 (t, J= 4.2 Hz, 2H), 3.79 (t, J= 4.2 Hz, 2H), 3.96-4.05 (m, 3H), 4.53 (br s, 1H), 5.53 (s, 1H), 6.66 (d, J= 8.8 Hz, 2H), 6.88 (d, J= 8.6 Hz, 2H), 8.20 (s, 1H) ppm.

^{13}C NMR ($CDCl_3$, 100 MHz): δ = 25.9 (CH2), 28.5 (CH3), 31.3 (CH2), 42.1 (CH2), 42.4 (CH2), 44.5 (CH2), 51.2 (CH2), 51.5 (CH2), 55.3 (CH), 79.9 (C), 114.9 (CH), 119.7 (CH), 141.5 (C), 156.0 (C), 167.0(C), 171.5 (C), 172.6 (C) ppm. One quaternary carbon missing.

HRMS: ESI$^+$, *m/z* 446.2393 [M+H]$^+$, Error: 1.11 ppm

Synthesis of tert-butyl (2-(2-(4-(4-((2,6-dioxopiperidin-3-yl)amino)phenyl)piperazin-1-yl)-2-oxoethoxy)ethyl)carbamate (S2.17c)

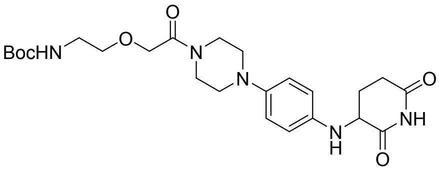

Classical HATU coupling (general procedure 3) delivered the product (190 mg) in 56% yield.

^{1}H NMR (DMSO-d6, 400MHz): δ = 1.36 (s, 9H), 1.79-1.90 (m, 1H), 2.06-2.14 (m, 1H), 2.53-2.61 (m, 1H), 2.65-2.78 (m, 1H), 2.85-2.95 (m 4H), 3.09 (q, J= 5.7 Hz, 2H), 3.43 (t, J= 5.8 Hz, 2H), 3.49-3.58 (m, 4H), 4.13-4.24 (m, 3H), 5.44 (d, J= 7.3 Hz, 1H), 6.62 (d, J= 8.0 Hz, 2H), 6.78 (d, J= 8.8 Hz, 2H), 6.82 (t, J= 5.4 Hz, 1H), 10.75 (s, 1H) ppm.

^{13}C NMR (DMSO-d6, 100 MHz): δ = 24.8 (CH2), 28.2 (CH3), 30.7 (CH2), 39.9 (CH2), 41.2 (CH2), 44.4 (CH2), 50.5 (CH2), 50.9 (CH2), 52.9 (CH), 69.1 (CH2), 69.2 (CH2), 77.6 (C), 113.5 (CH), 118.5 (CH), 142.1(C), 142.5 (C), 155.5 (C), 167.3 (C), 173.1 (C), 173.8 (C) ppm.

HRMS: ESI^{+}, *m/z* 490.2664 [M+H]$^{+}$, Error: 0.80 ppm

Synthesis of tert-butyl (2-(2-(2-(4-(4-((2,6-dioxopiperidin-3-yl)amino)phenyl)piperazin-1-yl)-2-oxoethoxy)ethoxy)ethyl) carbamate (S2.17d)

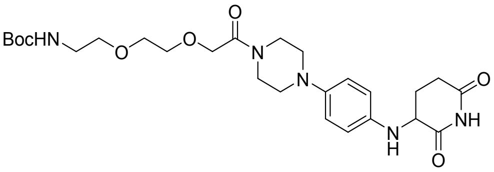

Classical HATU coupling (general procedure 3) delivered the product (410 mg) in 75% yield.

^{1}H NMR ($CDCl_3$, 400MHz): δ = 1.45 (s, 9H), 1.84-1.97 (m, 1H), 2.49-2.56 (m, 1H), 2.67-2.83 (m, 2H), 2.98-3.35 (m, 6H), 3.53 (t, J= 5.0 Hz, 2H), 3.60-4.00 (m, 8H), 4.03-4.07 (m, 1H), 4.25 (s, 2H), 5.04 (s, 1H), 6.67 (d, J= 8.0 Hz, 2H), 7.00 (br s, 2H), 8.17 (s, 1H) ppm.

^{13}C NMR ($CDCl_3$, 100 MHz): δ = 25.6 (CH2), 28.5 (CH3), 31.3 (CH2), 40.3 (CH2), 44.5 (CH2), 48.8 (CH2), 54.9 (CH), 70.2 (CH2), 70.3 (CH2), 70.6 (2x CH2), 79.3 (C), 114.9 (CH), 119.8 (CH), 141.5 (C), 156.0 (C), 168.1 (C), 171.5 (C), 172.6 (C) ppm.

One quaternary carbon missing.

HRMS: ESI^{+}, *m/z* 534.2920 [M+H]$^{+}$, Error: 0.42 ppm

Synthesis of tert-butyl 3-((4-(4-((2,6-dioxopiperidin-3-yl)amino)phenyl)piperazin-1-yl)methyl)azetidine-1-carboxylate (S2.17e)

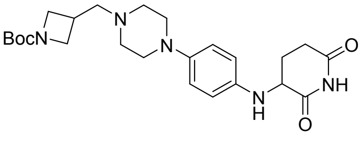

Tert-butyl 3-(hydroxymethyl)azetidine-1-carboxylate (217 mg, 1.16 mmol, 1.5 eq) was dissolved in DCM, Et_3N (0.21 mL, 1.54 mmol, 2.0 eq) added and cooled down to 0°C. Next, methanesulfonyl chloride (0.12 mL, 1.54 mmol, 2.0 eq) was added and stirred for 1h at RT. The crude was evaporated, redissolved in EA and filtered. The filtrate was collected, evaporated and redissolved with DMF. 3-((4-(piperazin-1-yl)phenyl)amino)piperidine-2,6-

dione (0.77 mmol, directly from previous step, 1 eq) and $NaHCO_3$ (650 mg, 7.7 mmol, 10 eq) were added and the RM was subsequently heated to 70°C for 2 days. Next, sat. $NaHCO_3$ was added, followed by extraction with DCM. The organic layer was dried over Na_2SO_4, filtered and evaporated. Purification using flash column chromatography (DCM/MeOH 0 -> 5% + 0.1% NH_4OH) delivered the intermediate (110 mg) in 31% yield.

^{1}H NMR ($CDCl_3$, 400MHz): δ = 1.44 (s, 9H), 1.87-1.93 (m, 1H), 2.49-2.60 (m, 5H), 2.64 (d, J= 7.3 Hz, 2H), 2.67-2.78 (m, 2H), 2.82-2.89 (m, 1H), 2.98-3.08 (m 4H), 3.61 (dd, J= 5.5; 8.6 Hz, 2H), 3.97-4.06 (m, 3H), 4.48 (br s, 1H), 6.66 (d, J= 8.8 Hz, 2H), 6.86 (d, J= 8.8 Hz, 2H), 8.06 (s, 1H) ppm.

^{13}C NMR ($CDCl_3$, 100 MHz): δ = 25.9 (CH2), 26.8 (CH), 28.5 (CH3), 31.3 (CH2), 50.7 (CH2), 53.5 (CH2), 53.6 (CH2, via HSQC), 55.6 (CH), 62.6 (CH2), 79.5 (C), 115.2 (CH), 118.7 (CH), 140.4 (C), 145.1 (C), 156.5 (C), 171.4 (C), 172.6 (C) ppm.

HRMS: ESI$^+$, *m/z* 458.2763 [M+H]$^+$, Error: 0.27 ppm

Synthesis of tert-butyl 3-(4-(4-((2,6-dioxopiperidin-3-yl)amino)-2-fluorophenyl)piperazin-1-yl)azetidine-1-carboxylate (S2.18a)

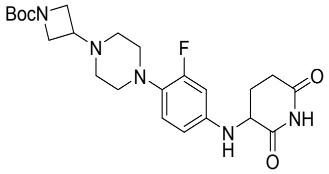

Following the general reductive amination procedure 6, the product was delivered (140 mg) with a yield of 47%.

^{1}H NMR ($CDCl_3$, 400MHz): δ = 1.43 (s, 9H), 1.87-1.93 (m, 1H), 2.48-2.56 (m, 5H), 2.70-2.80 (m, 1H), 2.84-2.90 (m, 1H), 3.02 (t, J= 4.2 Hz, 4H), 3.12-3.17 (m, 1H), 3.80-3.86 (m, 2H), 3.92-4.02 (m, 3H), 4.61 (d, J= 3.8 Hz, 1H), 6.38-6.45 (m, 2H), 6.84 (t, J= 9.7 Hz, 1H), 7.96 (s, 1H) ppm.

^{13}C NMR ($CDCl_3$, 100 MHz): δ = 25.7 (CH2), 28.5 (CH3), 31.3 (CH2), 50.0 (CH2), 55.1 (CH2), 52.2 (CH2, via HSQC), 54.0 (CH), 55.1 (CH), 79.6 (C), 102.5 (d, J= 24.2 Hz, CH), 109.3 (d, J= 2.8 Hz, CH), 120.8 (d, J= 4.3 Hz, CH), 132.2 (d, J= 9.9 Hz, C), 142.8 (d, J= 10.1 Hz, C), 156.5 (C), 157.0 (d, J= 245.7 Hz, C), 171.1 (C), 172.1 (C) ppm.

^{19}F NMR ($CDCl_3$, 370 MHz): δ = -121.7 ppm

HRMS: ESI$^+$, *m/z* 462.2505 [M+H]$^+$, Error: 1.31 ppm

Synthesis of *tert*-butyl 4-((4-(4-((2,6-dioxopiperidin-3-yl)amino)-2-fluorophenyl)piperazin-1-yl)methyl)piperidine-1-carboxylate (S2.18b)

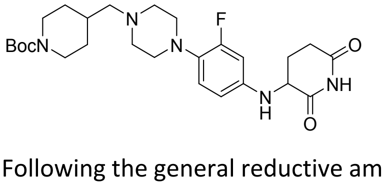

Following the general reductive amination procedure 6, the product was delivered (120 mg) with a yield of 37%.

^{1}H NMR ($CDCl_3$, 400MHz): δ = 1.08 (qd, J= 4.3; 12.5 Hz), 1.45 (s, 9H), 1.61-1.77 (m, 3H), 1.89 (qd, J= 4.7; 13.3 Hz, 1H), 2.23 (d, J= 7.0 Hz), 2.48-2.58 (m, 5H), 2.65-2.80 (m, 3H), 2.83-2.90 (m, 1H), 2.93 (t, J= 4.2 Hz, 4H), 3.99 (dt, J= 4.3; 12.2, 1H) (m, 1H), 4.08 (s, 2H), 4.61 (d, J= 3.8 Hz, 1H), 6.38-6.45 (m, 2H), 6.87 (t, J= 9.7 Hz, 1H), 7.99 (s, 1H) ppm.

^{13}C NMR ($CDCl_3$, 100 MHz): δ = 25.7 (CH2), 28.5 (CH3), 30.9 (CH2), 31.3 (CH2), 33.7 (CH), 43.7 (CH2, via HSQC), 51.4 (CH2), 54.0 (CH2), 55.2 (CH), 64.2 (CH2), 79.4 (C), 102.5 (d, J= 24.2 Hz, CH), 109.3 (d, J= 2.8 Hz, CH), 120.8 (d,

J= 4.3 Hz, CH), 132.6 (d, J= 9.9 Hz, C), 142.7 (d, J= 10.1 Hz, C), 155.0 (C), 157.0 (d, J= 245.7 Hz, C), 171.1 (C), 172.2 (C) ppm.

^{19}F NMR ($CDCl_3$, 370 MHz): δ = -121.6 ppm

HRMS: ESI$^+$, *m/z* 504.2968 $[M+H]^+$, Error: 2.49 ppm

Synthesis of *tert*-butyl 3-(4-(5-((2,6-dioxopiperidin-3-yl)amino)pyridin-2-yl)piperazin-1-yl)azetidine-1-carboxylate (S2.19)

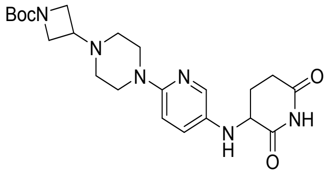

Following the general reductive amination procedure 6, the product was delivered (160 mg) with a yield of 65%.

^{1}H NMR ($CDCl_3$, 400MHz): δ = 1.43 (s, 9H), 1.92 (qd, J= 4.6; 13.3 Hz, 1H), 2.46-2.54 (m, 5H), 2.64-2.75 (m, 1H), 2.81-2.90 (m, 1H), 3.11 (tt, J= 5.4; 7.0 Hz, 1H), 3.43-3.48 (m, 4H), 3.85 (dd, J= 5.3; 8.8Hz, 2H), 3.92-4.00 (m, 3H), 4.30 (s, 1H), 6.63 (d, J= 8.9 Hz, 1H), 7.04 (dd, J= 2.9; 8.8 Hz, 1H), 7.79 (d, J= 2.9 Hz, 1H), 8.05 (s, 1H) ppm.

^{13}C NMR ($CDCl_3$, 100 MHz): δ = 25.7 (CH2), 28.5 (CH3), 31.2 (CH2), 46.2 (CH2), 49.6 (CH2), 52.5 (CH2, via HSQC), 54.05 (CH), 55.92 (CH), 80.0 (C), 108.9 (CH), 126.6 (CH), 134.7 (CH), 135.0 (C), 154.9 (C), 156.5 (C), 171.2 (C), 172.4 (C) ppm.

HRMS: ESI$^+$, *m/z* 445.2547 $[M+H]^+$, Error: 2.43 ppm

PROTAC synthesis (series 2)

Synthesis of PROTAC 12

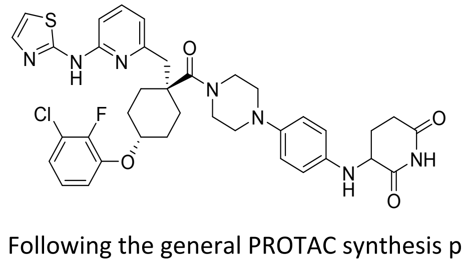

Following the general PROTAC synthesis procedure, the product was delivered (20 mg) with a yield of 27%.

^{1}H NMR (DMSO-d6, 400MHz): δ = 1.67-1.90 (m, 7H), 1.98-2.12 (m, 3H), 2.49-2.60 (m, 1H), 2.65-2.78 (m, 1H), 2.93 (s, 4H), 3.10 (s, 2H), 3.79 (s, 4H), 4.19-4.23 (m, 1H), 4.56 (s, 1H), 5.44 (d, J= 7.3 Hz, 1H), 6.63 (d, J= 8.8 Hz, 2H), 6.70 (d, J= 7.3 Hz, 1H), 6.78 (d, J= 8.8 Hz, 2H), 6.89 (d, J= 8.2 Hz, 1H), 6.98 (d, J= 3.6 Hz, 1H), 7.07-7.15 (m, 3H), 7.36 (d, J= 3.6 Hz, 1H), 7.59 (t, J= 7.8 Hz, 1H), 10.75 (s, 1H), 11.16 (s, 1H) ppm.

^{13}C NMR (DMSO-d6, 100MHz): δ = 24.8 (CH2), 26.9 (CH2), 29.6 (CH2), 30.7 (CH2), 45.0 (2x CH2), 47.2 (C), 50.7 (CH2), 53.0 (CH), 73.8 (CH), 108.3 (CH), 110.7 (CH), 113.6 (CH), 116.2 (CH), 116.5 (CH), 118.1 (CH), 120.4 (d, $^2J(C,F)$ = 15.1 Hz, C), 121.8 (CH), 124.9 (d, $^3J(C,F)$= 4.9 Hz, CH), 137.4 (CH), 137.7 (CH), 142.1 (C), 146.4 (C), 146.2 (d, $^2J(C,F)$ = 10.1 Hz, C), 148.8 (d, $^1J(C,F)$ = 246.0 Hz, C), 150.9 (C), 154.9 (C), 159.7 (C), 171.9 (C), 173.1 (C), 173.9 (C) ppm

^{19}F NMR (DMSO-d6, 370 MHz): δ = -136.4 ppm

HRMS: ESI$^+$, *m/z* 732.2518 $[M+H]^+$, Error: 1.58 ppm

Synthesis of PROTAC 13

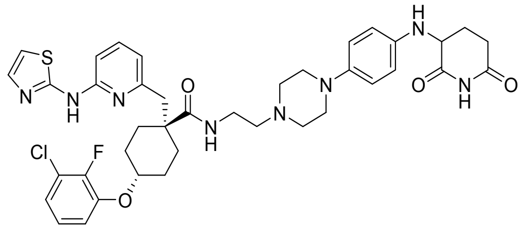

Following the general PROTAC synthesis procedure, 51 mg of the product was obtained with a yield of 67%.

^{1}H NMR (DMSO-d6, 400MHz): δ = 1.67-1.93 (m, 9H), 2.07-2.12 (m, 1H), 2.33 (t, J= 6.4 Hz, 2H), 2.46 (s, 4H), 2.57 (dt, J= 4.1; 17.5 Hz, 1H), 2.65-2.78 (m, 1H), 2.88 (s, 4H), 2.90 (s, 2H), 3.12-3.19 (m, 2H), 4.14-4.22 (m, 1H), 4.56 (s, 1H), 5.36 (d, J= 7.3 Hz, 1H), 6.60 (d, J= 8.8 Hz, 2H), 6.65 (d, J= 7.3 Hz, 1H), 6.72 (d, J= 8.8 Hz, 2H), 6.88 (d, J= 8.2 Hz, 1H), 6.97 (d, J= 3.6 Hz, 1H), 7.05-7.17 (m, 3H), 7.36 (d, J= 3.6 Hz, 1H), 7.40 (t, J= 5.0 Hz, 1H), 7.55 (t, J= 7.8 Hz, 1H), 10.75 (s, 1H), 11.13 (s, 1H) ppm.

^{13}C NMR (DMSO-d6, 100MHz): δ = 24.8 (CH2), 26.6 (CH2), 28.4 (CH2), 30.7 (CH2), 36.2 (CH2), 46.7 (C), 47.0 (CH2), 50.2 (CH2), 52.8 (CH2), 53.0 (CH), 56.6 (CH2), 74.3 (CH), 108.1 (CH), 110.7 (CH), 113.6 (CH), 116.2 (CH), 116.8 (CH), 117.6 (CH), 120.4 (d, ^{2}J(C,F) = 15.1 Hz, C), 121.8 (CH), 124.9 (d, ^{3}J(C,F)= 4.9 Hz, CH), 137.4 (CH), 137.5 (CH), 141.5 (C), 142.9 (C), 146.2 (d, ^{2}J(C,F) = 10.1 Hz, C), 148.8 (d, ^{1}J(C,F) = 246.0 Hz, C), 150.8 (C), 155.2 (C), 159.7 (C), 173.1 (C), 173.8 (C), 173.9 (C) ppm

^{19}F NMR (DMSO-d6, 377MHz): δ = -136.3 ppm

HRMS: ESI$^+$, *m/z* 775.2946 [M+H]$^+$, Error: 0.72 ppm

Synthesis of PROTAC 14

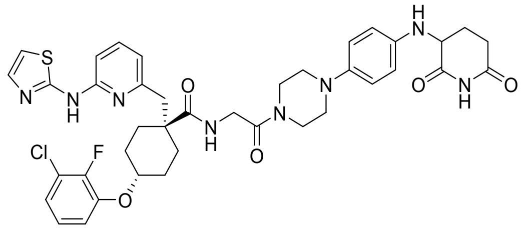

Following the general PROTAC synthesis procedure, 52 mg of the product was obtained with a yield of 66%.

^{1}H NMR (DMSO-d6, 400MHz): δ = 1.70-1.93 (m, 9H), 2.07-2.12 (m, 1H), 2.57 (dt, J= 4.1; 17.5 Hz, 1H), 2.65-2.78 (m, 1H), 2.89 (s, 4H), 2.90 (s, 2H), 3.53 (br s, 2H), 3.58 (br s, 2H), 3.95 (d, J= 5.1 Hz, 2H), 4.14-4.22 (m, 1H), 4.59 (s, 1H), 5.45 (d, J= 7.3 Hz, 1H), 6.63 (d, J= 8.8 Hz, 2H), 6.71 (d, J= 7.3 Hz, 1H), 6.79 (d, J= 8.8 Hz, 2H), 6.86 (d, J= 8.2 Hz, 1H), 6.95 (d, J= 3.6 Hz, 1H), 7.05-7.17 (m, 3H), 7.36 (d, J= 3.6 Hz, 1H), 7.54 (t, J= 7.8 Hz, 1H), 7.71 (t, J= 5.1 Hz, 1H), 10.75 (s, 1H), 11.11 (s, 1H) ppm.

^{13}C NMR (DMSO-d6, 100MHz): δ = 24.8 (CH2), 26.6 (CH2), 28.1 (CH2), 30.7 (CH2), 40.7 (CH2), 41.6 (CH2), 44.1 (CH2), 46.7 (C), 47.4 (CH2), 50.4 (CH2), 50.8 (CH2), 53.0 (CH), 74.1 (CH), 108.1 (CH), 110.7 (CH), 113.6 (CH), 116.2 (CH), 116.8 (CH), 118.5 (CH), 120.4 (d, ^{2}J(C,F) = 15.1 Hz, C), 121.8 (CH), 124.9 (d, ^{3}J(C,F)= 4.9 Hz, CH), 137.4 (CH), 137.5 (CH), 142.2 (C), 142.5 (C), 146.2 (d, ^{2}J(C,F) = 10.1 Hz, C), 148.8 (d, ^{1}J(C,F) = 246.0 Hz, C), 150.8 (C), 155.2 (C), 159.9 (C), 167.1 (C), 173.1 (C), 173.8 (C), 174.3 (C) ppm

^{19}F NMR (DMSO-d6, 377MHz): δ = -136.4 ppm

HRMS: ESI+, *m/z* 789.2731 [M+H]+, Error: 1.67 ppm

Synthesis of PROTAC 15

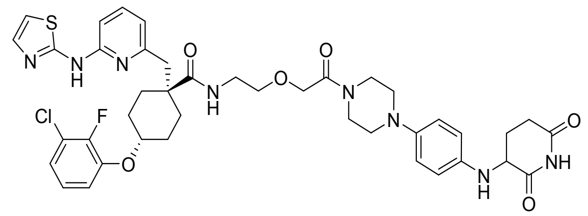

Following the general PROTAC synthesis procedure, 56 mg of the product was obtained with a yield of 67%.

^{1}H NMR (DMSO-d6, 400MHz): δ = 1.62-1.93 (m, 9H), 2.07-2.12 (m, 1H), 2.57 (dt, J= 4.1; 17.5 Hz, 1H), 2.65-2.78 (m, 1H), 2.86-2.96 (m, 6H), 3.18-3.24 (m, 2H), 3.44 (t, J= 5.9Hz, 2H), 3.48 (s, 2H), 3.54 (s, 2H), 4.16-4.22 (m, 3H), 4.54 (s, 1H), 5.45 (d, J= 7.3 Hz, 1H), 6.59-6.64 (m, 3H), 6.76 (d, J= 8.8 Hz, 2H), 6.86 (d, J= 8.2 Hz, 1H), 6.95 (d, J= 3.6 Hz, 1H), 7.05-7.17 (m, 3H), 7.36 (d, J= 3.6 Hz, 1H), 7.54 (t, J= 7.8 Hz, 1H), 7.81 (t, J= 5.1 Hz, 1H), 10.75 (s, 1H), 11.11 (s, 1H) ppm.

^{13}C NMR (DMSO-d6, 100MHz): δ = 24.8 (CH2), 26.6 (CH2), 28.3 (CH2), 30.7 (CH2), 38.9 (CH2), 41.2 (CH2), 44.2 (CH2), 46.6 (C), 46.8 (CH2), 50.4 (CH2), 50.8 (CH2), 53.0 (CH), 68.8 (CH2), 69.0 (CH2), 74.4 (CH), 108.1 (CH), 110.7 (CH), 113.6 (CH), 116.2 (CH), 116.8 (CH), 118.5 (CH), 120.4 (d, ^{2}J(C,F) = 15.1 Hz, C), 121.8 (CH), 124.9 (d, ^{3}J(C,F)= 4.9 Hz, CH), 137.4 (CH), 137.5 (CH), 142.2 (C), 142.5 (C), 146.2 (d, ^{2}J(C,F) = 10.1 Hz, C), 148.8 (d, ^{1}J(C,F) = 246.0 Hz, C), 150.8 (C), 155.2 (C), 159.9 (C), 167.5 (C), 173.1 (C), 173.8 (C), 174.2 (C) ppm

^{19}F NMR (DMSO-d6, 377MHz): δ = -136.3 ppm

HRMS: ESI+, *m/z* 833.3006 [M+H]+, Error: 0.04 ppm

Synthesis of PROTAC 16

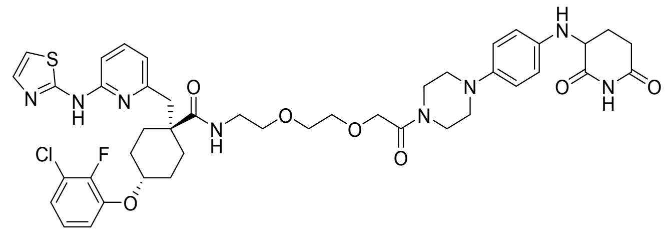

Following the general PROTAC synthesis procedure, 33 mg of the product was obtained with a yield of 38%.

^{1}H NMR (DMSO-d6, 400MHz): δ = 1.63-1.93 (m, 9H), 2.07-2.12 (m, 1H), 2.57 (dt, J= 4.1; 17.5 Hz, 1H), 2.65-2.78 (m, 1H), 2.89 (s, 4H), 2.90 (s, 2H), 3.18-3.23 (m, 2H), 3.39 (t, J= 5.9 Hz, 2H), 3.45-3.58 (m, 8H), 4.14-4.22 (m, 3H), 4.54 (s, 1H), 5.44 (d, J= 7.3 Hz, 1H), 6.59-6.64 (m, 3H), 6.76 (d, J= 8.8 Hz, 2H), 6.86 (d, J= 8.2 Hz, 1H), 6.95 (d, J= 3.6 Hz, 1H), 7.05-7.17 (m, 3H), 7.36 (d, J= 3.6 Hz, 1H), 7.54 (t, J= 7.8 Hz, 1H), 7.63 (t, J= 5.1 Hz, 1H), 10.76 (s, 1H), 11.12 (s, 1H) ppm.

^{13}C NMR (DMSO-d6, 100MHz): δ = 24.8 (CH2), 26.6 (CH2), 28.3 (CH2), 30.7 (CH2), 38.9 (CH2), 41.2 (CH2), 44.2 (CH2), 46.6 (C), 46.8 (CH2), 50.4 (CH2), 50.8 (CH2), 53.0 (CH), 68.8 (CH2), 69.3 (CH2), 69.4 (CH2), 69.8 (CH2), 74.4 (CH), 108.1 (CH), 110.7 (CH), 113.6 (CH), 116.2 (CH), 116.8 (CH), 118.5 (CH), 120.4 (d, ^{2}J(C,F) = 15.1 Hz, C), 121.8 (CH), 124.9 (d, ^{3}J(C,F)= 4.9 Hz, CH), 137.4 (CH), 137.5 (CH), 142.2 (C), 142.5 (C), 146.2 (d, ^{2}J(C,F) = 10.1 Hz, C), 148.8 (d, ^{1}J(C,F) = 246.0 Hz, C), 150.8 (C), 155.2 (C), 159.9 (C), 167.2 (C), 173.1 (C), 173.8 (C), 174.1 (C) ppm

^{19}F NMR (DMSO-d6, 377MHz): δ = -136.4 ppm

HRMS: ESI+, *m/z* 877.3261 [M+H]+, Error: 0.85 ppm

Synthesis of PROTAC 17

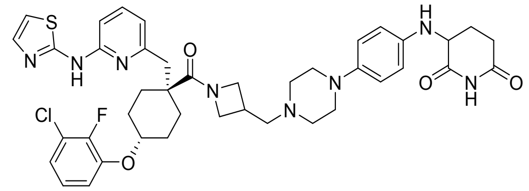

Following the general PROTAC synthesis procedure, 52 mg of the product was obtained with a yield of 65%.

^{1}H NMR (DMSO-d6, 400MHz): δ = 1.62-1.93 (m, 9H), 2.07-2.12 (m, 1H), 2.23-2.39 (m, 6H), 2.50-2.62 (m, 2H), 2.65-2.78 (m, 1H), 2.86-2.96 (m, 6H), 3.12-3.16 (m, 1H), 3.32-3.36 (m, 1H), 3.76-3.88 (m, 2H), 4.16-4.21 (m, 1H), 4.58 (s, 1H), 5.37 (d, J= 7.3 Hz, 1H), 6.60 (d, J=8.7 Hz, 2H), 6.68-6.74 (m, 3H), 6.93-7.00 (m, 2H), 7.10-7.21 (m, 3H), 7.36 (d, J= 3.6 Hz, 1H), 7.65 (t, J= 7.8 Hz, 1H), 10.75 (s, 1H), 11.23 (s, 1H) ppm.

^{13}C NMR (DMSO-d6, 100MHz): δ = 24.8 (CH2), 25.8 (CH), 26.8 (CH2), 27.9 (CH2), 29.7 (CH2), 30.7 (CH2), 46.7 (CH2), 47.5 (C), 50.1 (CH2), 52.7 (CH2), 52.8 (CH2), 53.0 (CH), 56.7 (CH2), 61.5 (CH2), 74.2 (CH), 108.1 (CH), 110.7 (CH), 113.6 (CH), 116.2 (CH), 116.8 (CH), 117.7 (CH), 120.4 (d, ^{2}J(C,F) = 15.1 Hz, C), 121.9 (CH), 125.0 (d, ^{3}J(C,F)= 4.9 Hz, CH), 137.4 (CH), 137.9 (CH), 141.6 (C), 142.9 (C), 146.2 (d, ^{2}J(C,F) = 10.1 Hz, C), 148.8 (d, ^{1}J(C,F) = 246.0 Hz, C), 151.0 (C), 155.2 (C), 159.9 (C), 173.1 (C), 173.1 (C), 173.9 (C) ppm

^{19}F NMR (DMSO-d6, 377MHz): δ = -136.3 ppm

HRMS: ESI+, *m/z* 801.3101 [M+H]+, Error: 0.88 ppm

Synthesis of PROTAC 18

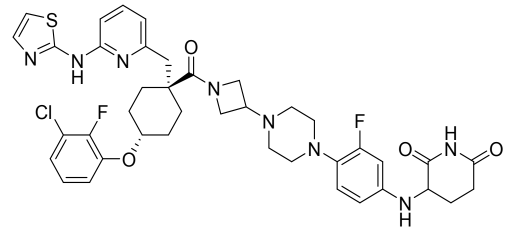

Following the general PROTAC synthesis procedure, 52 mg of the product was obtained with a yield of 65%.

^{1}H NMR (DMSO-d6, 400MHz): δ = 1.59-1.93 (m, 9H), 2.05-2.11 (m, 1H), 2.29 (br s, 4H), 2.50-2.60 (m, 1H), 2.67-2.78 (m, 1H), 2.83-2.91 (m, 7H), 3.62 (br s, 2H), 3.76-3.85 (m, 2H), 4.16-4.21 (m, 1H), 4.58 (s, 1H), 5.81 (d, J= 7.3 Hz, 1H), 6.43 (dd, J= 2.0; 8.7 Hz, 1H), 6.50 (dd, J= 2.2; 15.0 Hz, 1H), 6.71 (d, J=7.3 Hz, 1H), 6.84 (t, J= 9.4Hz, 1H), 6.93 (d, J= 8.2 Hz, 1H), 6.99 (d, J= 3.6 Hz, 1H), 7.10-7.21 (m, 3H), 7.36 (d, J= 3.6 Hz, 1H), 7.62 (t, J= 7.8 Hz, 1H), 10.78 (s, 1H), 11.17 (s, 1H) ppm.

^{13}C NMR (DMSO-d6, 100MHz): δ = 24.6 (CH2), 26.7 (CH2), 28.6 (CH2), 30.7 (CH2), 46.3 (CH2), 47.5 (C), 49.2 (CH2), 50.7 (CH2), 52.5 (CH2), 52.7 (CH), 53.3 (CH), 56.0 (CH2), 74.2 (CH), 100.8 (d, J= 24.4, CH), 108.3 (CH), 108.4 (CH), 110.7 (CH), 116.2 (CH), 116.8 (CH), 120.5 (CH), 120.5 (d, ^{2}J(C,F) = 15.1 Hz, C), 121.9 (CH), 125.0 (d, ^{3}J(C,F)= 4.9 Hz, CH), 129.6 (d, J= 9.1 Hz, C), 137.4 (CH), 137.9 (CH), 144.7 (d, J= 10.2 Hz, C), 146.2 (d, ^{2}J(C,F) = 10.1 Hz, C), 148.8 (d, ^{1}J(C,F) = 246.0 Hz, C), 151.0 (C), 154.9 (C), 156.2 (d, ^{1}J= 242.0 Hz, C), 159.8 (C), 173.1 (C), 173.1 (C), 173.6 (C) ppm

^{19}F NMR (DMSO-d6, 377MHz): δ = -136.2 ppm

HRMS: ESI+, *m/z* 805.2855 [M+H]+, Error: 0.29 ppm

Synthesis of PROTAC 19

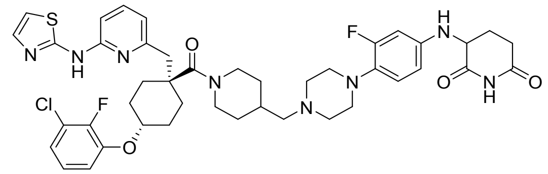

Following the general PROTAC synthesis procedure, 28 mg of the product was obtained with a yield of 33%.

^{1}H NMR (DMSO-d6, 400MHz): δ = 0.91-1.06 (m, 2H) 1.65-1.90 (m, 10H), 1.93-2.00 (m, 2H), 2.04-2.10 (m, 1H), 2.16-2.20 (m, 2H), 2.48-2.60 (m, 5H, overlap with solvent signal), 2.67-2.99 (m, 7H), 3.06 (s, 2H), 4.19-4.29 (m, 1H), 4.45 (d, J= 12.5 Hz, 2H), 4.55 (s, 1H), 5.80 (d, J= 7.3 Hz, 1H), 6.43 (dd, J= 2.0; 8.7 Hz, 1H), 6.50 (dd, J= 2.2; 15.0 Hz, 1H), 6.67 (d, J=7.3 Hz, 1H), 6.84 (t, J= 9.4Hz, 1H), 6.87 (d, J= 8.2 Hz, 1H), 6.98 (d, J= 3.6 Hz, 1H), 7.06-7.13 (m, 3H), 7.36 (d, J= 3.6 Hz, 1H), 7.58 (t, J= 7.8 Hz, 1H), 10.78 (s, 1H), 11.16 (s, 1H) ppm.

^{13}C NMR (DMSO-d6, 100MHz): δ = 24.6 (CH2), 27.0 (CH2), 29.5 (CH2), 30.7 (2x CH2), 32.9 (CH), 44.9 (CH2), 47.1 (C), 51.2 (CH2), 52.7 (CH), 53.4 (CH2), 63.8 (CH2), 73.8 (CH), 100.8 (d, J= 24.4, CH), 108.3 (CH), 108.4 (CH), 110.7 (CH), 116.2 (CH), 116.5 (CH), 120.3 (CH), 120.5 (C), 121.7 (CH), 124.9 (d, ^{3}J(C,F)= 4.9 Hz, CH), 129.8 (C), 137.4 (CH), 137.7 (CH), 144.7 (d, J= 10.2 Hz, C), 146.2 (d, ^{2}J(C,F) = 10.1 Hz, C), 148.8 (d, ^{1}J(C,F) = 246.0 Hz, C), 151.0 (C), 154.9 (C), 156.2 (d, ^{1}J= 242.0 Hz, C), 159.7 (C), 171.5 (C), 173.1 (C), 173.6 (C) ppm. One CH2 missing from piperidine, probably due to peak broadening.

^{19}F NMR (DMSO-d6, 377MHz): δ = -136.2 ppm

HRMS: ESI$^+$, *m/z* 847.3331 [M+H]$^+$, Error: 0.49 ppm

Synthesis of PROTAC 20

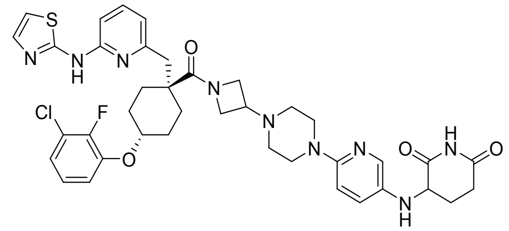

Following the general PROTAC synthesis procedure, 54 mg of the product was obtained with a yield of 69%.

^{1}H NMR (DMSO-d6, 400MHz): δ = 1.59-1.96 (m, 9H), 2.07-2.14 (m, 1H), 2.25 (br s, 4H), 2.50-2.60 (m, 1H), 2.67-2.78 (m, 1H), 2.83-2.91 (m, 3H), 3.25 (s, 4H), 3.55-3.63 (m, 2H), 3.76-3.82 (m, 2H), 4.18-4.23 (m, 1H), 4.58 (s, 1H), 5.42 (d, J= 7.1 Hz, 1H), 6.66-6.73 (m, 2H), 6.91 (d, J= 8.2 Hz, 1H), 6.98 (d, J= 3.5 Hz, 1H), 7.07 (dd, J= 2.5; 8.9 Hz, 1H), 7.08-7.21 (m, 3H), 7.36 (d, J= 3.6 Hz, 1H), 7.61 (t, J= 7.8 Hz, 1H), 7.70 (d, J= 2.4 Hz, 1H), 10.76 (s, 1H), 11.17 (s, 1H) ppm.

^{13}C NMR (DMSO-d6, 100MHz): δ = 24.7 (CH2), 26.7 (CH2), 28.6 (CH2), 28.8 (CH2), 30.7 (CH2), 45.8 (CH2), 46.4 (CH2), 47.5 (C), 48.9 (CH2), 52.5 (CH2), 53.0 (CH), 53.3 (CH), 56.0 (CH2), 74.2 (CH), 108.2 (CH), 108.3 (CH), 110.7 (CH), 116.2 (CH), 116.8 (CH), 120.5 (d, ^{2}J(C,F) = 15.1 Hz, C), 121.9 (CH), 123.7 (CH), 125.0 (d, ^{3}J(C,F)= 4.9 Hz, CH), 132.6 (CH), 136.9 (C), 137.4 (CH), 137.9 (CH), 146.2 (d, ^{2}J(C,F) = 10.1 Hz, C), 148.8 (d, ^{1}J(C,F) = 246.0 Hz, C), 151.0 (C), 152.6 (C), 154.9 (C), 159.7 (C), 173.1 (C), 173.1 (C), 173.8 (C) ppm

^{19}F NMR (DMSO-d6, 377MHz): δ = -136.2 ppm

HRMS: ESI$^+$, *m/z* 788.2903 [M+H]$^+$, Error: 0.13 ppm

SYNTHESIS OF SERIES 3 PROTACS

(DIHYDROURACIL-BASED PROTACS)

Synthesis of 1-(pyridin-3-yl-methyl)dihydrouracil-based CRBN intermediates

Scheme S5. *Synthesis of 1-(pyridin-3-yl-methyl)dihydrouracil-based PROTACs*

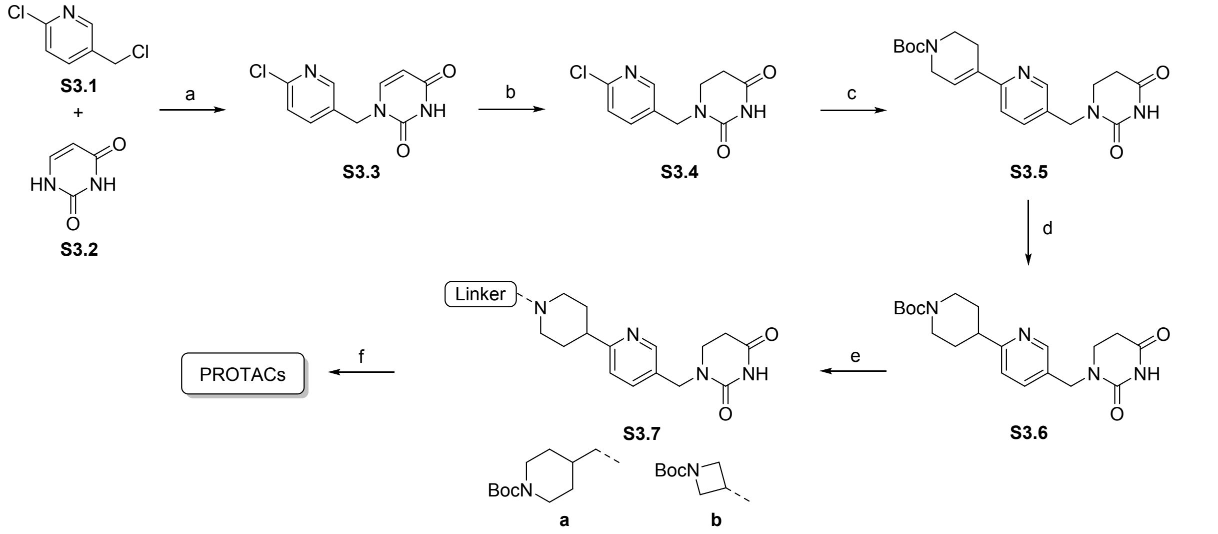

Reagents and conditions: ***a)*** *K_2CO_3, NaI, DMF, 70 °C, 19%;* ***b)*** *L-selectride, THF, 0 °C, 80%;* ***c)*** *appropriate pinacol ester, Na_2CO_3, $Pd(PPh_3)_4$, dioxane, water, 86%;* ***d)*** *H_2, Pd/C 10%, AcOH, EA, 84%;* ***e)*** *i.4M HCl, dioxane, ii. appropriate aldehyde or ketone, $NaBH(OAc)_3$, $NaHCO_3$, DMF, 47 % (a), 43% (b);* ***f)*** *i. TFA, DCM, ii. MK-5108, HATU, DIPEA, DMF, 45% (a), 69% (b).*

Synthesis of 1-((6-chloropyridin-3-yl)methyl)pyrimidine-2,4(1H, 3H)-dione (S3.3)

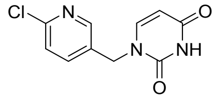

DMF (100 mL) was added to a flask with 2-chloro-5-(chloromethyl)pyridine (5g, 30.9 mmol, 1 eq), uracil (8.7 g, 77.2 mmol, 2.5 eq), K_2CO_3 (10.7 g, 77.2 mmol, 2.5 eq) and NaI (0.46 g, 3.1 mmol, 0.1 eq). The reaction was heated to 70°C for 4 h. Next, water was added, cooled down to 0°C and subsequently acidified with 0.1 M HCl up to pH 7. The mixture was extracted with EA and purified via flash column chromatography (DCM/MeOH 0 -> 5%). The product was redissolved in DCM heated to 50°C and slowly cooled down to 0°C. The precipitate was filtered and delivered the product (1.4 g) in 19% yield after drying in vacuo.

^{1}H NMR (DMSO-d6, 400MHz): δ = 4.89 (s, 2H), 5.61 (d, J= 7.9 Hz, 1H), 7.51 (d, J= 8.2 Hz, 1H), 7.78-7.84 (m, 2H), 8.41 (d, J= 2.4 Hz, 1H), 11.35 (s, 1H) ppm.

^{13}C NMR (DMSO-d6, 100 MHz): δ = 47.6 (CH2), 99.7 (CH), 124.3 (CH), 132.1 (C), 139.2 (CH), 145.4 (CH), 149.4 (CH), 149.6 (C), 151.0 (C), 163.6 (C) ppm.

Synthesis of 1-((6-chloropyridin-3-yl)methyl)dihydropyrimidine-2,4(1H, 3H)-dione (S3.4)

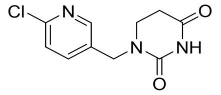

1-((6-chloropyridin-3-yl)methyl)pyrimidine-2,4(1H, 3H)-dione (800 mg, 3.36 mmol, 1.0 eq) was dissolved in THF and subsequently cooled down to 0°C. 1M L-selectride in THF (6.7 mL, 6.73 mmol, 2.0 eq) was slowly added and

stirred for 30 minutes at 0°C. Next, the reaction was quenched with sat. NH_4Cl and extracted with EA. The organic layers were dried over Na_2SO_4, filtered and evaporated. Flash column chromatography delivered the product (640 mg) in 80% yield.

^{1}H NMR (DMSO-d6, 400MHz): δ = 2.56 (t, J= 6.8 Hz, 2H), 3.35 (t, J= 6.8 Hz, 2H), 4.53 (s, 2H), 7.51 (d, J= 8.2 Hz, 1H), 7.78 (dd, J= 2.5; 8.2 Hz, 1H), 8.37 (d, J= 2.4 Hz, 1H), 10.24 (s, 1H) ppm.

^{13}C NMR (DMSO-d6, 100 MHz): δ = 30.7 (CH2), 41.4 (CH2), 46.6 (CH2), 124.3 (CH), 132.7(C), 139.2 (CH), 149.2 (CH), 149.2 (C), 153.2 (C), 170.5 (C) ppm.

Synthesis of *tert*-butyl 5-((2,4-dioxotetrahydropyrimidin-1(2H)-yl)methyl)-3',6'-dihydro-[2,4'-bipyridine]-1'(2'H)-carboxylate (S3.5)

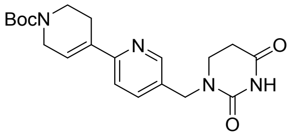

Dioxane (10 mL) and water (1 mL) were added to a flask with 1-((6-chloropyridin-3-yl)methyl)dihydropyrimidine-2,4(1H, 3H)-dione (360 mg, 1.5 mmol, 1.0 eq), *tert*-butyl 4-(4,4,5,5-tetramethyl-1,3,2-dioxaborolan-2-yl)-3,6-dihydropyridine-1(2H)-carboxylate (510 mg, 1.65 mmol, 1.1 eq) and Na_2CO_3 (477 mg, 4.5 mmol, 3.0 eq). The mixture was purged with argon for 5 minutes, followed by addition of $Pd(PPh_3)_4$ (87 mg, 0.075 mmol, 0.05 eq) and heating to 100°C for 6h. Brine was added and the mixture was extracted with EA. The organic layers were dried over Na_2SO_4, filtered and evaporated. Flash column chromatography delivered the product (500 mg) in 86% yield.

^{1}H NMR ($CDCl_3$, 400MHz): δ = 1.49 (s, 9H), 2.62-2.68 (m, 4H), 3.35 (t, J= 6.8 Hz, 2H), 3.64 (t, J= 5.4 Hz, 2H), 4.14 (d, J= 2.5 Hz, 2H), 4.62 (s, 2H), 6.60-6.63 (m, 1H), 7.38 (d, J= 8.2 Hz, 1H), 7.65 (dd, J= 2.2; 8.2 Hz, 1H), 7.79 (s, 1H), 8.49 (d, J= 2.1 Hz, 1H) ppm.

^{13}C NMR ($CDCl_3$, 100 MHz): δ = 26.0 (CH2), 28.6 (CH3), 31.1 (CH2), 39.7 (CH2, via HSQC), 41.6 (CH2), 43.8 (CH2), 48.2 (CH2), 79.9 (C), 119.4 (CH), 125.2 (CH), 130.0 (C), 135.0 (C), 136.7 (CH), 148.8 (CH), 152.9 (C), 155.0 (C), 157.3 (C), 169.3 (C) ppm.

HRMS: ESI$^+$, *m/z* 387.2019 [M+H]$^+$, Error: 2.03 ppm

Synthesis of *tert*-butyl 4-(5-((2,4-dioxotetrahydropyrimidin-1(2H)-yl)methyl)pyridin-2-yl)piperidine-1-carboxylate (S3.6)

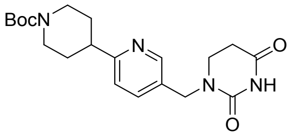

Tert-butyl 5-((2,4-dioxotetrahydropyrimidin-1(2H)-yl)methyl)-3',6'-dihydro-[2,4'-bipyridine]-1'(2'H)-carboxylate (500 mg, 1.29 mmol, 1 eq) was dissolved in EA, followed by addition of AcOH (73 µL, 1.29 mmol, 1eq) and Pd/C 10%. The reaction mixture was purged with N_2, followed by gas exchange with H_2 (2 balloons). After 18h of stirring, the reaction mixture was filtered over celite and washed with sat. $NaHCO_3$. This delivered the product (420 mg) in 84% yield after purification (DCM/MeOH 0 -> 5%).

^{1}H NMR ($CDCl_3$, 400MHz): δ = 1.47 (s, 9H), 1.63-1.76 (m, 2H), 1.86-1.92 (m, 2H), 2.65 (t, J= 6.8 Hz, 2H), 2.76-2.89 (m, 3H), 3.36 (t, J= 6.8 Hz, 2H), 4.25 (br s, 2H), 4.60 (s, 2H), 7.16 (d, J= 8.0 Hz, 1H), 7.62 (dd, J= 2.2; 8.2 Hz, 1H), 7.85 (s, 1H), 8.49 (d, J= 2.1 Hz, 1H) ppm.

^{13}C NMR ($CDCl_3$, 100 MHz): δ = 28.6 (CH3), 31.1 (CH2), 31.8 (CH2), 41.6 (CH2), 44.2 (CH2), 44.4 (CH), 48.2 (CH2), 79.6 (C), 121.4 (CH), 130.0 (C), 136.8 (CH), 148.9 (CH), 152.9 (C), 154.9 (C), 164.7 (C), 169.3 (C) ppm.

Synthesis of 1-((6-(piperidin-4-yl)pyridin-3-yl)methyl)dihydropyrimidine-2,4(1H,3H)-dione

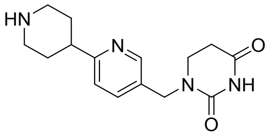

The Boc-protected intermediate (420 mg, 1 eq) was treated with 4M HCl in dioxane (5 mL) for 5h at RT. Then, diethyl ether was added and the solids filtered. The intermediate (320 mg) was obtained as HCl salt in 90% yield after drying *in vacuo*.

^{1}H NMR (DMSO-d6, 400MHz): δ = 2.00-2.17 (m, 4H), 2.59 (t, J= 6.8 Hz, 2H), 2.95-3.06 (m, 2H), 3.35-3.52 (m, 5H), 4.65 (s, 2H), 7.78 (d, J= 8.2 Hz, 1H), 8.34 (d, J= 7.9 Hz, 1H), 8.72 (s, 1H), 9.30 (s, 2H), 10.29 (s, 1H) ppm.

^{13}C NMR (DMSO-d6, 100 MHz): δ = 27.2 (CH2), 30.7 (CH2), 37.4 (CH), 41.8 (CH2), 42.6 (CH2), 46.8 (CH2), 123.8 (CH), 134.9 (C), 142.1 (CH), 143.8 (CH), 153.4 (C), 157.4 (C), 170.6 (C) ppm.

Synthesis of *tert*-butyl 4-((4-(5-((2,4-dioxotetrahydropyrimidin-1(2H)-yl)methyl)pyridin-2-yl)piperidin-1-yl)methyl) piperidine-1-carboxylate (S3.7a)

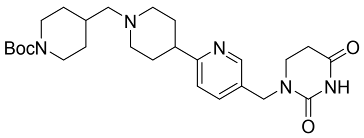

Following the general reductive amination procedure 6, the product was delivered (120 mg) with a yield of 47%.

^{1}H NMR ($CDCl_3$, 400MHz): δ = 1.09 (qd, J= 3.6; 12.4 Hz, 2H), 1.45 (s, 9H), 1.63-1.76 (m, 3H), 1.78-1.92 (m, 4H), 2.04 (td, J= 2.1; 11.5 Hz, 2H), 2.12 (d, J= 7.0 Hz, 2H), 2.62-2.73 (m, 5H), 2.94-3.01 (m, 2H), 3.36 (t, J= 6.8 Hz, 2H), 4.08 (br s, 2H), 4.59 (s, 2H), 7.19 (d, J= 8.0 Hz, 1H), 7.53 (s, 1H), 7.61 (dd, J= 2.2; 8.2 Hz, 1H), 8.45 (d, J= 2.1 Hz, 1H) ppm.

^{13}C NMR ($CDCl_3$, 100 MHz): δ = 28.6 (CH3), 31.0 (CH2), 31.1 (CH2), 32.1 (CH2), 34.0 (CH), 41.6 (CH2), 44.3 (CH2, via HSQC), 44.6 (CH), 48.2 (CH2), 54.7 (CH2), 65.0 (CH2), 79.4 (C), 121.4 (CH), 129.3 (C), 136.8 (CH), 148.9 (CH), 152.7 (C), 155.0 (C), 165.6 (C), 169.3 (C) ppm.

HRMS: ESI^{+}, *m/z* 486.3076 $[M+H]^{+}$, Error: 0.26 ppm

Synthesis of *tert*-butyl 3-(4-(5-((2,4-dioxotetrahydropyrimidin-1(2H)-yl)methyl)pyridin-2-yl)piperidin-1-yl)azetidine-1-carboxylate (S3.7b)

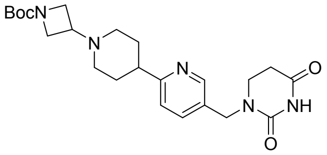

Following the general reductive amination procedure 6, the product was delivered (100 mg) with a yield of 43%.

^{1}H NMR (DMSO-d6, 400MHz): δ = 1.38 (s, 9H), 1.63-1.76 (m, 2H), 1.78-1.92 (m, 4H), 2.55 (t, J= 6.8 Hz, 2H), 2.60-2.70 (m, 1H), 2.82-2.89 (m, 2H), 3.01-3.06 (m, 1H), 3.33 (m, 2H), 3.66 (br s, 2H), 3.84 (br s, 2H), 4.59 (s, 2H), 7.25 (d, J= 8.0 Hz, 1H), 7.62 (dd, J= 2.2; 8.2 Hz, 1H), 8.43 (d, J= 2.1 Hz, 1H), 10.21 (s, 1H) ppm.

^{13}C NMR (DMSO-d6, 100 MHz): δ = 28.1 (CH3), 30.7 (CH2), 31.1 (CH2), 41.4 (CH2), 43.2 (CH), 47.2 (CH2), 49.6 (CH2), 52.6 (CH2), 53.5 (CH), 53.8 (CH2), 78.5 (C), 120.9 (CH), 130.4 (C), 135.9 (CH), 148.2 (CH), 153.1 (C), 155.6 (C), 163.5 (C), 170.5 (C) ppm.

HRMS: ESI$^+$, *m/z* 444.2605 [M+H]$^+$, Error: 0.06 ppm

Synthesis of 1-(6-(piperazin-1-yl)pyridin-3-yl)dihydrouracil- and 1-(6-(piperidin-4-yl)pyridin-3-yl)-based CRBN intermediates.

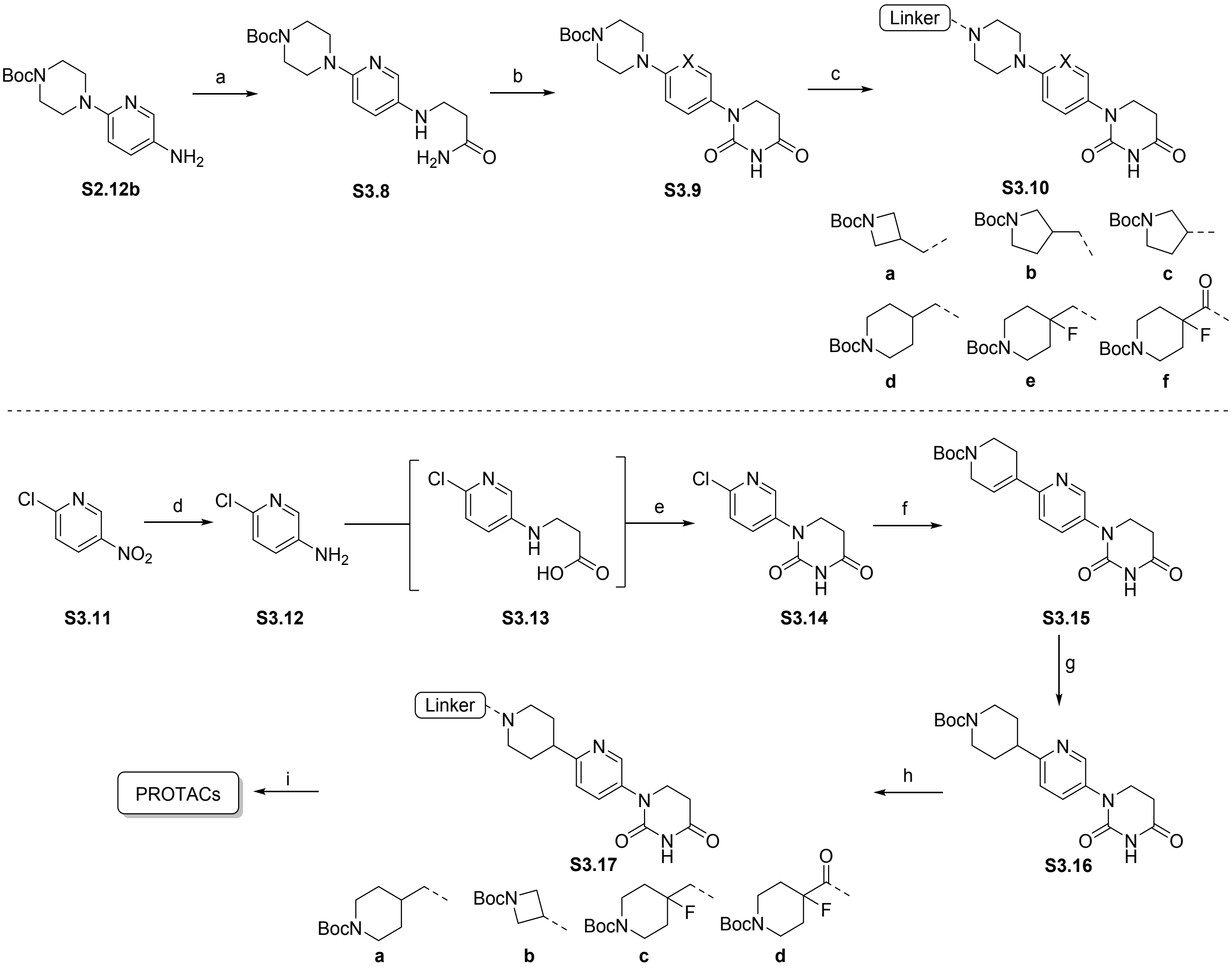

Reagents and conditions: ***a)*** *Acrylamide, toluene, 100 °C, 30%;* ***b)*** *4-nitrophenyl chloroformate, K_2CO_3, DMF, 100 °C, 58%;* ***c)*** *i. 4M HCl in dioxane, 94%, ii. Appropriate aldehyde or ketone, $NaBH(OAc)_3$, $NaHCO_3$, DMF, 49-80% or for (f) HATU, DIPEA, DMF, 55%;* ***d)*** *$SnCl_2$, EtOH, 80 °C, 69%;* ***e)*** *i. Acrylic acid, toluene, 100°C, ii. Ureum, AcOH, 100°C, 22% over 2 steps* ***f)*** *the appropriate pinacol ester, Na_2CO_3, $Pd(PPh_3)_4$, dioxane, water, 53%;* ***g)*** *H_2, $Pd(OH)_2/C$, AcOH, 79%;* ***h)*** *i. 4M HCl in dioxane, 94% ii. appropriate aldehyde or ketone, $NaBH(OAc)_3$, $NaHCO_3$, DMF, 89% (a), 80% (b), 39 % (c) or for (d): appropriate acid, HATU, DIPEA, DMF, 41%* ***i)*** *i. TFA, DCM, ii. MK-5108, HATU, DIPEA, DMF.*

Synthesis of *tert*-butyl 4-(5-((3-amino-3-oxopropyl)amino)pyridin-2-yl)piperazine-1-carboxylate (S3.8)

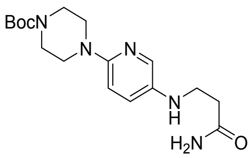

The aniline (15.1 g, 54.25 mmol, 1 eq) was dissolved in toluene and acrylamide (4.63 g, 65.1 mmol, 1.2 eq) was subsequently added. 24h and 48h later, extra acrylamide (1 eq) was added. After 72h of stirring, the reaction was evaporated on celite and purified via flash column chromatography (DCM/MeOH 0 -> 8%). 5.7 g of the product was obtained with a yield of 30%.

<u>^{1}H NMR ($CDCl_3$, 400MHz):</u> δ = 1.47 (s, 9H), 2.50 (t, J= 6.1 Hz, 2H), 3.32 (t, J= 5.0 Hz, 4H), 3.39 (t, J= 6.0 Hz, 2H), 3.56 (t, J= 5.2 Hz, 4H), 3.65 (br s, 1H), 5.57 (br s, 1H) 5.82 (br s, 1H), 6.60 (d, J= 8.9 Hz, 1H), 6.97 (dd, J= 3.0; 8.8 Hz, 1H), 7.74 (d, J= 2.9 Hz, 1H) ppm.

<u>^{13}C NMR ($CDCl_3$, 100 MHz):</u> δ = 28.6 (CH3), 34.8 (CH2), 41.1 (CH2), 43.4 (CH2, via HSQC), 46.9 (CH2), 79.9 (C), 109.1 (CH), 125.0 (CH), 134.1 (CH), 137.0 (C), 154.1 (C), 154.9 (C), 174.0 (C) ppm.

Synthesis of *tert*-butyl 4-(5-(2,4-dioxotetrahydropyrimidin-1(2H)-yl)pyridin-2-yl)piperazine-1-carboxylate (S3.9)

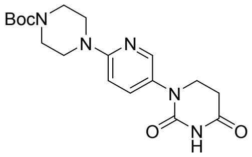

Tert-butyl 4-(5-((3-amino-3-oxopropyl)amino)pyridin-2-yl)piperazine-1-carboxylate (5.6 g, 16.0 mmol, 1.0 eq) was dissolved in DMF, followed by the addition of K_2CO_3 (5.5 g, 40.1 mmol, 2.5 eq) and 4-nitrophenyl chloroformate (4.2 g, 20.8 mmol, 1.3 eq). After stirring at 100 °C for 5 h, sat. NaHCO3 was added and extracted with DCM. Purification with flash column chromatography (DCM/MeOH 0 -> 5%) delivered the product (3.5 g) in 58% yield.

<u>^{1}H NMR ($CDCl_3$, 400MHz):</u> δ = 1.48 (s, 9H), 2.82 (t, J= 6.7 Hz, 2H), 3.53 (s, 8H), 3.79 (t, J= 6.7 Hz, 2H), 6.65 (d, J= 9.0 Hz, 1H), 7.45 (dd, J= 2.8; 9.0 Hz, 1H), 7.84 (s, 1H), 8.10 (d, J= 2.7 Hz, 1H) ppm.

<u>^{13}C NMR ($CDCl_3$, 100 MHz):</u> δ = 28.5 (CH3), 31.5 (CH2), 43.4 (CH2, via HSQC), 45.2 (CH2), 45.7 (CH2), 80.2 (C), 107.1 (CH), 128.4 (C), 135.4 (CH), 144.8 (CH), 152.2 (C), 154.9 (C), 157.9 (C), 169.5 (C) ppm.

<u>HRMS</u>: ESI$^+$, *m/z* 376.1964 $[M+H]^+$, Error: 4.07 ppm

Synthesis of 1-(6-(piperazin-1-yl)pyridin-3-yl)dihydropyrimidine-2,4(1H,3H)-dione

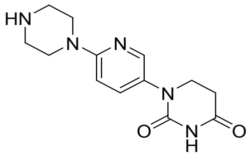

The Boc-protected intermediate (3.5 g, 9.32 mmol), was dissolved in 4M HCl in dioxane (10 mL) and stirred for 16h. The solids were filtered, washed once with diethyl ether and this delivered the product (3.0 g) as HCl salt in 94% yield after drying *in vacuo*. The product was used as such, without further characterization.

<u>HRMS</u>: ESI$^+$, *m/z* 276.1458 $[M+H]^+$, Error: 1.10 ppm

Synthesis of *tert*-butyl 3-((4-(5-(2,4-dioxotetrahydropyrimidin-1(2H)-yl)pyridin-2-yl)piperazin-1-yl)methyl)azetidine-1-carboxylate (S3.10a)

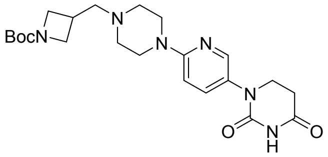

Following the general reductive amination procedure, the product was delivered (110 mg) with a yield of 62%.

^{1}H NMR ($CDCl_3$, 400MHz): δ = 1.45 (s, 9H), 2.48-2.53 (m, 4H), 2.62 (d, J= 7.3 Hz, 2H), 2.70-2.80 (m, 1H), 2.83 (t, J= 6.7 Hz, 2H), 3.52-3.57 (m, 4H), 3.61 (dd, J= 5.4; 8.6 Hz, 2H), 3.80 (t, J= 6.7 Hz, 2H), 4.03 (t, J= 8.3 Hz, 2H), 6.65 (d, J= 9.1 Hz, 1H), 7.43 (dd, J= 2.8; 9.0 Hz, 1H), 7.60 (s, 1H), 8.09 (d, J= 2.7 Hz, 1H) ppm.

^{13}C NMR ($CDCl_3$, 100 MHz): δ = 26.8 (CH), 28.6 (CH3), 31.6 (CH2), 45.3 (CH2), 45.8 (CH2), 53.1 (CH2), 53.5 (CH2, via HSQC), 62.6 (CH2), 79.4 (C), 106.9 (CH), 128.1 (C), 135.3 (CH), 144.8 (CH), 152.1 (C), 156.5 (C), 158.1 (C), 169.4 (C) ppm.

HRMS: ESI$^+$, *m/z* 445.2532 [M+H]$^+$, Error: 1.03 ppm

Synthesis of *tert*-butyl 3-((4-(5-(2,4-dioxotetrahydropyrimidin-1(2H)-yl)pyridin-2-yl)piperazin-1-yl)methyl)pyrrolidine-1-carboxylate (S3.10b)

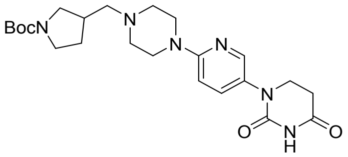

Following the general reductive amination procedure, the product was delivered (150 mg) with a yield of 60%.

^{1}H NMR ($CDCl_3$, 400MHz): δ = 1.46 (s, 9H), 1.58-1.65 (m, 1H), 1.95-2.05 (m, 1H), 2.31-2.62 (m, 7H), 2.82 (t, J= 6.7 Hz, 2H), 2.95-3.07 (m, 1H), 3.24-3.60 (m, 7H), 3.80 (t, J= 6.7 Hz, 2H), 6.65 (d, J= 9.1 Hz, 1H), 7.43 (dd, J= 2.8; 9.0 Hz, 1H), 7.82 (s, 1H), 8.09 (d, J= 2.7 Hz, 1H) ppm.

^{13}C NMR ($CDCl_3$, 100 MHz): δ = 28.6 (CH3), 29.7 (CH2), 30.4 (CH2), 31.5 (CH2), 35.8 (CH), 36.6 (CH), 45.2 (2 x CH2), 45.5 (CH2), 45.8 (CH2), 50.3 (CH2), 50.6 (CH2), 53.3 (CH2), 61.5 (CH2), 61.7 (CH2), 79.2 (C), 106.9 (CH), 128.1 (C), 135.3 (CH), 144.8 (CH), 152.1 (C), 154.7 (C), 158.1 (C), 169.5 (C) ppm.

HRMS: ESI$^+$, *m/z* 459.2711 [M+H]$^+$, Error: 0.71 ppm

Synthesis of *tert*-butyl 3-(4-(5-(2,4-dioxotetrahydropyrimidin-1(2H)-yl)pyridin-2-yl)piperazin-1-yl)pyrrolidine-1-carboxylate (S3.10c)

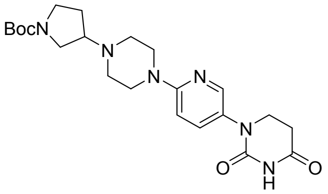

Following the general reductive amination procedure, the product was delivered (170 mg) with a yield of 71%.

^{1}H NMR (DMSO-d6, 400MHz): δ = 1.40 (s, 9H), 1.62-1.75 (m, 1H), 2.05 (br s, 1H), 2.39-2.58 (m, 4H), 2.70 (t, J= 6.7 Hz, 2H), 2.72-2.81 (m, 1H), 2.93-3.05 (m, 1H), 3.11-3.22 (m, 1H), 3.36-3.57 (m, 6H), 3.70 (t, J= 6.7 Hz, 2H), 6.84 (d, J= 9.1 Hz, 1H), 7.50 (dd, J= 2.8; 9.0 Hz, 1H), 8.06 (d, J= 2.6 Hz, 1H), 10.34 (s, 1H) ppm.

^{13}C NMR (DMSO-d6, 100 MHz): δ = 28.2 (CH3), 29.0 (CH2), 31.1 (CH2), 44.4 (CH2), 44.7 (CH2), 44.8 (CH2), 44.9 (CH2), 49.3 (CH2), 49.4 (CH2), 50.9 (CH2), 62.9 (CH), 63.7 (CH), 78.3 (C), 106.7 (CH), 129.0 (C), 135.3 (CH), 144.7 (CH), 152.1 (C), 153.4 (C), 157.1 (C), 170.6 (C) ppm.

HRMS: ESI$^+$, *m/z* 445.2556 [M+H]$^+$, Error: 0.40 ppm.

Synthesis of *tert*-butyl 4-((4-(5-(2,4-dioxotetrahydropyrimidin-1(2H)-yl)pyridin-2-yl)piperazin-1-yl)methyl)piperidine-1-carboxylate (S3.10d)

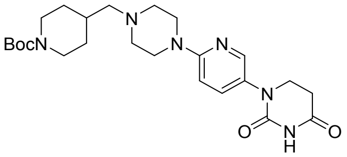

Following the general reductive amination procedure, the product was delivered (100 mg) with a yield of 58%.

^1H NMR ($CDCl_3$, 400MHz): δ = 1.09 (qd, J= 4.0; 12.3 Hz, 2H), 1.45 (s, 9H), 1.62-1.78 (m, 3H), 2.21 (d, J= 7.0 Hz, 2H), 2.47-2.51 (m, 4H), 2.70 (t, J= 12.3 Hz, 2H), 2.83 (t, J= 6.7 Hz, 2H), 3.52-3.56 (m, 4H), 3.80 (t, J= 6.7 Hz, 2H), 4.09 (br s, 2H), 6.65 (d, J= 9.1 Hz, 1H), 7.42 (dd, J= 2.8; 9.0 Hz, 1H), 7.63 (s, 1H), 8.09 (d, J= 2.6 Hz, 1H) ppm.

^{13}C NMR ($CDCl_3$, 100 MHz): δ = 28.6 (CH3), 30.9 (CH2), 31.6 (CH2), 33.7 (CH), 44.4 (CH2, broad signal), 45.4 (CH2), 45.8 (CH2), 53.5 (CH2), 64.7 (CH2), 79.4 (C), 106.9 (CH), 127.9 (C), 135.3 (CH), 144.7 (CH), 152.1 (C), 155.1 (C), 158.2 (C), 169.4 (C) ppm.

HRMS: ESI$^+$, *m/z* 473.2870 [M+H]$^+$, Error: 0.17 ppm.

Synthesis of *tert*-butyl 4-((4-(5-(2,4-dioxotetrahydropyrimidin-1(2H)-yl)pyridin-2-yl)piperazin-1-yl)methyl)-4-fluoropiperidine-1-carboxylate (S3.10e)

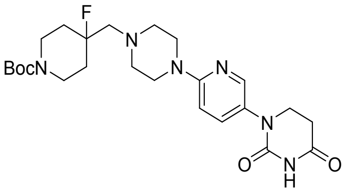

Following the general reductive amination procedure, the product was delivered (1.4 g) with a yield of 49%.

^1H NMR ($CDCl_3$, 400MHz): δ = 1.46 (s, 9H), 1.47-1.67 (m, 2H), 1.90-1.99 (m, 2H), 2.52 (d, J= 23.1 Hz, 2H), 2.61-2.68 (m, 4H), 2.83 (t, J= 6.7 Hz, 2H), 3.12 (t, J= 11.8 Hz, 2H), 3.50-3.57 (m, 2H), 3.80 (t, J= 6.7 Hz, 2H), 3.89 (br s, 2H), 6.65 (d, J= 9.1 Hz, 1H), 7.42 (dd, J= 2.8; 9.0 Hz, 1H), 7.49 (s, 1H), 8.09 (d, J= 2.6 Hz, 1H) ppm.

^{13}C NMR ($CDCl_3$, 100 MHz): δ = 28.6 (CH3), 31.6 (CH2), 33.4 (d, ^2J= 21.2 Hz, CH2), 39.6 (CH2, via HSQC), 45.5 (CH2), 45.8 (CH2), 54.6 (d, J= 2.5 Hz, CH2), 65.6 (d, ^2J= 20.9 Hz, CH2), 79.7 (C), 95.4 (d, ^1J= 172.5 Hz, C), 106.9 (CH), 128.0 (C), 135.3 (CH), 144.8 (CH), 152.1 (C), 154.9 (C), 158.2 (C), 169.4 (C) ppm.

^{19}F NMR ($CDCl_3$, 370 MHz): δ = -161.1 ppm.

HRMS: ESI$^+$, *m/z* 491.2773 [M+H]$^+$, Error: 0.73 ppm.

Synthesis of *tert*-butyl 4-(4-(5-(2,4-dioxotetrahydropyrimidin-1(2H)-yl)pyridin-2-yl)piperazine-1-carbonyl)-4-fluoropiperidine-1-carboxylate (S3.10f)

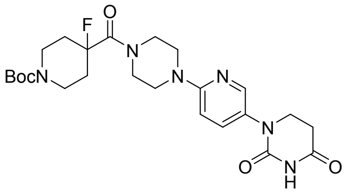

Following the general amide coupling procedure, the product was delivered (160 mg) with a yield of 55%.

^{1}H NMR (DMSO-d6, 400MHz): δ = 1.41 (s, 9H), 1.95-2.07 (m, 4H), 2.70 (t, J= 6.7 Hz, 2H), 2.99-3.15 (m, 2H), 3.51-3.68 (m, 6H), 3.71 (t, J= 6.7 Hz, 2H), 3.74-3.89 (m, 4H), 6.87 (d, J= 9.1 Hz, 1H), 7.55 (dd, J= 2.6; 9.0 Hz, 1H), 8.10 (d, J= 2.6 Hz, 1H), 10.35 (s, 1H) ppm.

^{13}C NMR (DMSO-d6, 100 MHz): δ = 28.0 (CH3), 31.1 (CH2), 32.4 (d, ^{2}J= 21.3 Hz, CH2), 39.3 (CH2, via HSQC), 42.4 (CH2), 44.7 (CH2), 44.9 (CH2), 45.2 (CH2), 79.0 (C), 95.5 (d, ^{1}J= 186.3 Hz, C), 106.8 (CH), 129.4 (C), 135.5 (CH), 144.8 (CH), 152.5 (C), 153.8 (C), 156.8 (C), 168.0 (d, ^{2}J= 20.8 Hz, C), 170.6 (C) ppm.

^{19}F NMR (DMSO-d6, 370 MHz): δ = -162.1 ppm

HRMS: ESI^{+}, *m/z* 505.2576 [M+H]$^{+}$, Error: 1.33 ppm

Synthesis of 6-chloropyridin-3-amine (S3.12)

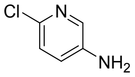

2-chloro-5-nitropyridine (10.0 g, 63.1 mmol, 1.0 eq), $SnCl_2$ (35.9 g, 189 mmol, 3.0 eq) and EtOH (200 mL) were heated at 80°C for 3h. Then, the reaction was cooled down to 0°C and sat. Na_2CO_3 was added, followed by filtration and an extraction of the filtrate with DCM. Drying over Na_2SO_4, and evaporation of the organic layer delivered 5.6 g of the product in 69% yield after column chromatography (DCM/MeOH 0 -> 3%).

^{1}H NMR ($CDCl_3$, 400MHz): δ = 3.76 (s, 2H), 6.95 (dd, J= 3.0; 8.5 Hz, 1H), 7.06 (d, J= 8.5 Hz, 1H), 7.83 (d, J= 3.0 Hz, 1H) ppm.

^{13}C NMR ($CDCl_3$, 100 MHz): δ = 124.2 (CH), 124.9 (CH), 136.4 (CH), 140.3 (C), 141.8 (C) ppm.

HRMS: ESI^{+}, *m/z* 129.0215 [M+H]$^{+}$, Error: 0.73 ppm

Synthesis of 1-(6-chloropyridin-3-yl)dihydropyrimidine-2,4(1H,3H)-dione (S3.14)

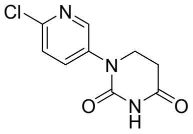

6-chloropyridin-3-amine (5.6 g, 43.6 mmol, 1.0 eq) was dissolved in toluene, followed by addition of acrylic acid (3.6 mL, 52.3 mmol, 1.2 eq) and heating to 100°C for 16h. Then, the reaction was evaporated and redissolved in AcOH. Ureum (7.9 g, 130.8 mmol, 3.0 eq) was added and after 8h stirring at 120°C, the AcOH was evaporated. The crude was redissolved in EA and washed with sat. Na_2CO_3. Drying over Na_2SO_4, and evaporation of the organic layer delivered 2.2 g of the product in 22% yield after column chromatography (DCM/MeOH 0 -> 4%).

^{1}H NMR (DMSO-d6, 400MHz): δ = 2.73 (t, J= 6.7 Hz, 2H), 3.85 (t, J= 6.7 Hz, 2H), 7.56 (d, J= 8.5 Hz, 1H), 7.86 (dd, J= 2.8; 8.5 Hz, 1H), 8.43 (d, J= 2.7 Hz, 1H), 10.56 (s, 1H) ppm.

^{13}C NMR (DMSO-d6, 100 MHz): δ = 30.9 (CH2), 43.9 (CH2), 123.9 (CH), 136.0 (CH), 138.0 (C), 146.2 (CH), 152.2 (C), 170.5 (C) ppm.

One quaternary carbon missing

HRMS: ESI$^+$, *m/z* 226.0386 $[M+H]^+$, Error: 3.65 ppm

Synthesis of *tert*-butyl 5-(2,4-dioxotetrahydropyrimidin-1(2H)-yl)-3',6'-dihydro-[2,4'-bipyridine]-1'(2'H)-carboxylate (S3.15)

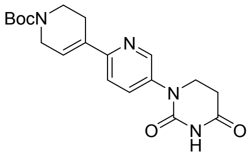

Dioxane (20 mL) and water (2 mL) were added to a flask with 1-(6-chloropyridin-3-yl)dihydropyrimidine-2,4(1H,3H)-dione (2.0 g, 8.86 mmol, 1.0 eq), *tert*-butyl 4-(4,4,5,5-tetramethyl-1,3,2-dioxaborolan-2-yl)-3,6-dihydropyridine-1(2H)-carboxylate (3.0 g, 9.75 mmol, 1.1 eq) and Na_2CO_3 (2.8 g, 26.6 mmol, 3.0 eq). The mixture was purged with argon for 5 minutes, followed by addition of $Pd(PPh_3)_4$ (512 mg, 0.43 mmol, 0.05 eq) and heating to 100°C for 16h. Brine was added and the mixture was extracted with EA. The organic layers were dried over Na_2SO_4, filtered and evaporated. Flash column chromatography (DCM/MeOH 0 ->5%) delivered the product (1.9 g) in 53% yield.

^{1}H NMR ($CDCl_3$, 400MHz): δ = 1.48 (s, 9H), 2.60-2.66 (m, 2H), 2.86 (t, J= 6.7 Hz, 2H), 3.64 (t, J= 5.4 Hz, 2H), 3.90 (t, J= 6.7 Hz, 2H), 4.11-4.15 (m, 2H), 6.57-6.62 (m, 1H), 7.41 (d, J= 8.5 Hz, 1H), 7.65 (dd, J= 2.8; 8.5 Hz, 1H), 8.02 (s, 1H), 8.52 (d, J= 2.7 Hz, 1H) ppm.

^{13}C NMR ($CDCl_3$ 100 MHz): δ = 26.0 (CH2), 28.6 (CH3), 31.4 (CH2), 39.8 (CH2, broad signal), 44.1 (CH2, broad signal), 44.9 (CH2), 79.9 (C), 119.0 (CH), 125.0 (CH, broad signal), 132.8 (CH), 134.8 (C), 136.2 (C), 144.9 (CH), 151.8 (C), 155.0 (C), 155.3 (C), 169.2 (C) ppm.

HRMS: ESI$^+$, *m/z* 373.1869 $[M+H]^+$, Error: 0.36 ppm

Synthesis of *tert*-butyl 4-(5-(2,4-dioxotetrahydropyrimidin-1(2H)-yl)pyridin-2-yl)piperidine-1-carboxylate (S3.16)

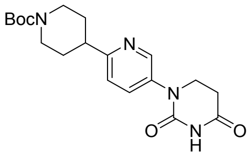

Tert-butyl 5-(2,4-dioxotetrahydropyrimidin-1(2H)-yl)-3',6'-dihydro-[2,4'-bipyridine]-1'(2'H)-carboxylate (1.9 g, 5.1 mmol, 1 eq) was dissolved in AcOH (10 mL, 1.29 mmol, 1eq), followed by addition of $Pd(OH)_2/C$. The reaction mixture was purged with N_2, followed by gas exchange with H_2 (2 balloons). After 48h of stirring, the reaction mixture was filtered over celite and washed with sat. $NaHCO_3$. This delivered the product (1.5 g) in 79% yield after purification (DCM/MeOH 0 -> 5%).

^{1}H NMR (DMSO-d6, 400MHz): δ = 1.41 (s, 9H), 1.57 (qd, J= 4.2; 12.5, 2H), 1.83 (d, J= 11.2 Hz, 2H), 2.72 (t, J= 6.7 Hz, 2H), 2.75-2.91 (m, 3H), 3.81 (t, J= 6.7 Hz, 2H), 4.03-4.10 (m, 2H), 7.33 (d, J= 8.5 Hz, 1H), 7.68 (dd, J= 2.6; 8.5 Hz, 1H), 8.48 (d, J= 2.5 Hz, 1H), 10.47 (s, 1H) ppm.

^{13}C NMR (DMSO-d6, 100 MHz): δ = 28.1 (CH3), 30.9 (CH2), 31.3 (CH2), 42.9 (CH), 43.9 (CH2, via HSQC), 44.2 (CH2), 78.6 (C), 121.0 (CH), 133.0 (CH), 136.6 (C), 145.8 (CH), 152.3 (C), 153.8 (C), 161.0 (C), 170.6 (C) ppm.

HRMS: ESI$^+$, *m/z* 375.2025 [M+H]$^+$, Error: 0.49 ppm

Synthesis of 1-(6-(piperidin-4-yl)pyridin-3-yl)dihydropyrimidine-2,4(1H,3H)-dione

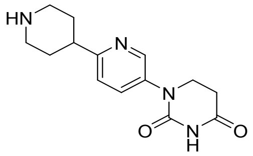

The Boc-protected intermediate (1.5 g, 4.0 mmol), was dissolved in 4M HCl in dioxane (10 mL) and stirred for 16h. The solids were filtered, washed once with diethyl ether and this delivered the product (1.3 g) as HCl salt in 94% yield after drying *in vacuo*. The product was used as such, without further characterization.

HRMS: ESI$^+$, *m/z* 275.1504 [M+H]$^+$, Error: 0.52 ppm

Synthesis of *tert*-butyl 4-((4-(5-(2,4-dioxotetrahydropyrimidin-1(2H)-yl)pyridin-2-yl)piperidin-1-yl)methyl)piperidine-1-carboxylate (S3.17a)

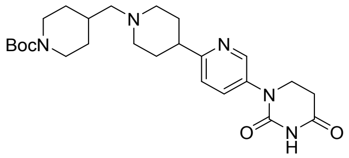

Following the general reductive amination procedure, the product was delivered (364 mg) with a yield of 89%.

^{1}H NMR ($CDCl_3$, 400MHz): δ = 1.08 (qd, J= 3.6; 11.6 Hz, 2H), 1.45 (s, 7H), 1.59-1.94 (m, 9H), 2.05 (td, J= 2.0; 11.5 Hz, 2H), 2.19 (d, J= 7.0 Hz, 2H), 2.63-2.76 (m, 3H), 2.86 (t, J= 6.7 Hz, 2H), 2.94-3.01 (m, 2H), 3.90 (t, J= 6.7 Hz, 2H), 4.07 (br s, 2H), 7.23 (d, J= 8.5 Hz, 1H), 7.62 (dd, J= 2.6; 8.5 Hz, 1H), 7.64 (s, 1H), 8.48 (d, J= 2.5 Hz, 1H) ppm.

^{13}C NMR ($CDCl_3$, 100 MHz): δ = 28.6 (CH3), 31.0 (CH2), 31.5 (CH2), 32.2 (CH2), 34.0 (CH), 44.0 (CH2, via HSQC), 44.5 (CH), 45.2 (CH2), 54.7 (CH2), 65.0 (CH2), 79.4 (C), 120.9 (CH), 133.3 (CH), 135.6 (C), 145.3 (CH), 151.8 (C), 155.1 (C), 163.8 (C), 169.1 (C) ppm.

HRMS: ESI$^+$, *m/z* 472.2902 [M+H]$^+$, Error: 3.45 ppm

Synthesis of *tert*-butyl 3-(4-(5-(2,4-dioxotetrahydropyrimidin-1(2H)-yl)pyridin-2-yl)piperidin-1-yl)azetidine-1-carboxylate (S3.17b)

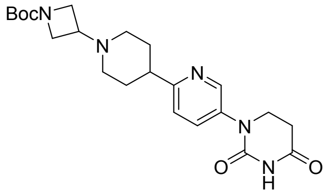

Following the general reductive amination procedure, the product was delivered (99 mg) with a yield of 80%.

^{1}H NMR ($CDCl_3$, 400MHz): δ = 1.45 (s, 9H), 1.79-1.89 (m, 2H), 1.92-2.03 (m, 4H), 2.77 (tt, J= 3.5; 12.1 Hz, 1H), 2.85 (t, J= 6.7 Hz, 2H), 2.95 (d, J= 10.6 Hz, 2H), 3.06-3.12 (m, 1H), 3.80-3.98 (m, 6H), 7.24 (d, J= 8.5 Hz, 1H), 7.62 (dd, J= 2.6; 8.5 Hz, 1H), 7.89 (s, 1H), 8.48 (d, J= 2.5 Hz, 1H) ppm.

^{13}C NMR ($CDCl_3$, 100 MHz): δ = 28.5 (CH3), 31.5 (CH2), 31.5 (CH2), 43.9 (CH), 45.1 (CH2), 50.4 (CH2), 52.4 (CH2, via HSQC), 54.1 (CH2, via HSQC), 54.2 (CH), 79.5 (C), 120.7 (CH), 133.4 (CH), 135.8 (C), 145.3 (CH), 151.8 (C), 156.5 (C), 163.1 (C), 169.2 (C) ppm.

HRMS: ESI$^+$, *m/z* 430.2448 $[M+H]^+$, Error: 0.18 ppm

Synthesis of *tert*-butyl 4-((4-(5-(2,4-dioxotetrahydropyrimidin-1(2H)-yl)pyridin-2-yl)piperidin-1-yl)methyl)-4-fluoropiperidine-1-carboxylate (S3.17c)

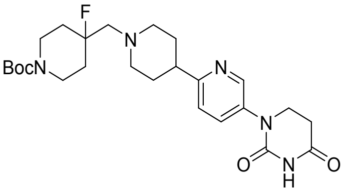

Following the general reductive amination procedure, the product was delivered (110 mg) with a yield of 39%.

^{1}H NMR ($CDCl_3$, 400MHz): δ = 1.46 (s, 9H), 1.49-1.70 (m, 2H), 1.79-1.95 (m, 6H), 2.23-2.36 (m, 2H), 2.50 (d, J= 22.5 Hz, 2H), 2.62-2.72 (m, 1H), 2.86 (t, J= 6.7 Hz, 2H), 2.95-3.06 (m, 2H), 3.11 (t, J= 12.0 Hz, 2H), 3.86 (br s, 2H), 3.90 (t, J= 6.7 Hz, 2H), 7.23 (d, J= 8.4 Hz, 1H), 7.62 (dd, J= 2.6; 8.5 Hz, 1H), 7.67 (s, 1H), 8.49 (d, J= 2.5 Hz, 1H) ppm.

^{13}C NMR ($CDCl_3$, 100 MHz): δ = 28.6 (CH3), 31.5 (CH2), 32.3 (CH2), 33.3 (d, ^{2}J= 21.8 Hz, CH2), 39.7 (CH2, via HSQC), 43.9 (CH), 45.2 (CH2), 55.8 (d, ^{4}J= 1.9 Hz, CH2), 65.7 (d, ^{2}J= 21.5 Hz, CH2), 79.7 (C), 95.5 (d, ^{2}J= 172.2 Hz, C), 121.1 (CH), 133.3 (CH), 135.7 (C), 145.3 (CH), 151.8 (C), 154.9 (C), 163.7 (C), 169.1 (C) ppm.

^{19}F NMR (DMSO-d6, 370 MHz): δ = -160.9 ppm.

HRMS: ESI$^+$, *m/z* 490.2825 $[M+H]^+$, Error: 0.18 ppm.

Synthesis of *tert*-butyl 4-(4-(5-(2,4-dioxotetrahydropyrimidin-1(2H)-yl)pyridin-2-yl)piperidine-1-carbonyl)-4-fluoropiperidine-1-carboxylate (S3.17d)

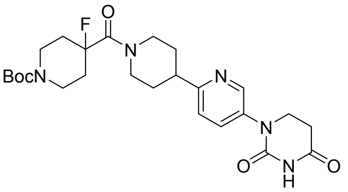

Following the general amide coupling procedure, the product was delivered (120 mg) with a yield of 41%.

^{1}H NMR ($CDCl_3$, 400MHz): δ = 1.45 (s, 9H), 1.79 (br s, 2H), 1.88-2.06 (m, 4H), 2.15 (br s, 2H), 2.75 (br s, 1H), 2.87 (t, J= 6.7 Hz, 2H), 3.01 (tt, J= 3.5; 11.9 Hz, 1H), 3.06-3.24 (m, 3H), 3.90 (t, J= 6.7 Hz, 2H), 3.99 (br s, 2H), 4.50-4.78 (m, 2H), 7.24 (d, J= 8.5 Hz, 1H), 7.62 (dd, J= 2.6; 8.5 Hz, 1H), 7.69 (s, 1H), 8.50 (d, J= 2.5 Hz, 1H) ppm.

^{13}C NMR ($CDCl_3$, 100 MHz): δ = 28.5 (CH3), 31.5 (CH2), 31.8 (CH2), 32.6 (CH2), 33.1 (CH2), 33.3 (CH2), 39.0 (CH2, via HSQC), 44.1 (CH2), 44.3 (CH), 45.1 (CH2), 46.4 (CH2), 80.0 (C), 95.9 (d, ^{2}J= 187.6 Hz, C), 121.3 (CH), 133.4 (CH), 136.0 (C), 145.5 (CH), 151.8 (C), 154.7 (C), 162.1 (C), 168.8 (C), 169.0 (C) ppm.

^{19}F NMR (DMSO-d6, 370 MHz): δ = -163.1 ppm.

HRMS: ESI$^+$, *m/z* 504.2606 $[M+H]^+$, Error: 2.14 ppm.

Synthesis of final dihydrouracil-based PROTACs (series 3)

Synthesis of PROTAC 21

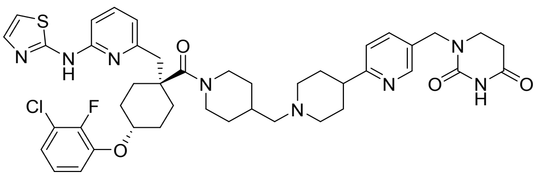

Following the general PROTAC synthesis procedure, 37 mg of the product was obtained with a yield of 45%.

^{1}H NMR (DMSO-d6, 400MHz): δ = 0.95-1.04 (m, 2H), 1.59-2.01 (m, 17H), 2.15 (d, J= 5.7 Hz, 2H), 2.50-2.66 (m, 3H), 2.67-3.00 (m, 4H), 3.06 (s, 2H), 3.34 (s, 2H), 4.41-4.56 (m, 5H), 6.66 (d, J= 7.3 Hz, 1H), 6.88 (d, J= 8.2 Hz, 1H), 6.98 (d, J= 3.5 Hz, 1H), 7.08-7.21 (m, 3H), 7.25 (d, J= 8.0 Hz, 1H), 7.36 (d, J= 3.5 Hz, 1H), 7.52-7.63 (m, 2H), 8.43 (s, 1H), 10.21 (s, 1H), 11.16 (s, 1H) ppm.

^{13}C NMR (DMSO-d6, 100MHz): δ = 27.0 (CH2), 29.5 (CH2), 30.7 (CH2), 30.9 (CH2), 31.7 (CH2), 33.2 (CH), 41.4 (CH2), 43.5 (CH), 44.9 (CH2), 47.1 (C), 47.2 (CH2), 54.0 (CH2), 64.2 (CH2), 73.8 (CH), 108.2 (CH), 110.7 (CH), 116.2 (CH), 116.8 (CH), 120.5 (d, ^{2}J(C,F) = 15.1 Hz, C), 120.9 (CH), 121.7 (CH), 124.9 (d, ^{3}J(C,F)= 4.9 Hz, CH), 130.4 (C), 135.9 (CH), 137.4 (CH), 137.7 (CH), 146.2 (d, ^{2}J(C,F) = 10.1 Hz, C), 148.3 (CH), 148.8 (d, ^{1}J(C,F) = 246.0 Hz, C), 150.8 (C), 153.2 (C), 154.9 (C), 159.7 (C), 163.7 (C), 170.5 (C), 171.5 (C) ppm

One CH2 from piperidine ring missing due to peak broadening.

^{19}F NMR (DMSO-d6, 377MHz): δ = -136.2 ppm

HRMS: ESI^{+}, *m/z* 829.3419 [M+H]$^{+}$, Error: 0.25 ppm

Synthesis of PROTAC 22

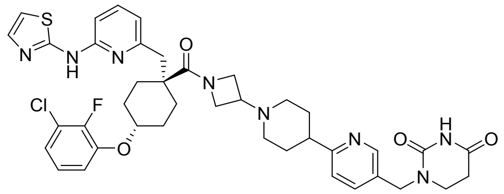

Following the general PROTAC synthesis procedure, 62 mg of the product was obtained with a yield of 79%.

^{1}H NMR (DMSO-d6, 400MHz): δ = 1.54-1.93 (m, 14H), 2.55 (t, J= 6.8 Hz, 2H), 2.59-2.91 (m, 6H), 3.34 (t, J= 6.8Hz, 2H), 3.63 (br s, 2H), 3.76-3.84 (m, 2H), 4.50 (s, 2H), 4.58 (s, 1H), 6.70 (d, J= 7.3 Hz, 1H), 6.92 (d, J= 8.2 Hz, 1H), 6.98 (d, J= 3.5 Hz, 1H), 7.08-7.21 (m, 3H), 7.27 (d, J= 8.0 Hz, 1H), 7.36 (d, J= 3.5 Hz, 1H), 7.59-7.66 (m, 2H), 8.43 (d, J= 1.8 Hz, 1H), 10.21 (s, 1H), 11.17 (s, 1H) ppm.

^{13}C NMR (DMSO-d6, 100MHz): δ = 26.8 (CH2), 28.7 (CH2), 30.7 (CH2), 31.1 (CH2), 41.4 (CH2), 43.2 (CH), 46.3 (CH2), 47.2 (CH2), 47.5 (C), 49.5 (CH2), 49.7 (CH2), 52.7 (CH2), 53.6 (CH), 56.3 (CH2), 74.2 (CH), 108.2 (CH), 110.7 (CH), 116.2 (CH), 116.8 (CH), 120.5 (d, ^{2}J(C,F) = 15.1 Hz, C), 120.9 (CH), 121.7 (CH), 124.9 (d, ^{3}J(C,F)= 4.9 Hz, CH), 130.4 (C), 135.9 (CH), 137.4 (CH), 137.7 (CH), 146.2 (d, ^{2}J(C,F) = 10.1 Hz, C), 148.3 (CH), 148.8 (d, ^{1}J(C,F) = 246.0 Hz, C), 150.8 (C), 153.2 (C), 154.9 (C), 159.7 (C), 163.7 (C), 170.5 (C), 173.1 (C) ppm

^{19}F NMR (DMSO-d6, 377MHz): δ = -136.2 ppm

HRMS: ESI^{+}, *m/z* 787.2943 [M+H]$^{+}$, Error: 1.09 ppm

Synthesis of PROTAC 23

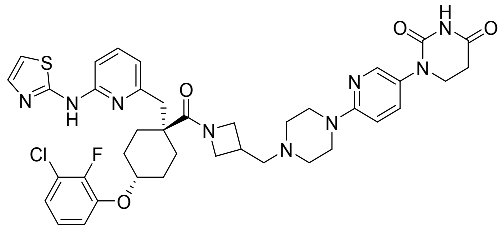

Following the general PROTAC synthesis procedure, 56 mg of the product was obtained with a yield of 71%.

^{1}H NMR (DMSO-d6, 400MHz): δ = 1.55-2.00 (m, 8H), 2.18-2.35 (m, 6H), 2.50-2.56 (m, 1H), 2.69 (t, J= 6.7 Hz, 2H), 2.81-2.93 (m, 2H), 3.08-3.12 (m, 1H), 3.34-3.44 (m, 5H), 3.69 (t, J= 6.7 Hz, 2H), 3.79 (t, J= 8.7 Hz, 1H), 3.87 (t, J= 7.8 Hz, 1H), 4.58 (s, 1H), 6.66 (d, J= 7.3 Hz, 1H), 6.84 (d, J= 9.1 Hz, 1H), 6.94 (d, J= 8.2 Hz, 1H), 6.97 (d, J= 3.6 Hz, 1H), 7.08-7.21 (m, 3H), 7.36 (d, J= 3.5 Hz, 1H), 7.50 (dd, J= 2.6; 9.0 Hz, 1H), 7.65 (t, J= 7.8 Hz, 1H), 8.06 (d, J= 2.6Hz, 1H), 10.35 (s, 1H), 11.23 (s, 1H) ppm.

^{13}C NMR (DMSO-d6, 100MHz): δ = 25.7 (CH), 26.8 (CH2), 27.9 (CH2), 29.8 (CH2), 31.1 (CH2), 44.8 (CH2), 44.9 (CH2), 46.8 (CH2), 47.5 (C), 52.4 (CH2), 52.7 (CH2), 56.7 (CH2), 61.5 (CH2), 74.2 (CH), 106.7 (CH), 108.3 (CH), 110.7 (CH), 116.2 (CH), 116.7 (CH), 120.4 (d, ^{2}J(C,F) = 15.1 Hz, C), 121.8 (CH), 124.9 (d, ^{3}J(C,F)= 4.9 Hz, CH), 129.0 (C), 135.4 (CH), 137.5 (CH), 137.9 (CH), 144.8 (CH), 146.2 (d, ^{2}J(C,F) = 10.1 Hz, C), 148.8 (d, ^{1}J(C,F) = 246.0 Hz, C), 151.1 (C), 152.6 (C), 155.1 (C), 157.2 (C), 159.7 (C), 170.7 (C), 173.1 (C) ppm.

^{19}F NMR (DMSO-d6, 377MHz): δ = -136.2 ppm

HRMS: ESI^{+}, *m/z* 788.2899 [M+H]$^{+}$, Error: 0.64 ppm

Synthesis of PROTAC 24

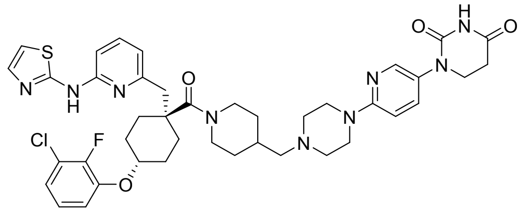

Following the general PROTAC synthesis procedure, 44 mg of the product was obtained with a yield of 54%.

^{1}H NMR (DMSO-d6, 400MHz): δ = 0.95-1.04 (m, 2H), 1.66-2.05 (m, 11H), 2.18 (d, J= 6.4 Hz, 2H), 2.44 (s, 4H), 2.70 (t, J= 6.7 Hz, 2H), 2.85 (br s, 2H), 3.06 (s, 2H), 3.47 (s, 4H), 3.70 (t, J= 6.7 Hz, 2H), 4.46 (d, J= 12.6 Hz, 2H), 4.55 (s, 1H), 6.66 (d, J= 7.3 Hz, 1H), 6.84 (d, J= 9.1 Hz, 1H), 6.88 (d, J= 8.2 Hz, 1H), 6.97 (d, J= 3.6 Hz, 1H), 7.08-7.15 (m, 3H), 7.36 (d, J= 3.5 Hz, 1H), 7.50 (dd, J= 2.6; 9.0 Hz, 1H), 7.58 (t, J= 7.8 Hz, 1H), 8.06 (d, J= 2.6Hz, 1H), 10.34 (s, 1H), 11.16 (s, 1H) ppm.

^{13}C NMR (DMSO-d6, 100MHz): δ = 27.0 (CH2), 29.5 (CH2), 30.7 (CH2), 31.1 (CH2), 32.9 (CH), 44.9 (CH2), 45.0 (2x CH2), 47.1 (C), 52.9 (CH2), 63.8 (CH2), 73.8 (CH), 106.7 (CH), 108.3 (CH), 110.7 (CH), 116.2 (CH), 116.5 (CH), 120.4 (d, ^{2}J(C,F) = 15.1 Hz, C), 121.8 (CH), 124.9 (d, ^{3}J(C,F)= 4.9 Hz, CH), 129.0 (C), 135.4 (CH), 137.5 (CH), 137.7 (CH), 144.8 (CH), 146.2 (d, ^{2}J(C,F) = 10.1 Hz, C), 148.8 (d, ^{1}J(C,F) = 246.0 Hz, C), 150.8 (C), 152.6 (C), 155.0 (C), 157.2 (C), 159.7 (C), 170.7 (C), 171.5 (C) ppm.

CH2 from piperidine ring missing due to peak broadening.

^{19}F NMR (DMSO-d6, 377MHz): δ = -136.4 ppm

HRMS: ESI^{+}, *m/z* 816.3226 [M+H]$^{+}$, Error: 1.10 ppm

Synthesis of PROTAC 25

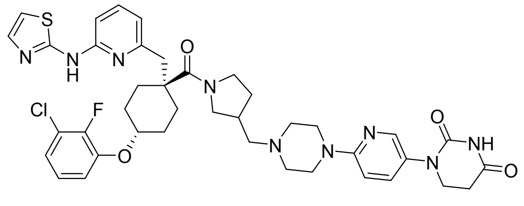

Following the general PROTAC synthesis procedure, 57 mg of the product was obtained with a yield of 71%.

^{1}H NMR (DMSO-d6, 400MHz): δ = 1.26-1.47 (m, 1H), 1.55-2.00 (m, 7H), 2.04-2.26 (m, 5H), 2.27-2.47 (m, 4H), 2.70 (t, J= 6.7 Hz, 2H), 2.88-3.05 (m, 3H), 3.15-3.55 (m, 7H), 3.69 (t, J= 6.7 Hz, 2H), 4.58 (s, 1H), 6.64 (d, J= 7.3 Hz, 1H), 6.84 (d, J= 8.5 Hz, 1H), 6.94 (d, J= 8.0 Hz, 1H), 6.96 (s, 1H), 7.08-7.19 (m, 3H), 7.36 (d, J= 3.5 Hz, 1H), 7.50 (dd, J= 2.6; 9.0 Hz, 1H), 7.59 (t, J= 7.8 Hz, 1H), 8.06 (d, J= 2.6Hz, 1H), 10.34 (s, 1H), 11.16 (s, 1H) ppm.

^{13}C NMR (DMSO-d6, 100MHz): δ = 26.7 (CH2), 27.0 (CH2), 28.7 (CH2), 30.3 (CH2), 31.1 (CH2), 32.6 (CH), 37.1 (CH), 44.9 (2x CH2), 46.1 (2x CH2), 47.7 (CH2), 48.2 (C), 50.9 (CH2), 52.7 (CH2), 53.0 (CH2), 60.3 (CH2), 61.1 (CH2), 74.2 (CH), 106.7 (CH), 108.3 (CH), 110.7 (CH), 116.2 (CH), 116.7 (CH), 120.4 (d, ^{2}J(C,F) = 15.1 Hz, C), 121.8 (CH), 124.9 (d, ^{3}J(C,F)= 4.9 Hz, CH), 129.0 (C), 135.4 (CH), 137.5 (CH), 137.7 (CH), 144.8 (CH), 146.2 (d, ^{2}J(C,F) = 10.1 Hz, C), 148.8 (d, ^{1}J(C,F) = 246.0 Hz, C), 151.0 (C), 152.6 (C), 155.1 (C), 157.1 (C), 159.7 (C), 170.7 (C), 171.6 (C), 172.0 (C) ppm.

^{19}F NMR (DMSO-d6, 377MHz): δ = -136.2 ppm.

HRMS: ESI$^+$, *m/z* 802.3054 [M+H]$^+$, Error: 0.82 ppm

Synthesis of PROTAC 26

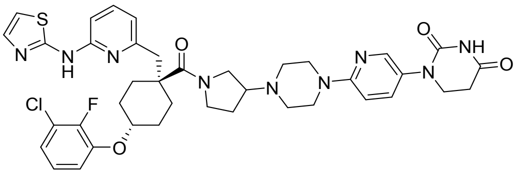

Following the general PROTAC synthesis procedure, 58 mg of the product was obtained with a yield of 74%.

^{1}H NMR (DMSO-d6, 400MHz, 70°C): δ = 1.53-2.01 (m, 8H), 2.10-2.17 (m, 2H), 2.40-2.44 (s, 2H), 2.50 (m, 2H, overlap with solvent signal), 2.57-2.65 (m, 1H), 2.71 (t, J= 6.7 Hz, 2H), 2.99 (s, 2H), 3.07-3.12 (m, 1H), 3.24-3.30 (m, 1H), 3.43-3.58 (m, 6H), 3.71 (t, J= 6.7 Hz, 2H), 4.55 (s, 1H), 6.66 (d, J= 7.3 Hz, 1H), 6.80 (d, J= 9.1 Hz, 1H), 6.90-6.97 (m, 2H), 7.08-7.15 (m, 3H), 7.36 (d, J= 3.5 Hz, 1H), 7.50 (dd, J= 2.6; 9.0 Hz, 1H), 7.58 (t, J= 7.8 Hz, 1H), 8.07 (d, J= 2.6Hz, 1H), 10.10 (s, 1H), 10.95 (s, 1H) ppm.

^{13}C NMR (DMSO-d6, 100MHz, 70°C): δ = 26.8 (CH2), 26.9 (CH2), 28.3 (CH2), 30.3 (CH2), 30.8 (CH2), 44.6 (CH2), 44.7 (CH2), 45.5 (CH2), 46.3 (CH2), 47.9 (C), 50.5 (CH2), 50.7 (CH2), 63.4 (CH, via HSQC), 74.5 (CH), 106.3 (CH), 108.1 (CH), 110.3 (CH), 115.8 (CH), 116.8 (CH), 120.2 (d, ^{2}J(C,F) = 15.1 Hz, C), 121.7 (CH), 124.5 (d, ^{3}J(C,F)= 4.9 Hz, CH), 128.8 (C), 135.0 (CH), 137.1 (CH), 137.3 (CH), 144.5 (CH), 146.2 (d, ^{2}J(C,F) = 10.1 Hz, C), 148.8 (d, ^{1}J(C,F) = 246.0 Hz, C), 150.8 (C), 152.2 (C), 154.9 (C), 156.9 (C), 159.6 (C), 170.1 (C), 171.6 (C) ppm

^{19}F NMR (DMSO-d6, 377MHz, 70°C): δ = -135.8 ppm

HRMS: ESI$^+$, *m/z* 788.2897 [M+H]$^+$, Error: 0.90 ppm

Synthesis of PROTAC 27

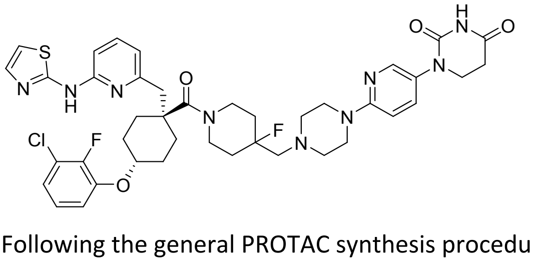

Following the general PROTAC synthesis procedure, 50 mg of the product was obtained with a yield of 60%.

^{1}H NMR (DMSO-d6, 400MHz): δ = 1.51-2.06 (m, 12H), 2.50-2.60 (m, 6H), 2.70 (t, J= 6.7 Hz, 2H), 3.08 (s, 2H), 3.24 (br s, 2H), 3.46 (s, 4H), 3.70 (t, J= 6.7 Hz, 2H), 4.16-4.23 (m, 2H), 4.55 (s, 1H), 6.68 (d, J= 7.3 Hz, 1H), 6.84 (d, J= 9.1 Hz, 1H), 6.88 (d, J= 8.2 Hz, 1H), 6.97 (d, J= 3.6 Hz, 1H), 7.08-7.15 (m, 3H), 7.36 (d, J= 3.5 Hz, 1H), 7.50 (dd, J= 2.6; 9.0 Hz, 1H), 7.59 (t, J= 7.8 Hz, 1H), 8.06 (d, J= 2.6Hz, 1H), 10.34 (s, 1H), 11.16 (s, 1H) ppm.

^{13}C NMR (DMSO-d6, 100MHz): δ = 26.9 (CH2), 29.5 (CH2), 31.1 (CH2), 33.1 (d, ^{2}J= 21.7 Hz, CH2), 44.9 (CH2), 45.1 (2x CH2), 47.1 (C), 54.0 (CH2), 64.1 (d, ^{2}J= 20.4 Hz, CH2), 73.8 (CH), 95.8 (d, ^{1}J= 171.7 Hz, C), 106.7 (CH), 108.3 (CH), 110.7 (CH), 116.2 (CH), 116.5 (CH), 120.4 (d, ^{2}J(C,F) = 15.1 Hz, C), 121.8 (CH), 124.9 (d, ^{3}J(C,F)= 4.9 Hz, CH), 129.0 (C), 135.4 (CH), 137.5 (CH), 137.7 (CH), 144.8 (CH), 146.2 (d, ^{2}J(C,F) = 10.1 Hz, C), 148.8 (d, ^{1}J(C,F) = 246.0 Hz, C), 150.9 (C), 152.6 (C), 155.0 (C), 157.2 (C), 159.7 (C), 170.7 (C), 171.8 (C) ppm

^{19}F NMR (DMSO-d6, 377MHz): δ = -136.4, -156.1 (broad s) ppm

HRMS: ESI^{+}, *m/z* 834.3133 [M+H]$^{+}$, Error: 1.22 ppm

Synthesis of PROTAC 28

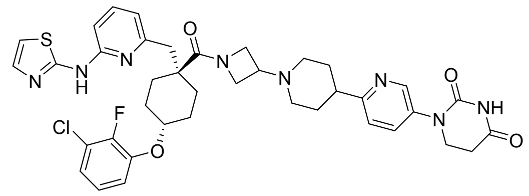

Following the general PROTAC synthesis procedure, 56 mg of the product was obtained with a yield of 72%.

^{1}H NMR (DMSO-d6, 400MHz): δ = 1.55-2.01 (m, 14H), 2.58-2.93 (m, 8H), 3.60-3.68 (m, 2H), 3.75-3.88 (m, 4H), 4.58 (s, 1H), 6.70 (d, J= 7.3 Hz, 1H), 6.93 (d, J= 8.2 Hz, 1H), 6.98 (d, J= 3.6 Hz, 1H), 7.08-7.21 (m, 3H), 7.33 (d, J= 8.5 Hz, 1H), 7.36 (d, J= 3.5 Hz, 1H), 7.61 (t, J= 7.8 Hz, 1H), 7.69 (dd, J= 2.6; 8.4 Hz, 1H), 8.48 (d, J= 2.5 Hz, 1H), 10.47 (s, 1H), 11.17 (s, 1H) ppm.

^{13}C NMR (DMSO-d6, 100MHz): δ = 26.8 (CH2), 28.7 (CH2), 31.0 (CH2), 31.1 (CH2), 43.0 (CH), 44.3 (CH2), 46.2 (CH2), 47.5 (C), 49.4 (CH2), 49.7 (CH2), 52.7 (CH2), 53.6 (CH), 56.3 (CH2), 74.2 (CH), 108.3 (CH), 110.8 (CH), 116.2 (CH), 116.7 (CH), 120.4 (d, ^{2}J(C,F) = 15.1 Hz, C), 120.8 (CH), 121.9 (CH), 124.9 (d, ^{3}J(C,F)= 4.9 Hz, CH), 133.0 (CH), 136.5 (C), 137.4 (CH), 137.9 (CH), 145.7 (CH), 146.2 (d, ^{2}J(C,F) = 10.1 Hz, C), 148.8 (d, ^{1}J(C,F) = 246.0 Hz, C), 151.0 (C), 152.3 (C), 155.0 (C), 159.7 (C), 161.5 (C), 170.6 (C), 173.1 (C) ppm.

^{19}F NMR (DMSO-d6, 377MHz): δ = -136.2 ppm

HRMS: ESI^{+}, *m/z* 773.2795 [M+H]$^{+}$, Error: 0.01 ppm

SYNTHESIS OF SERIES 4 PROTACS

(PROTACS BASED ON AURKA LIGAND 55)

Synthesis of AURKA ligand 55

A scheme for the synthesis of AURKA ligand **55**, can be found in the main text.

Synthesis of 6-bromo-2-(bromomethyl)-3-fluoropyridine (46)

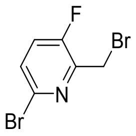

6-bromo-3-fluoro-2-methylpyridine (10 g, 52.6 mmol, 1 eq) and NBS (9.8 g, 55.2 mmol, 1.1 eq) were dissolved in CCl_4 (100 mL), followed by the addition of AIBN (0.43 g, 2.6 mmol, 0.05 eq). The reaction was heated to 75°C for 16h. After cooling down to RT, the precipitate was filtered and washed once with DCM. The filtrate was evaporated and purified via flash column chromatography (isocratic column with 100 % toluene). This delivered the product (8.25 g) in 89% yield.

^{1}H NMR (DMSO-d6, 400MHz): δ = 4.53 (d, J= 2.0 Hz, 2H), 7.31 (t, J= 8.5 Hz, 1H), 7.44 (dd, J= 3.4; 8.6 Hz, 1H) ppm.

^{13}C NMR (DMSO-d6, 100MHz): δ = 25.7 (d, ^{3}J= 1.7 Hz, CH2), 126.7 (d, ^{2}J= 20.6 Hz, CH), 129.8 (d, ^{3}J= 4.3 Hz, CH), 135.3 (d, ^{4}J= 3.0 Hz, C), 146.2 (d, ^{2}J= 16.3 Hz, C), 157.3 (d, ^{1}J= 261.0 Hz, C) ppm

^{19}F NMR (DMSO-d6, 377MHz): δ = -125.1 ppm

HRMS: ESI$^+$, *m/z* 267.8781 [M+H]$^+$, Error: 5.11 ppm

Synthesis of 1-(*tert*-butyl) 4-methyl 4-((6-bromo-3-fluoropyridin-2-yl)methyl)piperidine-1,4-dicarboxylate (49)

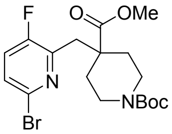

LDA (4.46 mL, 8.9 mmol, 1.2 eq, 2M in THF) was added to a previously dried 2-necked flask under Argon at -78°C with 10 mL dry THF. Then 1-(*tert*-butyl) 4-methyl piperidine-1,4-dicarboxylate (1.8 g, 7.4 mmol, 1.0 eq) was dissolved in dry THF and added to the RM, followed by the addition of 6-bromo-2-(bromomethyl)-3-fluoropyridine (2.0 g, 7.4 mmol, 1.0 eq, dissolved in dry THF) after 1h. 30 minutes later, the RM was quenched with NH_4Cl sat. until pH 7. Water was added and extracted 2 times with EA. The organic phase was dried over Na_2SO_4, filtered and evaporated. The product was purified using flash column chromatography with gradient T/EA 0 -> 5%. This yielded the product as a white powder (2.5 g) in 78% yield.

^{1}H NMR (DMSO-d6, 400MHz): δ = 1.44 (s, 9H), 1.45-1.55 (m, 2H), 2.03-2.09 (m, 2H), 2.81-2.95 (m, 2H), 3.03 (s, 2H), 3.73 (s, 3H), 3.88 (s, 2H), 7.21 (t, J= 8.5 Hz, 1H), 7.30 (dd, J= 3.4; 8.6 Hz, 1H) ppm.

^{13}C NMR (DMSO-d6, 100MHz): δ = 28.5 (CH3), 33.2 (CH2), 40.7 (d, ^{3}J= 2.7 Hz, CH2), 41.5 (CH2), 46.6 (C), 52.2 (CH3), 79.6 (C), 125.7 (d, ^{2}J= 22.0 Hz, CH), 129.7 (d, ^{3}J= 4.3 Hz, CH), 134.8 (d, ^{4}J= 2.8 Hz, C), 146.7 (d, ^{2}J= 17.8 Hz, C), 154.9 (C), 157.9 (d, ^{1}J= 256.0 Hz, C), 174.9 (C) ppm

^{19}F NMR (DMSO-d6, 377MHz): δ = -127.4 ppm

HRMS: ESI$^+$, *m/z* 431.0972 [M+H]$^+$, Error: 0.99 ppm

Synthesis of 1-(*tert*-butyl)-3-methyl-1H-pyrazol-5-amine (52)

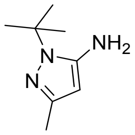

The product was synthesized according to a previous published method [4].

Tert-butyl hydrazine HCl (5.0 g, 40.1 mmol, 1 eq) was deprotonated by treatment with 2M NaOH (20 mL, 40.1 mmol, 1 eq), followed by addition of 3-aminobut-2-enenitril (3.3 g, 40.1 mmol, 1 eq) and heating to 90°C for 18h. Then, the RM was cooled down to 0°C and a spoon (+/- 2 g) of NaCl was added. The crystals were filtered and delivered the product (4.5g) in 73% yield after drying *in vacuo*.

^{1}H NMR (DMSO-d6, 400MHz): δ = 1.47 (s, 9H), 1.94 (s, 3H), 4.70 (s, 2H), 5.17 (s, 1H) ppm.

^{13}C NMR (DMSO-d6, 100MHz): δ = 13.9 (CH3), 29.1 (CH3), 56.9 (C), 91.4 (CH), 142.9 (C), 146.7 (C) ppm

HRMS: ESI$^+$, *m/z* 154.1342 [M+H]$^+$, Error: 2.08 ppm

Synthesis of 1-(*tert*-butyl) 4-methyl 4-((6-((1-(tert-butyl)-3-methyl-1H-pyrazol-5-yl)amino)-3-fluoropyridin-2-yl)methyl) piperidine-1,4-dicarboxylate (53)

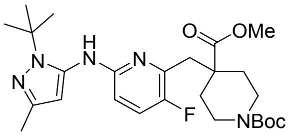

Toluene (15 mL) was added to a flask with 1-(*tert*-butyl)-3-methyl-1H-pyrazol-5-amine (0.9 g, 5.7 mmol, 1.0 eq), 1-(*tert*-butyl) 4-methyl 4-((6-bromo-3-fluoropyridin-2-yl)methyl)piperidine-1,4-dicarboxylate (2.5 g, 5.7 mmol, 1.0 eq), Xantphos (0.33 g, 0.57 mmol, 0.1 eq), Cs_2CO_3 (2.0 g, 6.3 mmol, 1.1 eq) and $Pd_2(dba)_3$ (0.26 g, 0.28 mmol, 0.05 eq) under argon atmosphere. After 18h of stirring at 110 °C, water was added and extracted with EA twice. The organic layers were washed with brine, dried over Na_2SO_4, filtered and evaporated. Flash column chromatography (PE/EA 0 -> 40%) delivered the product (1.9 g) in 66% yield.

^{1}H NMR ($CDCl_3$, 400MHz): δ = 1.44 (s, 9H), 1.48-1.57 (m, 2H), 1.59 (s, 9H), 2.06-2.13 (m, 2H), 2.23 (s, 3H), 2.85-2.97 (m, 4H), 3.68 (s, 3H), 3.87 (br s, 2H), 5.71 (s, 1H), 5.89 (s, 1H), 6.30 (dd, J= 2.9, 8.9 Hz, 1H), 7.14 (t, J= 8.8 Hz, 1H) ppm.

^{13}C NMR ($CDCl_3$, 100MHz): δ = 14.3 (CH3), 28.6 (CH3), 30.1 (CH3), 33.3 (CH2), 40.6 (CH2), 41.7 (CH2, via HSQC), 46.4 (C), 51.9 (CH3), 59.3 (C), 79.6 (C), 103.3 (CH), 106.4 (d, ^{3}J= 3.8 Hz, CH), 125.4 (d, ^{2}J= 22.4 Hz, CH), 138.7 (C), 142.7 (d, ^{2}J= 17.3 Hz, C), 145.8 (C), 153.0 (d, ^{1}J= 244.3 Hz, C), 153.1 (d, ^{4}J= 1.7 Hz, C), 155.0 (C), 175.4 (C) ppm

^{19}F NMR (DMSO-d6, 377MHz): δ = -137.9 ppm

HRMS: ESI$^+$, *m/z* 504.2983 [M+H]$^+$, Error: 0.48 ppm

Synthesis of methyl 4-((6-((1-(*tert*-butyl)-3-methyl-1H-pyrazol-5-yl)amino)-3-fluoropyridin-2-yl)methyl)-1-(3-chloro-2-fluorobenzyl)piperidine-4-carboxylate (54)

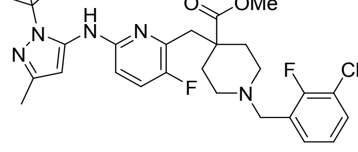

The Boc-protected intermediate (0.85 g, 1.7 mmol, 1.0 eq) was treated with TFA (5 mL) in DCM (10 mL) for 1 h. Next, the reaction was fully evaporated, redissolved in DMF (10 mL) and cooled down to 0°C. K_2CO_3 (1.2 g, 8.5

mmol, 5 eq) was slowly added, followed by 3-chloro-2-fluoro-benzyl bromide (0.42 g, 1.9 mmol, 1.2 eq). After 2h, water was added and extracted with DCM. The organic layers were washed with brine, dried over Na_2SO_4, filtered and evaporated. Flash column chromatography (DCM/MeOH 0 -> 5%) delivered the product (800 mg) in 87 % yield.

<u>^{1}H NMR ($CDCl_3$, 400MHz):</u> δ = 1.59 (s, 9H), 1.63-1.73 (m, 2H), 2.09-2.18 (m, 4H), 2.23 (s, 3H), 2.68-2.76 (m, 2H), 2.93 (d, J= 2.5 Hz, 2H), 3.53 (s, 2H), 3.67 (s, 3H), 5.71 (s, 1H), 5.89 (s, 1H), 6.29 (dd, J= 2.9, 8.9 Hz, 1H), 7.03 (t, J= 8.2 Hz, 1H), 7.12 (t, J= 8.8 Hz, 1H), 7.25-7.31 (m, 2H) ppm.

<u>^{13}C NMR ($CDCl_3$, 100MHz)</u>: δ = 14.3 (CH3), 30.1 (CH3), 33.5 (CH2), 40.8 (CH2), 46.1 (C), 51.0 (CH2), 51.7 (CH3), 55.6 (CH2), 59.3 (C), 103.3 (CH), 106.4 (d, ^{3}J= 3.8 Hz, CH), <u>121.0 (d, ^{2}J= 18.3 Hz, C), 124.3 (d, ^{3}J= 4.5 Hz, CH),</u> 125.3 (d, ^{2}J= 22.4 Hz, CH), <u>127.2 (d, ^{2}J= 14.5 Hz, C), 129.3 (CH), 129.7 (d, ^{3}J= 4.1 Hz, CH),</u> 138.8 (C), 143.0 (d, ^{2}J= 17.3 Hz, C), 145.8 (C), 153.0 (d, ^{1}J= 244.3 Hz, C), 153.1 (d, ^{4}J= 1.7 Hz, C), <u>156.8 (d, ^{1}J= 248.5 Hz, C),</u> 175.7 (C) ppm

<u>Carbons from phenyl ring</u>

<u>^{19}F NMR (DMSO-d6, 377MHz):</u> δ = -120.0, -137.8 ppm

<u>HRMS</u>: ESI$^+$, *m/z* 546.2446 [M+H]$^+$, Error: 0.76 ppm

Synthesis of 1-(3-chloro-2-fluorobenzyl)-4-((3-fluoro-6-((5-methyl-1H-pyrazol-3-yl)amino)pyridin-2-yl)methyl) piperidine-4-carboxylic acid (55)

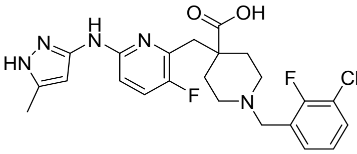

The methyl ester (0.80 g, 1.5 mmol, 1.0 eq) was treated with 2M NaOH (7.4 mL, 14.7 mmol, 10 eq) in MeOH (14 mL) for 24h at 60°C. Next, the reaction was cooled down and quenched with 4M HCl until pH = 5 to 6, then water was added and the precipitate filtered and dried *in vacuo*. The intermediate was redissolved in 4M HCl in dioxane and stirred at 95°C for 6h. Finally, the reaction was again cooled down to 0°C, Et_2O added and filtered. This delivered the product (710 mg) in 82 % yield over 2 steps as 3. HCl salt.

<u>^{1}H NMR (DMSO-d6, 400MHz):</u> δ = 1.91-2.05 (m, 2H), 2.09-2.24 (m, 2H), 2.29 (s, 3H), 2.83-2.94 (m, 2H), 3.01 (s, 2H), 3.32-3.43 (m, 2H), 4.36 (s, 2H), 6.20 (s, 1H), 7.05 (dd, J= 2.9, 9.0 Hz, 1H), 7.31 (t, J= 8.0 Hz, 1H), 7.56-7.62 (m, 1H), 7.65-7.76 (m, 2H), 10.26-10.87 (m, 2H), 12.87 (br s, 1H) ppm.

The product appears as rotameric mixture in a 1/3 ratio. Only peaks of the major rotamer are reported.

<u>^{13}C NMR (DMSO-d6, 100MHz)</u>: No ^{13}C NMR is reported due to peak broadening/ rotamers

<u>^{19}F NMR (DMSO-d6, 377MHz):</u> δ = -137.8 ppm

<u>HRMS</u>: ESI$^+$, *m/z* 476.1656 [M+H]$^+$, Error: 0.71 ppm

Synthesis of final AURKA ligand 55-based PROTACs (SERIES 4)

Synthesis of PROTAC 29

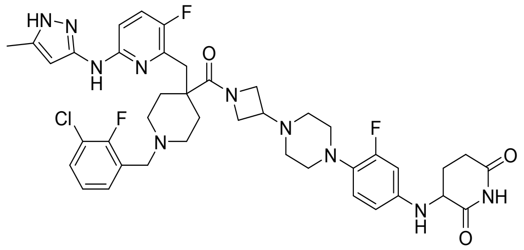

Following the general PROTAC synthesis procedure, 32 mg of the product was obtained with a yield of 39%.

^{1}H NMR (DMSO-d6, 400MHz, 70°C): δ = 1.59 (t, J= 10.5 Hz, 2H), 1.88 (qd, J= 4.1, 12.3 Hz, 1H), 2.00-2.22 (m, 8H), 2.35 (s, 4H), 2.55-2.79 (m, 4H), 2.80-2.89 (m, 6H), 2.95-3.03 (m, 1H), 3.51 (s, 2H), 3.64-3.95 (m, 4H), 4.16-4.26 (m, 1H), 5.63 (d, J= 7.3 Hz, 1H), 6.02 (br s, 1H), 6.45 (d, J= 8.6 Hz, 1H), 6.51 (dd, J= 1.9; 15.0 Hz, 1H), 6.80 (t, J= 9.2 Hz, 1H), 7.06 (br s, 1H), 7.18 (t, J= 7.8 Hz, 1H), 7.32-7.40 (m, 2H), 7.44 (t, J= 7.1 Hz, 1H), 8.67 (br s, 1H), 10.56 (s, 1H), 11.59 (br s, 1H) ppm.

^{13}C NMR (DMSO-d6, 100MHz, 70°C): δ = 10.8 (CH3, via HSQC), 24.4 (CH2), 30.3 (CH2), 32.9 (CH2), 38.5 (CH2), 45.4 (C), 49.0 (CH2), 50.0 (CH2), 50.4 (CH2), 52.7 (CH), 53.3 (CH), 54.5 (CH2), 94.4 (CH), 100.8 (d, ^{2}J= 24.4, CH), 108.4 (2 x CH), 119.2 (d, ^{2}J= 17.5 Hz, C), 120.4 (CH), 124.6 (2 x CH), 127.0 (d, ^{2}J(C,F)= 13.8 Hz, C), 128.8 (CH), 129.6 (d, ^{2}J= 9.8 Hz, C), 129.8 (CH), 137.9 (C, via HMBC), 141.4 (d, ^{2}J= 16.1 Hz, C), 144.3 (d, ^{3}J= 10.4 Hz, C), 151.0 (C), 151.4 (d, ^{1}J(C,F) = 241.6 Hz, C), 155.6 (d, ^{1}J= 247.0 Hz, C), 156.1 (d, ^{1}J= 242.0 Hz, C), 172.4 (C), 173.0 (C), 173.1 (C), ppm

CH2 from azetidine, and one quaternary carbon missing

^{19}F NMR (DMSO-d6, 377MHz): δ = -120.8, -123.3, -136.3 ppm

HRMS: ESI^{+}, *m/z* 819.3471 [M+H]$^{+}$, Error: 0.40 ppm

Synthesis of PROTAC 30

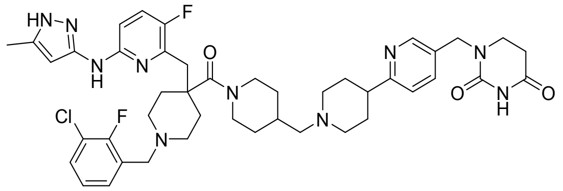

Following the general PROTAC synthesis procedure, 53 mg of the product was obtained with a yield of 63%.

^{1}H NMR (DMSO-d6, 400MHz): δ = 0.88-1.04 (m, 2H), 1.56 (t, J= 11.5 Hz, 2H), 1.67-1.85 (m, 7H), 1.97 (t, J= 9.9 Hz, 2H), 2.05-2.22 (m, 9H), 2.52-2.65 (m, 5H), 2.81 (br s, 2H), 2.87-3.00 (m, 4H), 3.30-3.35 (m, 2H, overlap with H_2O signal), 3.34 (s, 2H), 4.30-4.39 (m, 2H), 4.50 (s, 2H), 6.09 (br s, 1H), 7.06 (br s, 1H), 7.16 (t, J= 7.8 Hz, 1H), 7.26 (d, J= 8.1 Hz, 1H), 7.30-7.48 (m, 3H), 7.62 (dd, J= 2.2; 8.0 Hz, 1H), 8.43 (d, J= 1.9 Hz, 1H), 8.91 (br s, 1H), 10.22 (s, 1H), 11.70 (br s, 1H) ppm.

^{13}C NMR (DMSO-d6, 100MHz): δ = 10.8 (CH3), 30.7 (CH2), 30.8 (CH2), 31.7 (CH2), 33.2 (CH), 34.3 (CH2), 38.3 (CH2), 41.5 (CH2), 43.5 (CH), 45.5 (C), 45.6 (CH2, via HSQC), 47.2 (CH2), 50.6 (CH2), 54.0 (CH2), 54.8 (CH2), 64.2 (CH2), 94.2 (CH), 108.8 (CH), 119.4 (d, ^{2}J= 18.0 Hz, C), 120.9 (CH), 124.9 (CH), 125.0 (d, ^{3}J= 4.2 Hz, CH), 127.1 (d, ^{2}J(C,F)= 14.2 Hz, C), 129.1 (CH), 130.2 (d, ^{3}J= 4.0 Hz, CH), 130.4 (C), 136.0 (CH), 137.9 (C, via HMBC), 141.4 (d, ^{2}J=

16.1 Hz, C), 148.3 (CH), 151.0 (C), 151.6 (d, ^1J(C,F) = 241.0 Hz, C), 153.2 (C), 155.6 (d, ^1J= 247.0 Hz, C), 163.7 (C), 170.5 (C), 171.5 (C), ppm

One quaternary carbon missing

<u>^{19}F NMR (DMSO-d6, 377MHz):</u> δ = -120.8, -138.4 ppm

<u>HRMS</u>: ESI$^+$, *m/z* 843.4029 [M+H]$^+$, Error: 0.29 ppm

Synthesis of PROTAC 31

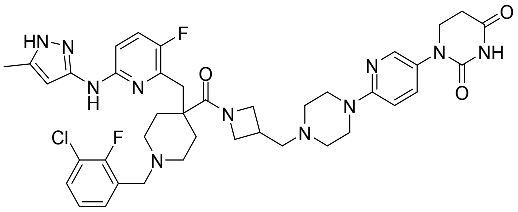

Following the general PROTAC synthesis procedure, 27 mg of the product was obtained with a yield of 34%.

<u>^{1}H NMR (DMSO-d6, 400MHz):</u> δ = 1.46-1.65 (m, 2H), 1.95-2.16 (m, 4H), 2.16 (s, 3H), 2.25-2.35 (m, 6H), 2.54-2.65 (m, 3H), 2.70 (t, J= 6.7 Hz, 2H), 2.79 (s, 2H), 3.23 (br s, 1H), 3.34-3.52 (m, 7H), 3.69 (t, J= 6.7 Hz, 2H), 3.77-3.99 (m, 2H), 6.18 (br s, 1H), 6.81 (d, J= 9.1 Hz, 1H), 7.09-7.23 (m, 2H), 7.34-7.53 (m, 4H), 8.05 (d, J= 2.6 Hz, 1H), 9.00 (s, 1H), 10.34 (s, 1H), 11.68 (s, 1H) ppm.

<u>^{13}C NMR (DMSO-d6, 100MHz)</u>: δ = 10.8 (CH3), 25.9 (CH), 31.1 (CH2), 32.5 (CH2), 34.1 (CH2), 39.4 (CH2, via HSQC), 44.8 (CH2), 44.9 (CH2), 45.9 (C), 50.3 (CH2), 52.3 (CH2), 52.8 (CH2), 54.8 (CH2), 56.7 (CH2), 61.5 (CH2), 94.4 (CH, via HSQC), 106.6 (CH), 109.0 (CH), 119.5 (d, ^2J= 18.0 Hz, C), 125.1 (2x CH), 127.3 (C), 129.0 (C), 129.2 (CH), 130.2 (CH), 135.4 (CH), 137.9 (C, via HMBC), 141.5 (d, ^2J= 15.9 Hz, C), 144.8 (CH), 151.0 (C), 151.6 (d, ^1J(C,F) = 241.9 Hz, C), 152.6 (C), 155.8 (d, ^1J= 247.1 Hz, C), 157.1 (C), 170.7 (C) ppm

Two quaternary carbon missing

<u>^{19}F NMR (DMSO-d6, 377MHz):</u> δ = -120.8, -139.1 ppm

<u>HRMS</u>: ESI$^+$, *m/z* 802.3514 [M+H]$^+$, Error: 0.06 ppm

Synthesis of PROTAC 32 (SK4454)

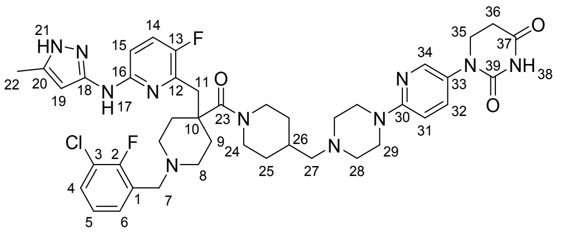

Following the general PROTAC synthesis procedure, 36 mg of the product was obtained with a yield of 43%.

<u>^{1}H NMR (DMSO-d6, 400MHz):</u> δ = 0.89-1.06 (m, 2H, $H_{25\alpha}$), 1.48-1.67 (m, 2H, $H_{9\alpha}$), 1.70-1.89 (m, 3H, $H_{25\beta\,\&\,26}$), 2.05-2.20 (m, 9H, $H_{8\alpha,\,9\beta,\,22,\,27}$), 2.44 (s, 4H, $H_{28/29}$), 2.54-2.64 (m, 2H, $H_{8\beta}$), 2.70 (t, J= 6.7 Hz, 2H, H_{36}), 2.81 (br s, 2H, $H_{24\alpha}$), 2.93 (s, 2H, H_{11}), 3.39-3.52 (m, 6H, $H_{7\,\&\,28/29}$), 3.70 (t, J= 6.7 Hz, 2H, H_{35}), 4.28-4.41 (m, 2H, $H_{24\beta}$), 6.09 (br s, 1H, H_{19}), 6.83 (d, J= 9.1 Hz, 1H, H_{31}), 7.05 (br s, 1H, H_{15}), 7.16 (t, J= 7.8 Hz, 1H, H_5), 7.28-7.48 (m, 3H, $H_{4,\,6,\,14}$), 7.50 (dd, J= 2.2; 9.0 Hz, 1H, H_{32}), 8.06 (d, J= 2.6 Hz, 1H, H_{34}), 8.94 (s, 1H, H_{17}), 10.35 (s, 1H, H_{38}), 11.70 (s, 1H, H_{21}) ppm.

<u>^{13}C NMR (DMSO-d6, 100MHz)</u>: δ = 10.9 ($CH3_{22}$), 30.7 ($CH2_{25}$), 31.1 ($CH2_{36}$), 32.9 (CH_{26}), 34.2 ($CH2_9$), 38.3 ($CH2_{11}$), 44.9 ($CH2_{35}$), 45.0 (2x $CH2_{28/29\,\&\,24}$), 45.5 (C_{10}), 50.6 ($CH2_8$), 53.0 ($CH2_{28/29}$), 54.8 ($CH2_7$), 63.9 ($CH2_{27}$), 94.3 (CH_{19}),

106.6 (CH_{31}), 108.9 (CH_{15}), 119.4 (d, ^2J= 18.0 Hz, C_3), 125.0 (CH_5), 125.0 (d, ^2J= 4.1 Hz, CH_{14}), 127.1 (d, ^2J= 14.0 Hz, C_1), 129.0 (C_{33}), 129.1 (CH_4), 130.2 (CH_6), 135.4 (CH_{32}), 137.9 (C_{20}, via HMBC), 141.4 (d, ^2J= 16.4 Hz, C_{12}), 144.8 (CH_{34}), 151.0 (C_{16}), 151.6 (d, $^1J(C,F)$ = 240.9 Hz, C_{13}), 152.5 (C_{39}), 155.7 (d, ^1J= 247.0 Hz, C_2), 157.2 (C_{30}), 170.7 (C_{37}), 171.5 (C_{23}), ppm. One quaternary (C_{18}) carbon missing.

^{19}F NMR (DMSO-d6, 377MHz): δ = -120.8, -138.4 ppm

HRMS: ESI$^+$, *m/z* 830.3836 [M+H]$^+$, Error: 1.03 ppm

Synthesis of PROTAC 33 (SK5527)

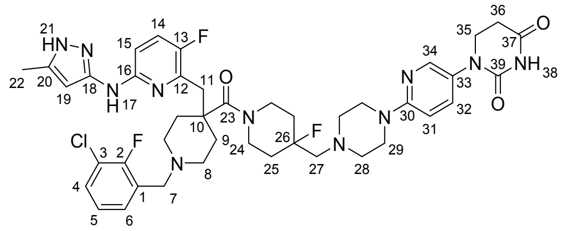

Following the general PROTAC synthesis procedure, 1.3 g of the product was obtained with a yield of 65%.

^{1}H NMR (DMSO-d6, 400MHz): δ = 1.49-1.72 (m, 4H, $H_{9\alpha\,\&\,25\alpha}$), 1.81-1.94 (m, 2H, $H_{25\beta}$), 2.05-2.22 (m, 7H, $H_{8\alpha,\,9\beta,\,22}$), 2.53-2.65 (m, 8H, $H_{8\beta,\,28/29,\,27}$), 2.69 (t, J= 6.7 Hz, 2H, H_{36}), 2.96 (s, 2H, H_{11}), 3.16 (br s, 2H, $H_{24\alpha}$), 3.40-3.53 (m, 6H, $H_{7,\,28/29}$), 3.69 (t, J= 6.7 Hz, 2H, H_{35}), 4.06-4.17 (m, 2H, $H_{24\beta}$), 6.11 (br s, 1H, H_{19}), 6.83 (d, J= 9.1 Hz, 1H, H_{31}), 7.02 (br s, 1H, H_{15}), 7.15 (t, J= 7.8 Hz, 1H, H_5), 7.28-7.55 (m, 3H, $H_{4,\,6,\,15}$), 7.50 (dd, J= 2.7; 9.0 Hz, 1H, H_{32}), 8.06 (d, J= 2.6 Hz, 1H, H_{34}), 8.94 (s, 1H, H_{17}), 10.34 (s, 1H, H_{38}), 11.69 (s, 1H, H_{21}) ppm.

^{13}C NMR (DMSO-d6, 100MHz): δ = 10.8 ($CH3_{22}$), 31.1 ($CH2_{36}$), 33.1 (d, ^2J= 21.6 Hz, $CH2_{25}$), 34.3 ($CH2_9$), 38.3 ($CH2_{11}$), 41.7 ($CH2_{24}$, via HSQC), 44.9 ($CH2_{35}$), 45.0 ($CH2_{28/29}$), 45.5 (C_{10}), 50.5 ($CH2_8$), 54.0 ($CH2_{28/29}$), 54.8 ($CH2_7$), 64.2 (d, ^2J= 19.7 Hz, $CH2_{27}$), 94.1 (CH_{19}), 95.9 (d^1, J= 171.6 Hz, C_{26}), 106.6 (CH_{31}), 109.0 (CH_{15}), 119.5 (d, ^2J= 17.8 Hz, C_3), 125.0 (2x $CH_{5,\,14}$), 127.1 (d, ^2J= 14.2 Hz, C_1), 129.0 (C_{33}), 129.1 (CH_4), 130.1 (CH_6), 135.4 (CH_{32}), 137.8 (C_{20}, via HMBC), 141.4 (d, ^2J= 16.7 Hz, C_{12}), 144.8 (CH_{34}), 149.9 (C_{18}), 151.1 (C_{16}), 151.6 (d, $^1J(C,F)$ = 240.9 Hz, C_{13}), 152.5 (C_{39}), 155.7 (d, ^1J= 247.1 Hz, C_2), 157.1 (C_{30}), 170.7 (C_{37}), 171.6 (C_{23}), ppm

^{19}F NMR (DMSO-d6, 377MHz): δ = -120.8, -138.4, -156.1 ppm

HRMS: ESI$^+$, *m/z* 848.3728 [M+H]$^+$, Error: 0.62 ppm

Synthesis of PROTAC 34

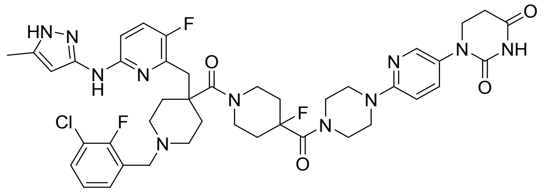

Following the general PROTAC synthesis procedure, 59 mg of the product was obtained with a yield of 68%.

^{1}H NMR (DMSO-d6, 400MHz): δ = 1.60 (br s, 2H), 1.93-2.23 (m, 9H), 2.53-2.74 (m, 6H), 2.93 (s, 2H), 3.16 (br s, 2H), 3.45 (s, 2H), 3.57 (s, 4H), 3.63-3.75 (m, 4H), 3.90 (br s, 2H), 4.25-4.35 (m, 2H), 5.75 (br s, 1H), 6.88 (d, J= 9.1 Hz, 1H), 7.18 (t, J= 7.8 Hz, 1H), 7.28-7.55 (m, 4H), 7.55 (dd, J= 2.6; 9.0 Hz, 1H), 8.10 (d, J= 2.6 Hz, 1H), 8.59 (s, 1H), 10.36 (s, 1H), 11.72 (s, 1H) ppm.

^{13}C NMR (DMSO-d6, 100MHz): δ = 10.7 (CH3), 31.1 (CH2), 32.5 (d, ^2J= 20.6 Hz, CH2), 34.4 (CH2), 38.3 (CH2), 42.7 (CH2), 44.6 (CH2), 44.9 (CH2), 45.3 (CH2), 45.7 (C), 50.6 (CH2), 54.8 (CH2), 93.7 (CH), 96.3 (d, J= 188.9 Hz, C),

106.8 (CH), 108.5 (CH), 119.5 (d, ^{2}J= 17.8 Hz, C), 124.8 (CH), 125.0 (d, ^{3}J= 4.0 Hz, CH), 127.1 (d, ^{2}J= 14.2 Hz, C), 129.2 (CH), 129.4 (C), 130.2 (d, ^{3}J= 3.6 Hz, CH), 135.4 (CH), 138.1 (C), 141.6 (d, ^{2}J= 16.7 Hz, C), 144.8 (CH), 150.9 (2x C), 151.6 (d, ^{1}J(C,F) = 240.9 Hz, C), 152.5 (C), 155.7 (d, ^{1}J= 247.1 Hz, C), 156.7 (C), 168.9 (C), 170.7 (C), 171.3 (C), ppm

^{19}F NMR (DMSO-d6, 377MHz): δ = -120.8, -137.4, -161.2 ppm

HRMS: ESI$^+$, *m/z* 862.3526 [M+H]$^+$, Error: 0.02 ppm

Synthesis of PROTAC 35

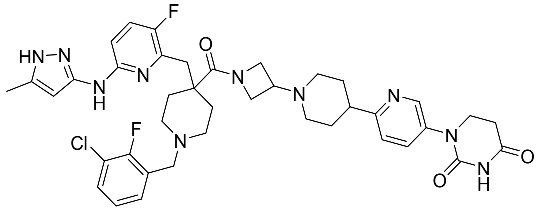

Following the general PROTAC synthesis procedure, 61 mg of the product was obtained with a yield of 78%.

^{1}H NMR (DMSO-d6, 400MHz): δ = 1.46-1.89 (m, 8H), 1.95-2.16 (m, 4H), 2.16 (s, 3H), 2.56-2.99 (m, 10H), 3.49 (br s, 2H), 3.64 (br s, 1H), 3.81 (t, J= 6.7 Hz, 2H), 3.81-3.98 (m, 3H), 6.11 (br s, 1H), 7.09 (br s, 1H), 7.19 (t, J= 7.7 Hz, 1H), 7.28 (d, J= 8.5 Hz, 1H), 7.33-7.53 (m, 3H), 7.68 (dd, J= 2.6; 8.4 Hz, 1H), 8.47 (d, J= 2.6 Hz, 1H), 8.89 (br s, 1H), 10.47 (s, 1H), 11.72 (s, 1H) ppm.

^{13}C NMR (DMSO-d6, 100MHz): δ = 11.1 (CH3, via HSQC), 31.0 (2 x CH2), 33.4 (CH2, br s), 39.4 (CH2, via HSQC), 43.1 (CH), 44.3 (CH2), 45.7 (C), 49.6 (CH2), 49.7 (CH2), 50.3 (CH2), 52.9 (CH2), 53.8 (CH), 54.8 (CH2), 56.3 (CH2), 94.4 (CH), 108.7 (CH), 119.4 (d, ^{2}J= 18.0 Hz, C), 120.7 (CH), 125.1 (2x CH), 127.1 (C), 129.2 (CH), 130.2 (CH), 133.0 (CH), 136.5 (C), 137.9 (C, via HMBC), 141.4 (C), 145.7 (CH), 151.2 (C), 151.6 (d, ^{1}J(C,F) = 241.5 Hz, C), 152.3 (C), 155.8 (d, ^{1}J= 246.6 Hz, C), 161.5 (C), 170.6 (C), 173.1 (C), ppm

One quaternary carbon missing

^{19}F NMR (DMSO-d6, 377MHz): δ = -120.7, -139.3 ppm

HRMS: ESI$^+$, *m/z* 787.3405 [M+H]$^+$, Error: 0.06 ppm

Synthesis of PROTAC 36

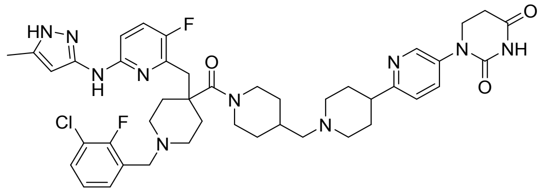

Following the general PROTAC synthesis procedure, 49 mg of the product was obtained with a yield of 59%.

^{1}H NMR (DMSO-d6, 400MHz): δ = 0.90-1.09 (m, 2H), 1.49-1.65 (m, 2H), 1.69-2.20 (m, 16H), 2.54-2.75 (m, 5H), 2.76-3.12 (m, 6H), 3.34-3.48 (m, 4H), 3.81 (t, J= 6.7 Hz, 2H), 4.30-4.39 (m, 2H), 6.07 (br s, 1H), 7.05 (br s, 1H), 7.16 (t, J= 7.8 Hz, 1H), 7.28-7.51 (m, 4H), 7.69 (d, J= 7.5 Hz, 1H), 8.49 (s, 1H), 8.92 (s, 1H), 10.48 (s, 1H), 11.71 (s, 1H) ppm.

^{13}C NMR (DMSO-d6, 100MHz): δ = 10.6 (CH3, via HSQC), 30.6 (CH2), 31.0 (CH2), 34.2 (CH2), 38.3 (CH2), 44.2 (CH2), 45.4 (CH2, via HSQC), 45.5 (C), 50.6 (CH2), 54.8 (CH2), 93.9 (CH), 108.9 (CH), 119.5 (d, ^{2}J= 17.8 Hz, C), 120.9 (CH), 125.0 (2x CH), 127.1 (C), 129.2 (CH), 130.2 (CH), 133.0 (CH), 136.4 (C), 138.8 (C, via HMBC), 141.3 (C), 145.8

(CH), 150.9 (C), 151.7 (d, $^1J(C,F)$ = 241.3 Hz, C), 152.3 (C), 155.7 (d, ^1J= 247.1 Hz, C), 161.8 (C, via HMBC), 170.6 (C), 171.6 (C), ppm

1x C, 1x CH and 3x CH2 missing

^{19}F NMR (DMSO-d6, 377MHz): δ = -120.8, -138.4 ppm

HRMS: ESI$^+$, *m/z* 829.3871 [M+H]$^+$, Error: 0.48 ppm

Synthesis of PROTAC 37

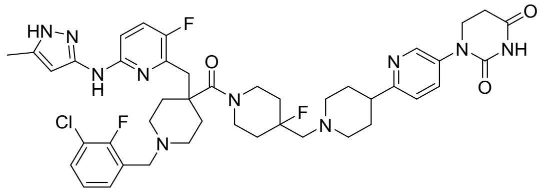

Following the general PROTAC synthesis procedure, 55 mg of the product was obtained with a yield of 65%.

^{1}H NMR (DMSO-d6, 400MHz): δ = 1.49-1.65 (m, 4H), 1.67-1.90 (m, 6H), 2.06-2.26 (m, 9H), 2.53-2.68 (m, 5H), 2.71 (t, J= 6.7 Hz, 2H), 2.91-3.06 (m, 4H), 3.16 (br s, 2H), 3.45 (s, 2H), 3.79 (t, J= 6.7 Hz, 2H), 4.05-4.19 (m, 2H), 6.10 (br s, 1H), 7.02 (br s, 1H), 7.16 (t, J= 7.8 Hz, 1H), 7.28-7.51 (m, 4H), 7.66 (dd, J= 2.6; 8.3 Hz, 1H), 8.46 (d, J= 2.6 Hz, 1H), 8.97 (s, 1H), 10.47 (s, 1H), 11.70 (s, 1H) ppm.

^{13}C NMR (DMSO-d6, 100MHz): δ = 10.6 (CH3, via HSQC), 31.0 (CH2), 31.8 (CH2), 33.0 (d, ^2J= 21.5 Hz, CH2), 34.3 (CH2), 38.3 (CH2), 41.7 (CH2, via HSQC), 42.8 (CH), 44.2 (CH2), 45.5 (C), 50.5 (CH2), 54.8 (CH2), 55.1 (CH2), 64.4 (d, ^2J= 20.0 Hz, CH2), 93.9 (CH), 95.9 (d, J= 172.0 Hz, C), 109.0 (CH), 119.5 (d, ^2J= 17.8 Hz, C), 120.8 (CH), 125.0 (2x CH), 127.1 (C), 129.2 (CH), 130.1 (CH), 133.0 (CH), 136.4 (C), 138.8 (C, via HMBC), 141.3 (d, ^2J= 17.1 Hz, C), 145.7 (CH), 150.9 (2x C), 151.7 (d, $^1J(C,F)$ = 241.3 Hz, C), 152.3 (C), 155.7 (d, ^1J= 247.1 Hz, C), 161.7 (C), 170.6 (C), 171.6 (C), ppm

^{19}F NMR (DMSO-d6, 377MHz): δ = -120.8, -138.4, -156.2 (br s) ppm

HRMS: ESI$^+$, *m/z* 847.3779 [M+H]$^+$, Error: 0.21 ppm

Synthesis of PROTAC 38

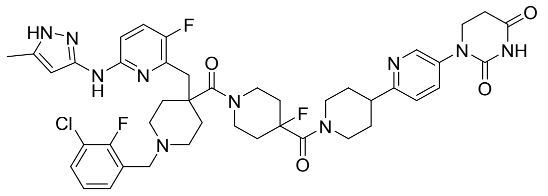

Following the general PROTAC synthesis procedure, 60 mg of the product was obtained with a yield of 70%.

^{1}H NMR (DMSO-d6, 400MHz): δ = 1.52-1.81 (br s, 4H), 1.87-2.23 (m, 11H), 2.53-2.76 (m, 4H), 2.77-3.10 (m, 5H), 3.15-3.44 (m, 4H), 3.45 (s, 2H), 3.81 (t, J= 6.7 Hz, 2H), 4.23-4.33 (m, 2H), 4.43-4.61 (m, 2H), 5.89 (br s, 1H), 7.18 (t, J= 7.8 Hz, 1H), 7.28-7.55 (m, 5H), 7.70 (dd, J= 2.6; 8.3 Hz, 1H), 8.48 (d, J= 2.6 Hz, 1H), 8.58 (s, 1H), 10.47 (s, 1H), 11.72 (s, 1H) ppm.

^{13}C NMR (DMSO-d6, 100MHz): δ = 10.6 (CH3), 31.0 (CH2), 31.3 (CH2), 32.3 (CH2), 32.7 (CH2), 34.5 (CH2), 38.9 (CH2), 42.8 (CH), 43.7 (CH2), 44.2 (CH2), 45.7 (CH2), 45.7 (C), 50.6 (CH2), 54.8 (CH2), 93.7 (CH), 96.3 (d, J= 187.2 Hz, C), 108.5 (CH), 119.5 (d, ^2J= 17.8 Hz, C), 121.1 (CH), 124.8 (CH), 125.0 (d, ^3J= 3.9 Hz, CH), 127.3 (C), 129.2 (CH),

130.2 (CH), 133.0 (CH), 136.7 (C), 138.3 (C, via HMBC), 141.6 (d, ^{2}J= 17.1 Hz, C), 145.8 (CH), 150.9 (2x C), 151.7 (d, ^{1}J(C,F) = 241.3 Hz, C), 152.3 (C), 155.7 (d, ^{1}J= 247.1 Hz, C), 160.6 (C), 168.6 (C), 170.6 (C), 171.3 (C), ppm

^{19}F NMR (DMSO-d6, 377MHz): δ = -120.8, -137.4, -160.9 ppm

HRMS: ESI$^+$, *m/z* 861.3571 [M+H]$^+$, Error: 0.27 ppm

SYNTHESIS OF INACTIVE CONTROLS

Synthesis of *tert*-butyl 4-fluoro-4-((4-(5-(3-methyl-2,4-dioxotetrahydropyrimidin-1(2H)-yl)pyridin-2-yl)piperazin-1-yl)methyl)piperidine-1-carboxylate

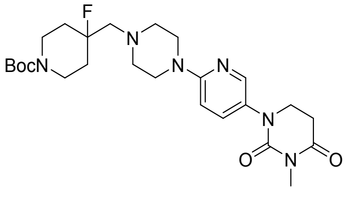

MeCN (3 mL) was added to a flask with the SM (130 mg, 0.26 mmol, 1 eq) and Cs_2CO_3 (170 mg, 0.52 mmol, 2.0 eq), followed by the addition of MeI (18 µL, 0.29 mmol, 1.1 eq). After 4h, the reaction was evaporated on celite and purified via flash column chromatography (DCM/MeOH 0 -> 5% + 0.1 % NH4OH). This delivered the product (120 mg) in 92 % yield.

^{1}H NMR ($CDCl_3$, 400MHz): δ = 1.46 (s, 9H), 1.47-1.67 (m, 2H), 1.90-1.99 (m, 2H), 2.52 (d, J= 23.1 Hz, 2H), 2.61-2.68 (m, 4H), 2.87 (t, J= 6.7 Hz, 2H), 3.11 (t, J= 11.6 Hz, 2H), 3.22 (s, 3H), 3.50-3.57 (m, 2H), 3.73 (t, J= 6.7 Hz, 2H), 3.89 (br s, 2H), 6.64 (d, J= 9.1 Hz, 1H), 7.41 (dd, J= 2.8; 9.0 Hz, 1H), 8.07 (d, J= 2.6 Hz, 1H) ppm.

^{13}C NMR ($CDCl_3$, 100 MHz): δ = 28.0 (CH3), 28.6 (CH3), 32.2 (CH2), 33.3 (d, ^{2}J= 21.8 Hz, CH2), 39.6 (CH2, via HSQC), 44.6 (CH2), 45.5 (CH2), 54.6 (d, J= 2.5 Hz, CH2), 65.6 (d, ^{2}J= 20.9 Hz, CH2), 79.7 (C), 95.4 (d, ^{1}J= 172.5 Hz, C), 106.9 (CH), 129.2 (C), 135.4 (CH), 144.7 (CH), 153.7 (C), 154.9 (C), 158.1 (C), 169.5 (C) ppm.

^{19}F NMR ($CDCl_3$, 370 MHz): δ = -161.2 ppm

HRMS: ESI$^+$, *m/z* 505.2959 [M+H]$^+$, Error: 5.13 ppm

Synthesis of inactive PROTAC SK5527-CRBi

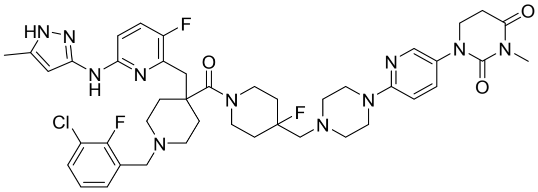

Following the general PROTAC synthesis procedure, 41 mg of the product was obtained with a yield of 48%.

^{1}H NMR (DMSO-d6, 400MHz): δ = 1.49-1.70 (m, 4H), 1.81-1.94 (m, 2H), 2.05-2.22 (m, 7H), 2.53-2.65 (m, 8H), 2.82 (t, J= 6.7 Hz, 2H), 2.96 (s, 2H), 3.03 (s, 3H), 3.16 (br s, 2H), 3.40-3.53 (m, 6H), 3.67 (t, J= 6.7 Hz, 2H), 4.06-4.17 (m, 2H), 6.11 (br s, 1H), 6.83 (d, J= 9.1 Hz, 1H), 7.02 (br s, 1H), 7.15 (t, J= 7.8 Hz, 1H), 7.28-7.55 (m, 4H), 8.06 (d, J= 2.6 Hz, 1H), 8.96 (s, 1H), 11.70 (s, 1H) ppm.

^{13}C NMR (DMSO-d6, 100MHz): δ = 10.8 (CH3), 27.3 (CH3), 31.4 (CH2), 33.1 (d, ^{2}J= 21.6 Hz, CH2), 34.3 (CH2), 38.3 (CH2), 41.7 (CH2, via HSQC), 43.9 (CH2), 45.0 (CH2), 45.5 (C), 50.5 (CH2), 54.0 (CH2), 54.8 (CH2), 64.2 (d, ^{2}J= 20.2 Hz, CH2), 94.1 (CH), 95.9 (d, J= 171.6 Hz, C), 106.6 (CH), 109.0 (CH), 119.5 (d, ^{2}J= 18.2 Hz, C), 125.0 (2x CH), 127.1

(d, ^2J= 14.2 Hz, C), 129.2 (CH), 129.6 (C), 130.1 (CH), 135.4 (CH), 137.9 (C, via HMBC), 141.4 (d, ^2J= 16.7 Hz, C), 144.8 (CH), 149.9 (C), 151.0 (C), 151.6 (d, $^1J(C,F)$ = 240.9 Hz, C), 153.1 (C), 155.7 (d, ^1J= 247.1 Hz, C), 157.2 (C), 169.8 (C), 171.7 (C), ppm

^{19}F NMR (DMSO-d6, 377MHz): δ = -120.8, -138.4, -156.1 ppm

HRMS: ESI$^+$, *m/z* 862.3893 [M+H]$^+$, Error: 0.38 ppm

Synthesis of 1-(*tert*-butyl) 4-methyl 4-((6-((1-(tert-butyl)-3-methyl-1H-pyrazol-5-yl)(methyl)amino)-3-fluoropyridin-2-yl)methyl)piperidine-1,4-dicarboxylate

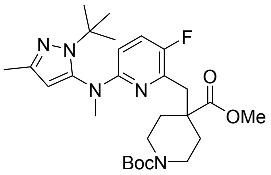

MeCN (10 mL) was added to a flask with 1-(*tert*-butyl) 4-methyl 4-((6-((1-(tert-butyl)-3-methyl-1H-pyrazol-5-yl)amino)-3-fluoropyridin-2-yl)methyl) piperidine-1,4-dicarboxylate (740 mg, 1.5 mmol, 1.0 eq) and Cs_2CO_3 (958 mg, 2.9 mmol, 2.0 eq), followed by the addition of MeI (0.1 mL, 1.6 mmol, 1.1 eq). After 16h, the reaction was evaporated on celite and purified via flash column chromatography (DCM/MeOH 0 -> 4% + 0.1 % NH4OH). This delivered the product (500 mg) in 66 % yield.

^{1}H NMR ($CDCl_3$, 400MHz): δ = 1.44 (s, 9H), 1.51 (s, 9H), 1.52-1.59 (m, 2H), 2.06-2.15 (m, 2H), 2.24 (s, 3H), 2.98-3.08 (m, 4H), 3.20 (s, 3H), 3.69 (s, 3H), 3.79 (br s, 2H), 5.79 (s, 1H), 5.98 (dd, J= 2.8, 9.0 Hz, 1H), 7.03 (t, J= 8.9 Hz, 1H) ppm.

^{13}C NMR ($CDCl_3$, 100MHz): δ = 14.4 (CH3), 28.6 (CH3), 30.1 (CH3), 33.3 (CH2), 39.6 (CH3), 39.7 (CH2), 40.5 (CH2), 41.6 (CH2), 45.7 (C), 51.9 (CH3), 59.7 (C), 79.6 (C), 103.3 (CH), 108.1 (d, ^3J= 3.6 Hz, CH), 124.5 (d, ^2J= 22.4 Hz, CH), 141.5 (d, ^2J= 16.5 Hz, C), 144.6 (C), 146.6 (C), 152.3 (d, ^1J= 243.6 Hz, C), 154.6 (C), 155.0 (C), 175.6 (C) ppm

^{19}F NMR (DMSO-d6, 377MHz): δ = -137.8 ppm

Synthesis of methyl 4-((6-((1-(*tert*-butyl)-3-methyl-1H-pyrazol-5-yl)(methyl)amino)-3-fluoropyridin-2-yl)methyl)-1-(3-chloro-2-fluorobenzyl)piperidine-4-carboxylate

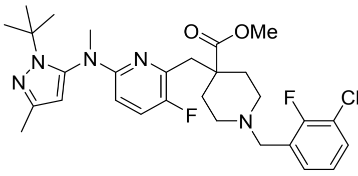

The Boc-protected intermediate (0.50 g, 0.97 mmol, 1.0 eq) was treated with TFA (3 mL) in DCM (6 mL) for 1 h. Next, the reaction was fully evaporated, redissolved in DMF (10 mL) and cooled down to 0°C. K_2CO_3 (0.67 g, 4.9 mmol, 5 eq) was slowly added, followed by 3-chloro-2-fluoro-benzyl bromide (0.24 g, 1.1 mmol, 1.1 eq). After 2h, water was added and extracted with DCM. The organic layers were washed with brine, dried over Na_2SO_4, filtered and evaporated. Flash column chromatography (DCM/MeOH 0 -> 5%) delivered the product (430 mg) in 79 % yield.

^{1}H NMR ($CDCl_3$, 400MHz): δ = 1.51 (s, 9H), 1.65-1.75 (m, 2H), 2.11-2.24 (m, 4H), 2.24 (s, 3H), 2.68-2.76 (m, 2H), 3.01 (s, 2H), 3.20 (s, 3H), 3.53 (s, 2H), 3.68 (s, 3H), 5.79 (s, 1H), 5.96 (dd, J= 2.8, 8.9 Hz, 1H), 7.00-7.08 (m, 2H), 7.25-7.31 (m, 2H) ppm.

^{13}C NMR ($CDCl_3$, 100MHz): δ = 14.4 (CH3), 30.1 (CH3), 33.5 (CH2), 39.6 (CH3), 40.0 (CH2), 45.5 (C), 50.8 (CH2), 51.7 (CH3), 55.7 (CH2), 59.2 (C), 103.3 (CH), 107.9 (d, ^3J= 3.8 Hz, CH), 121.0 (d, ^2J= 18.3 Hz, C), 124.3 (CH), 124.4 (d, ^2J= 17.9 Hz, CH), 127.2 (d, ^2J= 14.5 Hz, C), 129.3 (CH), 129.7 (d, ^3J= 4.1 Hz, CH), 141.8 (d, ^2J= 16.5 Hz, C), 144.7 (C), 146.6 (C), 152.3 (d, ^1J= 243.7 Hz, C), 154.6 (C), 156.8 (d, ^1J= 248.5 Hz, C), 176.0 (C) ppm

Carbons from phenyl ring

^{19}F NMR (DMSO-d6, 377MHz): δ = -119.9, -140.6 ppm

Synthesis of 1-(3-chloro-2-fluorobenzyl)-4-((3-fluoro-6-(methyl(5-methyl-1H-pyrazol-3-yl)amino)pyridin-2-yl)methyl)piperidine-4-carboxylic acid

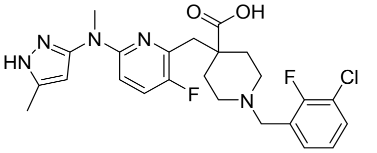

The methyl ester (0.43 g, 0.77 mmol, 1.0 eq) was treated with 2M NaOH (3.85 mL, 7.7 mmol, 10 eq) in MeOH (7.7 mL) for 24h at 60°C. Next, the reaction was cooled down and quenched with 4M HCl until pH = 5 to 6, then water was added and the precipitate filtered and dried *in vacuo*. The intermediate was redissolved in 4M HCl in dioxane and stirred at 95°C for 6h. Finally, the reaction was again cooled down to 0°C, Et_2O added and filtered. This delivered the product (230 mg) in 50 % yield over 2 steps as 3. HCl salt. The product was used as such in the next step, without further characterization.

HRMS: ESI$^+$, *m/z* 490.1810 [M+H]$^+$, Error: 1.20 ppm

Synthesis of inactive PROTAC SK5527-AURi

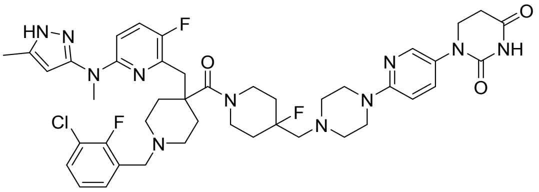

Following the general PROTAC synthesis procedure, 16 mg of the product was obtained with a yield of 19%.

^1H NMR (DMSO-d6, 400MHz): δ = 1.49-1.72 (m, 4H), 1.81-1.94 (m, 2H), 2.05-2.22 (m, 7H), 2.53-2.65 (m, 8H), 2.69 (t, J= 6.7 Hz, 2H), 3.01 (s, 2H), 3.16 (br s, 2H), 3.28 (s, 3H), 3.40-3.53 (m, 6H), 3.69 (t, J= 6.7 Hz, 2H), 4.06-4.17 (m, 2H), 5.89 (s, 1H), 6.83 (d, J= 9.1 Hz, 1H), 7.02 (d, J= 9.0 Hz, 1H), 7.16 (t, J= 7.8 Hz, 1H), 7.28-7.38 (m, 2H), 7.43-7.53 (m, 2H), 8.06 (d, J= 2.6 Hz, 1H), 10.34 (s, 1H), 12.09 (s, 1H) ppm.

^{13}C NMR (DMSO-d6, 100MHz): δ = 10.8 (CH3), 31.1 (CH2), 33.0 (d, ^2J= 21.6 Hz, CH2), 34.4 (CH2), 36.5 (CH3), 38.1 (CH2), 41.7 (CH2, via HSQC), 44.9 (CH2), 45.0 (CH2), 45.2 (C), 50.4 (CH2), 54.0 (CH2), 54.9 (CH2), 64.2 (d, ^2J= 20.7 Hz, CH2), 96.4 (CH), 95.9 (d, J= 171.6 Hz, C), 106.6 (CH), 109.0 (CH), 119.5 (d, ^2J= 17.8 Hz, C), 124.6 (d, ^2J= 20.9 Hz, CH), 125.0 (CH), 127.2 (C), 129.0 (C), 129.2 (CH), 130.1 (CH), 135.4 (CH), 139.0 (C, via HMBC), 141.4 (C, via HMBC), 144.8 (CH), 150.3 (C), 151.6 (d, $^1J(C,F)$ = 240.9 Hz, C), 152.6 (C), 152.6 (C), 155.8 (d, ^1J= 246.8 Hz, C), 157.1 (C), 170.7 (C), 171.7 (C) ppm

^{19}F NMR (DMSO-d6, 377MHz): δ = -120.7, -139.5, -156.1 ppm

HRMS: ESI$^+$, *m/z* 862.3899 [M+H]$^+$, Error: 1.07 ppm

CHARACTERIZATION SPECTRA FOR SELECTED PANEL OF PROTACS

PROTAC 3

^{1}H NMR (DMSO-d6)

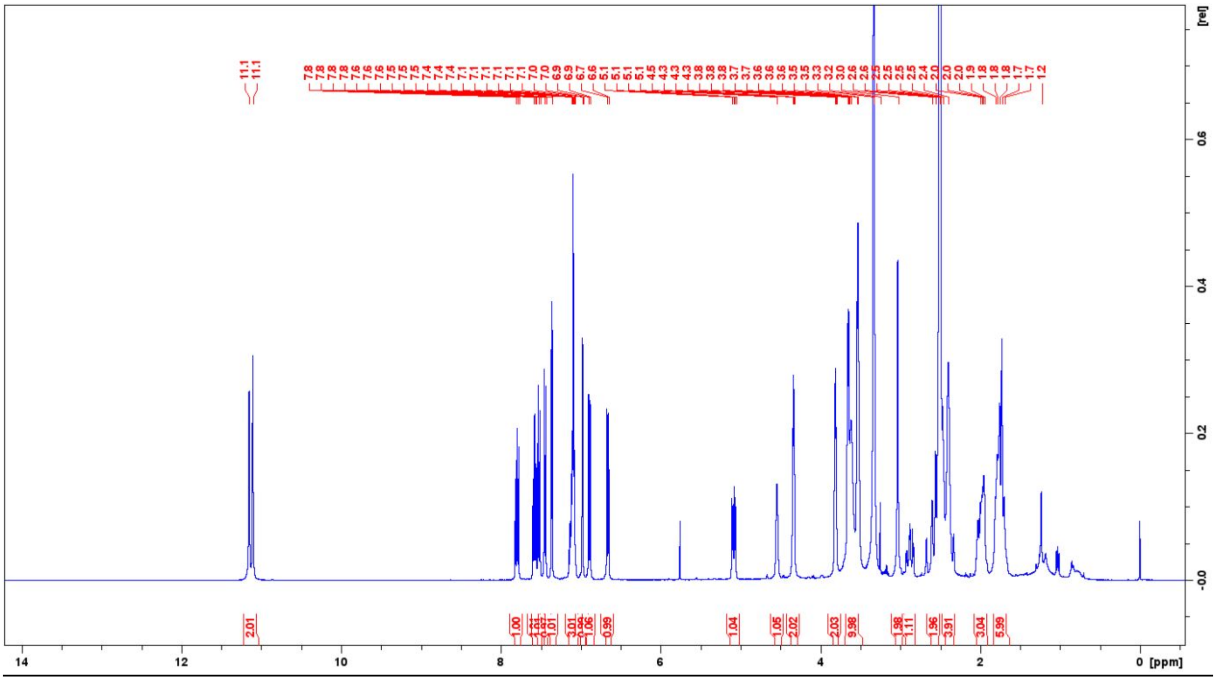

^{13}C NMR (DMSO-d6):

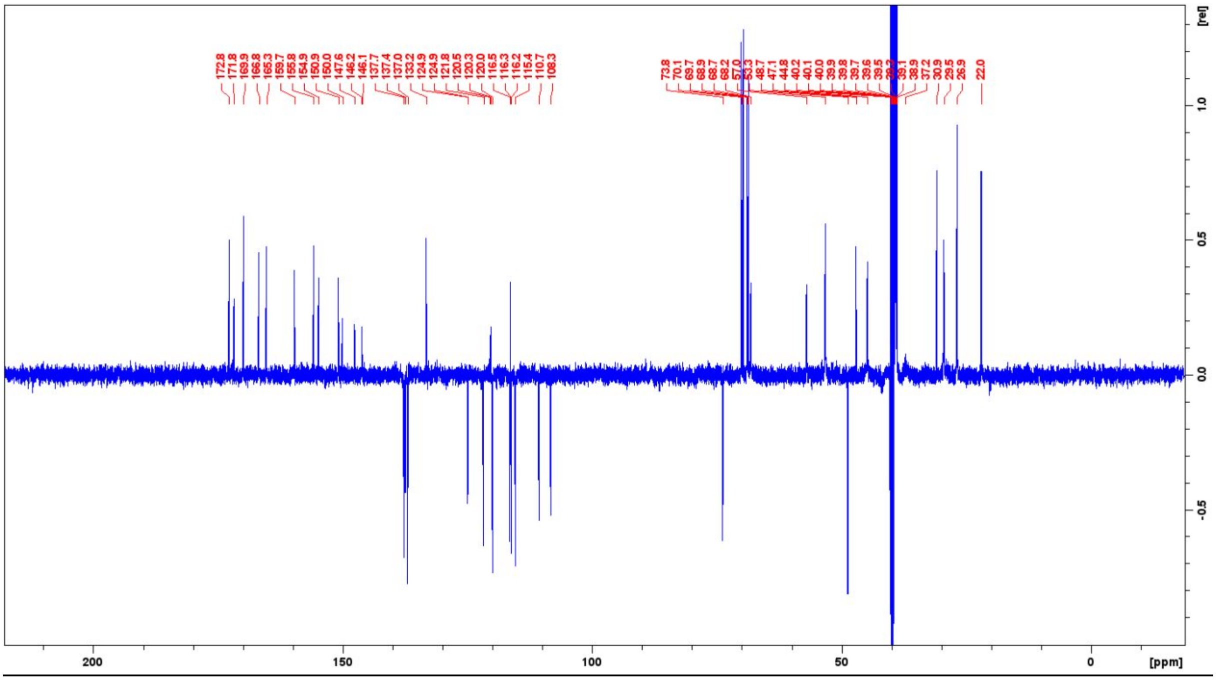

<u>HPLC:</u>

Purity:

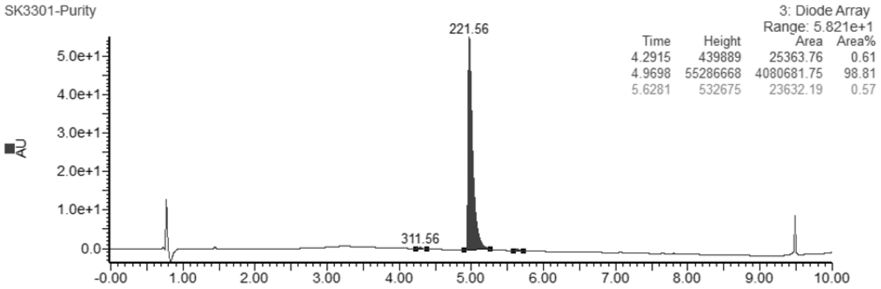

PROTAC 4

^{1}H NMR (DMSO-d6)

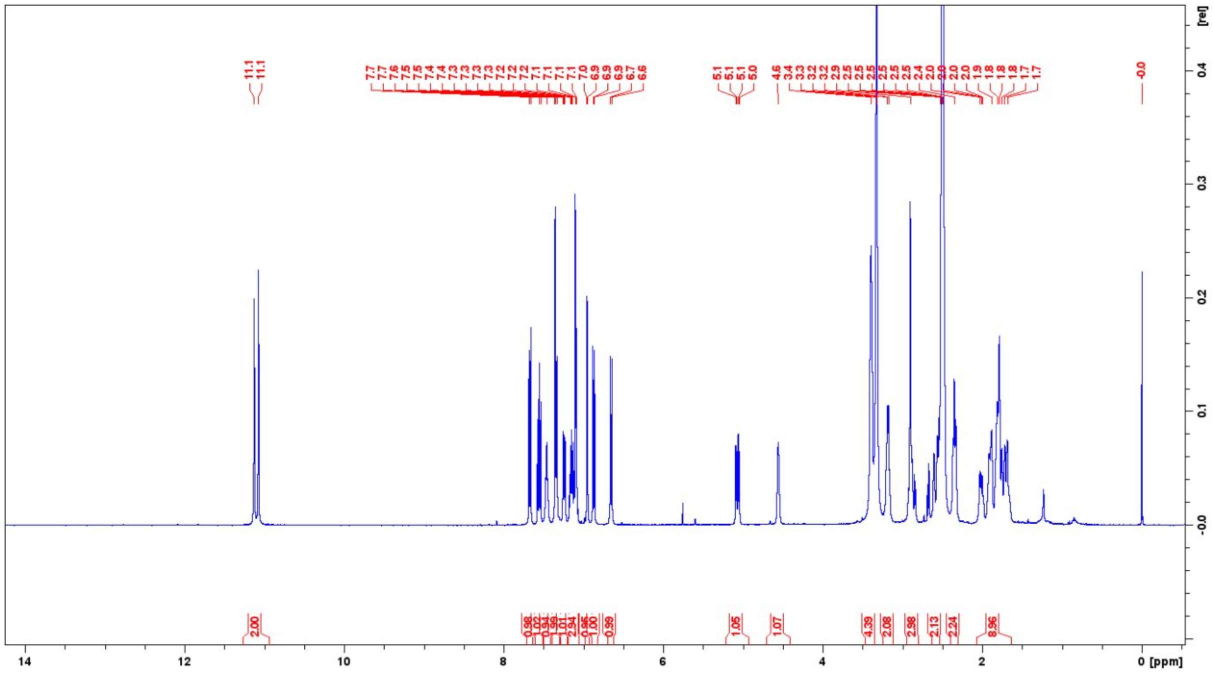

^{13}C NMR (DMSO-d6):

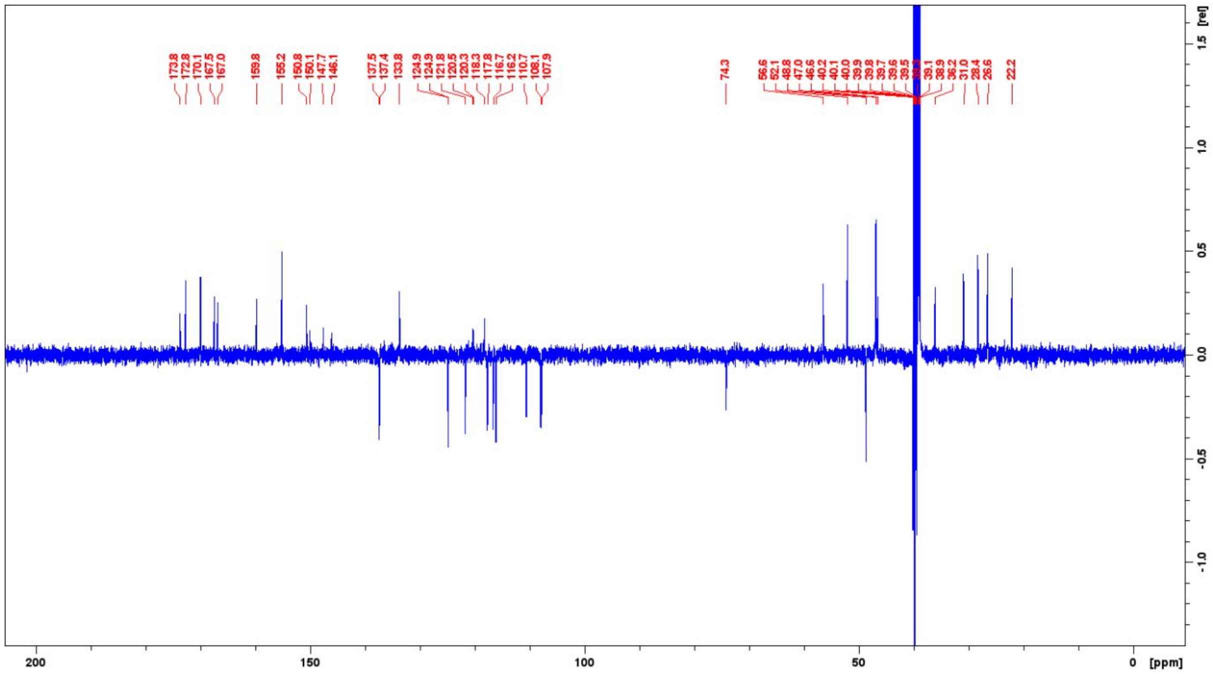

HPLC:

Purity: 98.7%

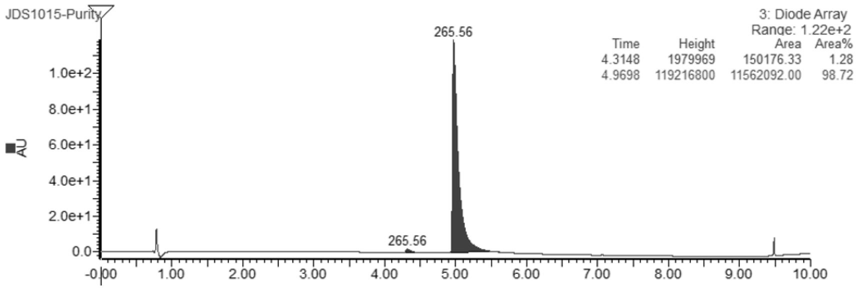

PROTAC 14

^{1}H NMR (DMSO-d6)

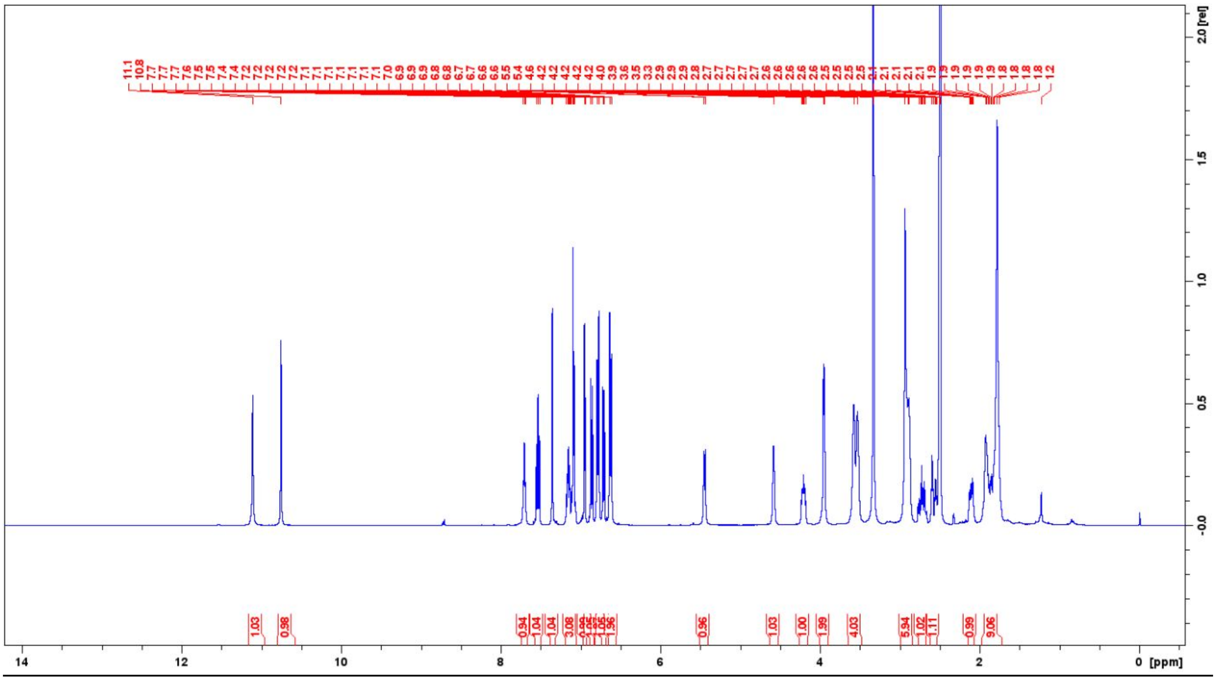

^{13}C NMR (DMSO-d6):

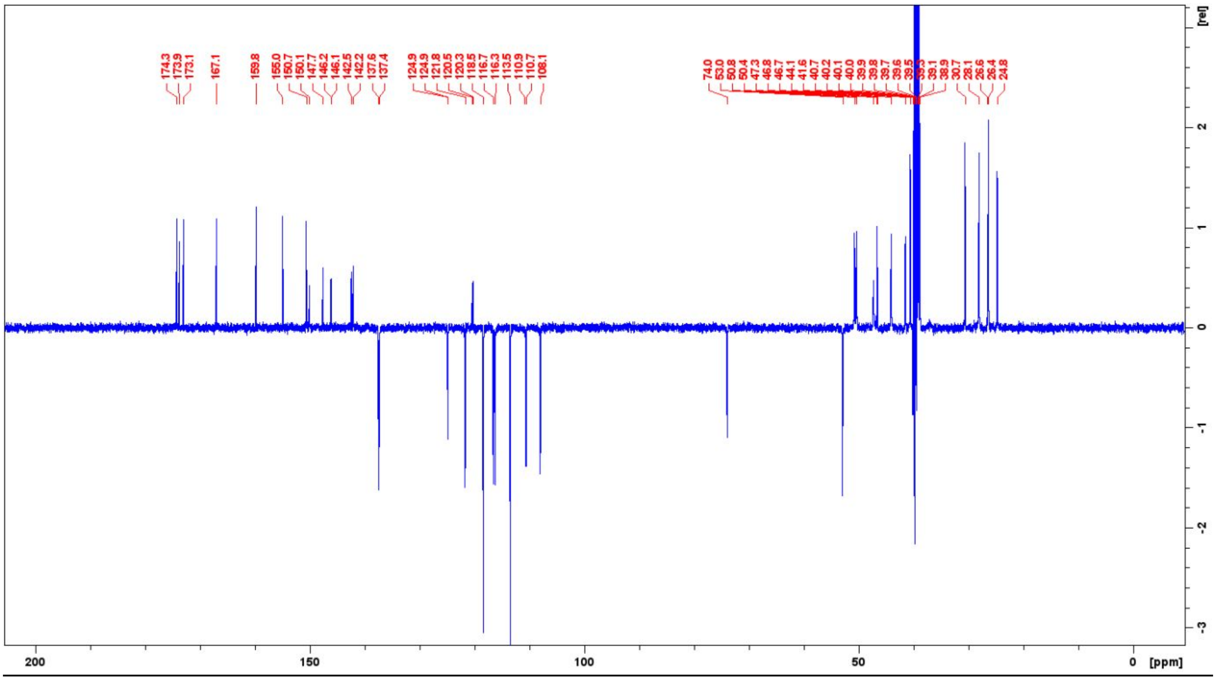

HPLC:

Purity: 96.7%

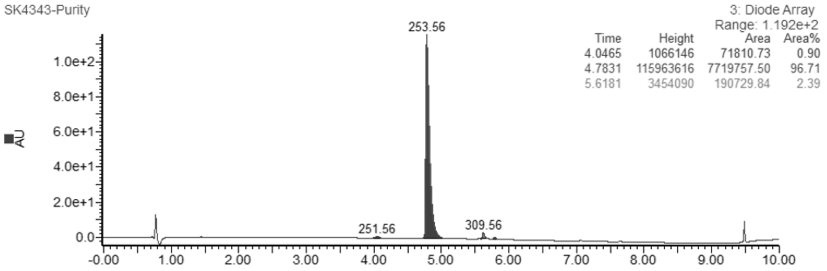

PROTAC 18

^{1}H NMR (DMSO-d6)

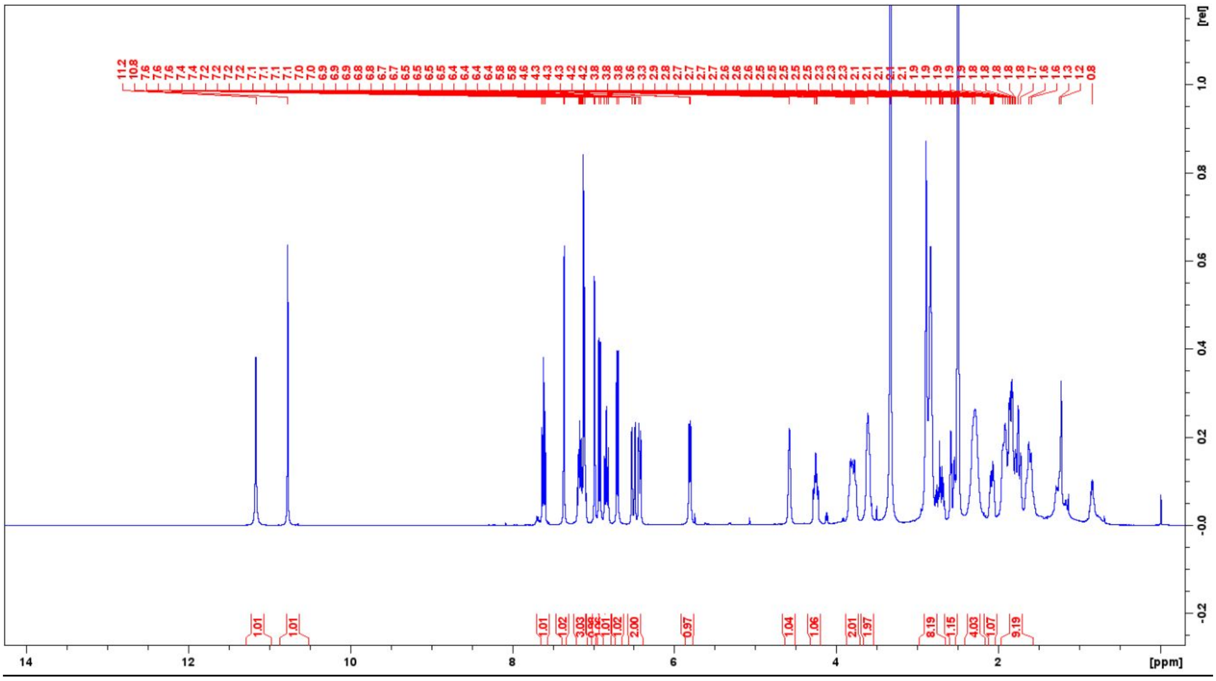

^{13}C NMR (DMSO-d6):

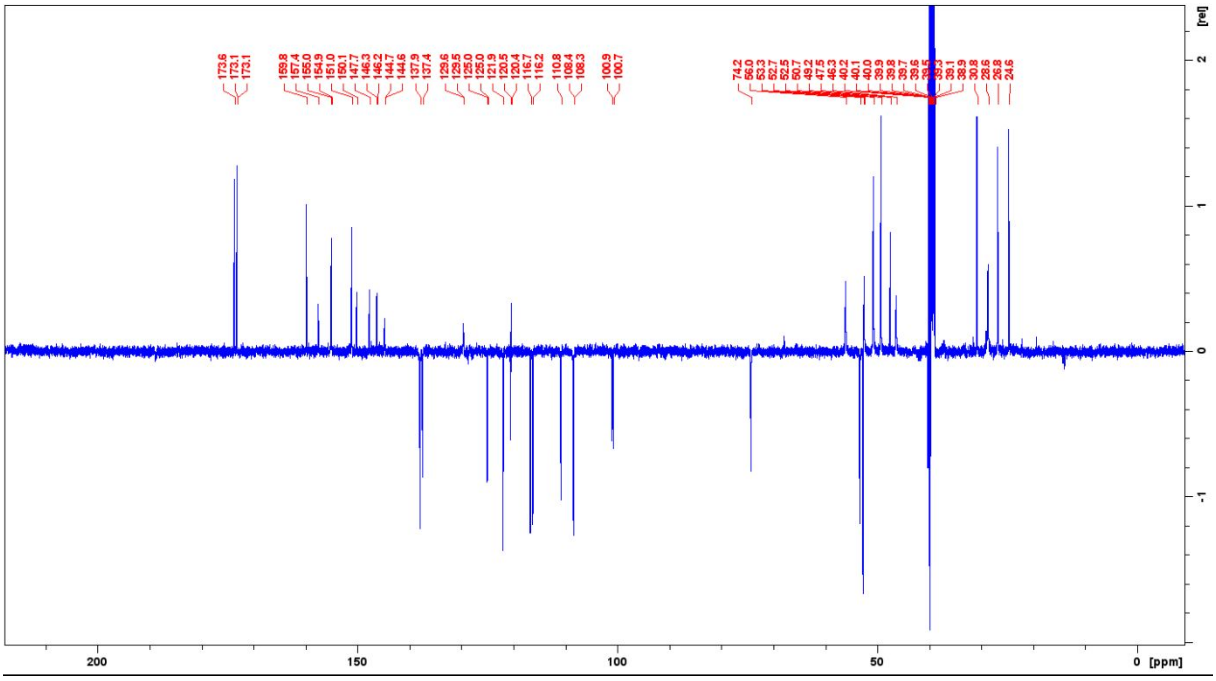

HPLC:

Purity: >99%

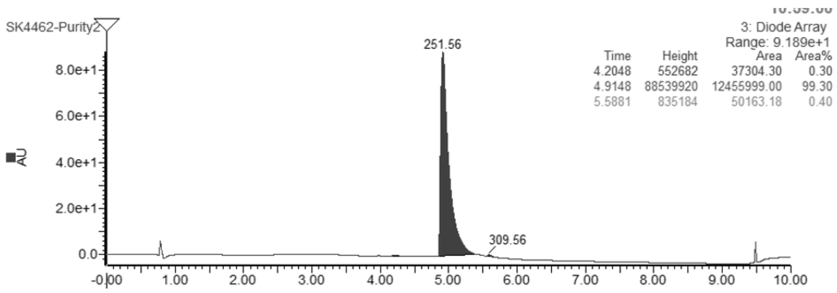

PROTAC 20

<u>^{1}H NMR (DMSO-d6)</u>

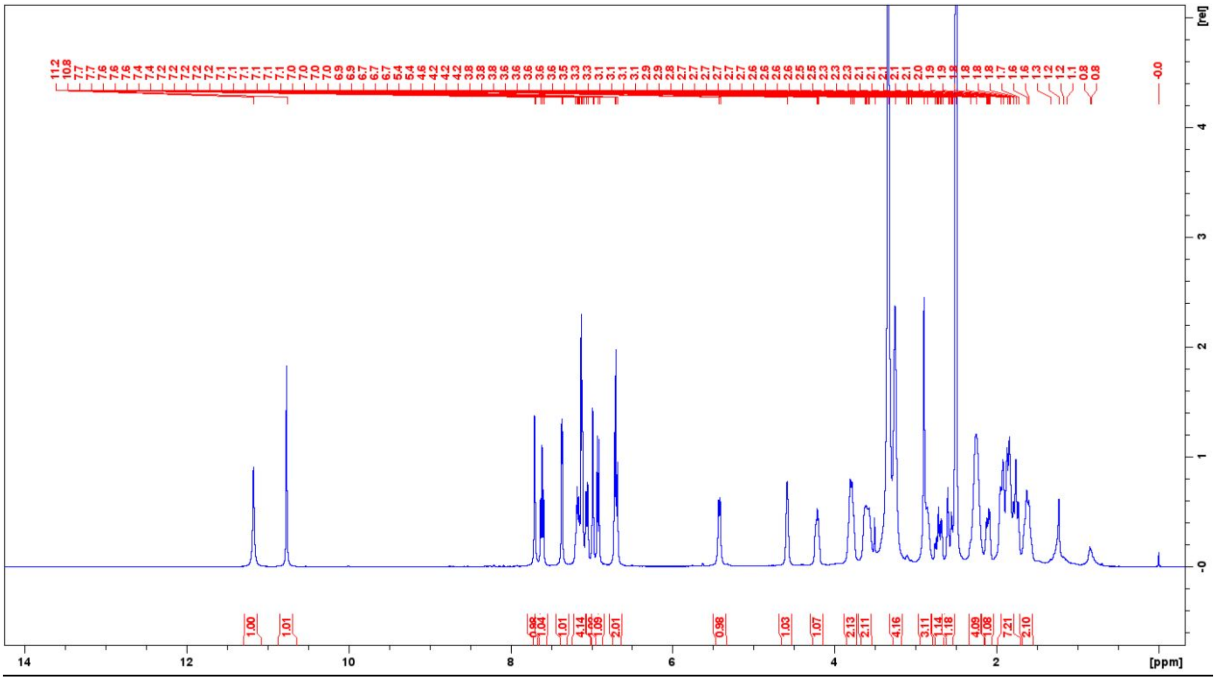

<u>^{13}C NMR (DMSO-d6):</u>

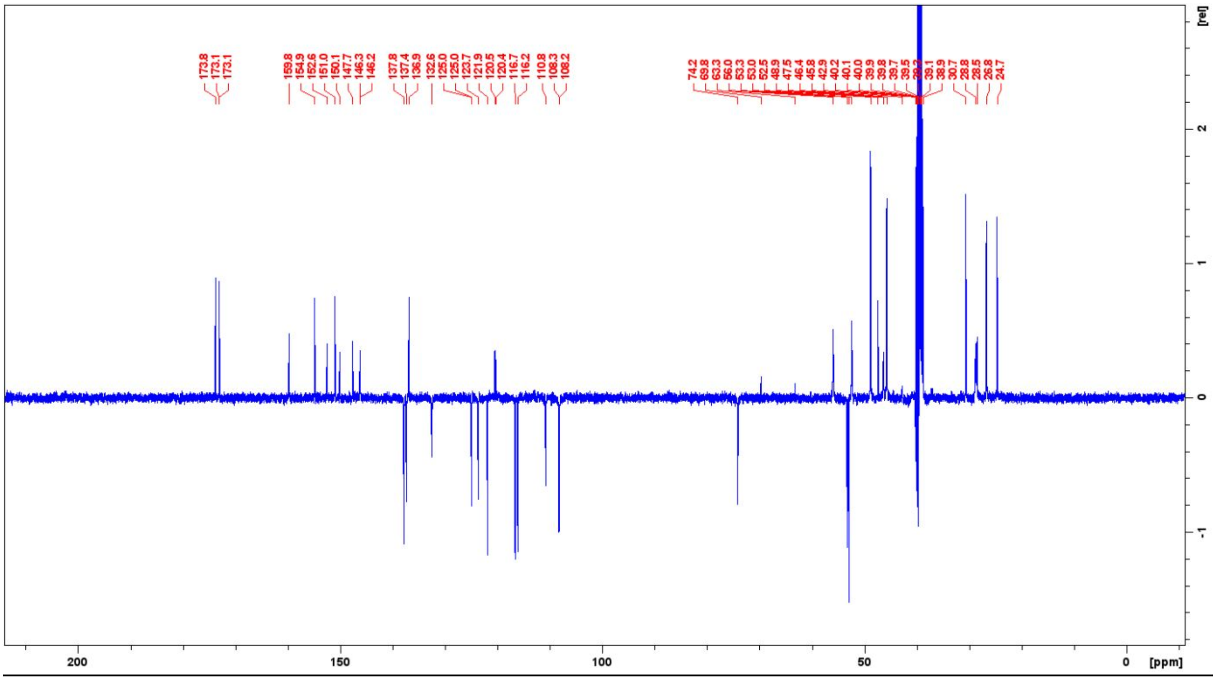

<u>HPLC:</u>

Purity: 99.5%

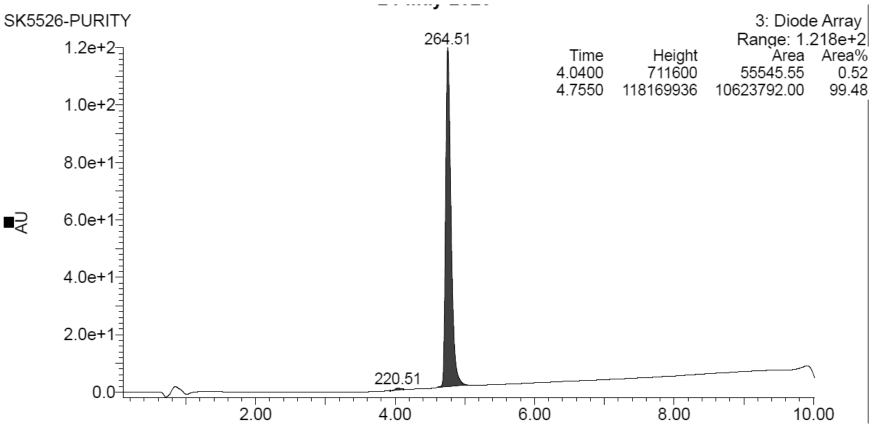

PROTAC 21

^{1}H NMR (DMSO-d6)

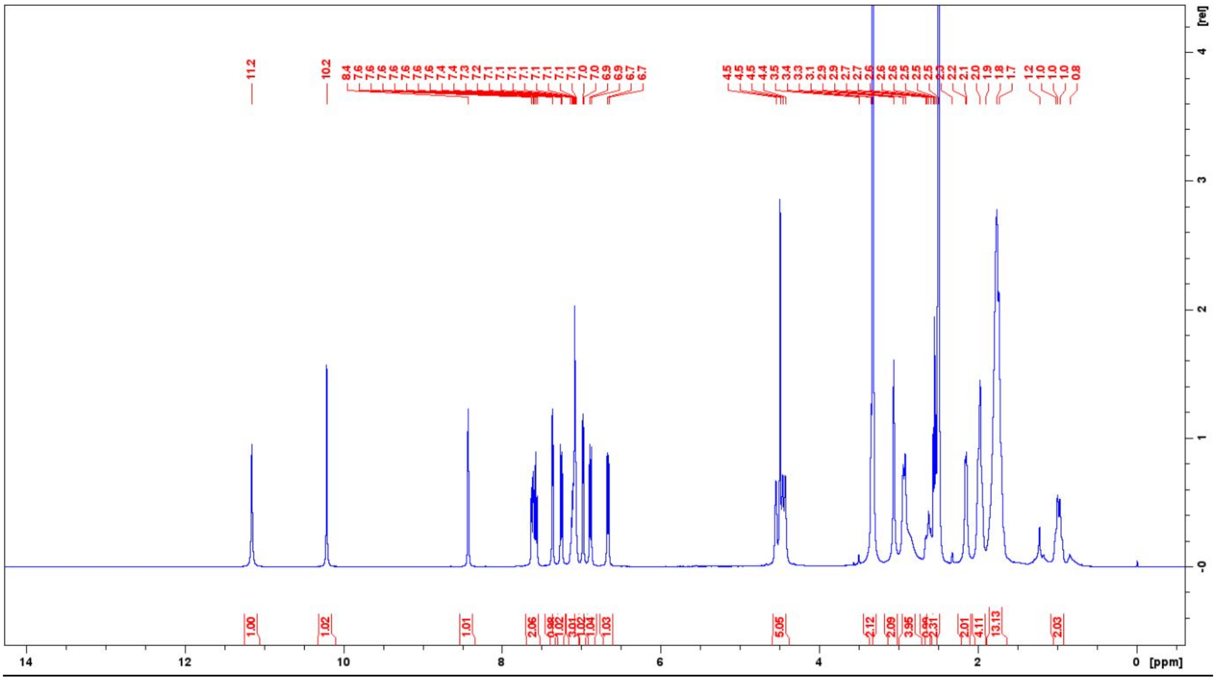

^{13}C NMR (DMSO-d6):

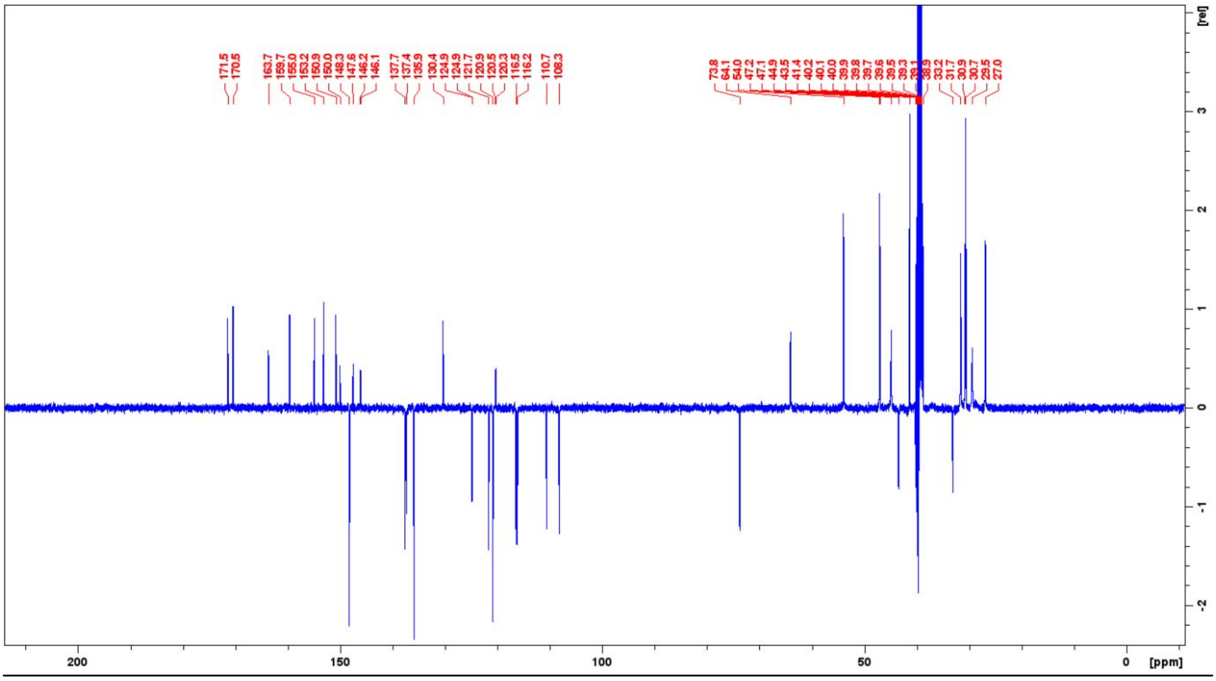

HPLC:

Purity: 99.4%

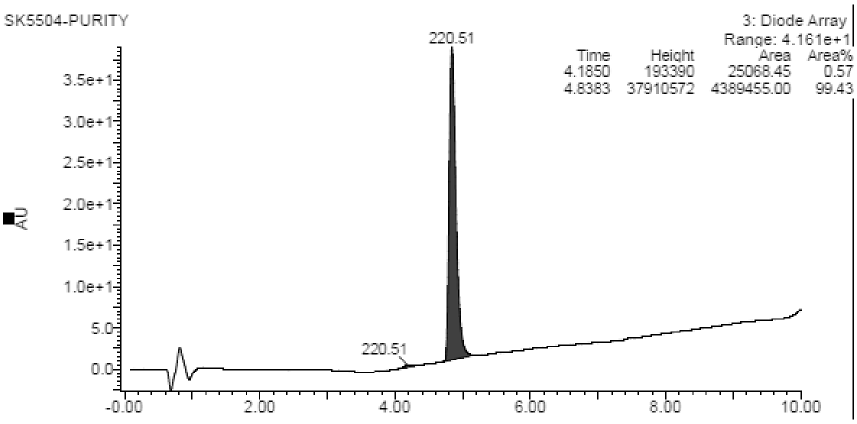

PROTAC 23

<u>^{1}H NMR (DMSO-d6)</u>

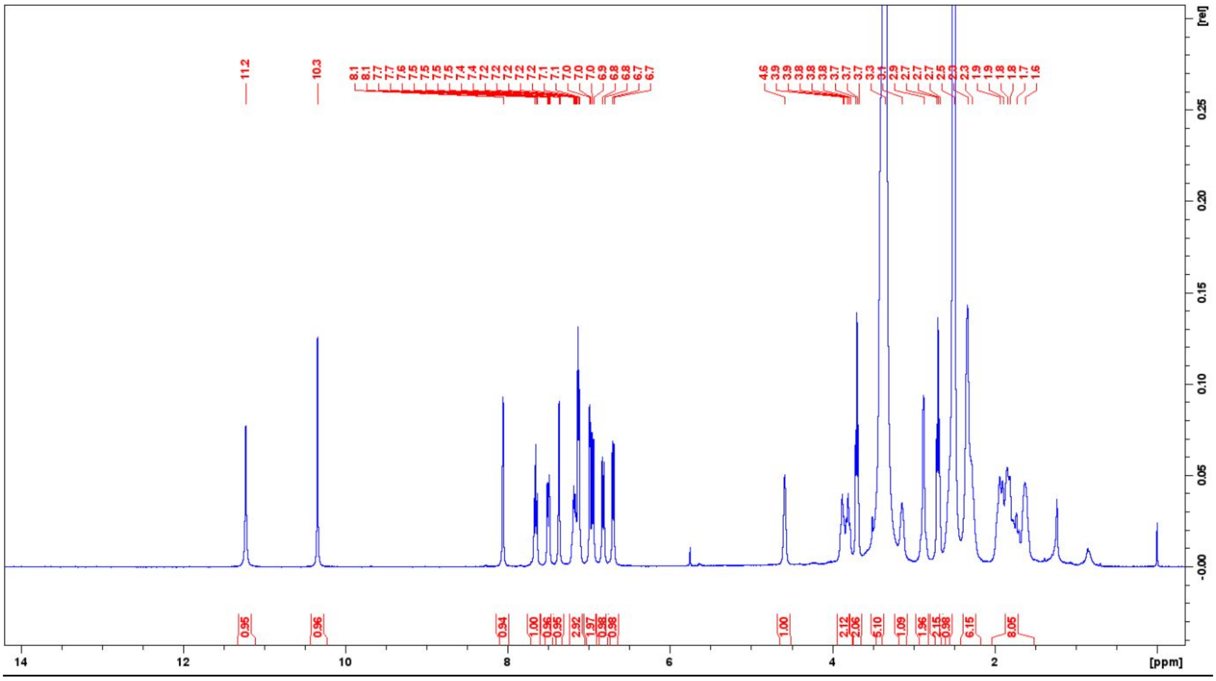

<u>^{13}C NMR (DMSO-d6):</u>

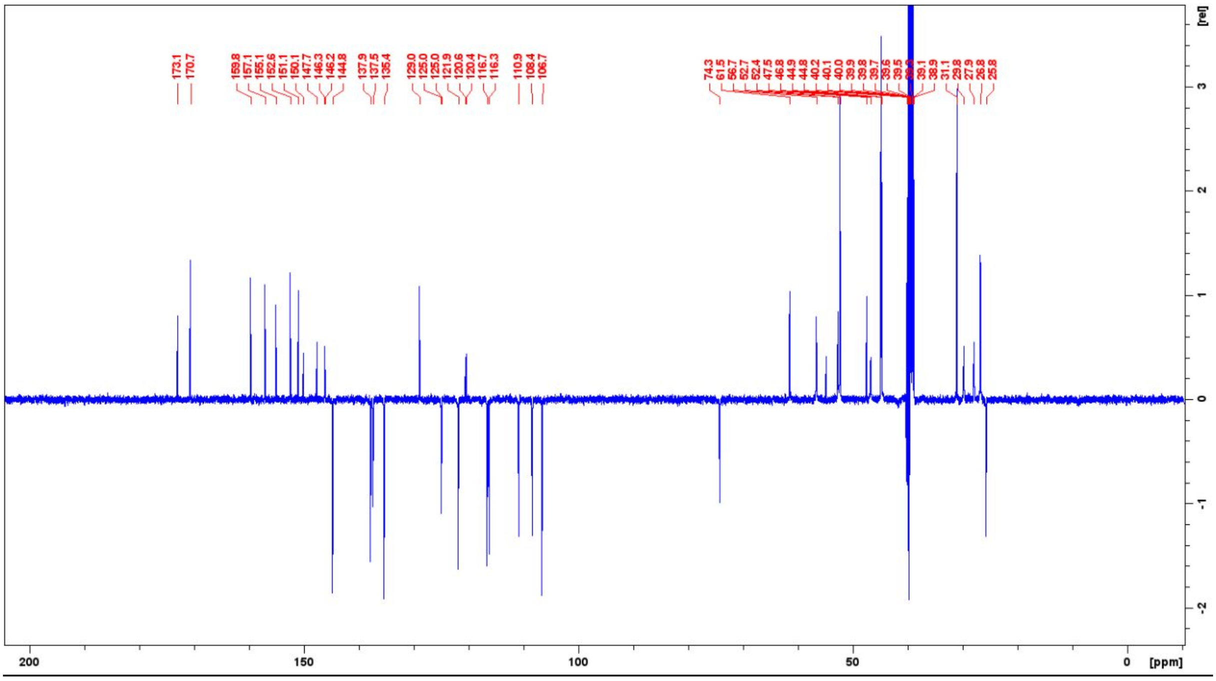

HPLC

Purity: 96.3%

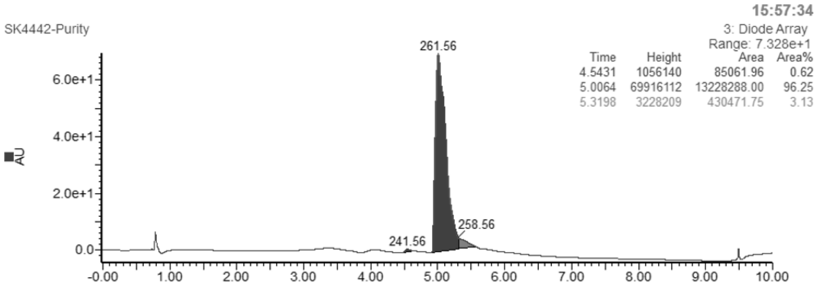

PROTAC 24

^{1}H NMR (DMSO-d6)

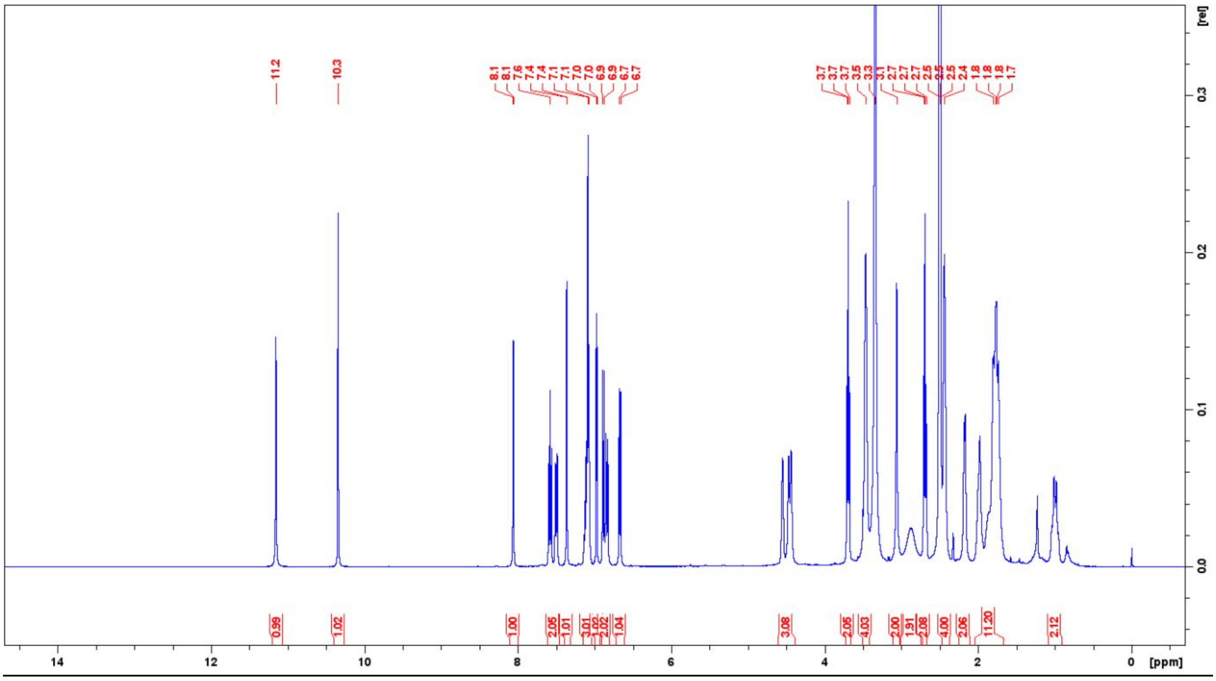

^{13}C NMR (DMSO-d6):

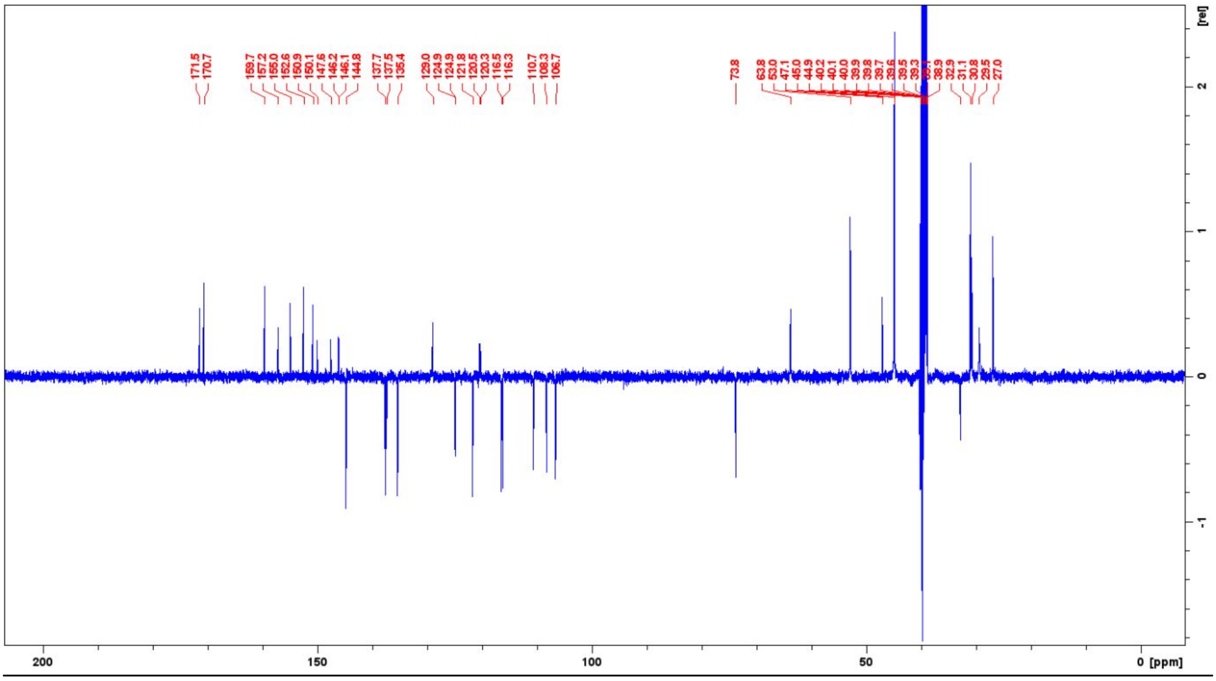

<u>HPLC:</u>

Purity: > 99%

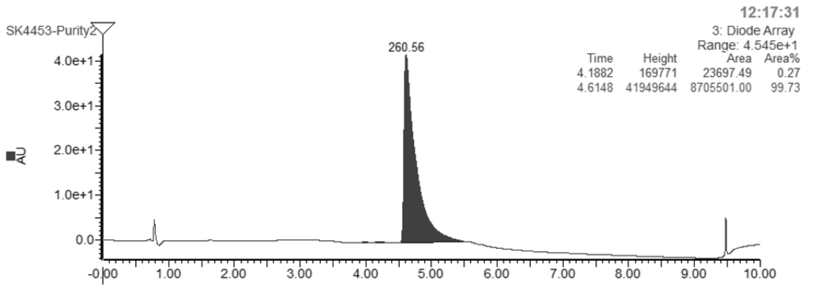

PROTAC 29

^{1}H NMR (DMSO-d6, 70°C)

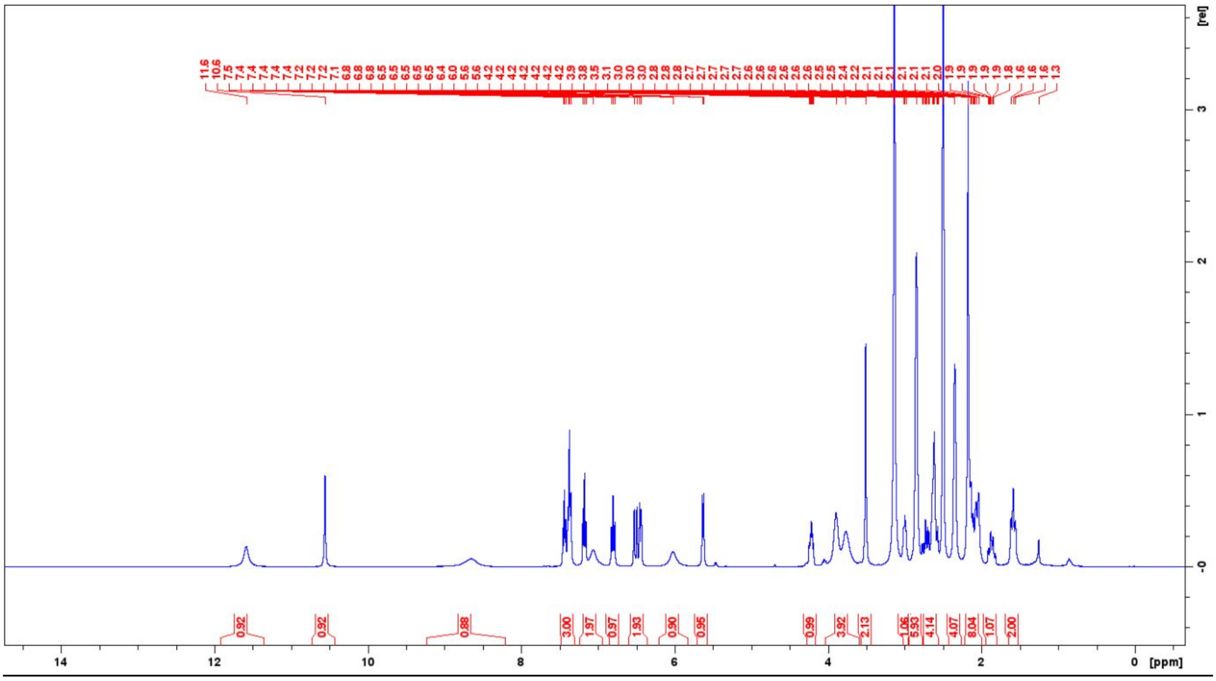

^{13}C NMR (DMSO-d6, 70 °C):

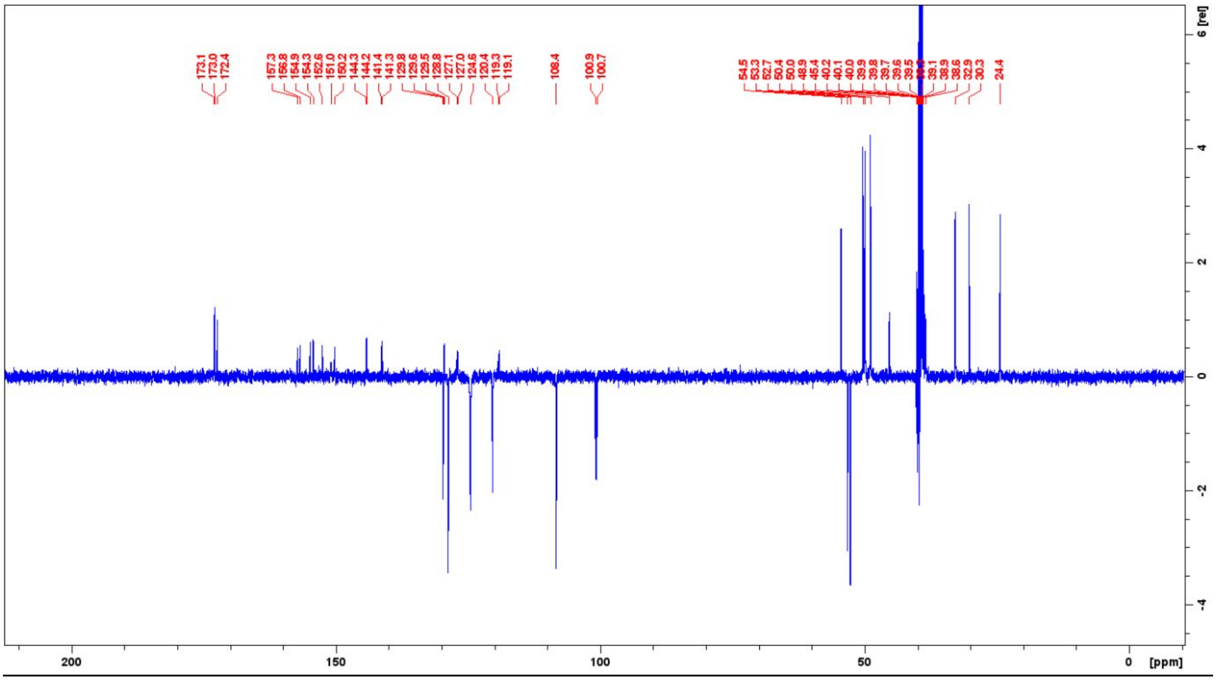

<u>HPLC:</u>

Purity: 97%

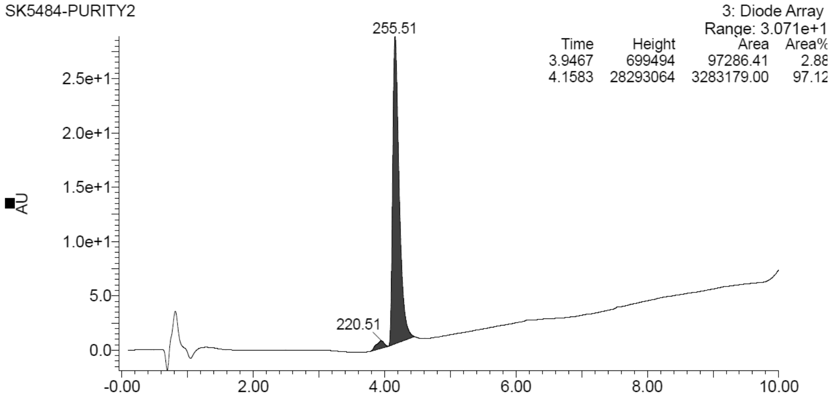

PROTAC 30

^{1}H NMR (DMSO-d6)

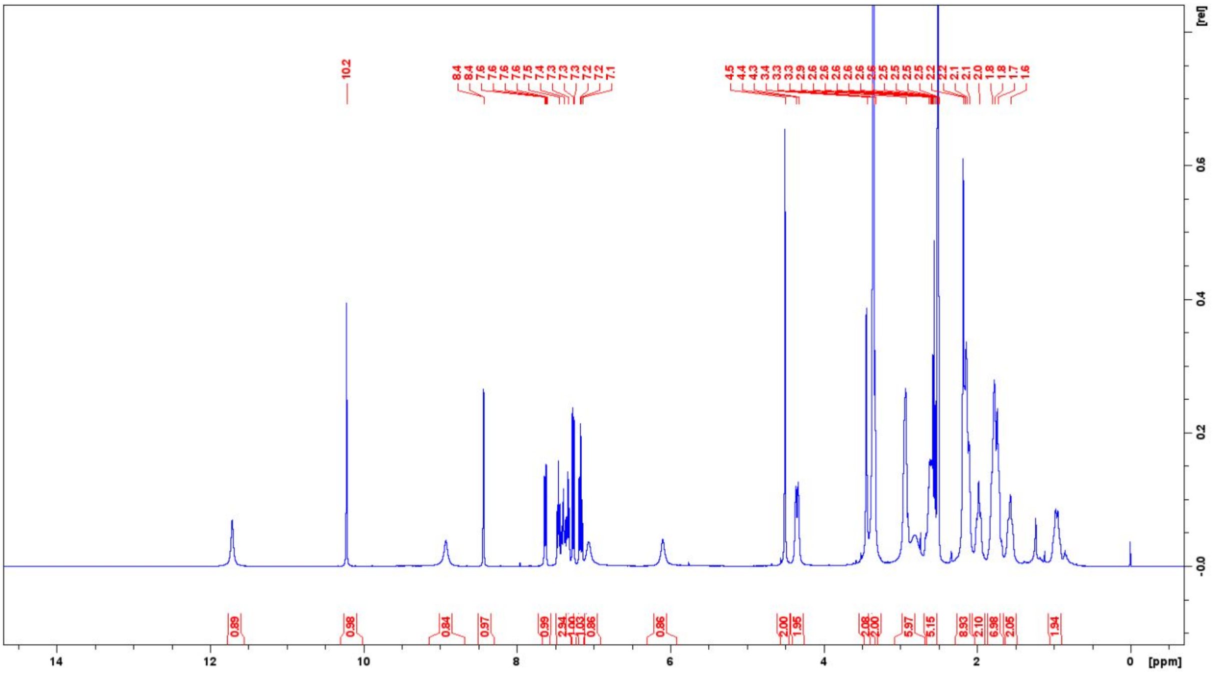

^{13}C NMR (DMSO-d6):

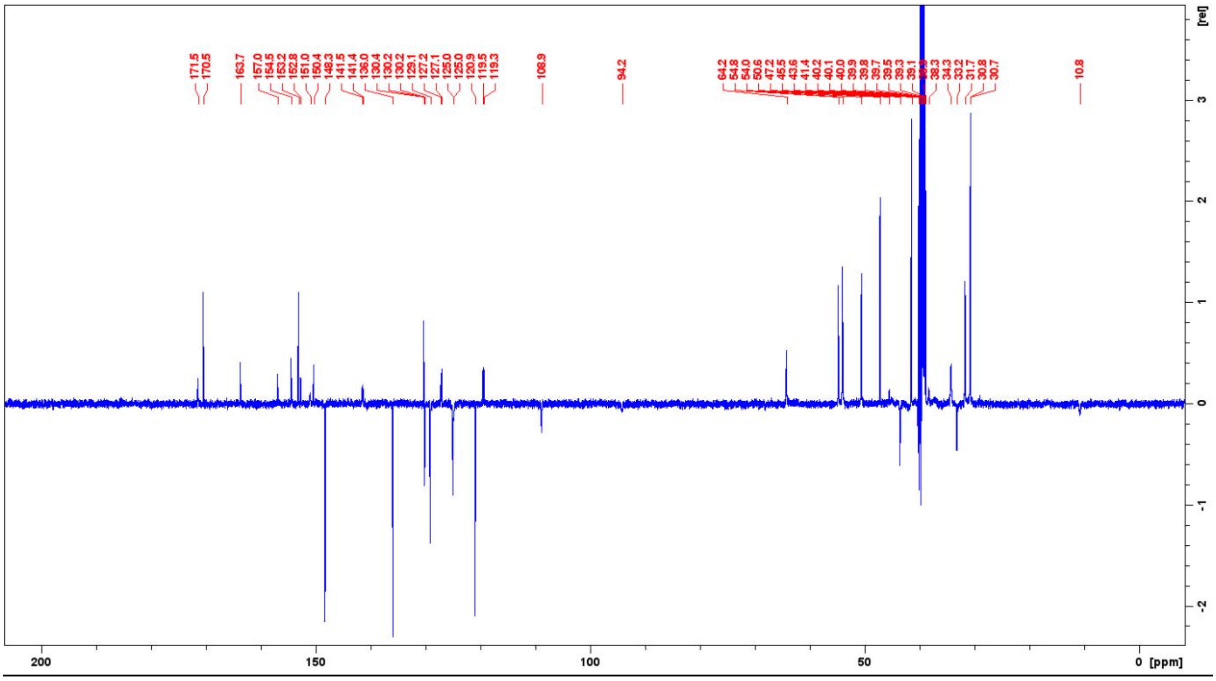

HPLC:

Purity: 95.8%

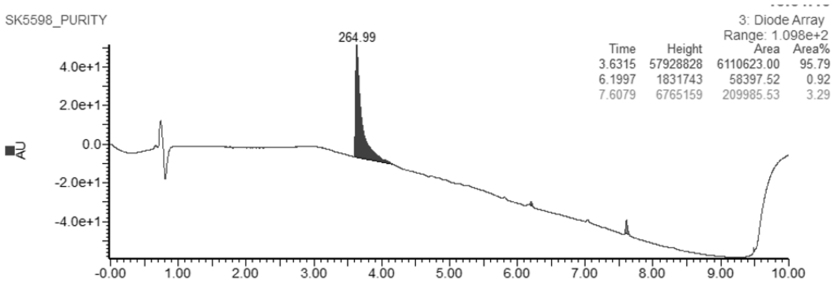

PROTAC 32 (SK4454)

<u>^{1}H NMR (DMSO-d6)</u>

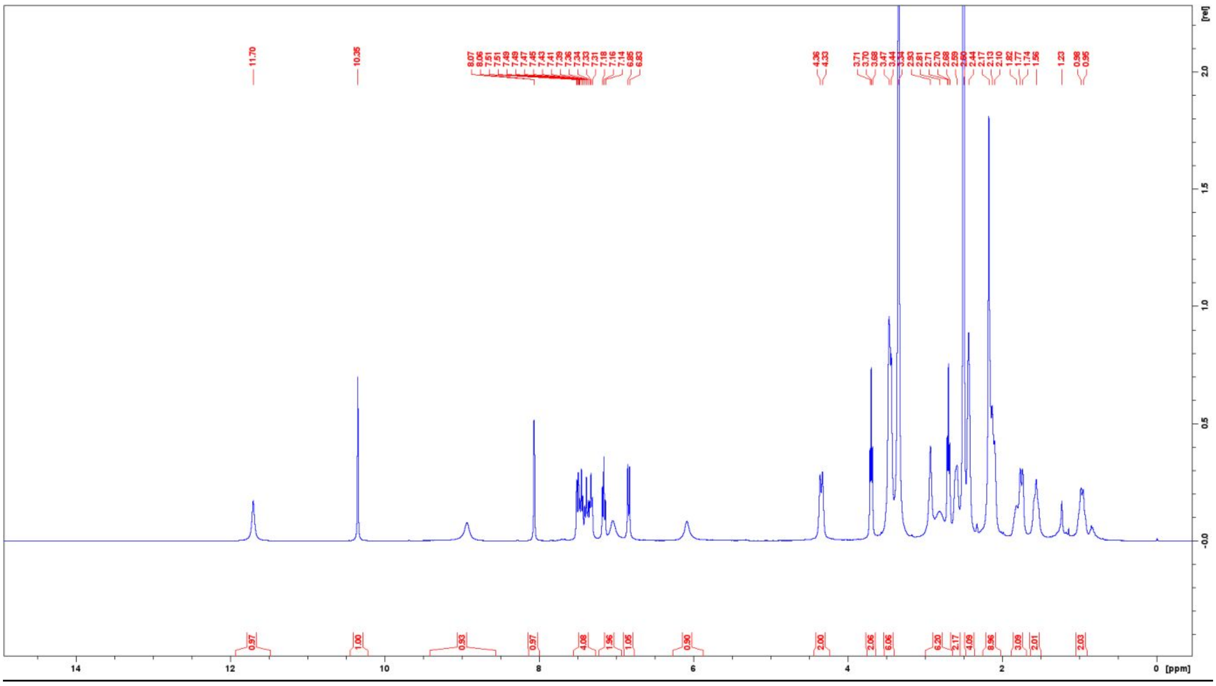

<u>^{13}C NMR (DMSO-d6)</u>

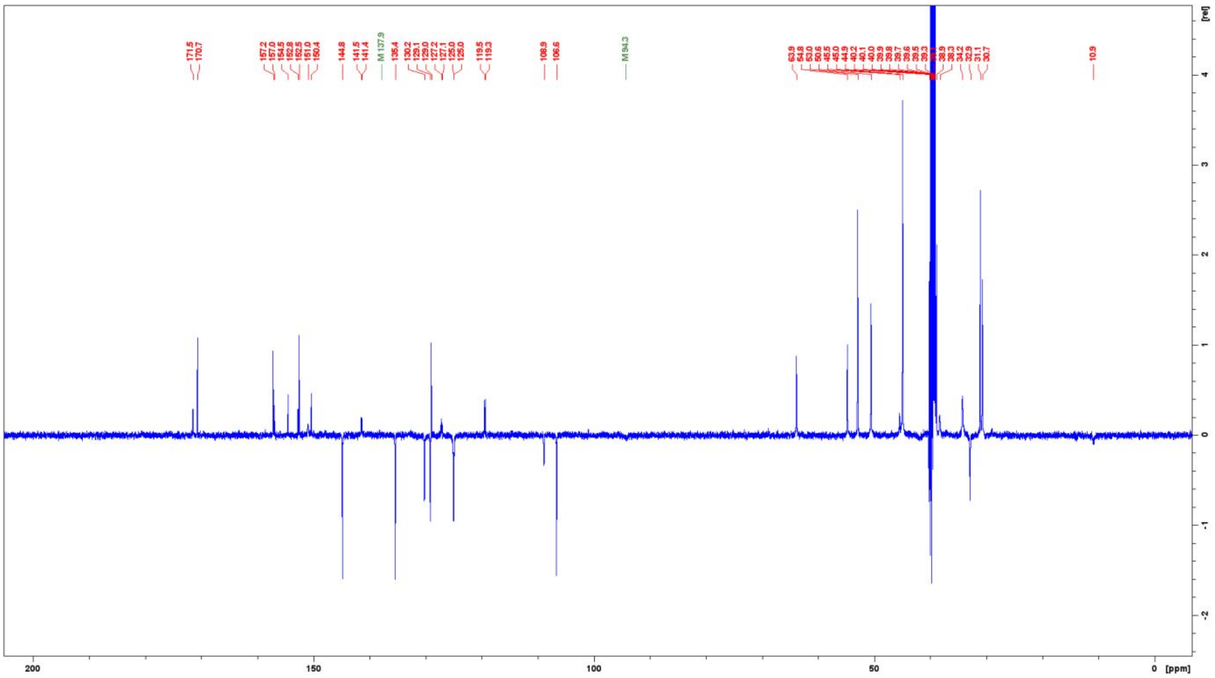

^{19}F NMR (DMSO-d6)

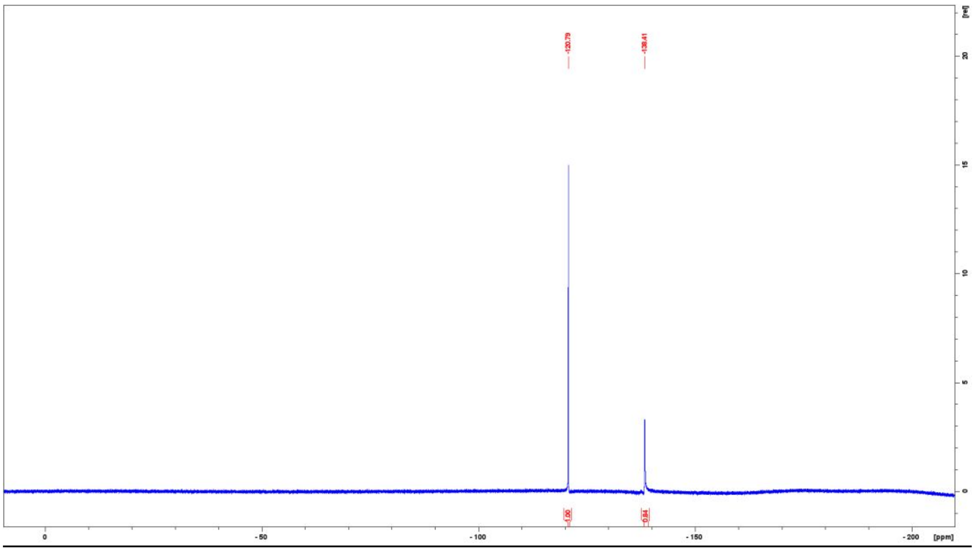

HRMS

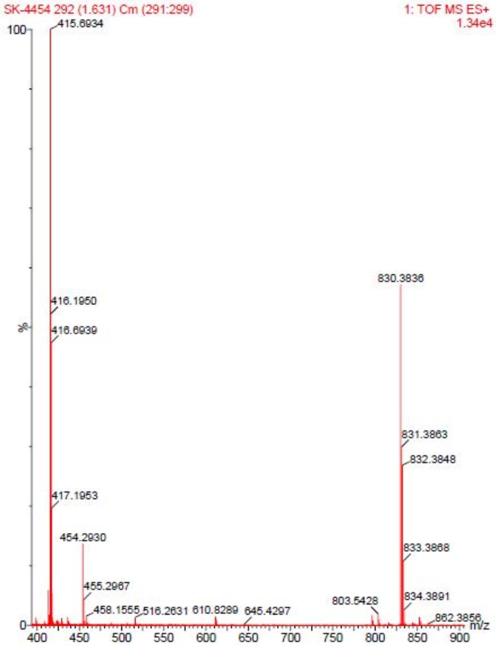

HPLC

Purity: > 99%

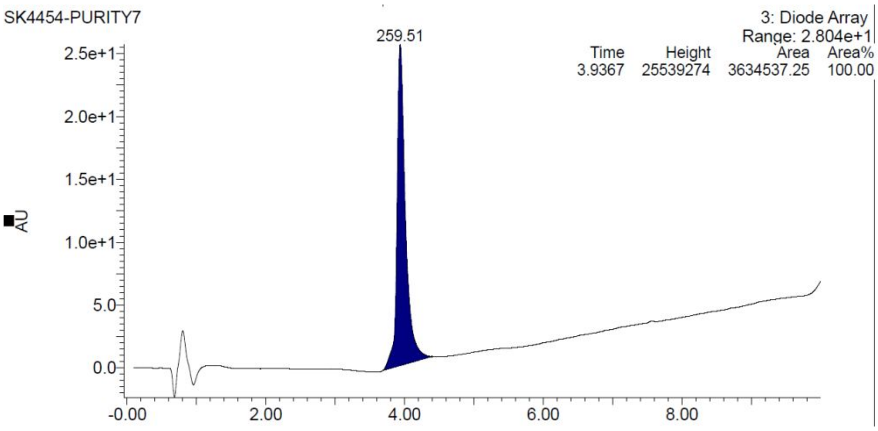

PROTAC 33 (SK5527)

^{1}H NMR (DMSO-d6)

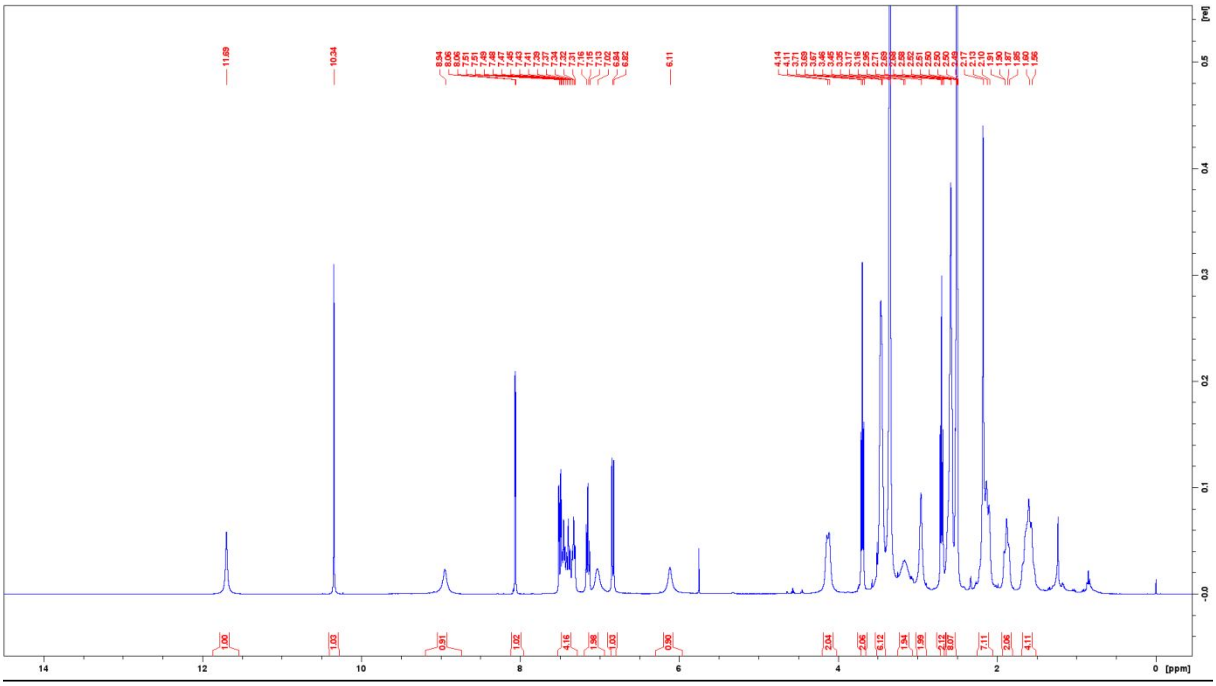

^{13}C NMR (DMSO-d6)

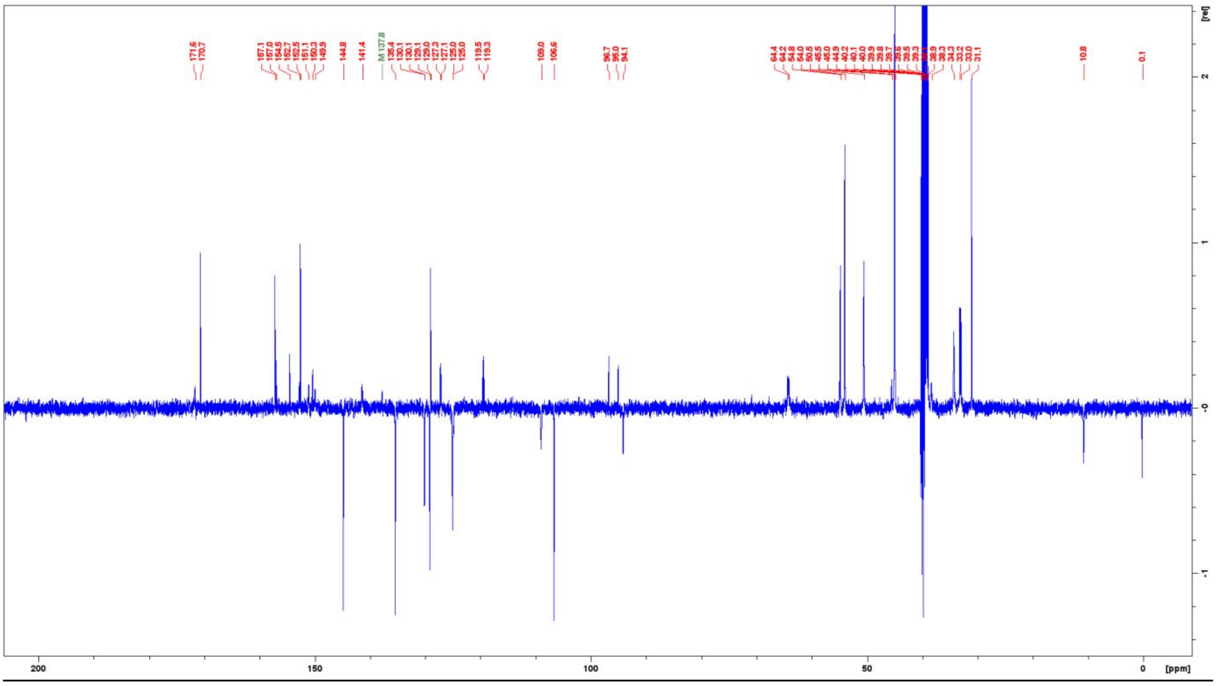

^{19}F NMR SK5527

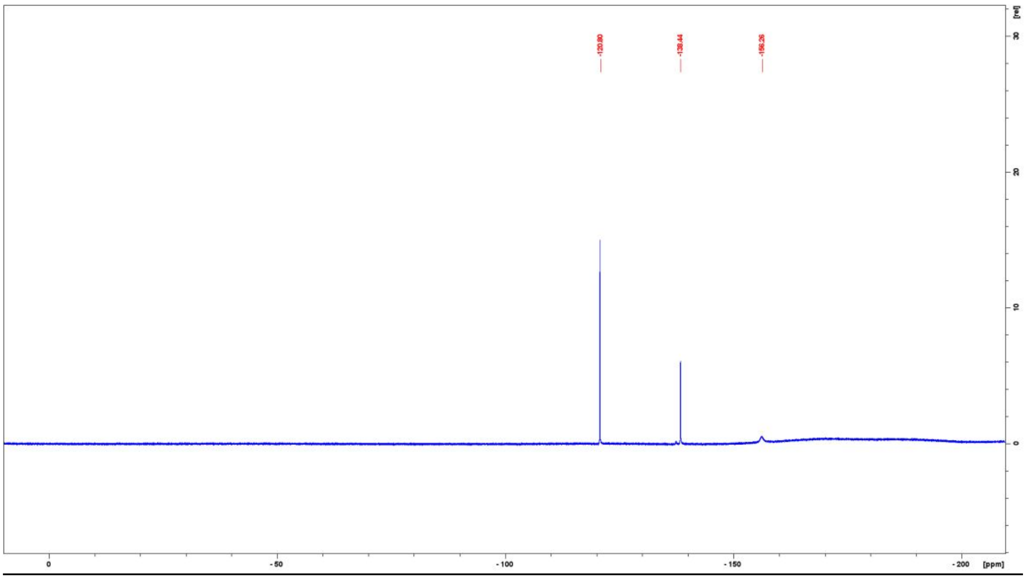

HRMS

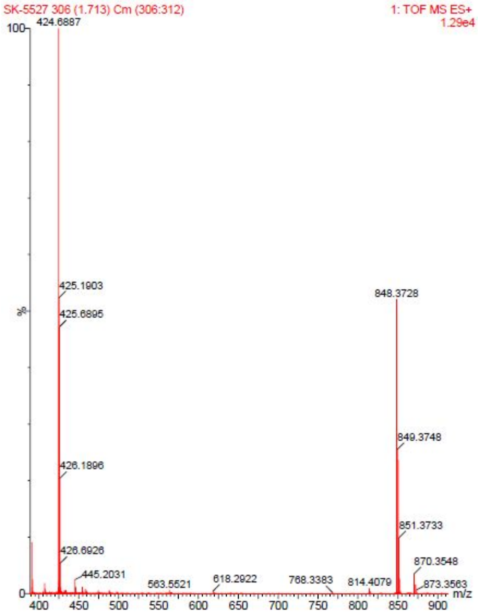

HPLC

Purity: 98%

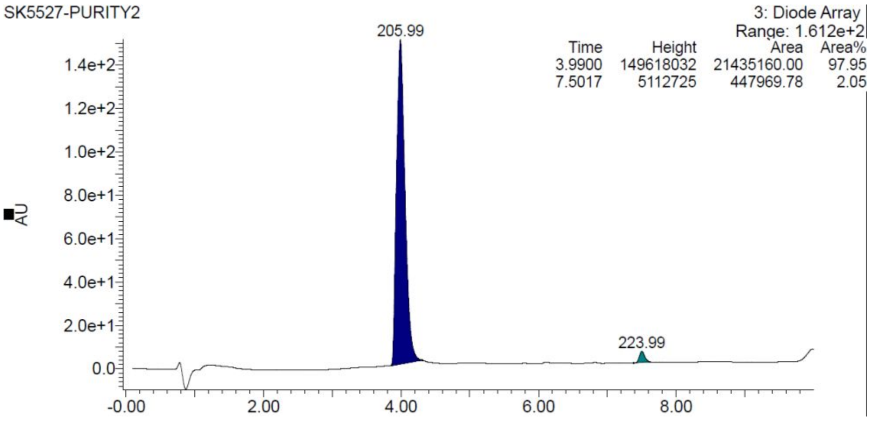

INACTIVE PROTAC SK5527-CRBi

^{1}H NMR (DMSO-d6)

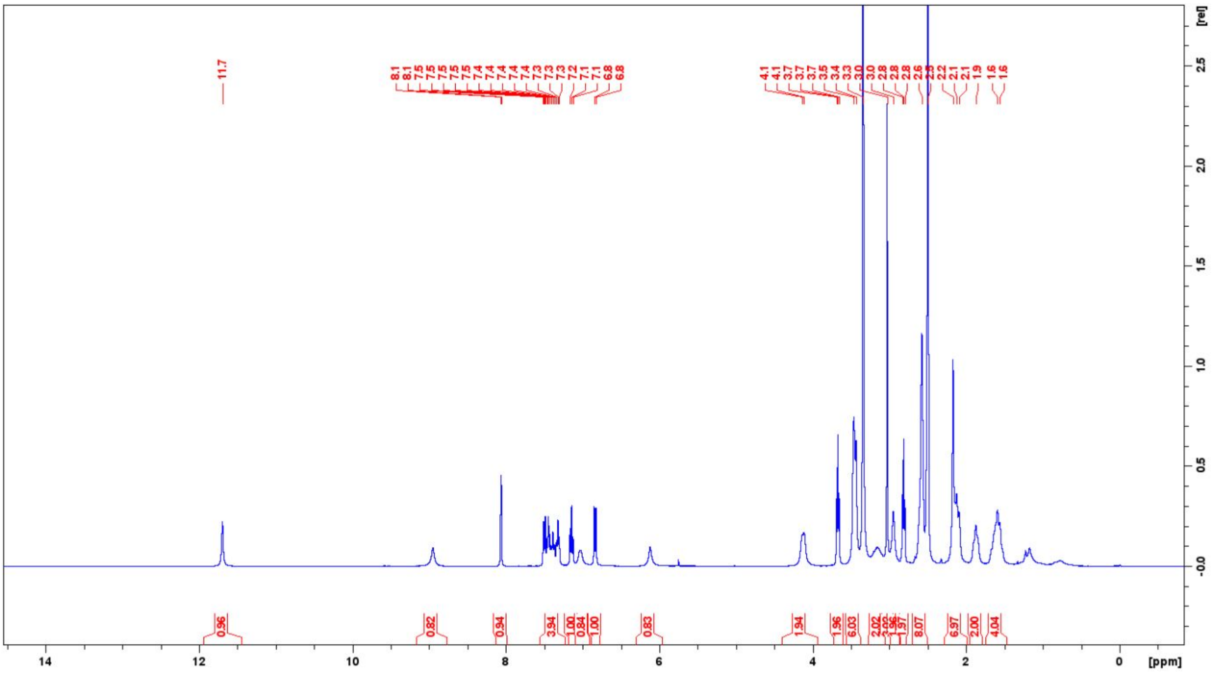

^{13}C NMR (DMSO-d6)

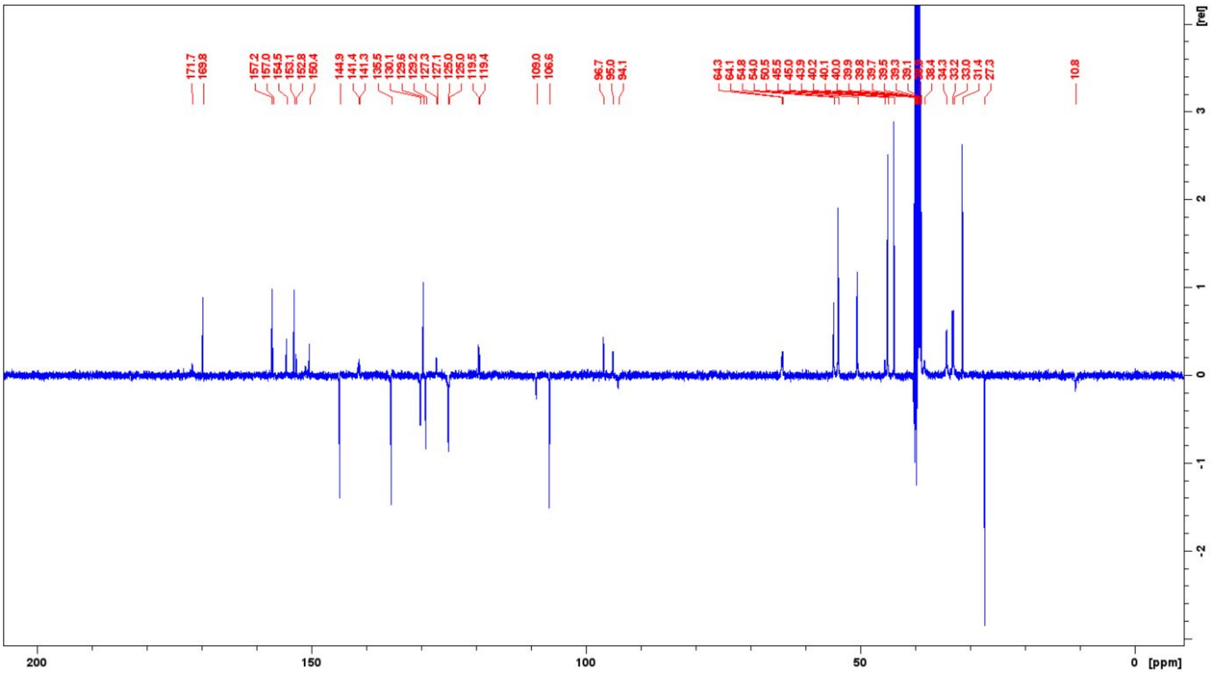

HPLC

Purity: > 99%

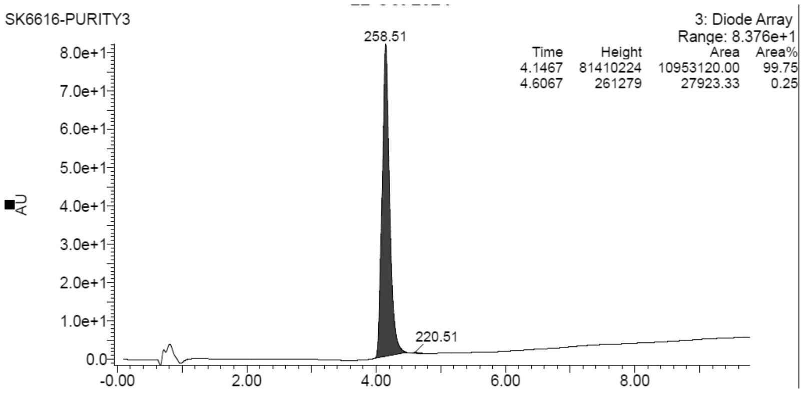

INACTIVE PROTAC SK5527-AURi

^{1}H NMR (DMSO-d6)

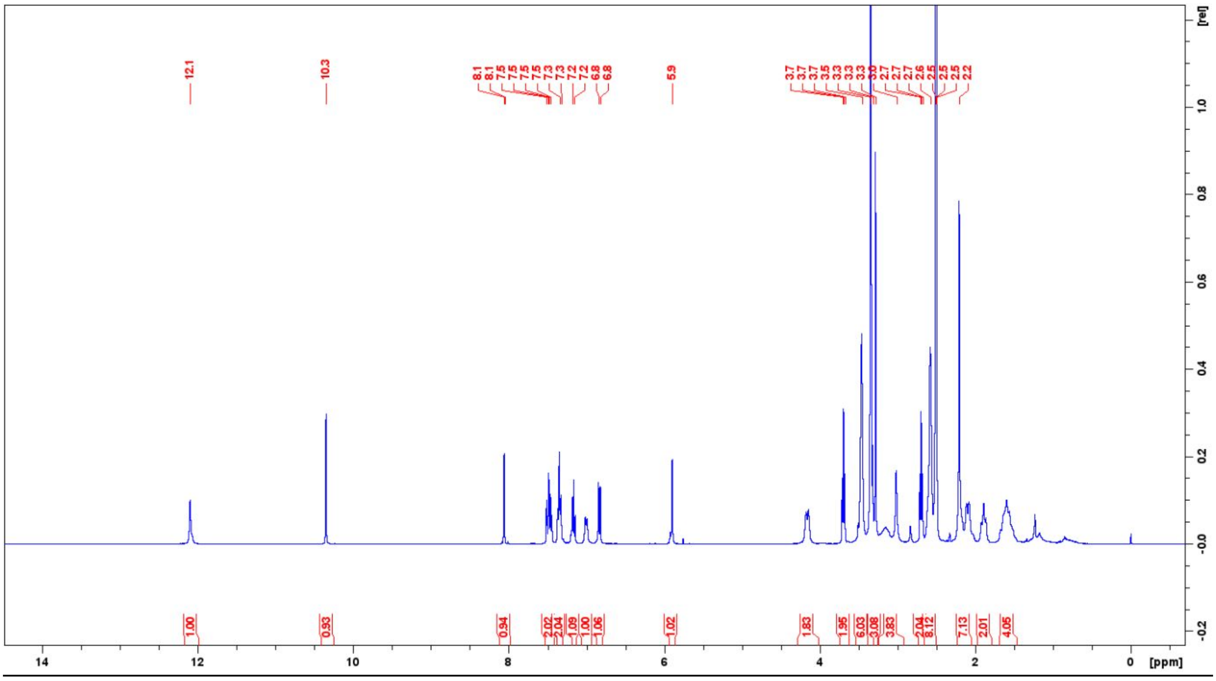

^{13}C NMR (DMSO-d6)

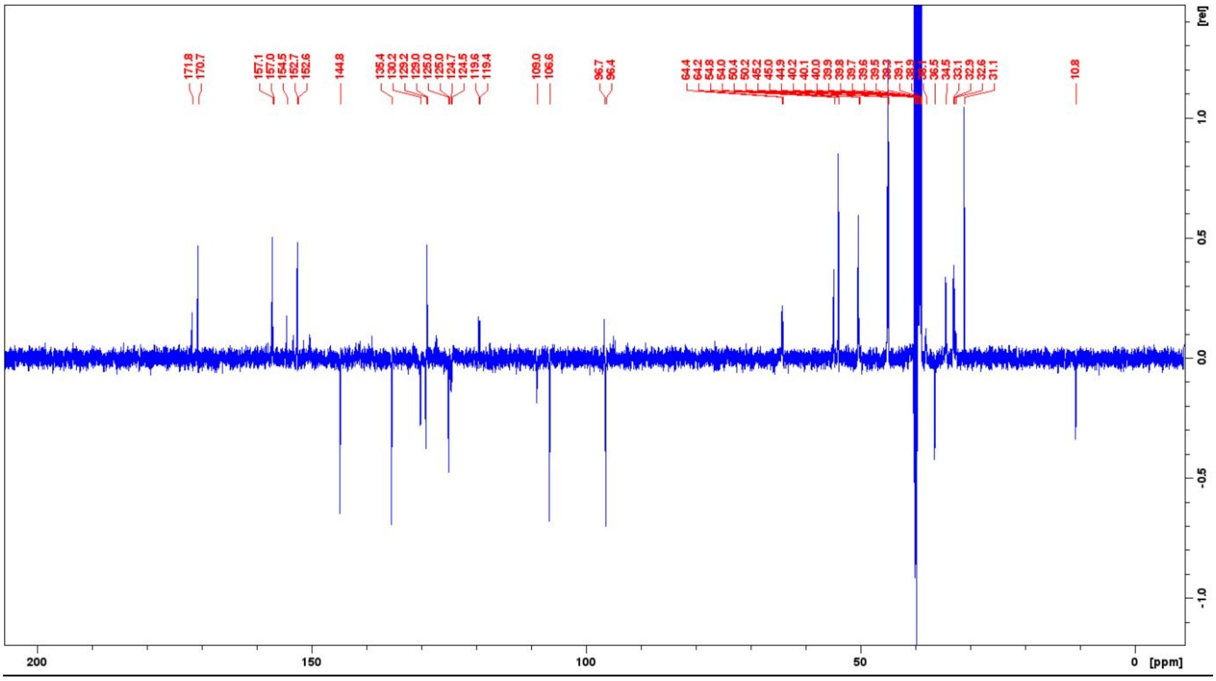

HPLC

Purity: > 99%

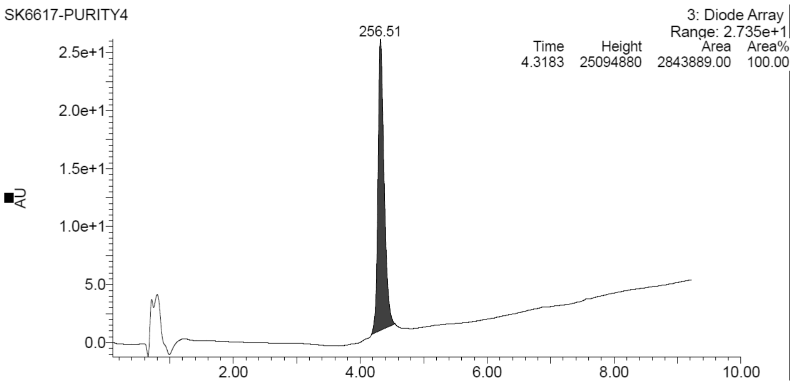

References

[1] M. Rishfi, S. Krols, F. Martens, S.L. Bekaert, E. Sanders, A. Eggermont, F. De Vloed, J.R. Goulding, M. Risseeuw, J. Molenaar, B. De Wilde, S. Van Calenbergh, K. Durinck, Targeted AURKA degradation: Towards new therapeutic agents for neuroblastoma, Eur J Med Chem 247 (2023). https://doi.org/10.1016/J.EJMECH.2022.115033.

[2] C. Chiva, R. Olivella, E. Borràs, G. Espadas, O. Pastor, A. Solé, E. Sabidó, QCloud: A cloud-based quality control system for mass spectrometry-based proteomics laboratories, PLoS One 13 (2018). https://doi.org/10.1371/JOURNAL.PONE.0189209.

[3] R. Olivella, C. Chiva, M. Serret, D. Mancera, L. Cozzuto, A. Hermoso, E. Borràs, G. Espadas, J. Morales, O. Pastor, A. Solé, J. Ponomarenko, E. Sabidó, QCloud2: An Improved Cloud-based Quality-Control System for Mass-Spectrometry-based Proteomics Laboratories, J Proteome Res 20 (2021) 2010–2013. https://doi.org/10.1021/ACS.JPROTEOME.0C00853.

[4] D. Mitchell, K.P. Cole, P.M. Pollock, D.M. Coppert, T.P. Burkholder, J.R. Clayton, Development and a Practical Synthesis of the JAK2 Inhibitor LY2784544, Org Process Res Dev 16 (2012) 70–81. https://doi.org/10.1021/op200229j.
